# Supplementary figures and images for: Fecal transplant from myostatin deletion pigs positively impacts the gut-muscle axis (part 1 of 2)
Source: eLife. 2023 Apr 11;12:e81858. doi: 10.7554/eLife.81858 (PMC10121221; doi:10.7554/eLife.81858)

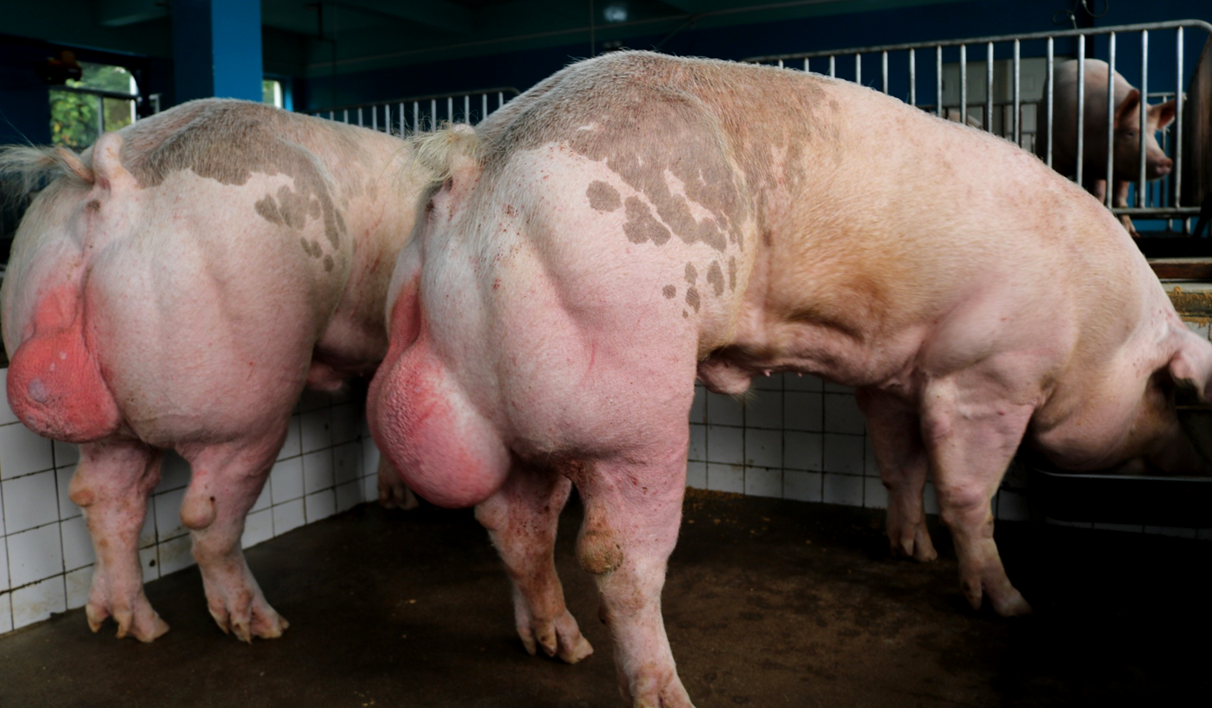

Supplement: Figure 1—source data 1. [file elife-81858-fig1-data1.zip › Figure 1-source data 1/fig1.a/MSTN-KO pig.tif]

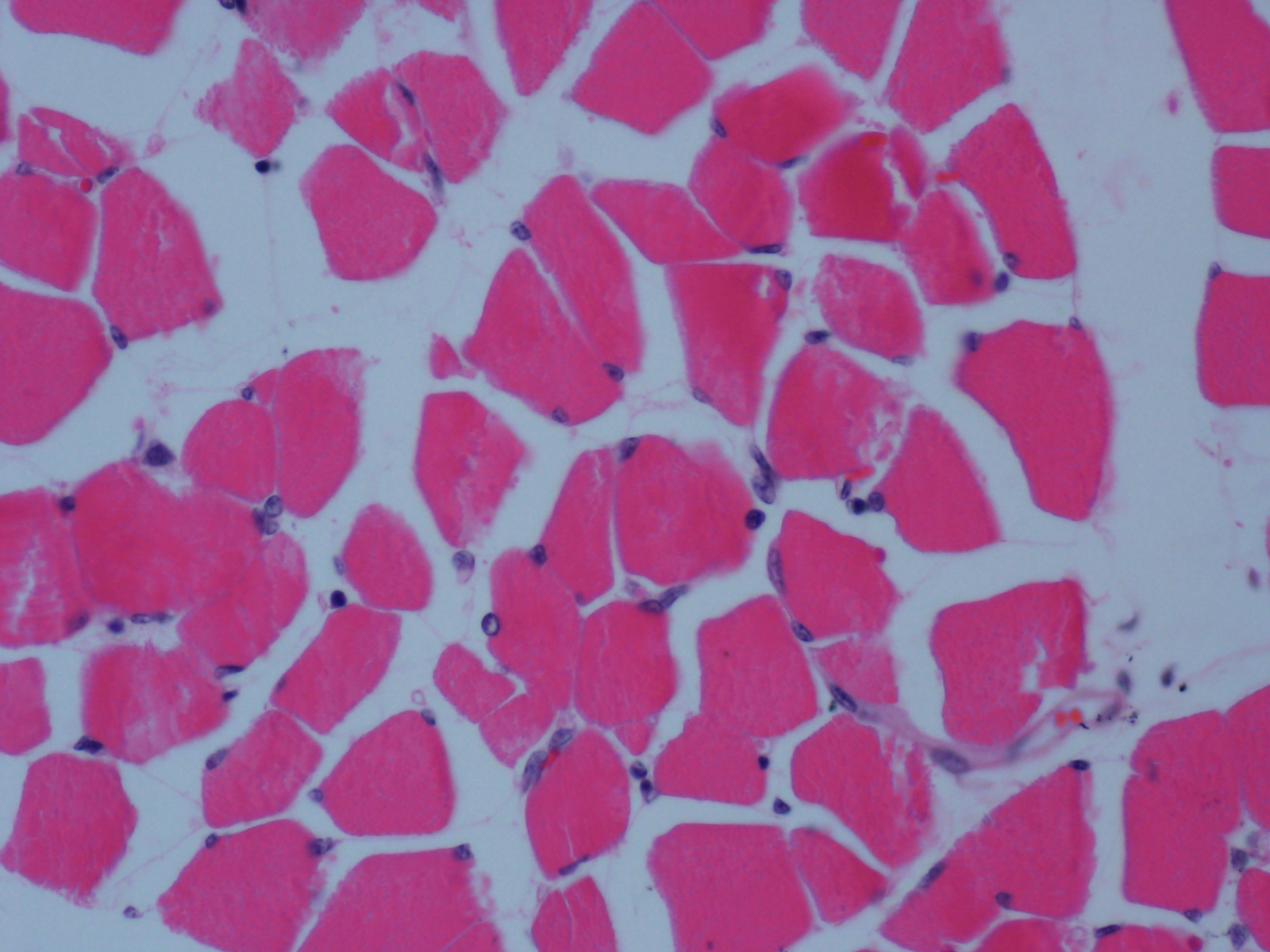

Supplement: Figure 1—source data 1. [file elife-81858-fig1-data1.zip › Figure 1-source data 1/fig1.b/MSTN-KO/KO (1).tif]

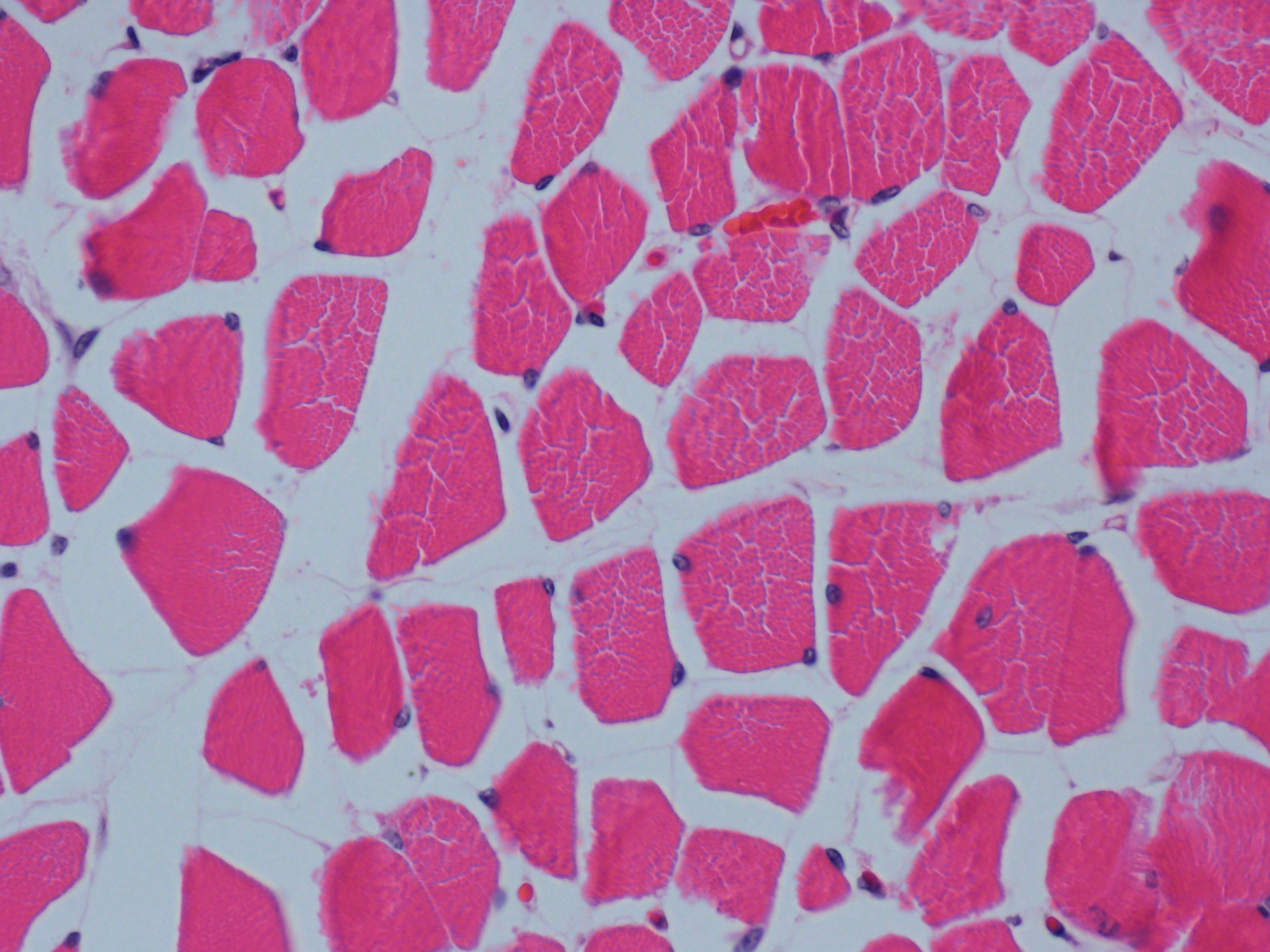

Supplement: Figure 1—source data 1. [file elife-81858-fig1-data1.zip › Figure 1-source data 1/fig1.b/MSTN-KO/KO (2).tif]

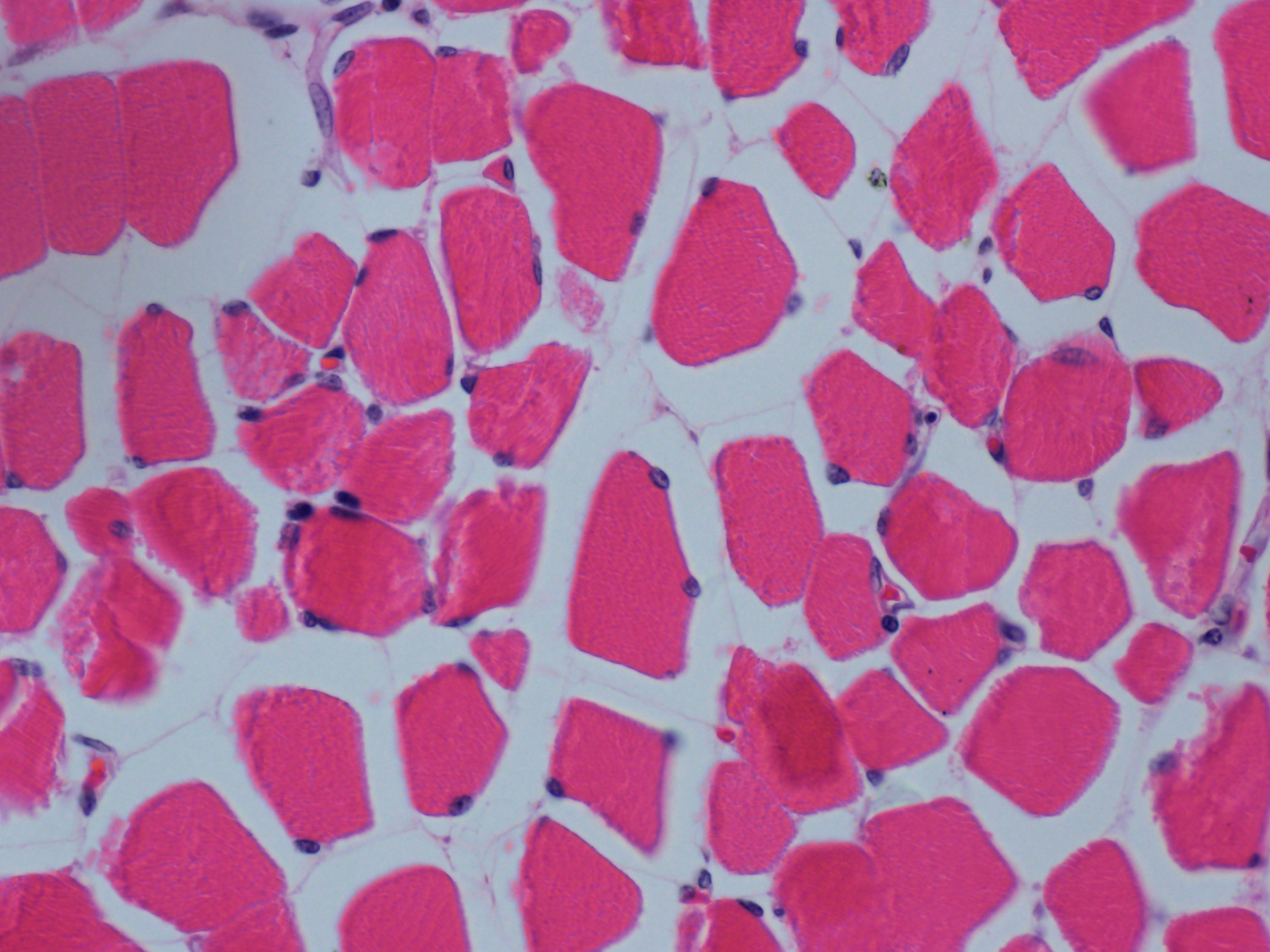

Supplement: Figure 1—source data 1. [file elife-81858-fig1-data1.zip › Figure 1-source data 1/fig1.b/MSTN-KO/KO (3).tif]

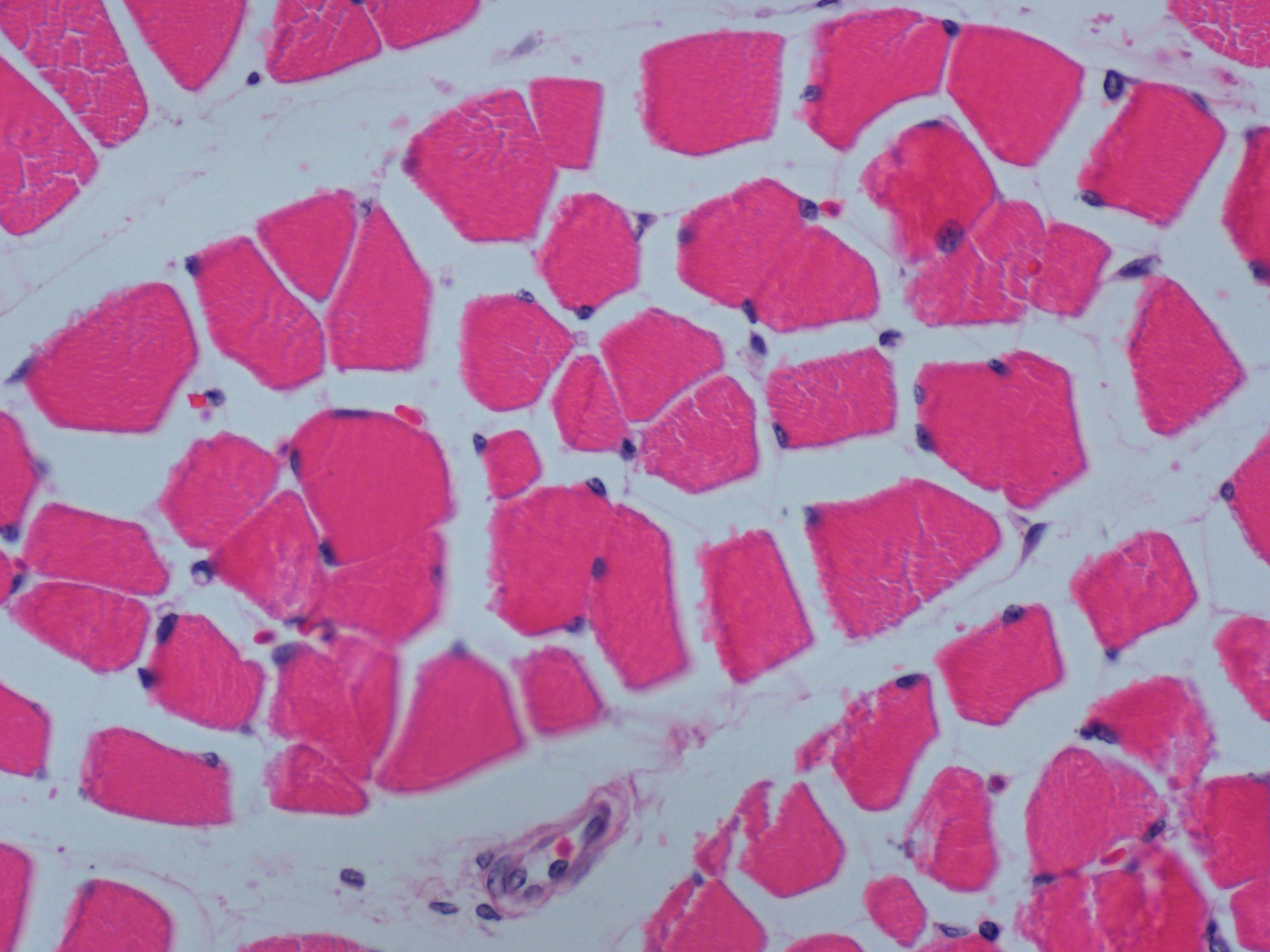

Supplement: Figure 1—source data 1. [file elife-81858-fig1-data1.zip › Figure 1-source data 1/fig1.b/MSTN-KO/KO (4).tif]

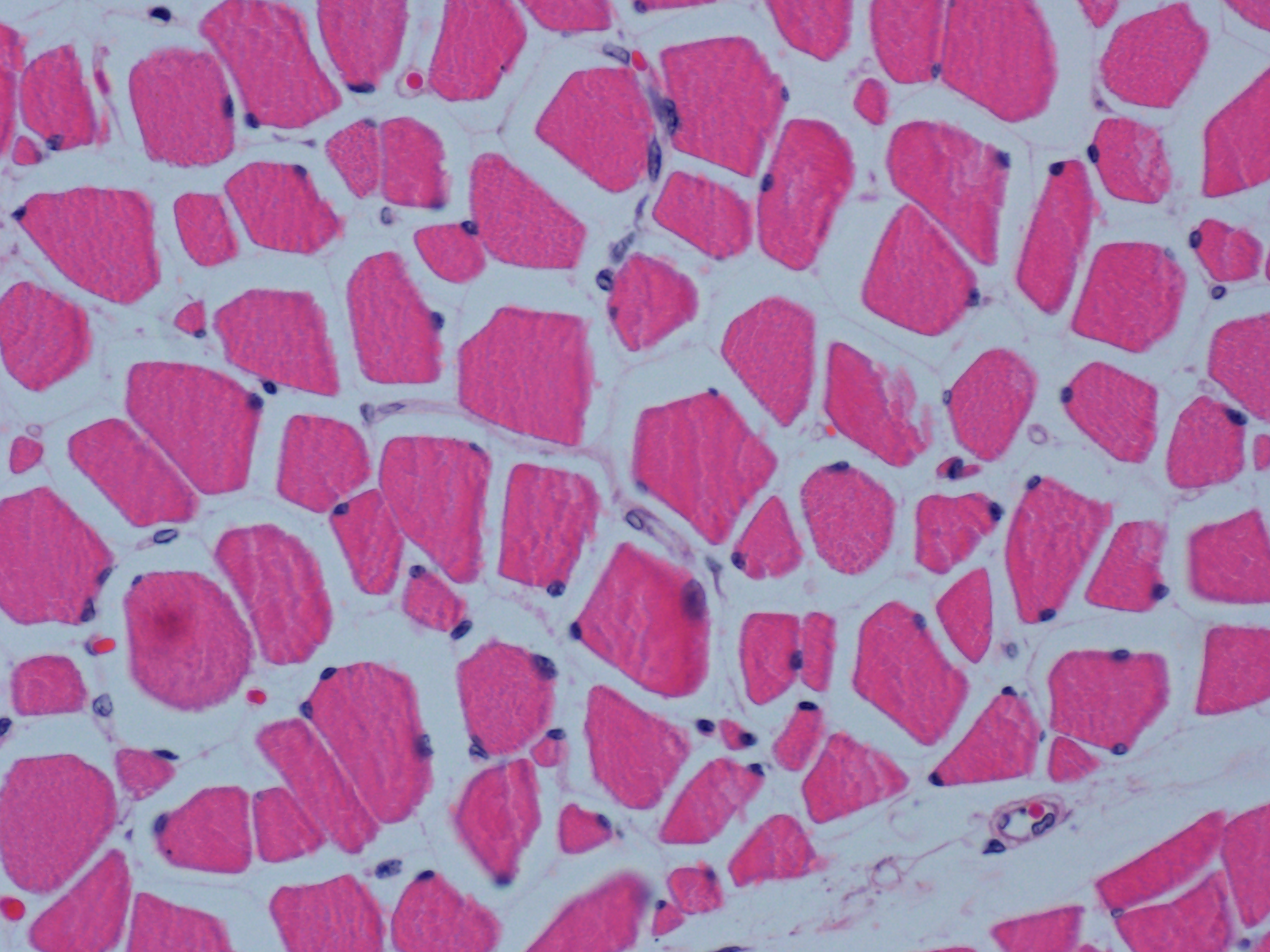

Supplement: Figure 1—source data 1. [file elife-81858-fig1-data1.zip › Figure 1-source data 1/fig1.b/WT/WT (1).tif]

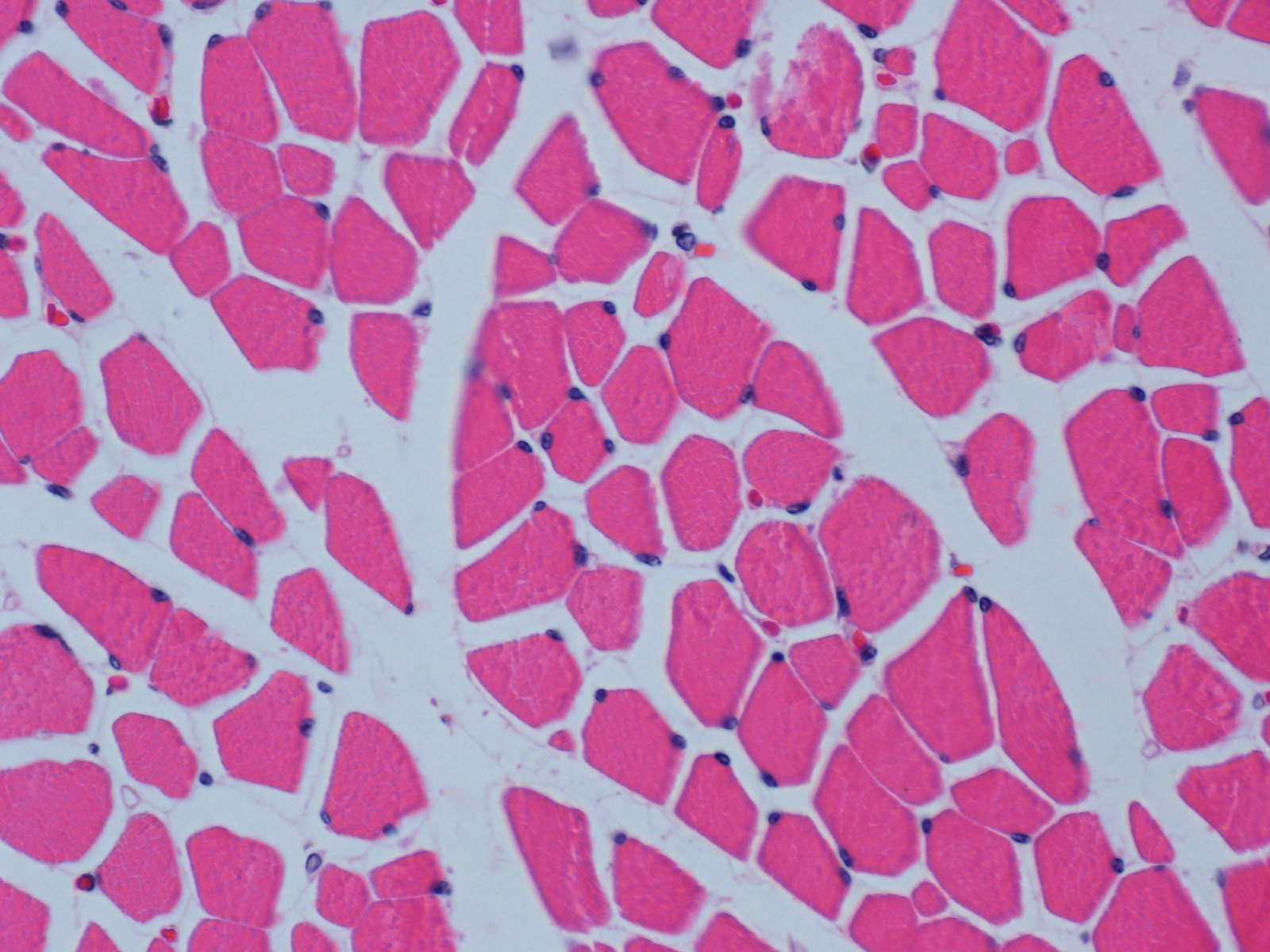

Supplement: Figure 1—source data 1. [file elife-81858-fig1-data1.zip › Figure 1-source data 1/fig1.b/WT/WT (2).tif]

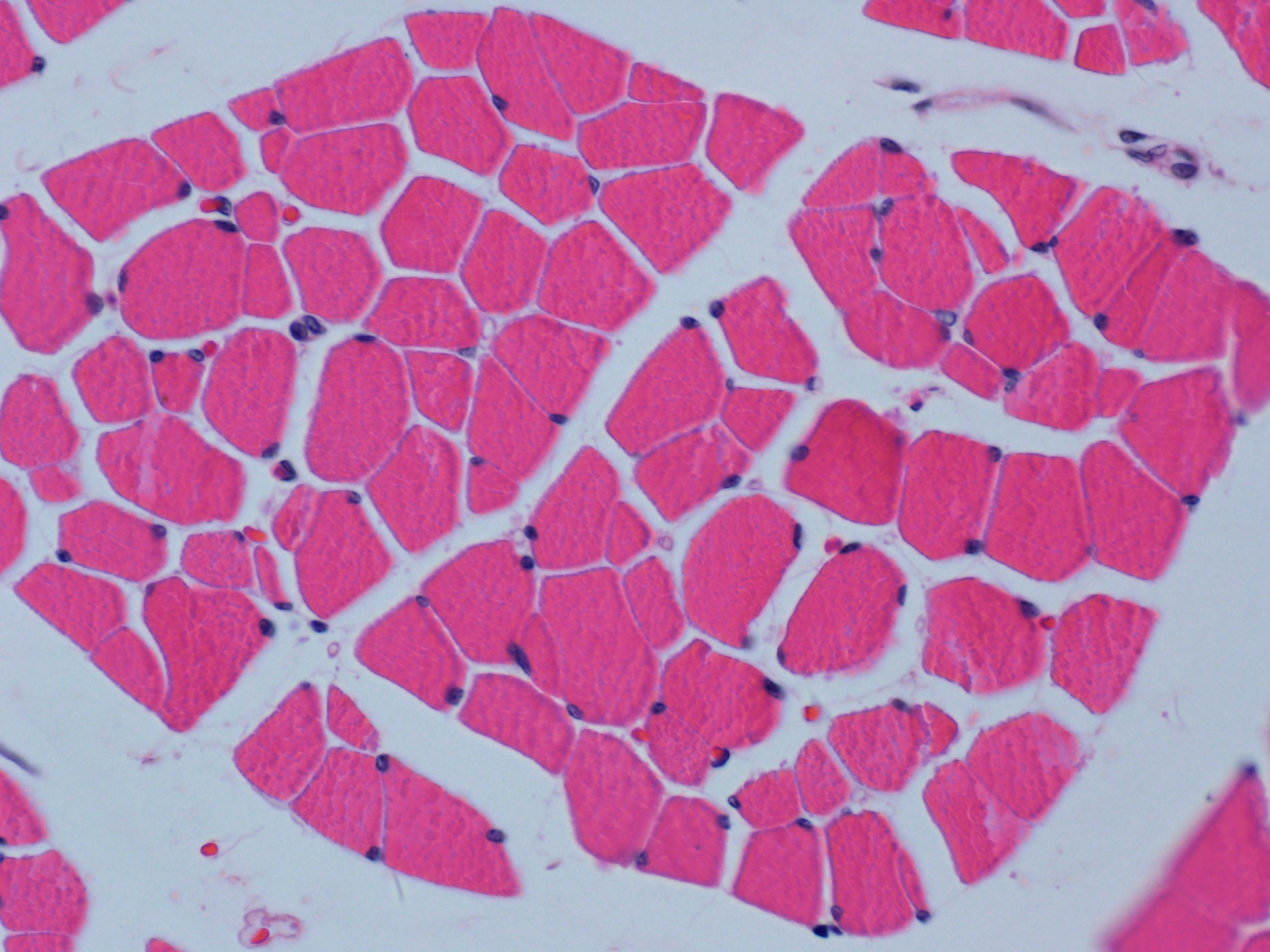

Supplement: Figure 1—source data 1. [file elife-81858-fig1-data1.zip › Figure 1-source data 1/fig1.b/WT/WT (3).tif]

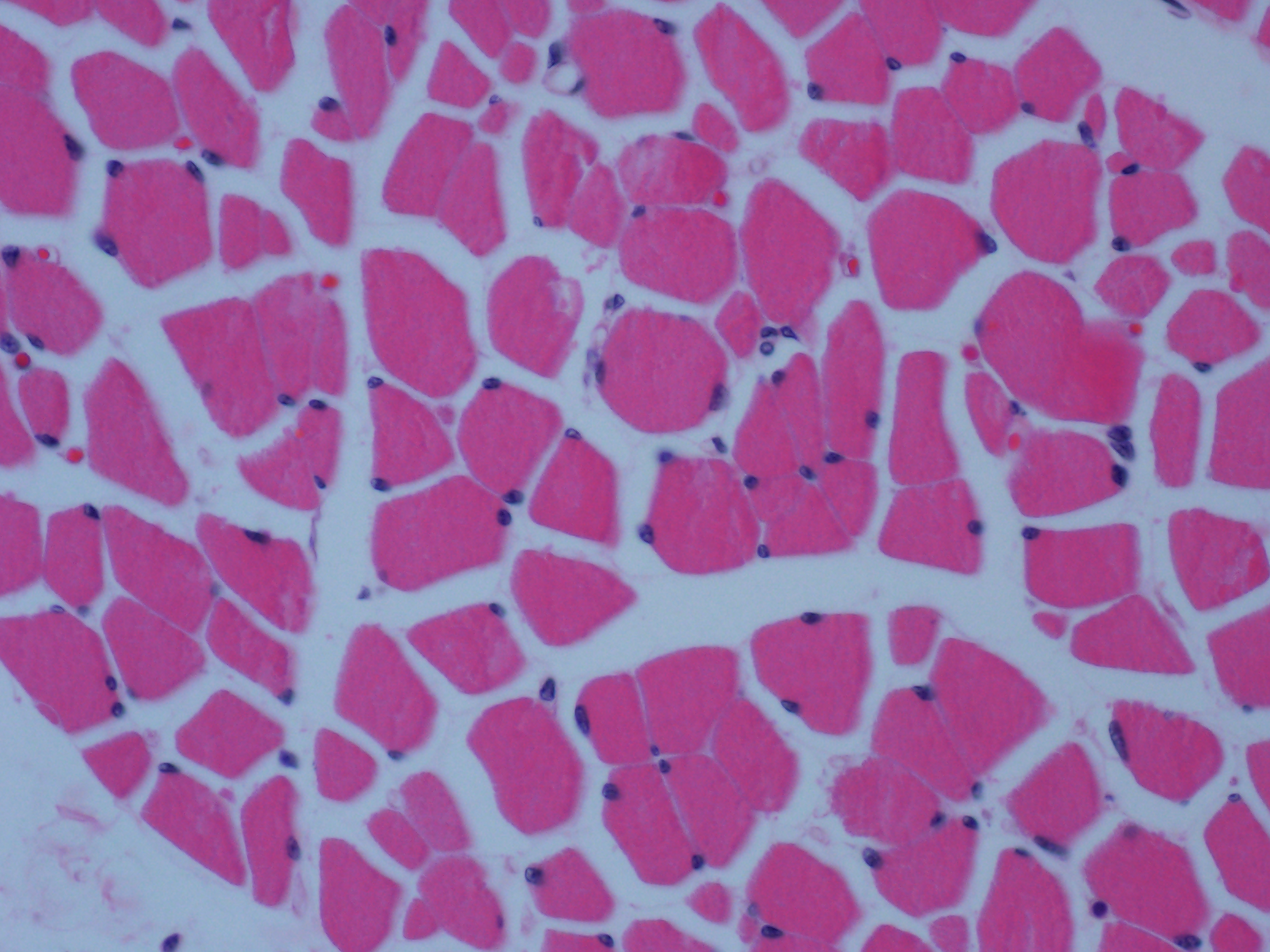

Supplement: Figure 1—source data 1. [file elife-81858-fig1-data1.zip › Figure 1-source data 1/fig1.b/WT/WT (4).tif]

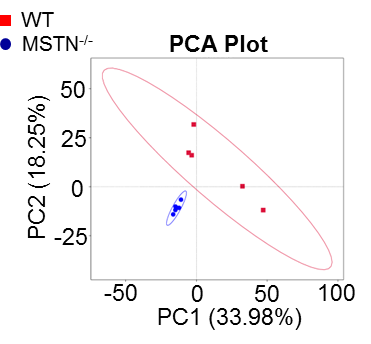

Supplement: Figure 1—source data 1. [file elife-81858-fig1-data1.zip › Figure 1-source data 1/fig1.e/pig-PCA-WTvs.MSTN.tif]

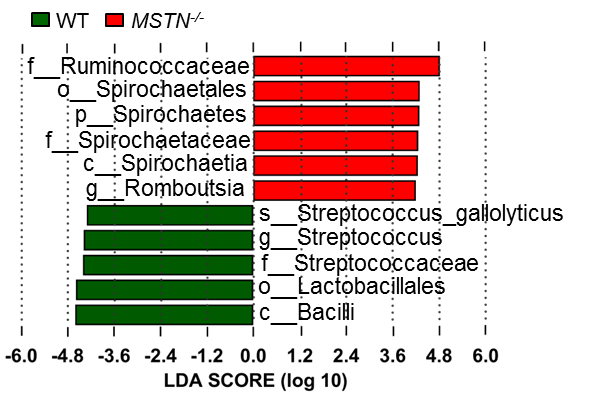

Supplement: Figure 1—source data 1. [file elife-81858-fig1-data1.zip › Figure 1-source data 1/fig1.f/Pig-LDA-WTvs.MSTN.tif]

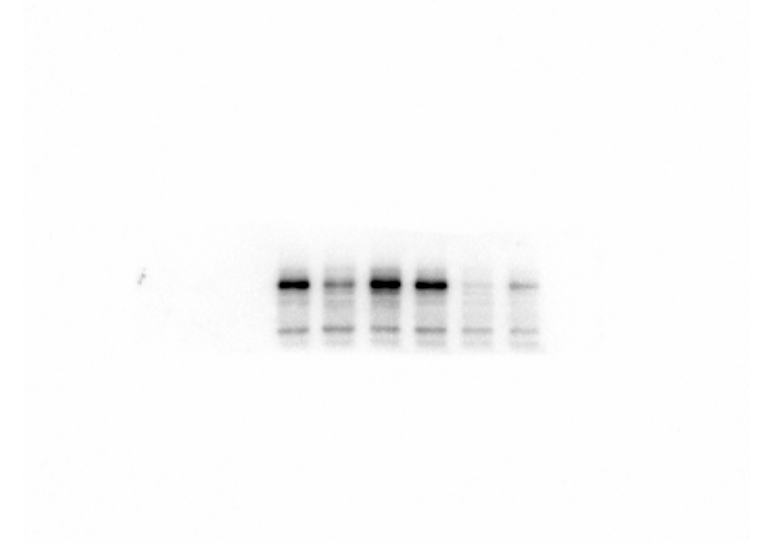

Supplement: Figure 1—source data 2. [file elife-81858-fig1-data2.zip › Figure 1-source data 2/fig1c.MSTN.tif]

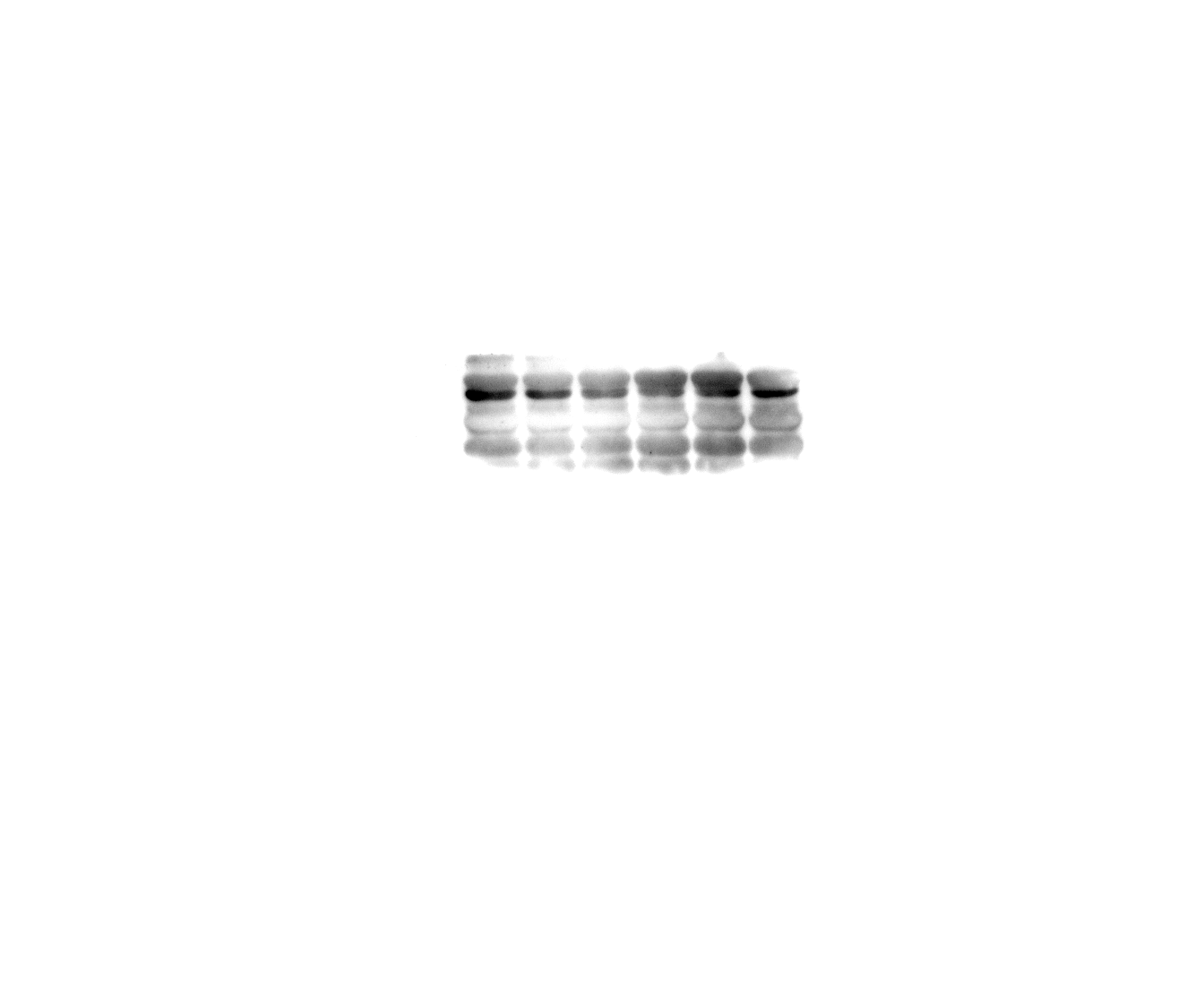

Supplement: Figure 1—source data 2. [file elife-81858-fig1-data2.zip › Figure 1-source data 2/fig1c.MyhcI.tif]

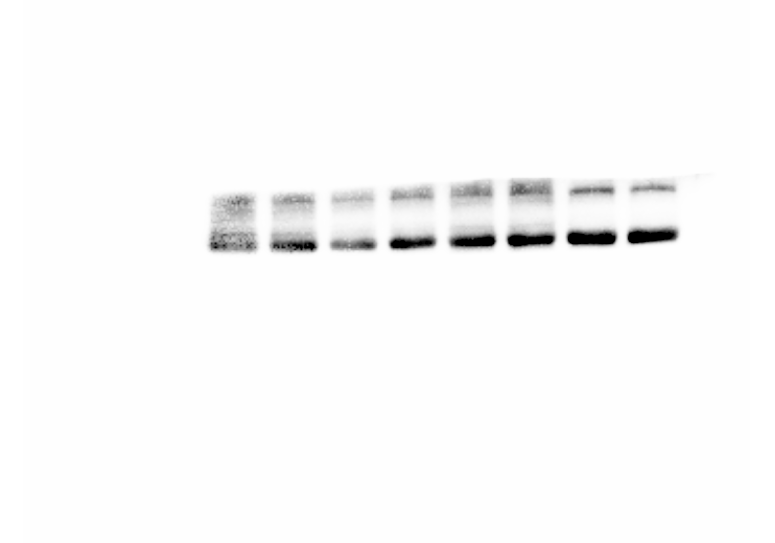

Supplement: Figure 1—source data 2. [file elife-81858-fig1-data2.zip › Figure 1-source data 2/fig1c.MyhcIIa.tif]

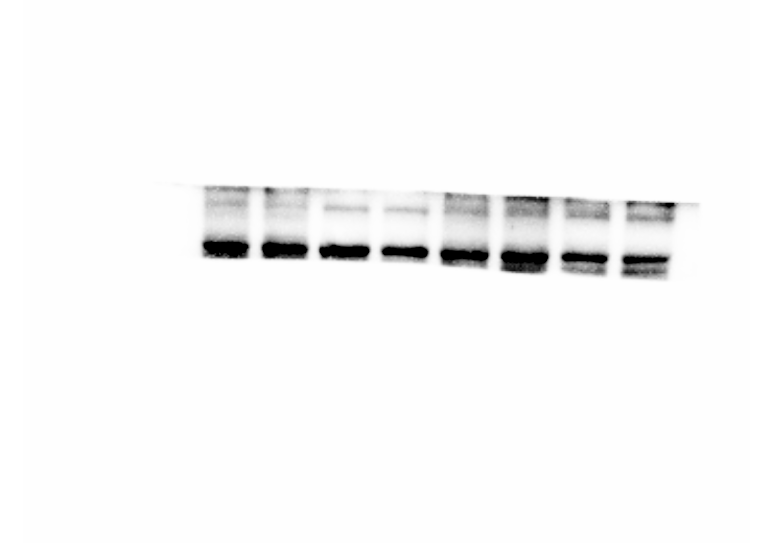

Supplement: Figure 1—source data 2. [file elife-81858-fig1-data2.zip › Figure 1-source data 2/fig1c.MyhcIIb.tif]

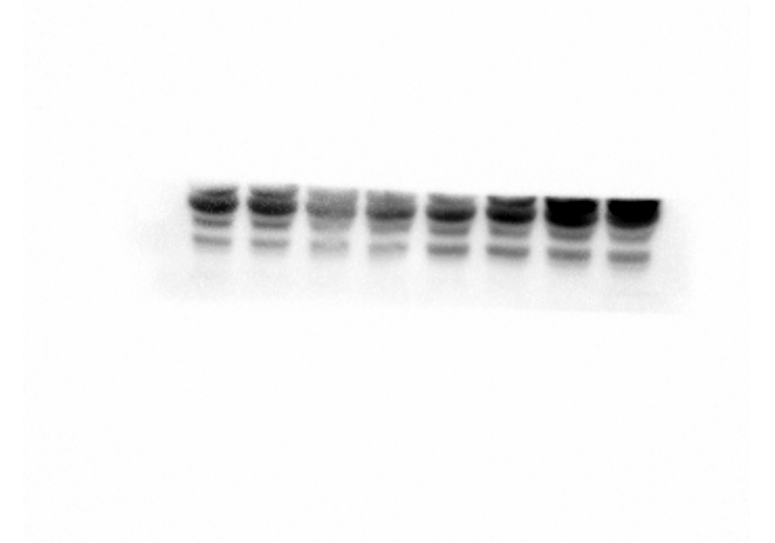

Supplement: Figure 1—source data 2. [file elife-81858-fig1-data2.zip › Figure 1-source data 2/fig1c.Myod.tif]

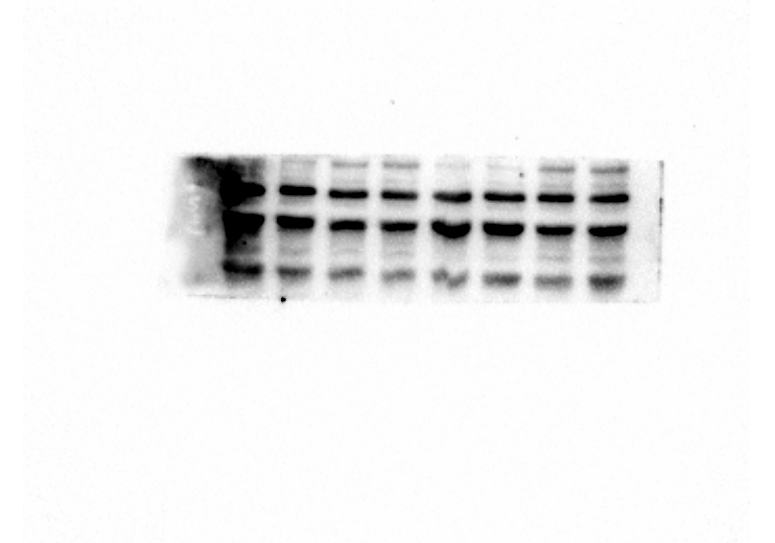

Supplement: Figure 1—source data 2. [file elife-81858-fig1-data2.zip › Figure 1-source data 2/fig1c.Smad23.tif]

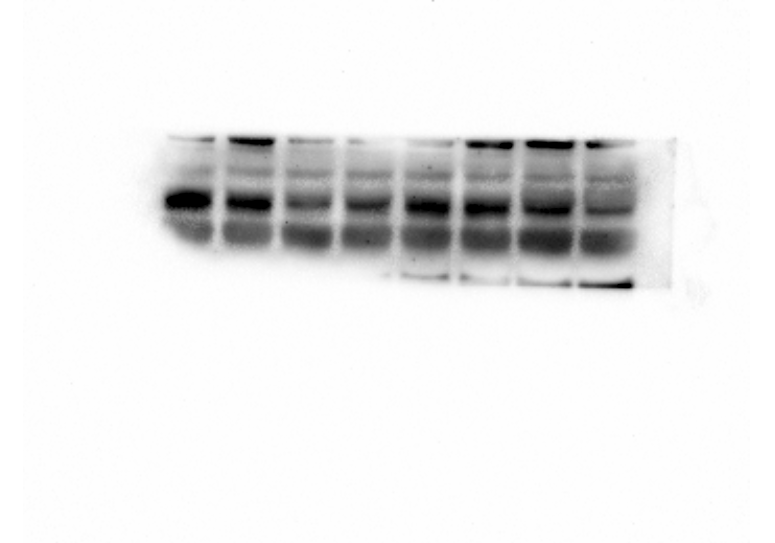

Supplement: Figure 1—source data 2. [file elife-81858-fig1-data2.zip › Figure 1-source data 2/fig1c.p-Smad23.tif]

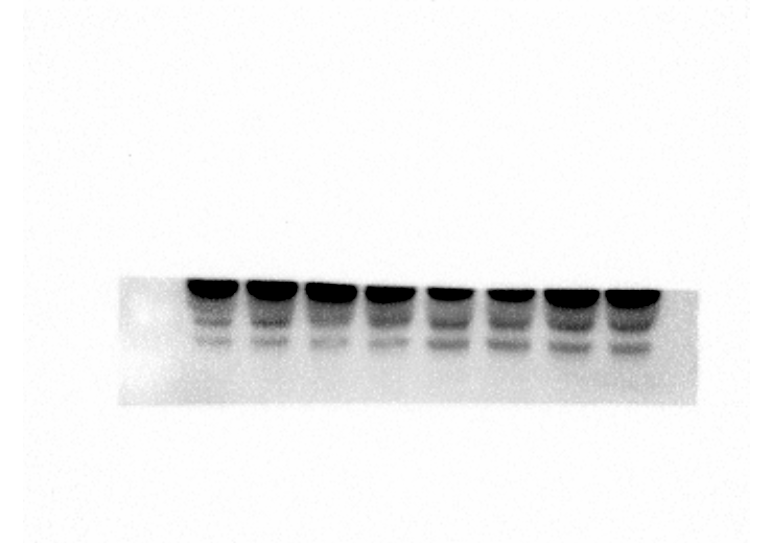

Supplement: Figure 1—source data 2. [file elife-81858-fig1-data2.zip › Figure 1-source data 2/fig1c.tublin.tif]

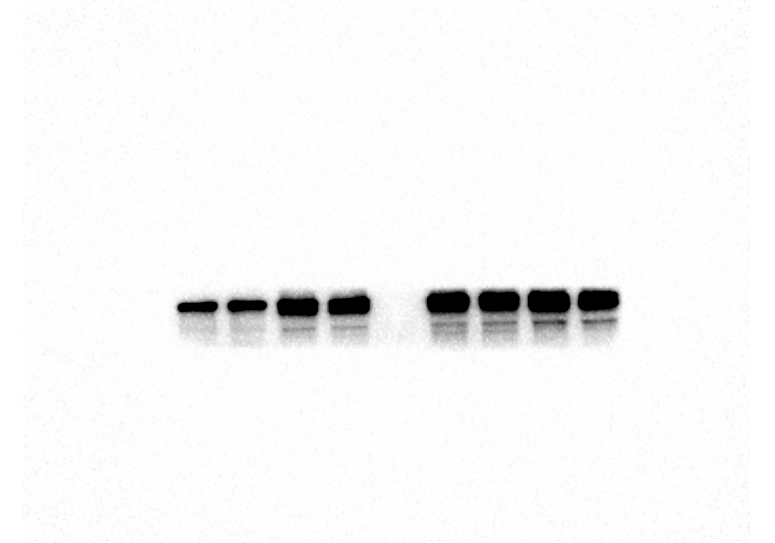

Supplement: Figure 1—source data 2. [file elife-81858-fig1-data2.zip › Figure 1-source data 2/fig1d.HK2.tif]

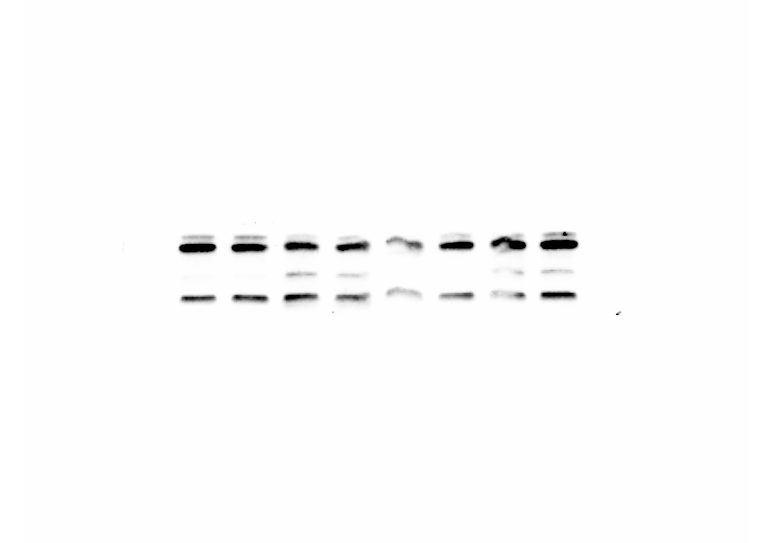

Supplement: Figure 1—source data 2. [file elife-81858-fig1-data2.zip › Figure 1-source data 2/fig1d.PFK1.tif]

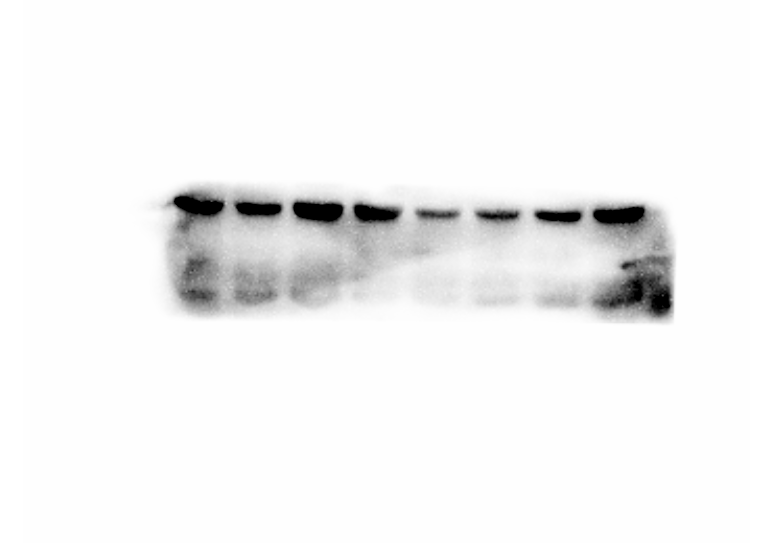

Supplement: Figure 1—source data 2. [file elife-81858-fig1-data2.zip › Figure 1-source data 2/fig1d.PKM2.tif]

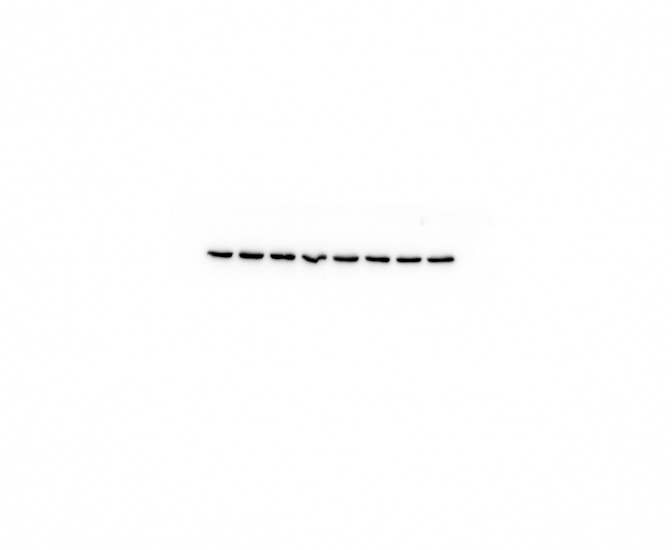

Supplement: Figure 1—source data 2. [file elife-81858-fig1-data2.zip › Figure 1-source data 2/fig1d.actin.tif]

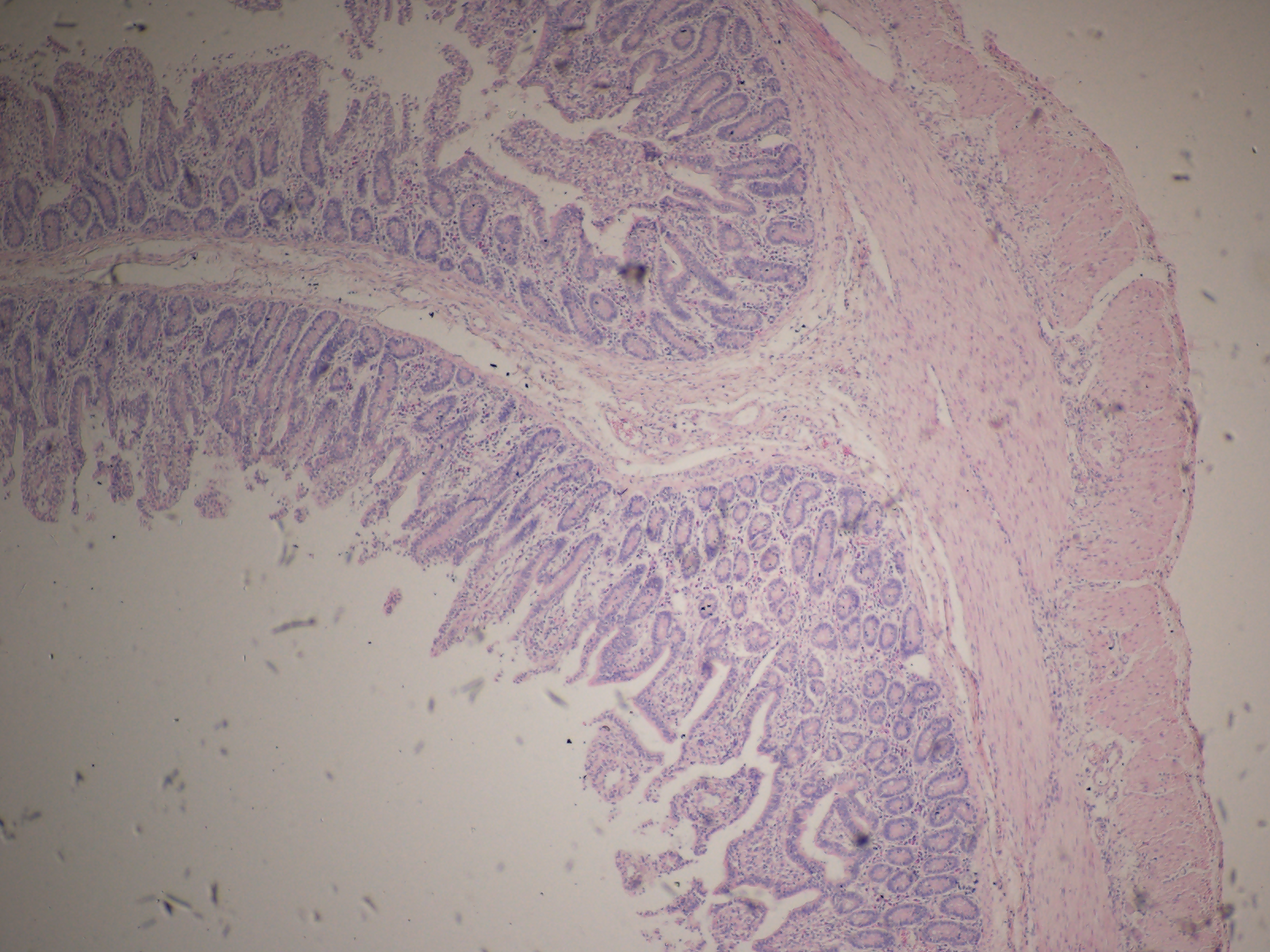

Supplement: Figure 2—source data 1. [file elife-81858-fig2-data1.zip › Figure 2-source data 1/fig2.a/MSTN KO/KO (1).tif]

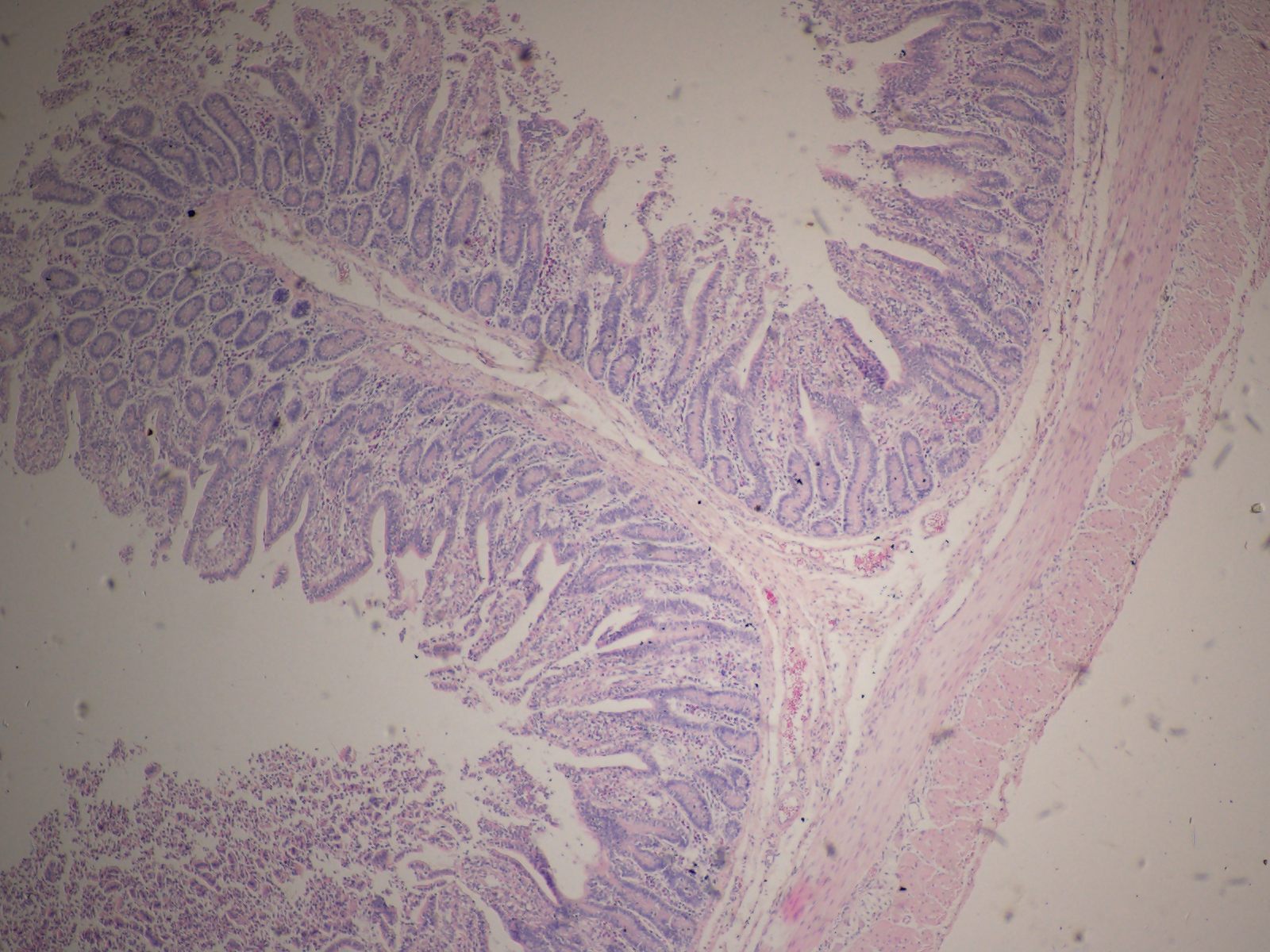

Supplement: Figure 2—source data 1. [file elife-81858-fig2-data1.zip › Figure 2-source data 1/fig2.a/MSTN KO/KO (2).tif]

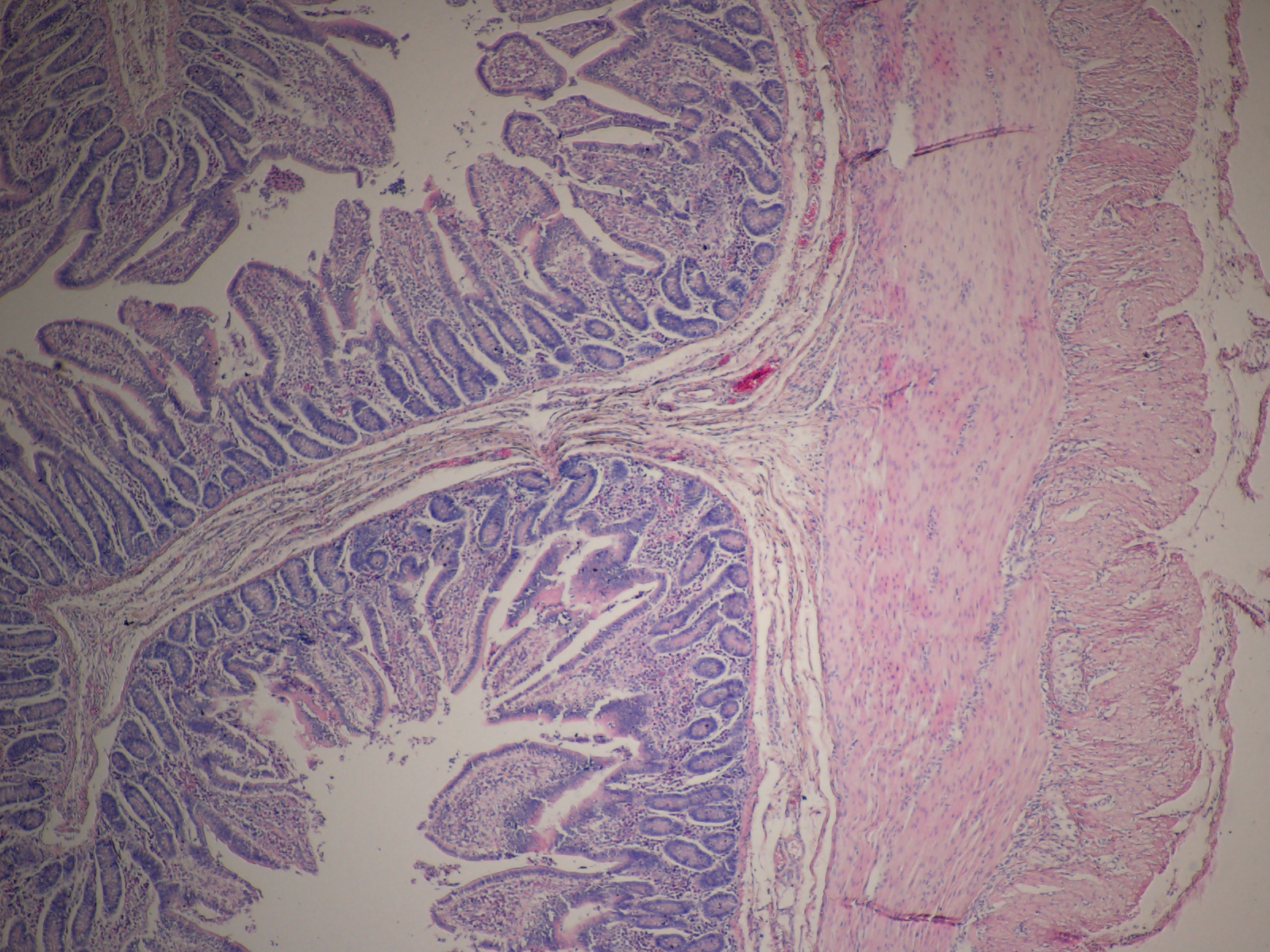

Supplement: Figure 2—source data 1. [file elife-81858-fig2-data1.zip › Figure 2-source data 1/fig2.a/MSTN KO/KO (3).tif]

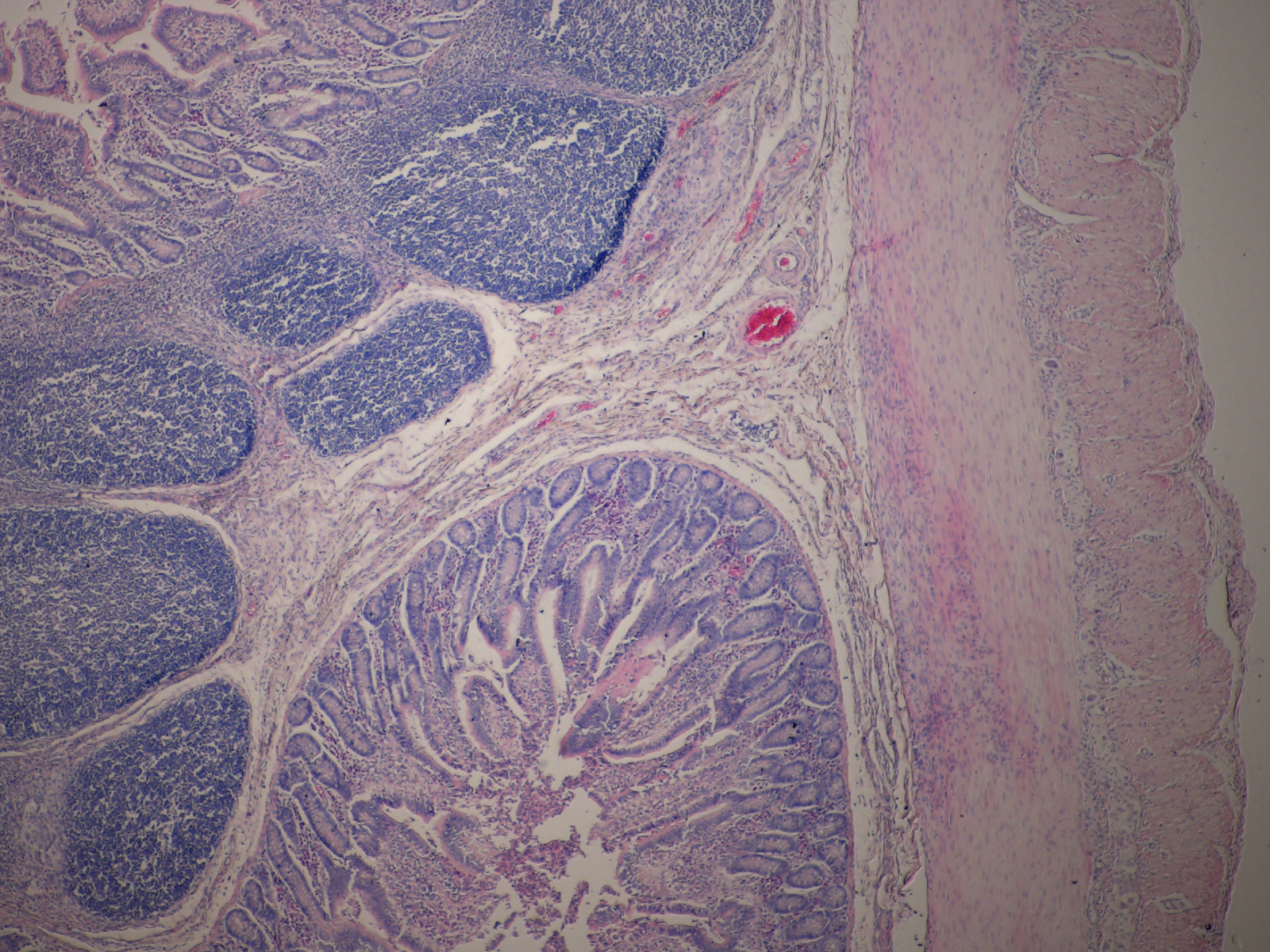

Supplement: Figure 2—source data 1. [file elife-81858-fig2-data1.zip › Figure 2-source data 1/fig2.a/MSTN KO/KO (4).tif]

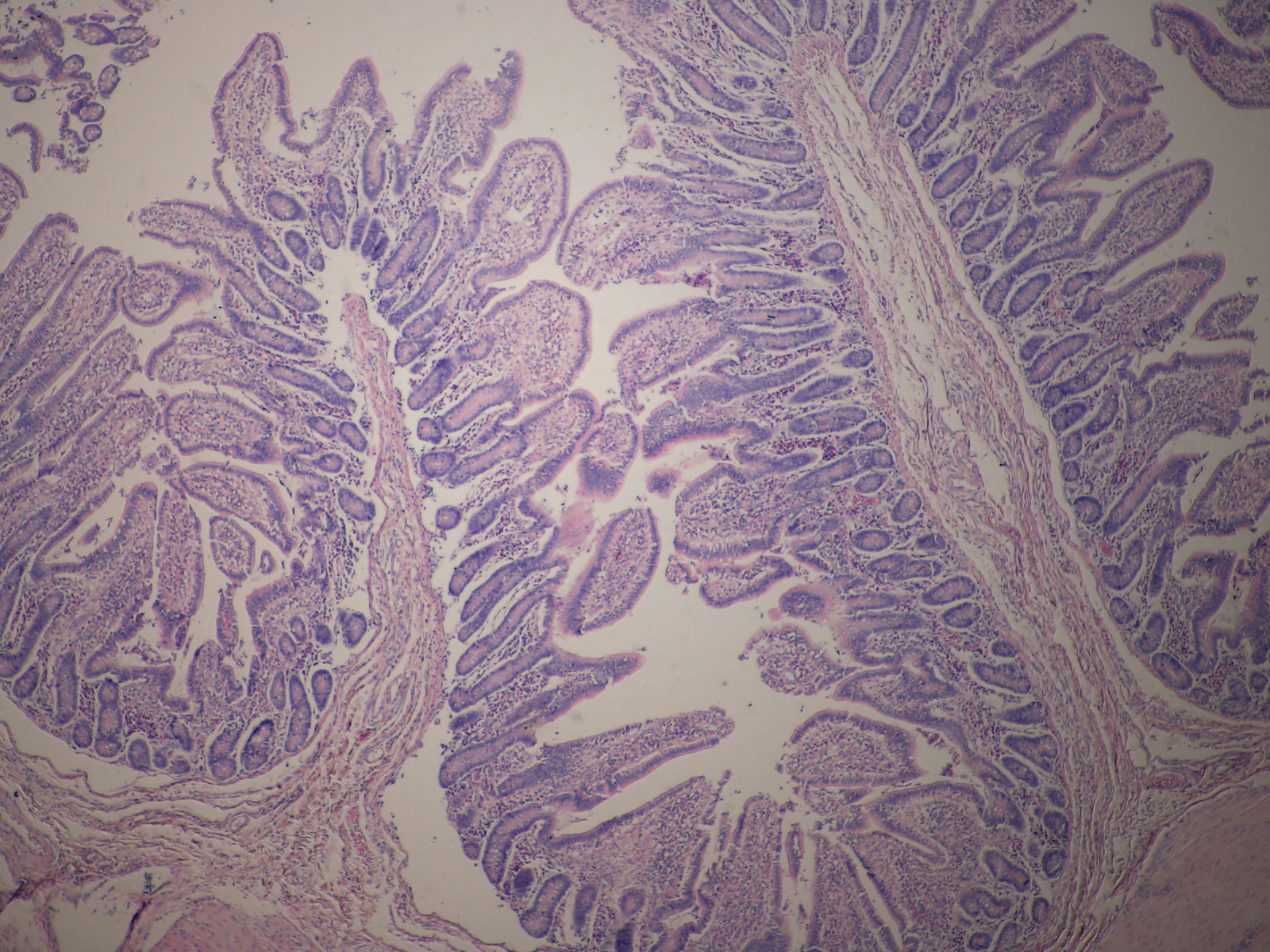

Supplement: Figure 2—source data 1. [file elife-81858-fig2-data1.zip › Figure 2-source data 1/fig2.a/MSTN KO/KO (5).tif]

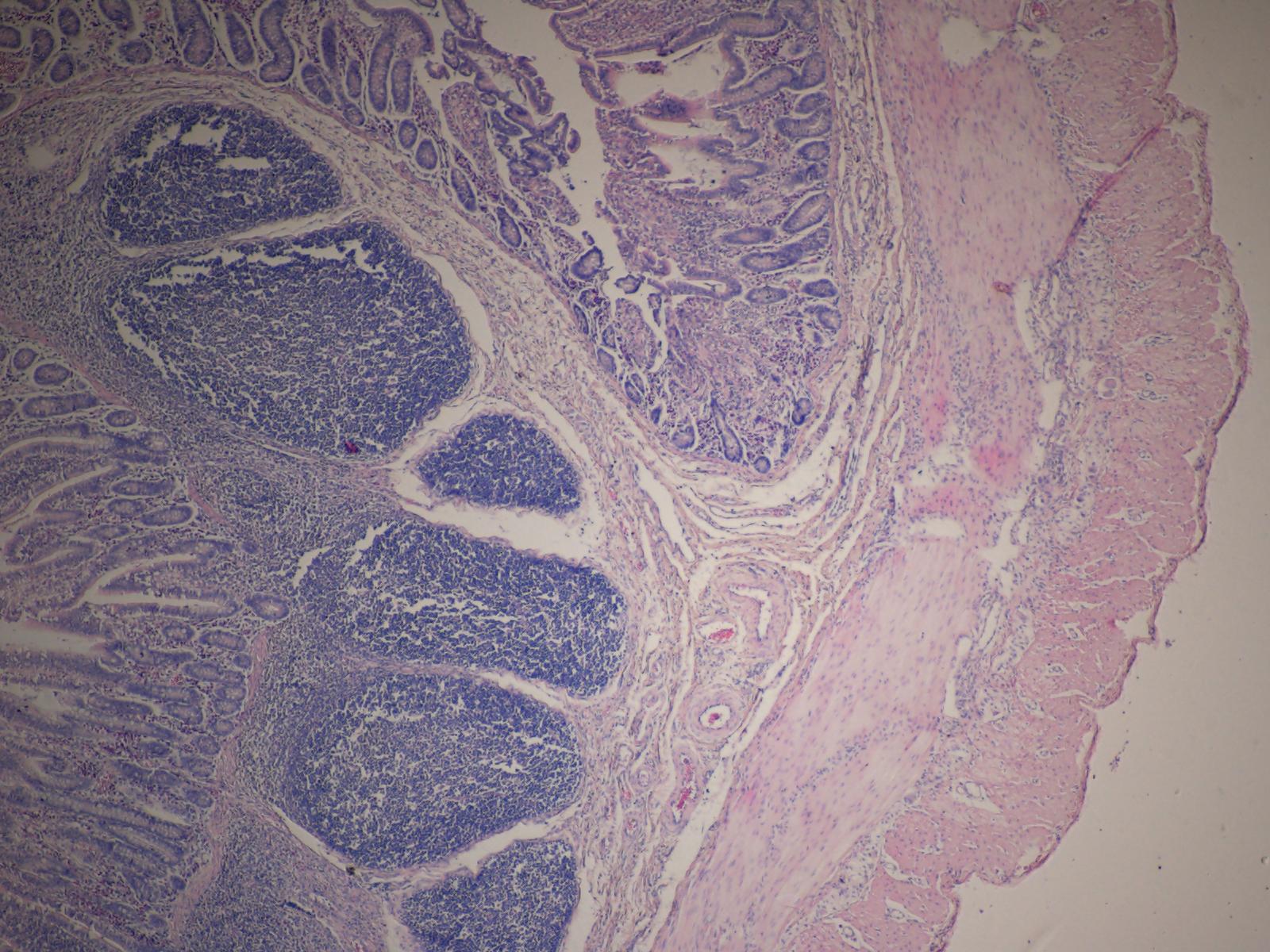

Supplement: Figure 2—source data 1. [file elife-81858-fig2-data1.zip › Figure 2-source data 1/fig2.a/MSTN KO/KO (6).tif]

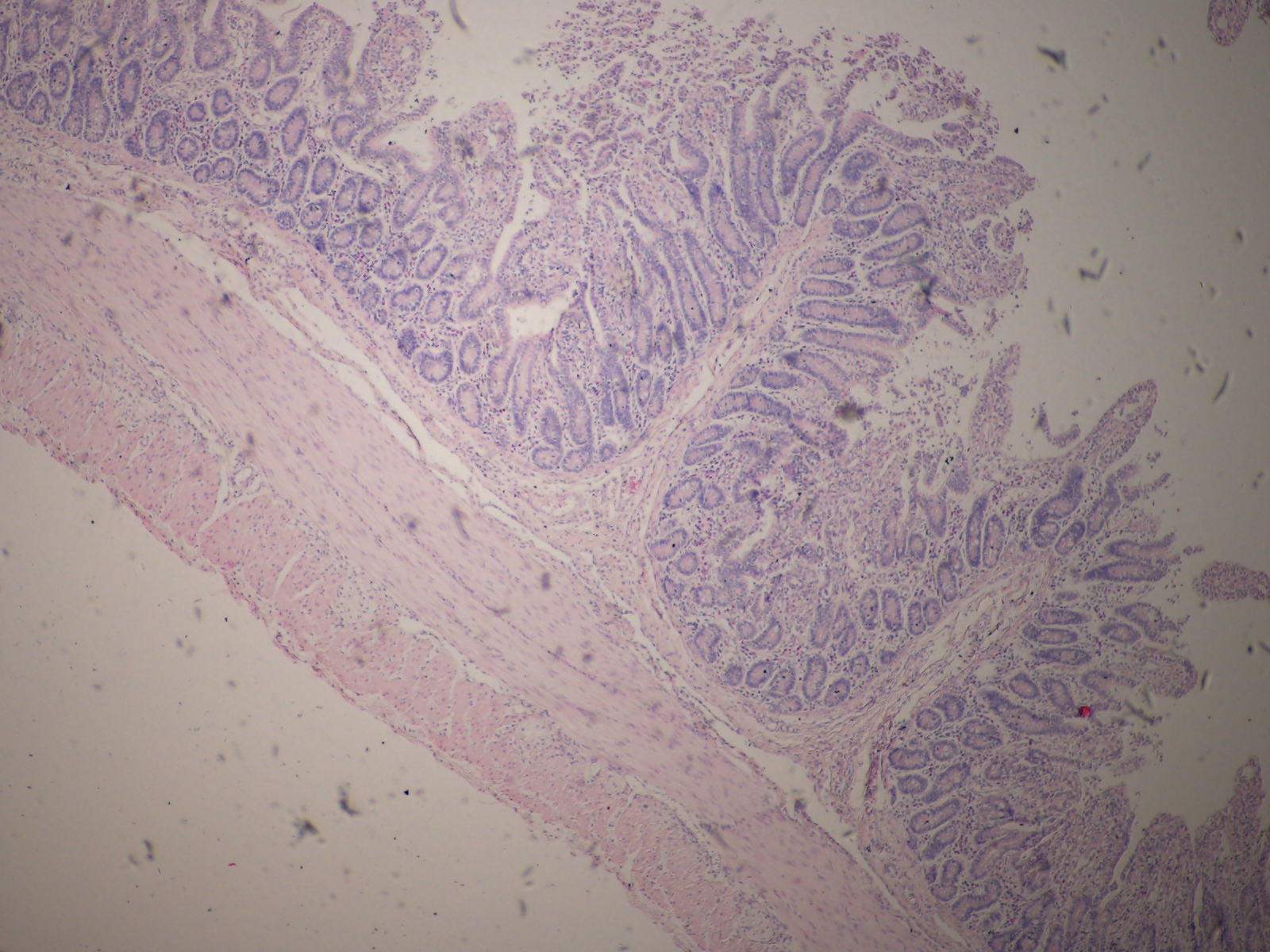

Supplement: Figure 2—source data 1. [file elife-81858-fig2-data1.zip › Figure 2-source data 1/fig2.a/WT/WT (1).tif]

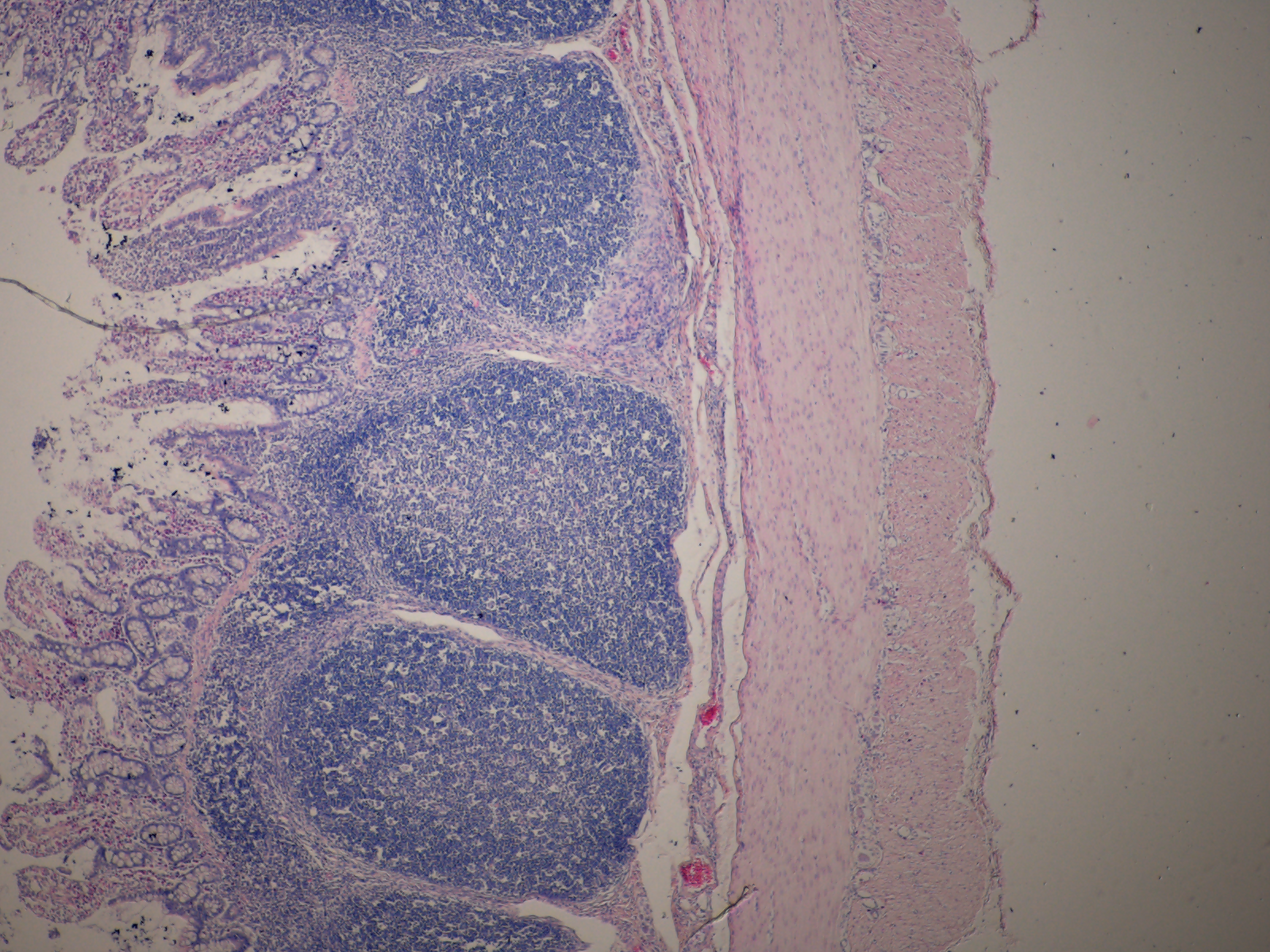

Supplement: Figure 2—source data 1. [file elife-81858-fig2-data1.zip › Figure 2-source data 1/fig2.a/WT/WT (2).tif]

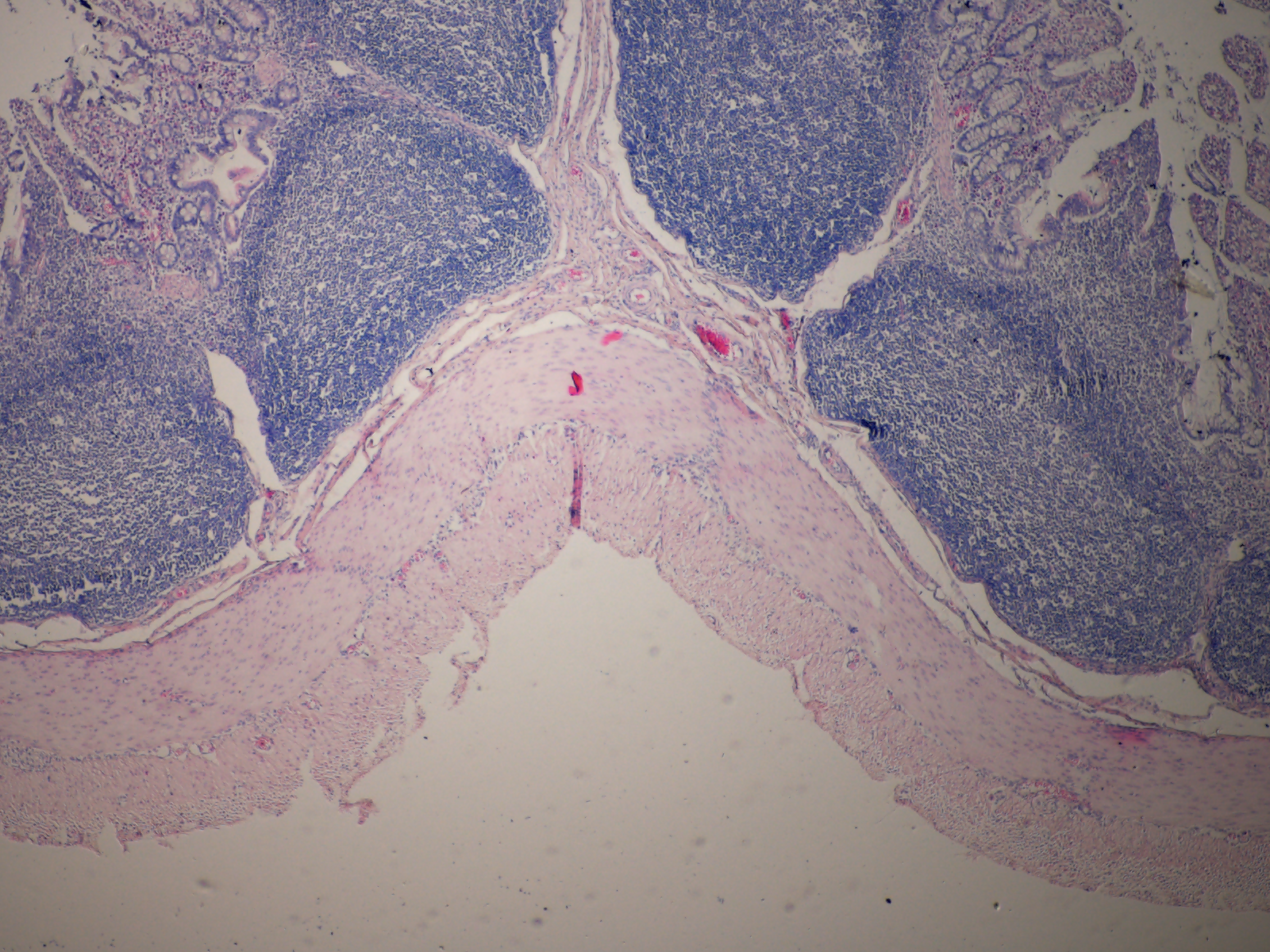

Supplement: Figure 2—source data 1. [file elife-81858-fig2-data1.zip › Figure 2-source data 1/fig2.a/WT/WT (3).tif]

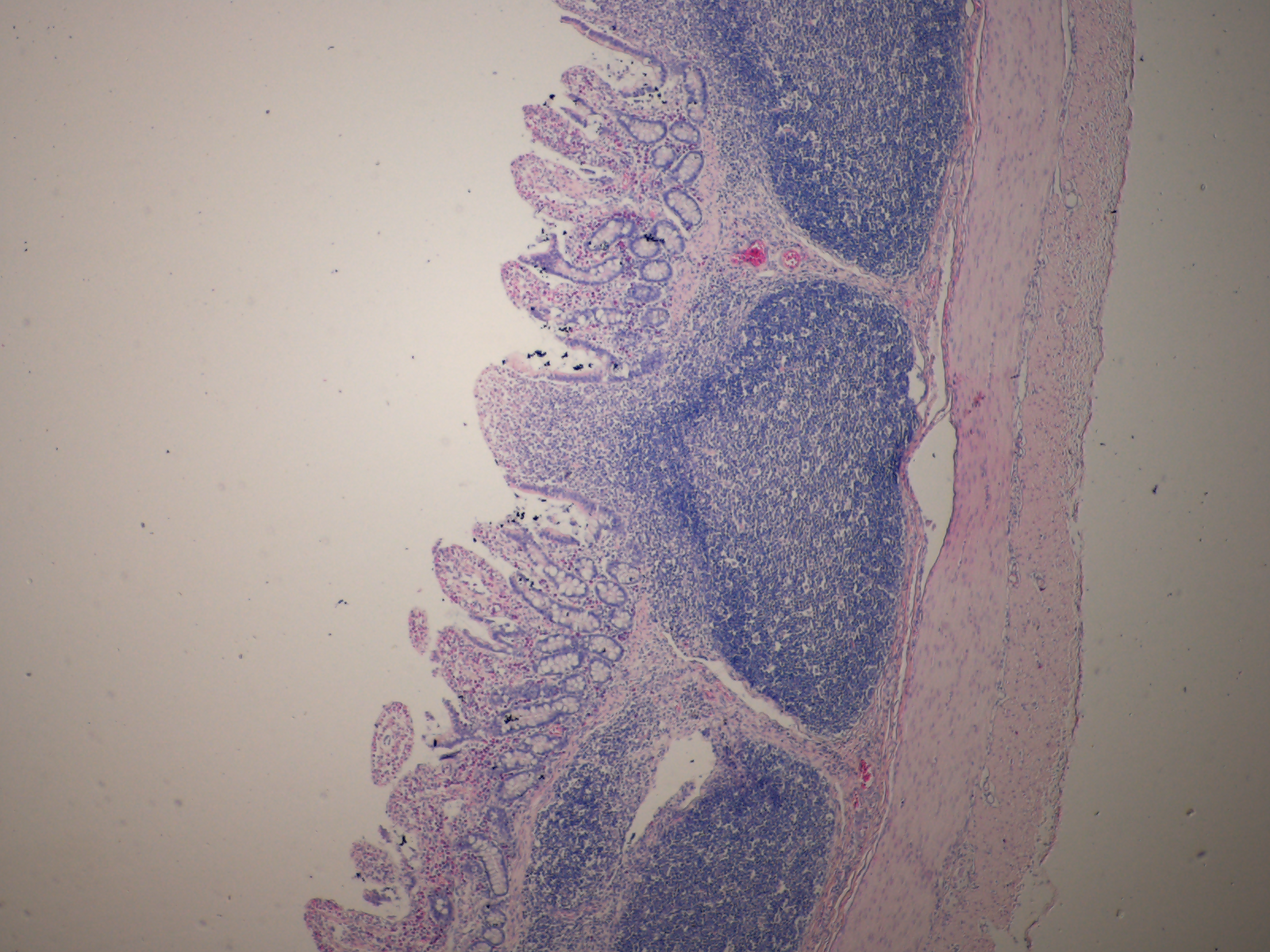

Supplement: Figure 2—source data 1. [file elife-81858-fig2-data1.zip › Figure 2-source data 1/fig2.a/WT/WT (4).tif]

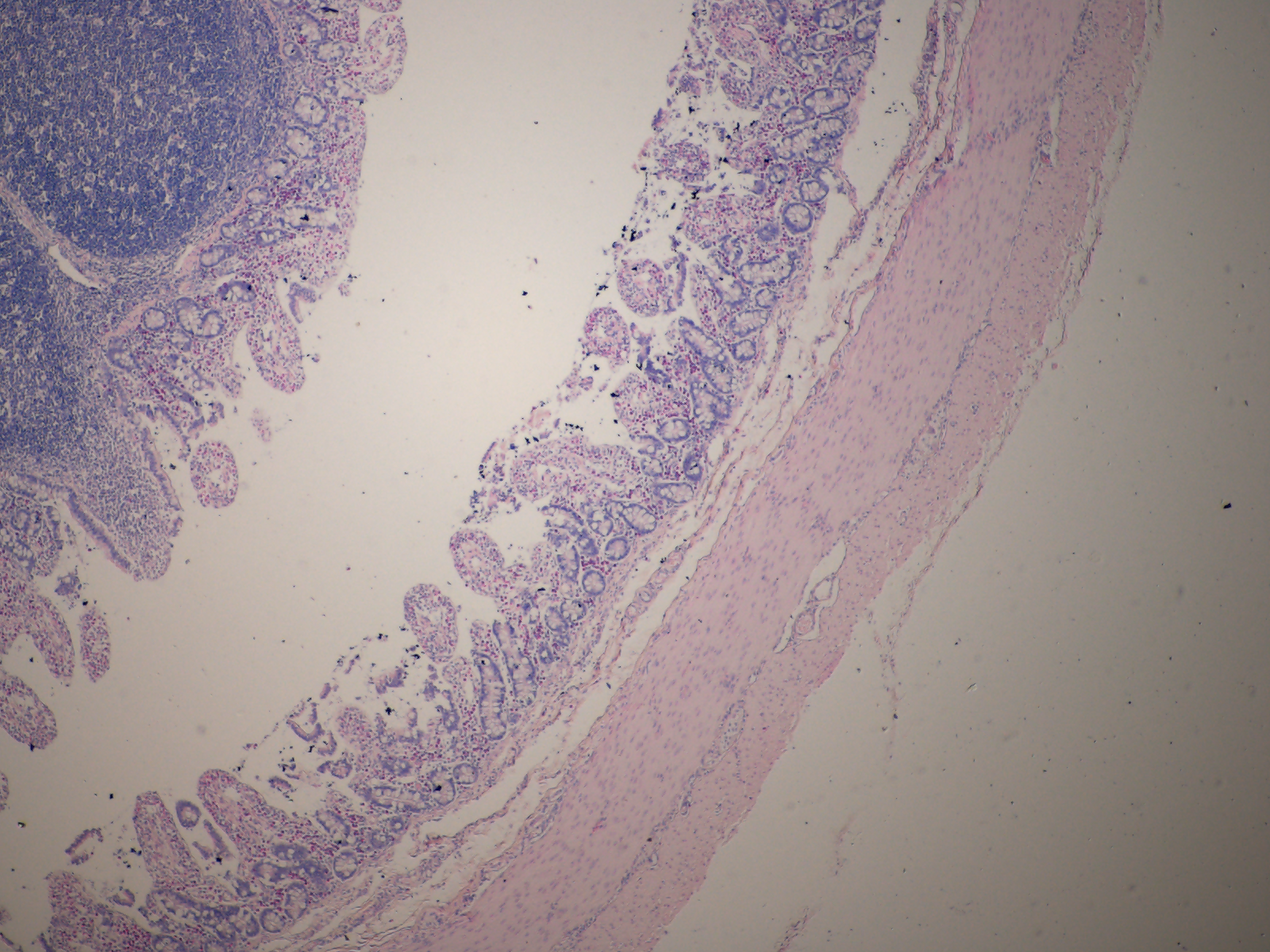

Supplement: Figure 2—source data 1. [file elife-81858-fig2-data1.zip › Figure 2-source data 1/fig2.a/WT/WT (5).tif]

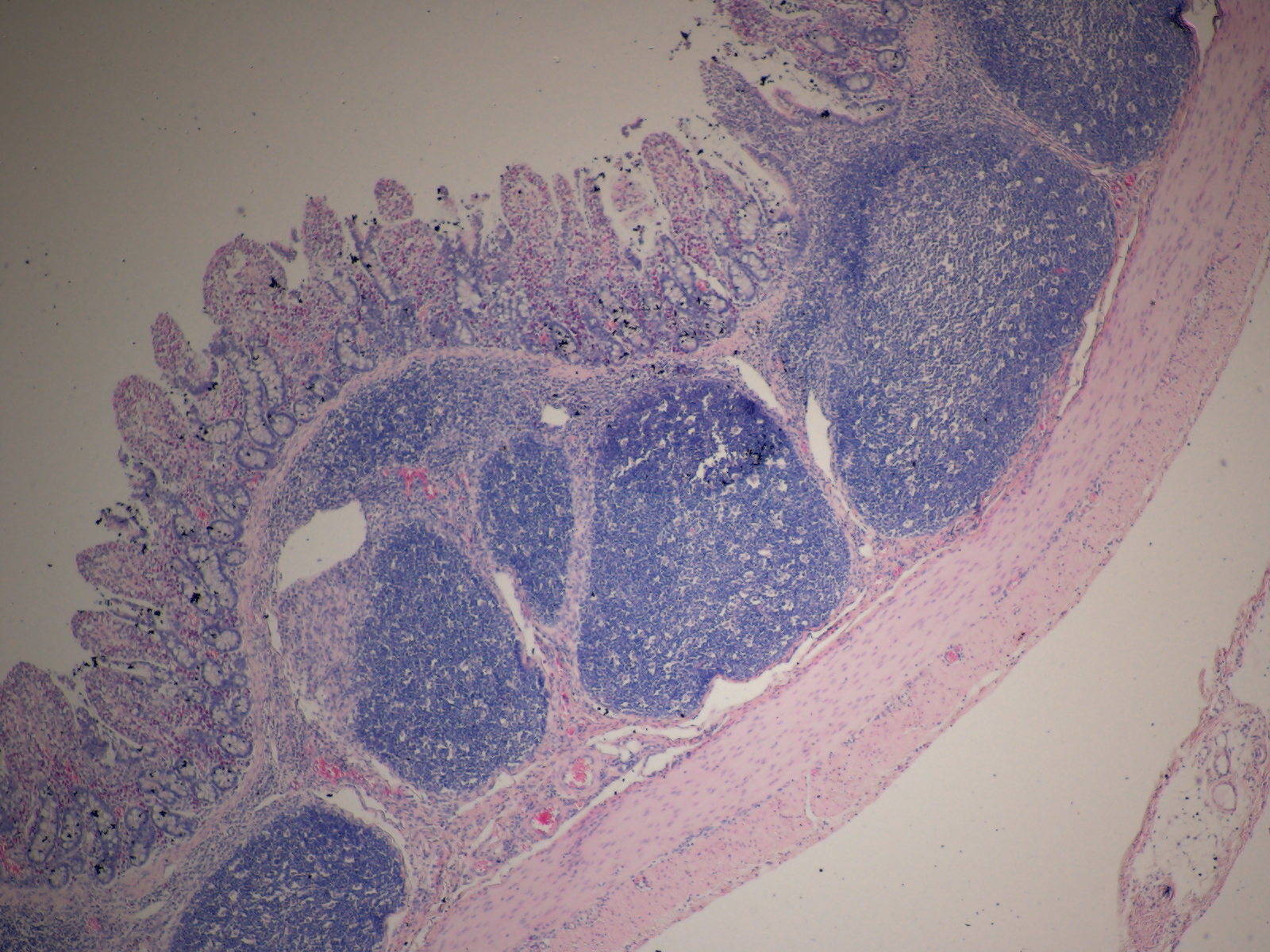

Supplement: Figure 2—source data 1. [file elife-81858-fig2-data1.zip › Figure 2-source data 1/fig2.a/WT/WT (6).tif]

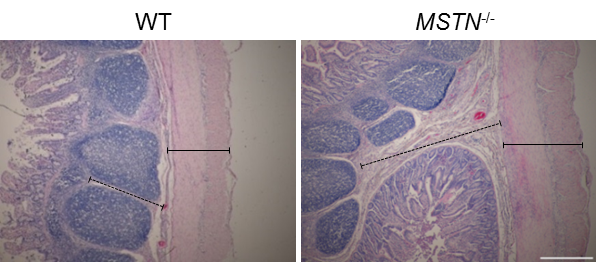

Supplement: Figure 2—source data 1. [file elife-81858-fig2-data1.zip › Figure 2-source data 1/fig2.a/fig.2a.tif]

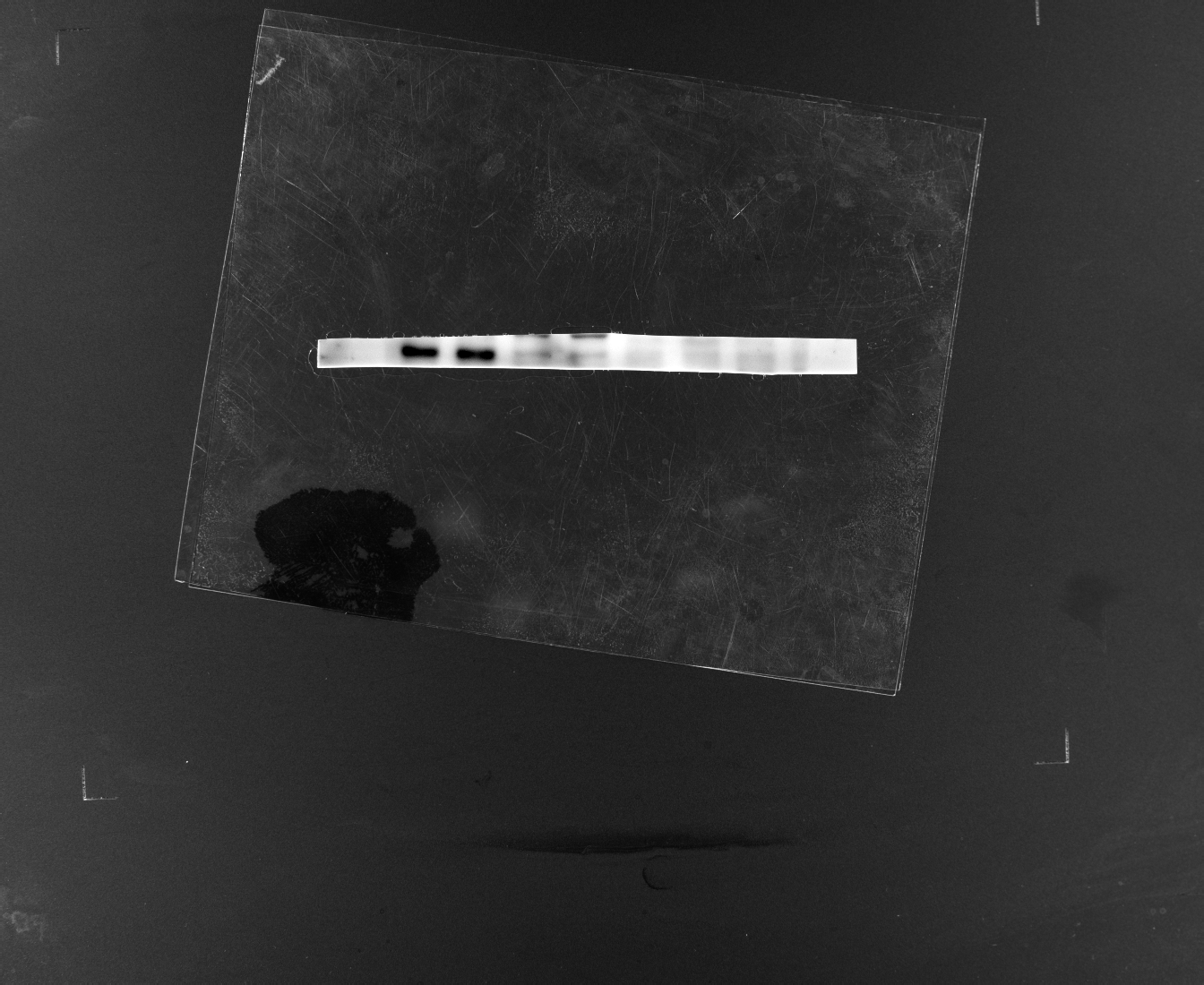

Supplement: Figure 2—source data 2. [file elife-81858-fig2-data2.zip › Figure 2-source data 2/fig2b.MSTN.tif]

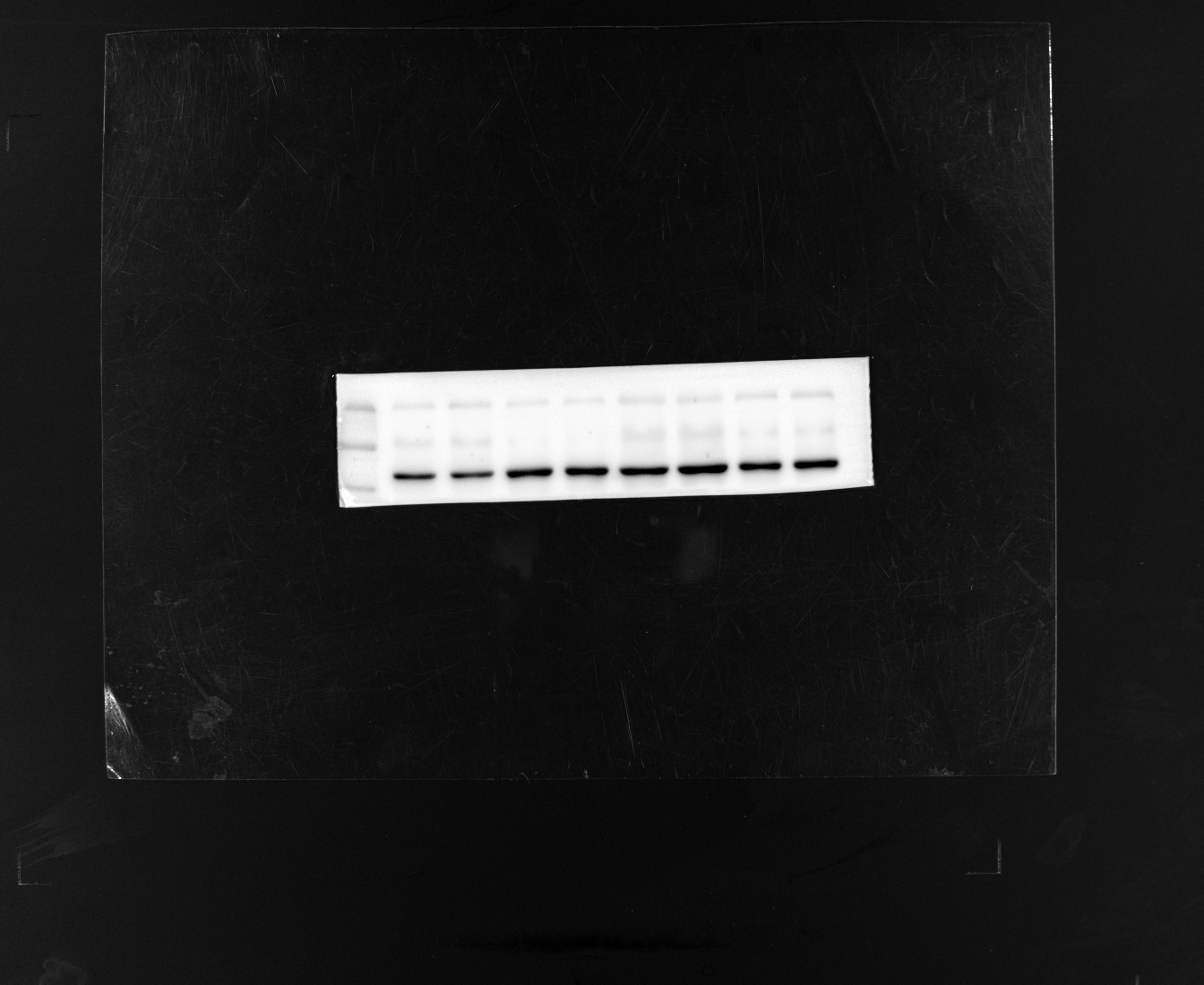

Supplement: Figure 2—source data 2. [file elife-81858-fig2-data2.zip › Figure 2-source data 2/fig2b.aSMA.tif]

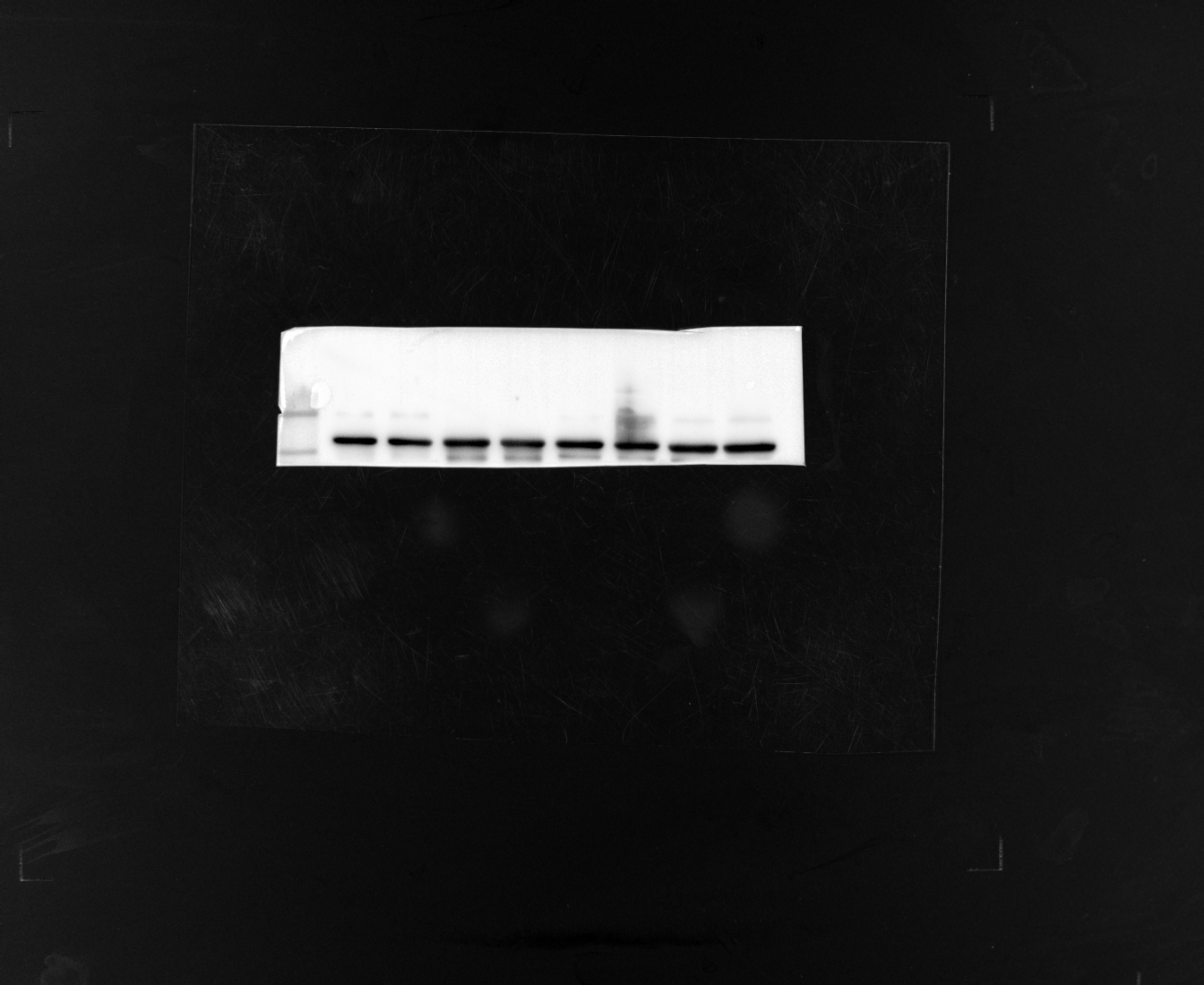

Supplement: Figure 2—source data 2. [file elife-81858-fig2-data2.zip › Figure 2-source data 2/fig2b.actin.tif]

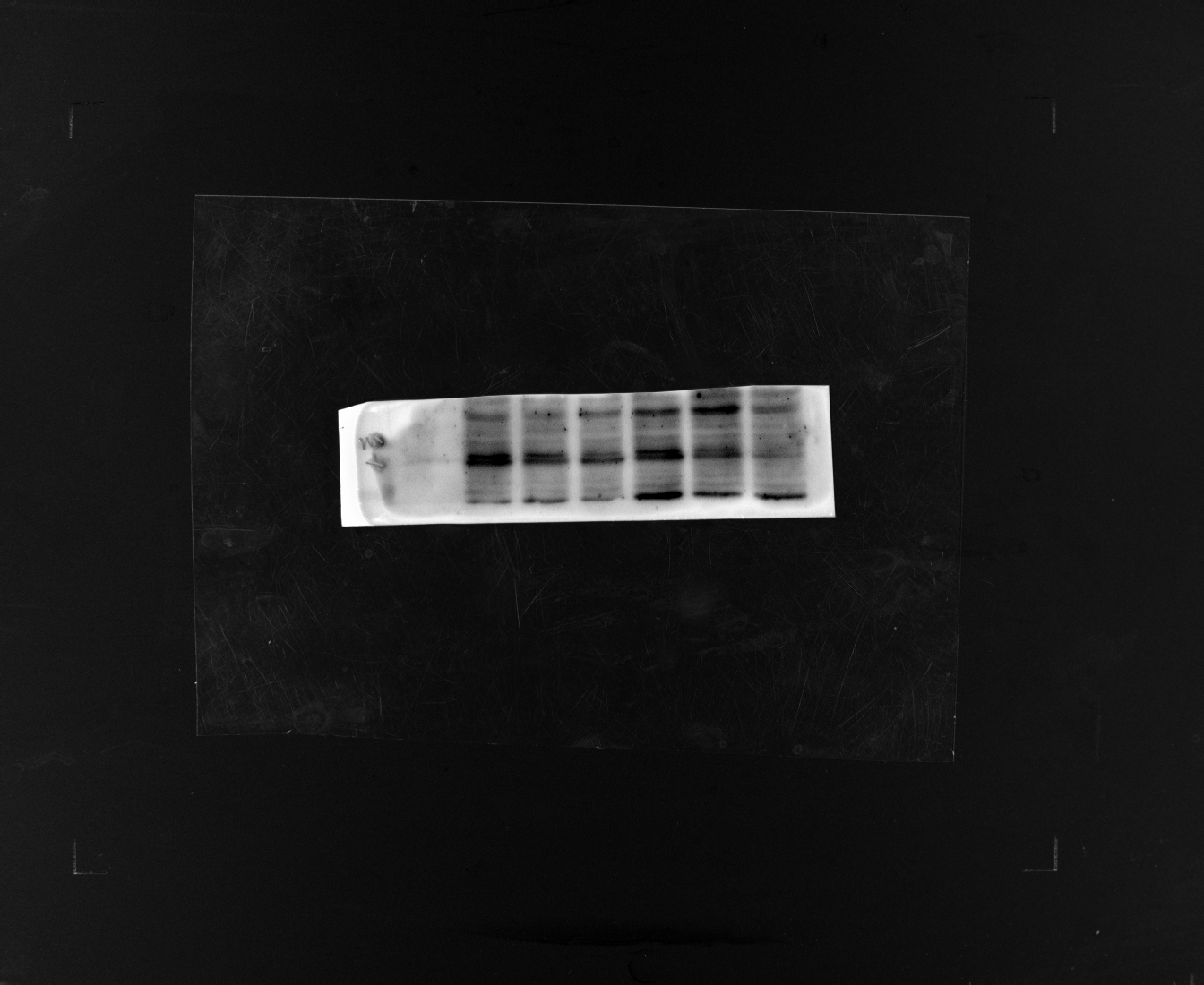

Supplement: Figure 2—source data 2. [file elife-81858-fig2-data2.zip › Figure 2-source data 2/fig2b.calponin-1.tif]

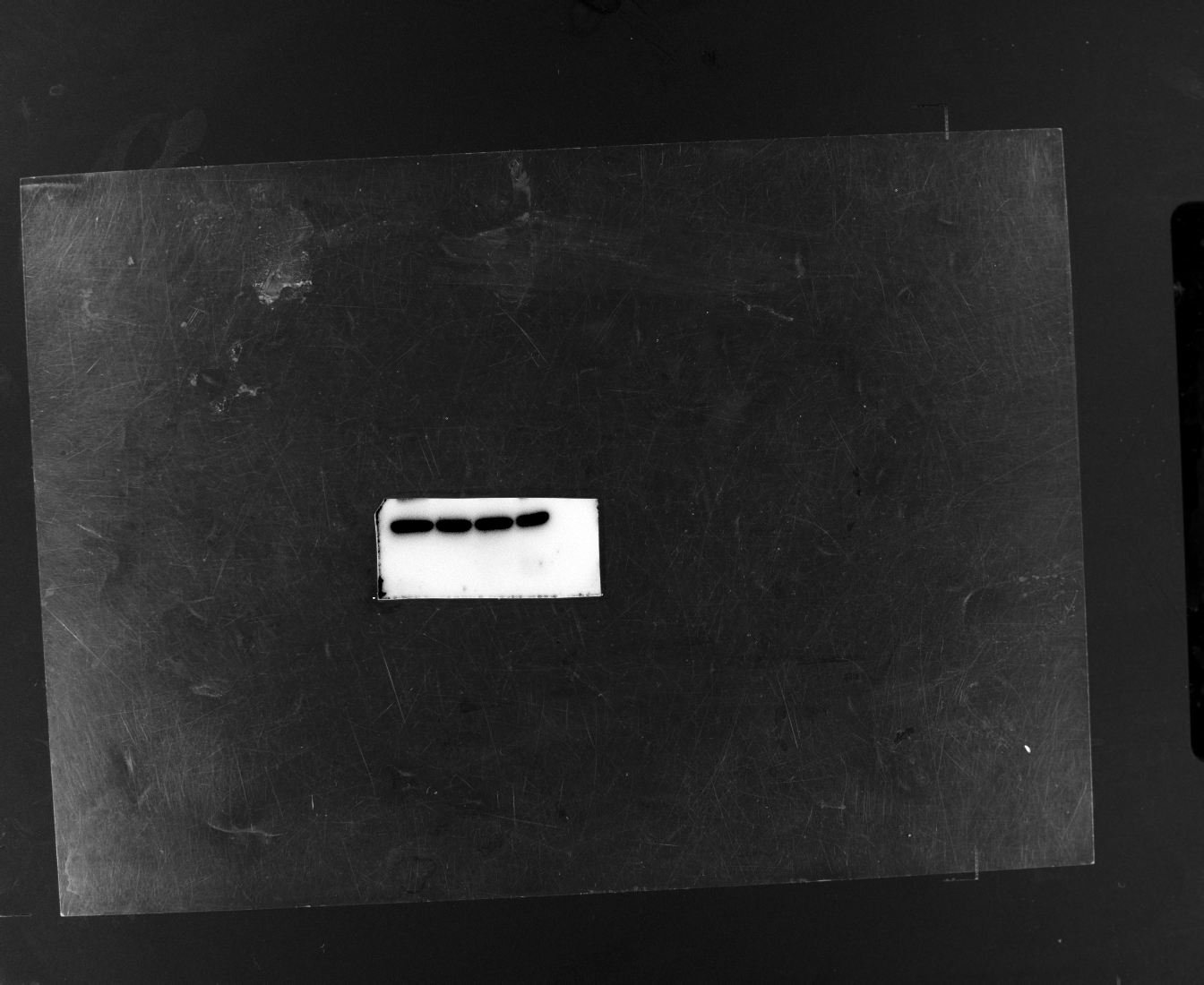

Supplement: Figure 2—source data 2. [file elife-81858-fig2-data2.zip › Figure 2-source data 2/fig2d.MLC.tif]

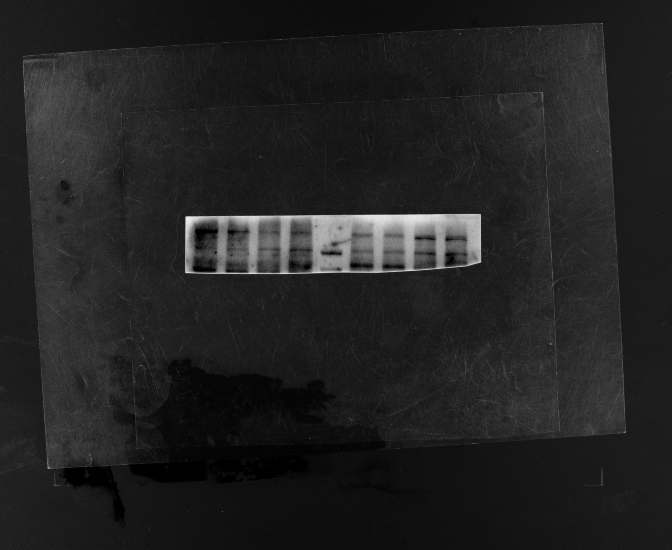

Supplement: Figure 2—source data 2. [file elife-81858-fig2-data2.zip › Figure 2-source data 2/fig2d.MLCK.tif]

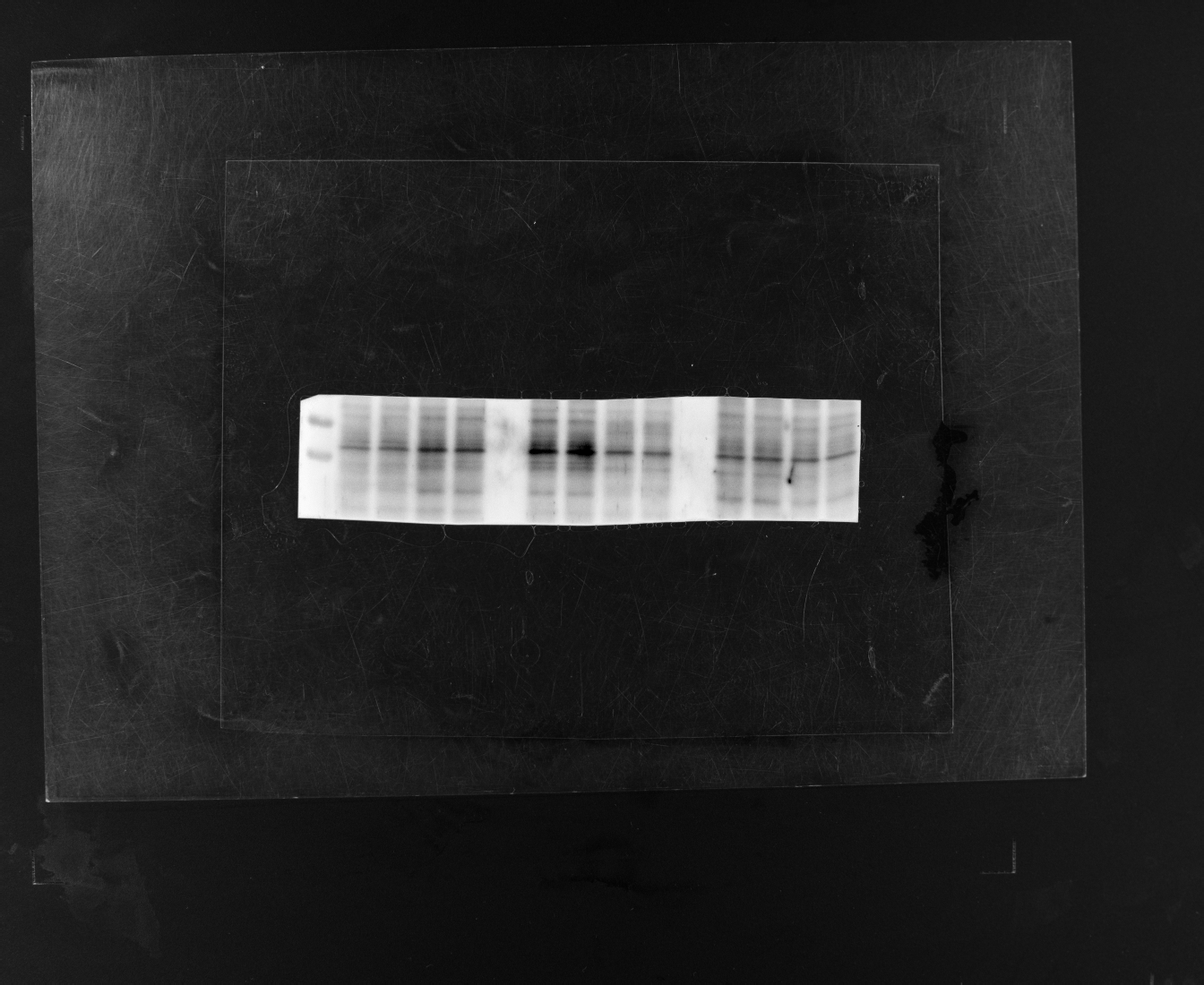

Supplement: Figure 2—source data 2. [file elife-81858-fig2-data2.zip › Figure 2-source data 2/fig2d.pMLC.tif]

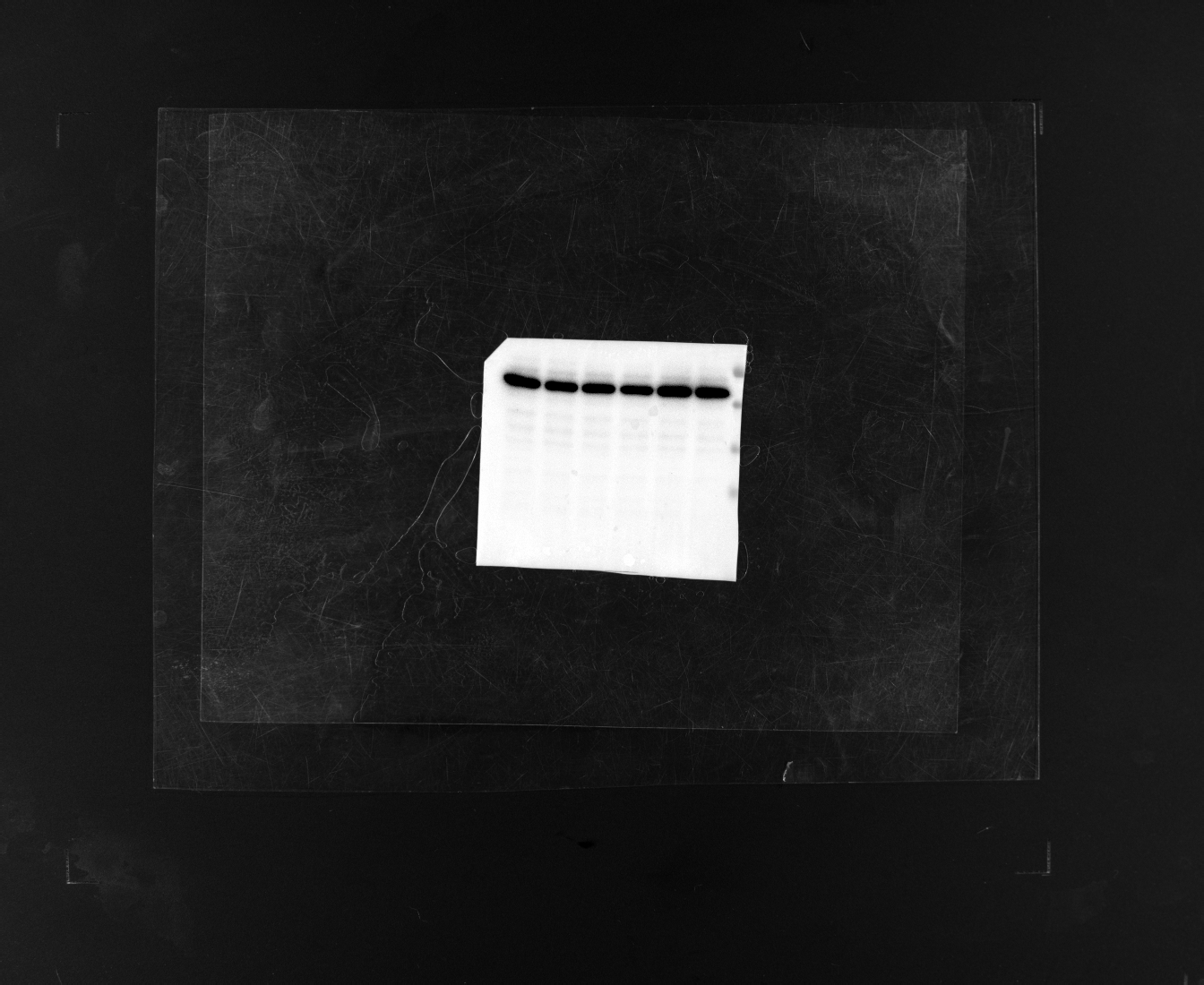

Supplement: Figure 2—source data 2. [file elife-81858-fig2-data2.zip › Figure 2-source data 2/fig2e.MLC.tif]

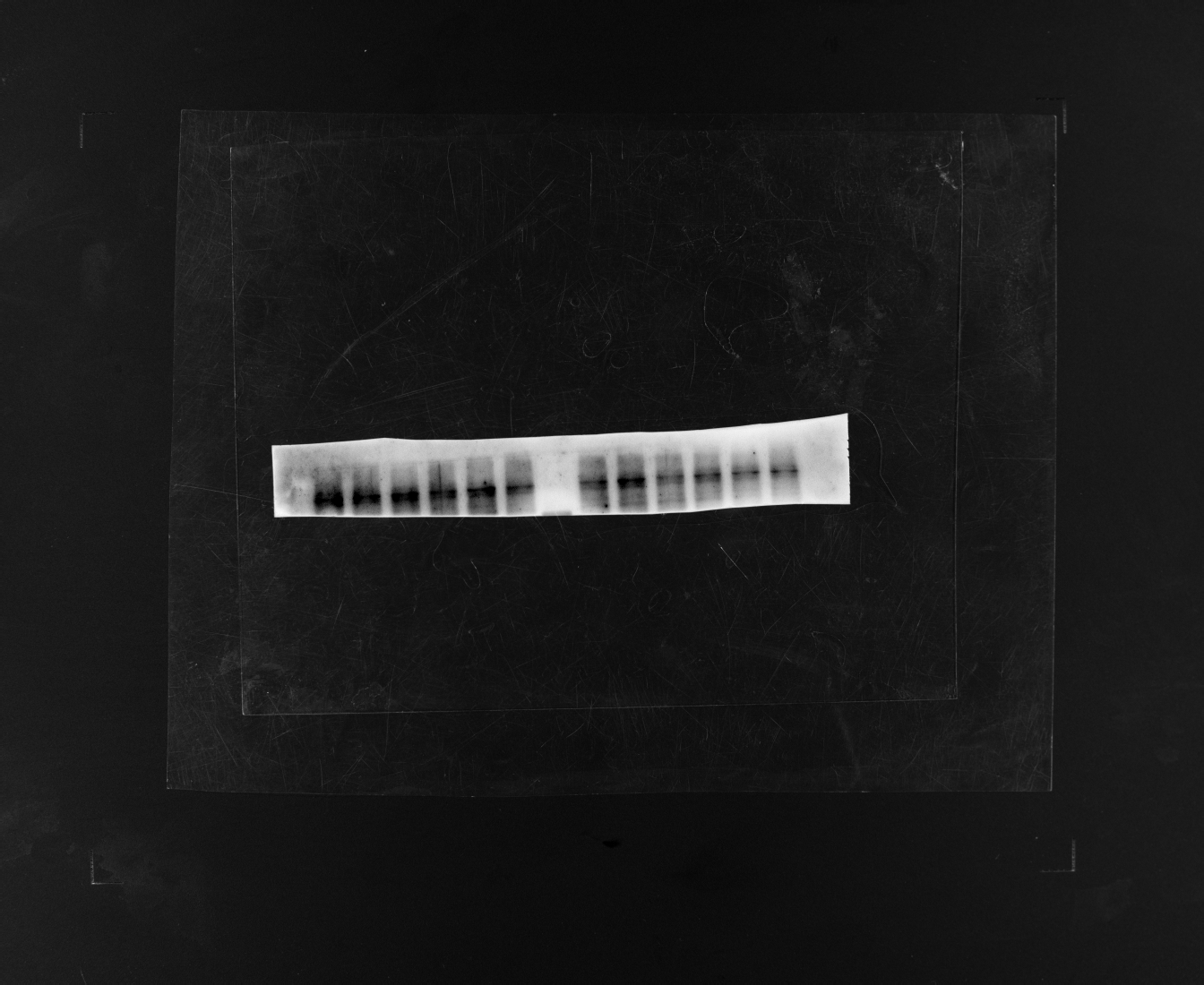

Supplement: Figure 2—source data 2. [file elife-81858-fig2-data2.zip › Figure 2-source data 2/fig2e.MLCK.tif]

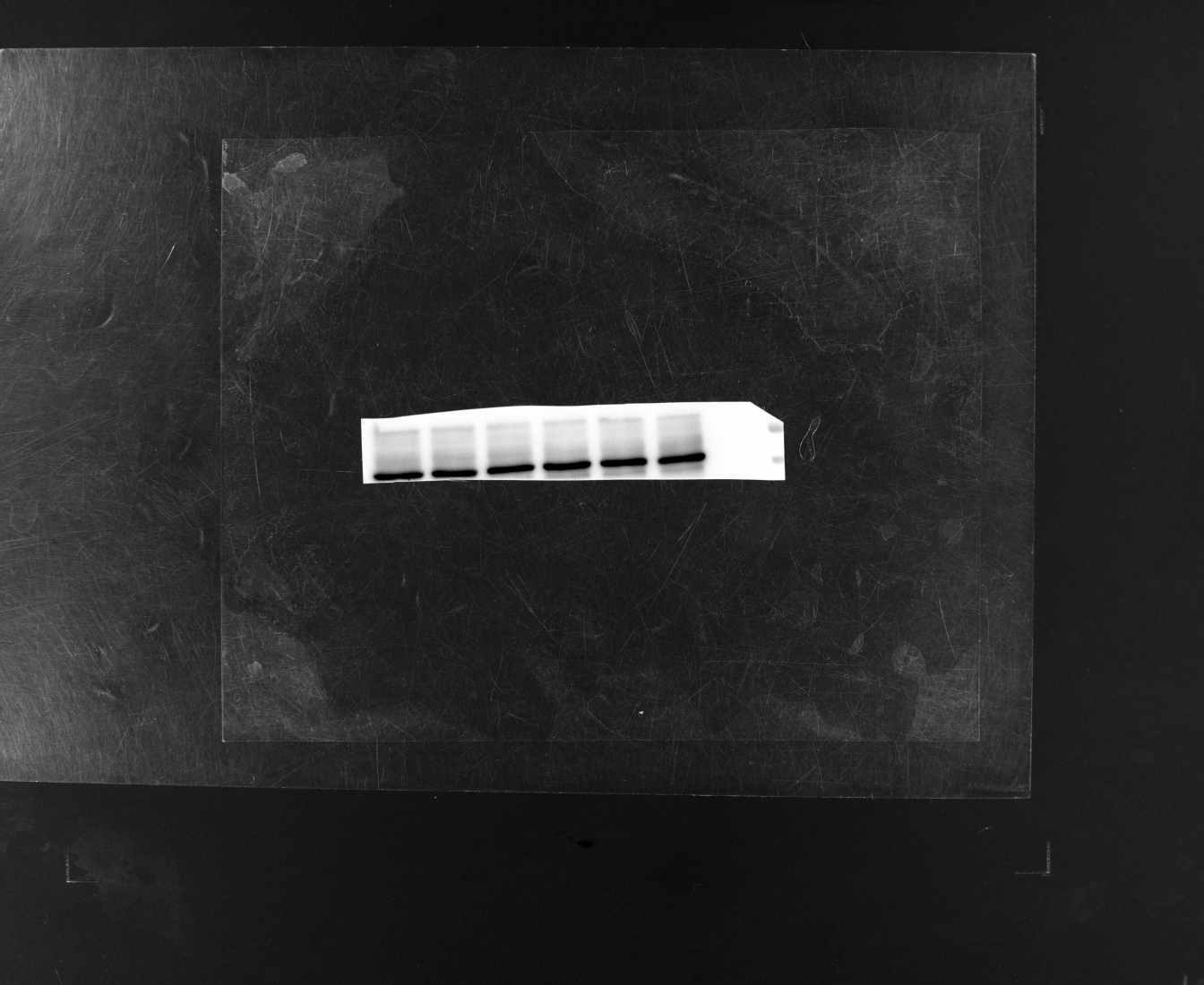

Supplement: Figure 2—source data 2. [file elife-81858-fig2-data2.zip › Figure 2-source data 2/fig2e.actin.tif]

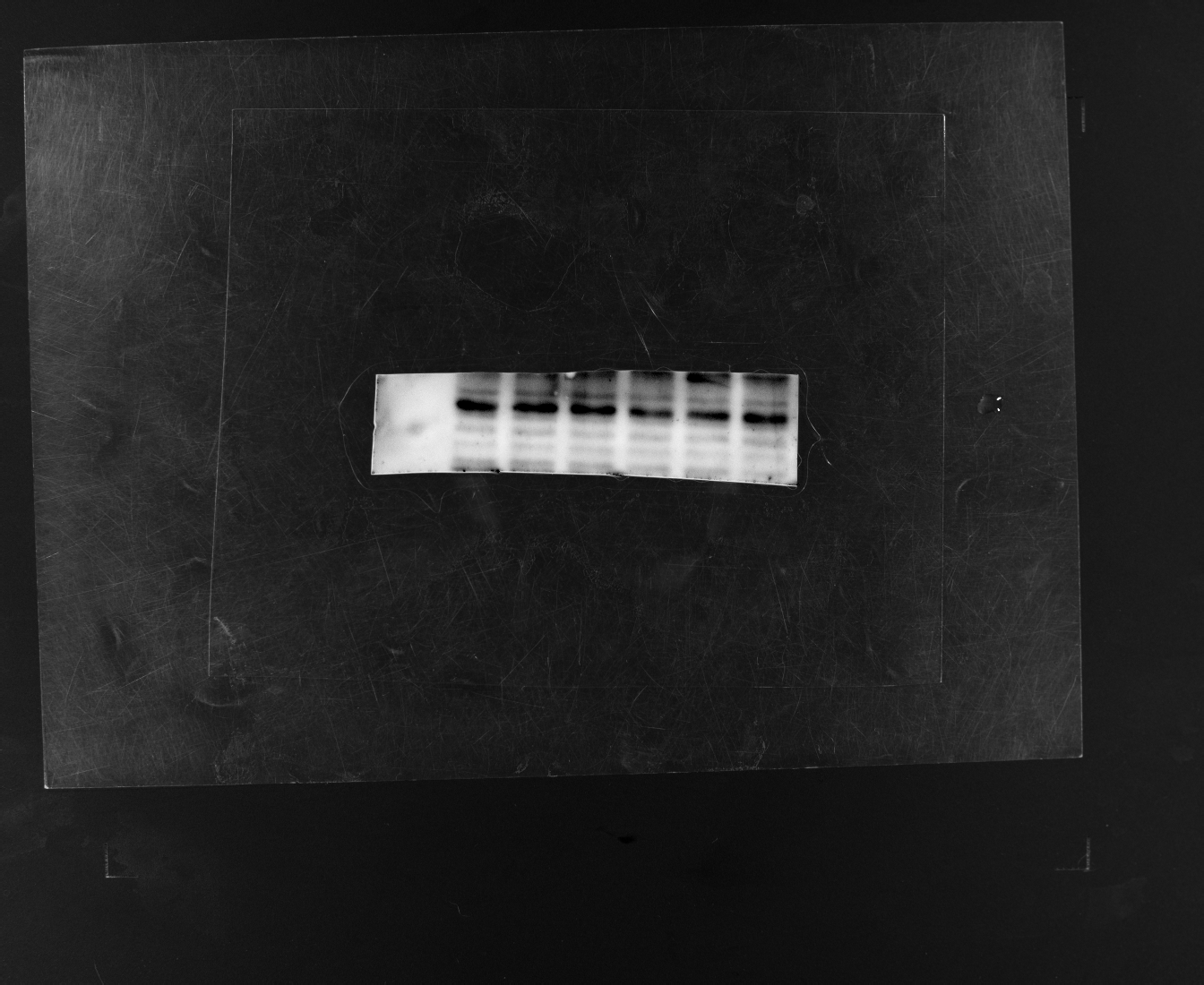

Supplement: Figure 2—source data 2. [file elife-81858-fig2-data2.zip › Figure 2-source data 2/fig2e.pMLC.tif]

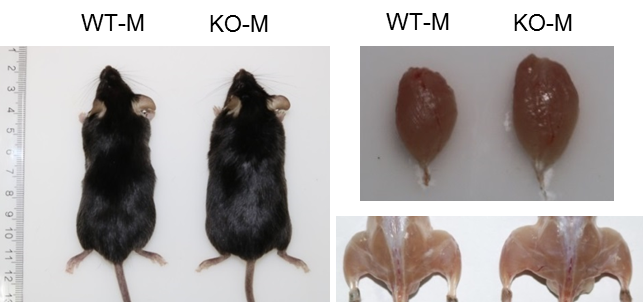

Supplement: Figure 3—source data 1. [file elife-81858-fig3-data1.zip › Figure 3-source data 1/fig3.a/fig3.a.tif]

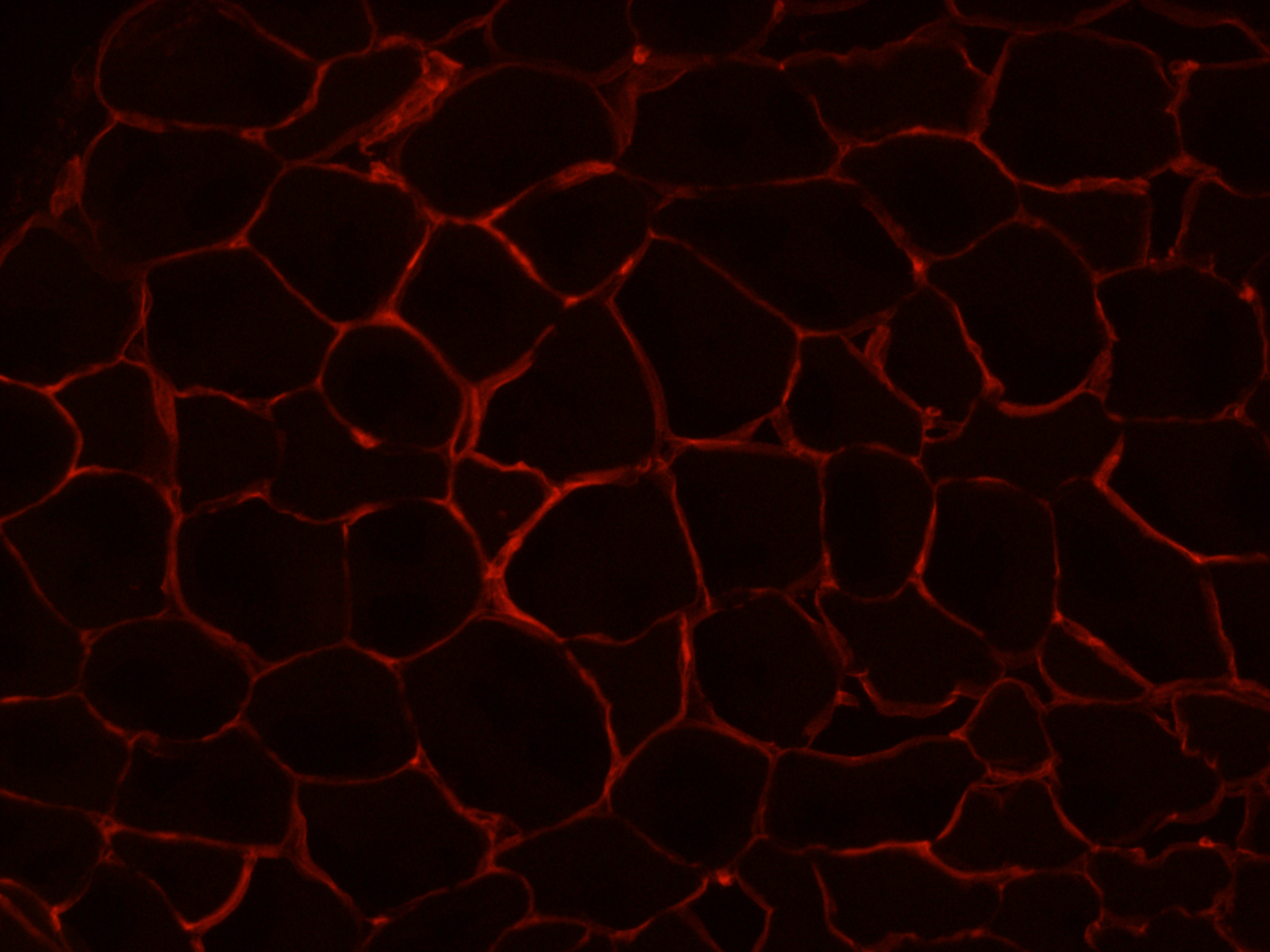

Supplement: Figure 3—source data 1. [file elife-81858-fig3-data1.zip › Figure 3-source data 1/fig3.c/KO-M/_57990.tif]

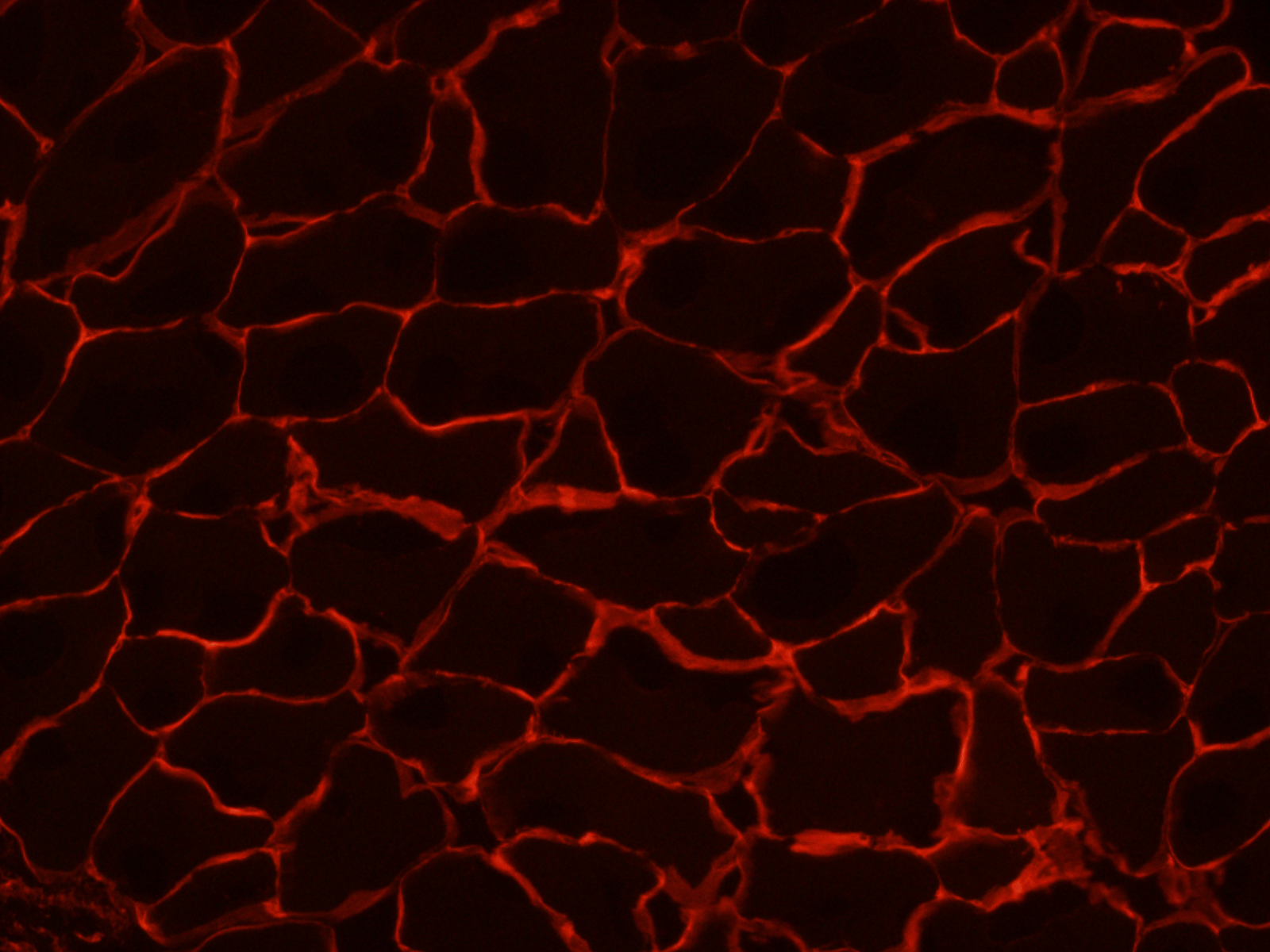

Supplement: Figure 3—source data 1. [file elife-81858-fig3-data1.zip › Figure 3-source data 1/fig3.c/KO-M/_58007.tif]

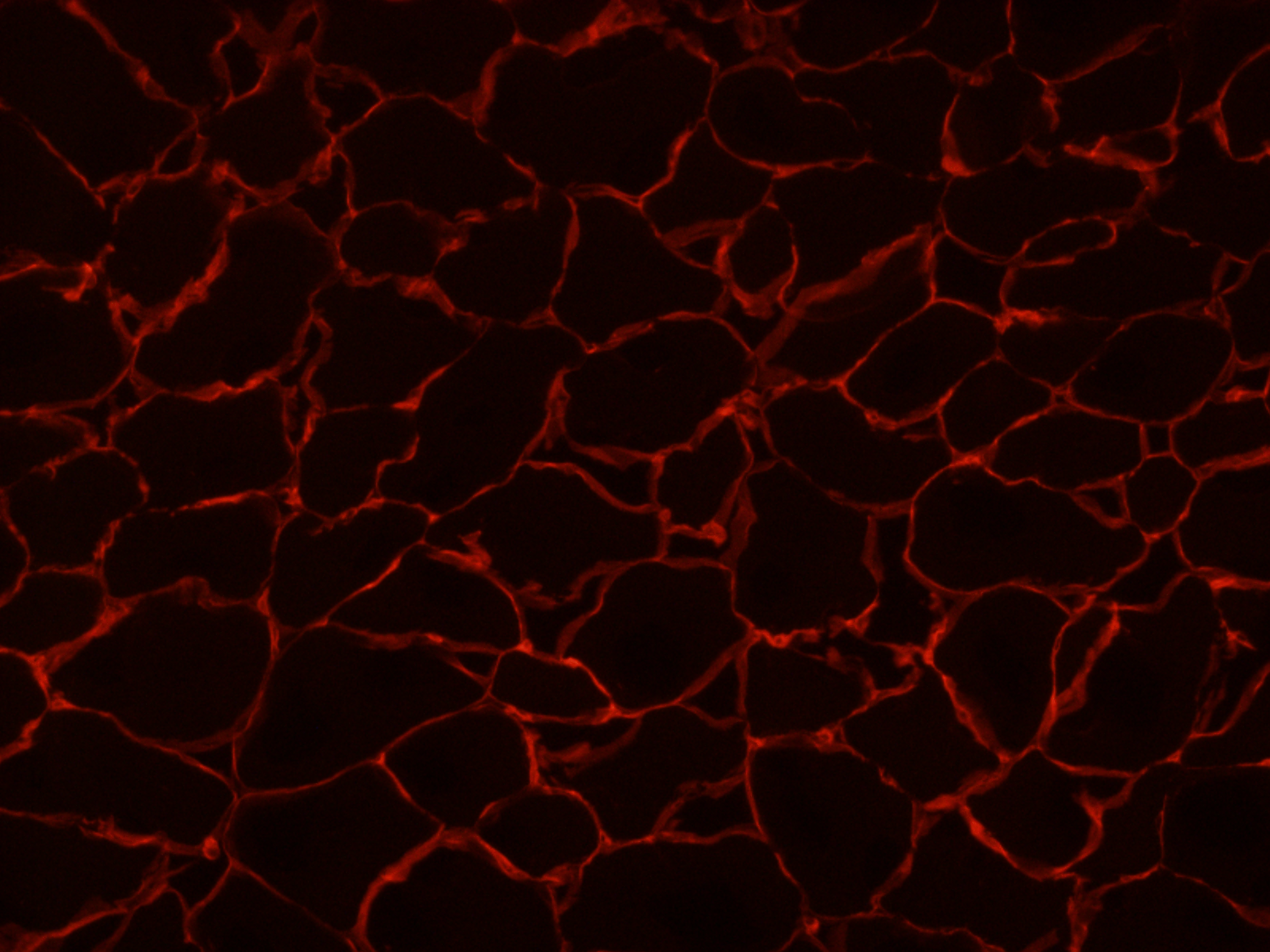

Supplement: Figure 3—source data 1. [file elife-81858-fig3-data1.zip › Figure 3-source data 1/fig3.c/KO-M/_58010.tif]

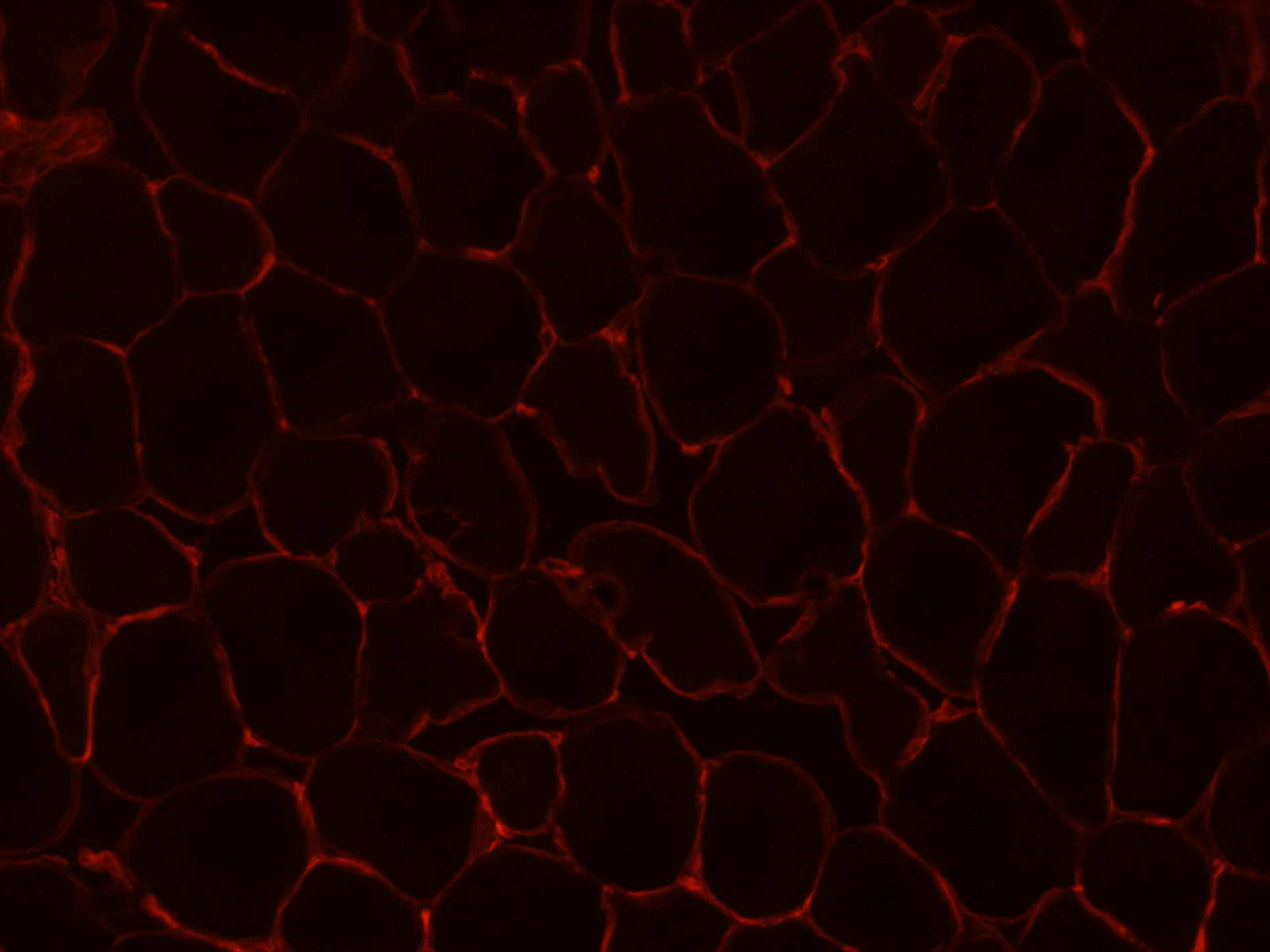

Supplement: Figure 3—source data 1. [file elife-81858-fig3-data1.zip › Figure 3-source data 1/fig3.c/KO-M/_58023.tif]

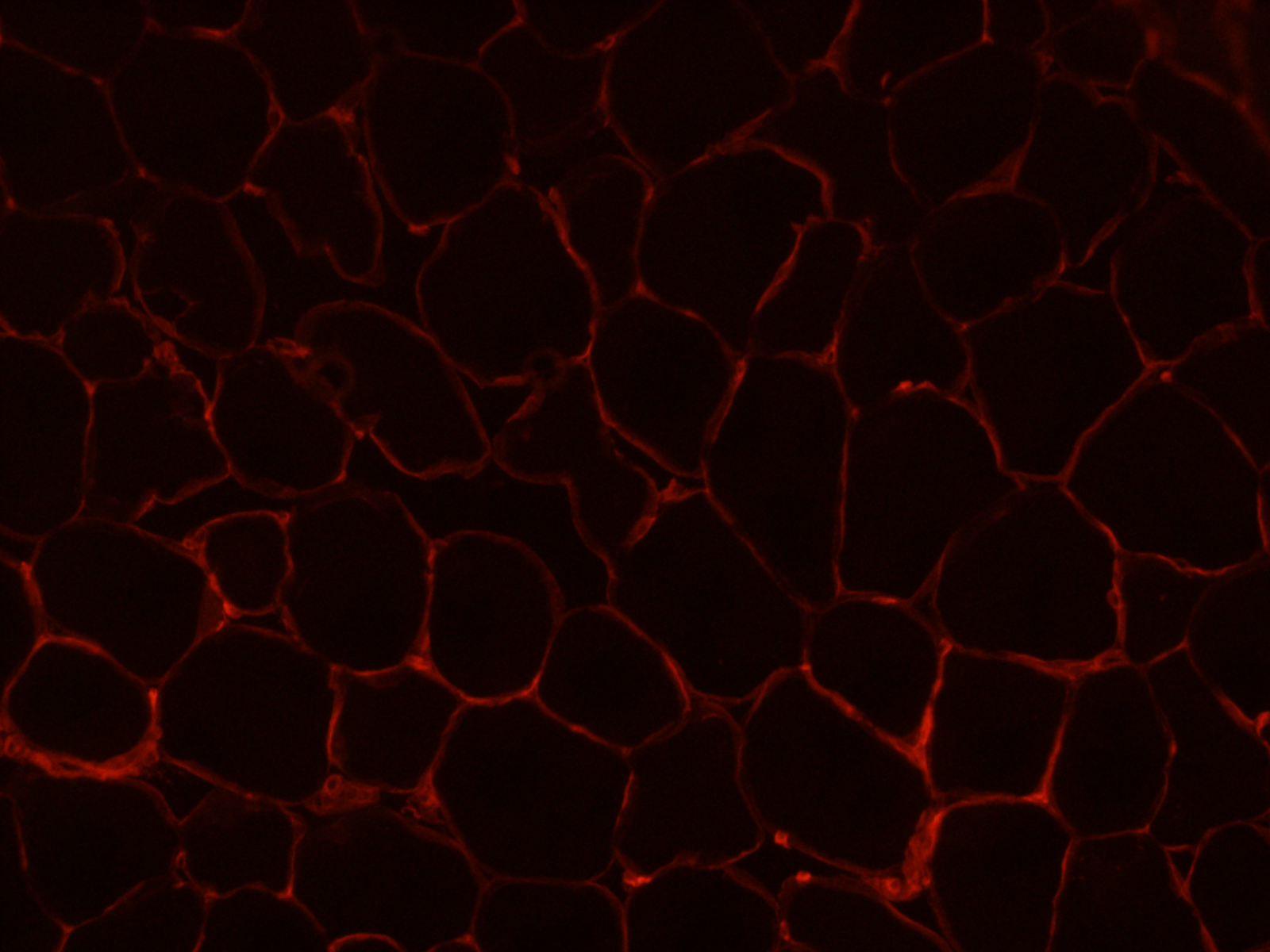

Supplement: Figure 3—source data 1. [file elife-81858-fig3-data1.zip › Figure 3-source data 1/fig3.c/KO-M/_58025.tif]

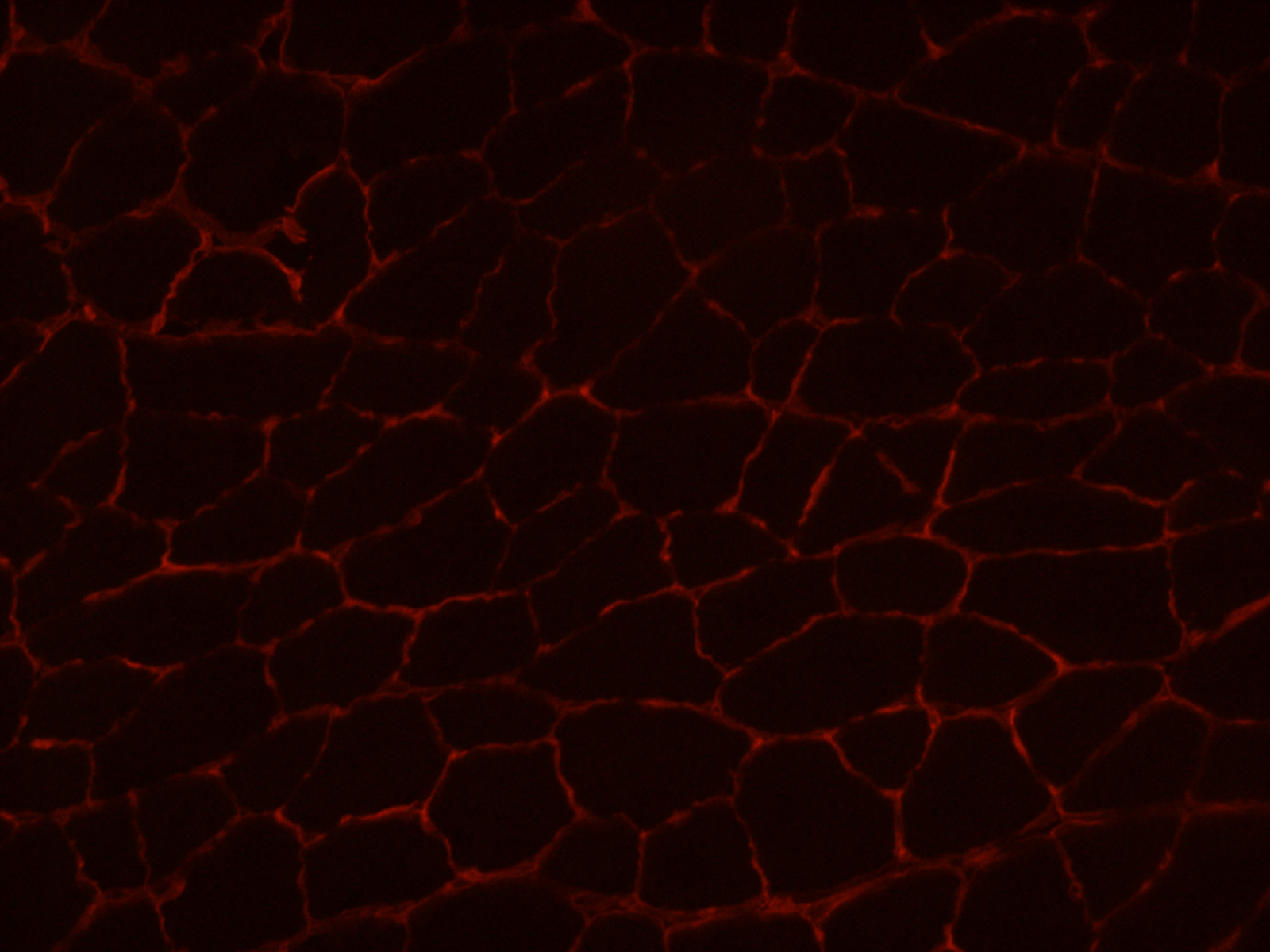

Supplement: Figure 3—source data 1. [file elife-81858-fig3-data1.zip › Figure 3-source data 1/fig3.c/WT-M/_58039.tif]

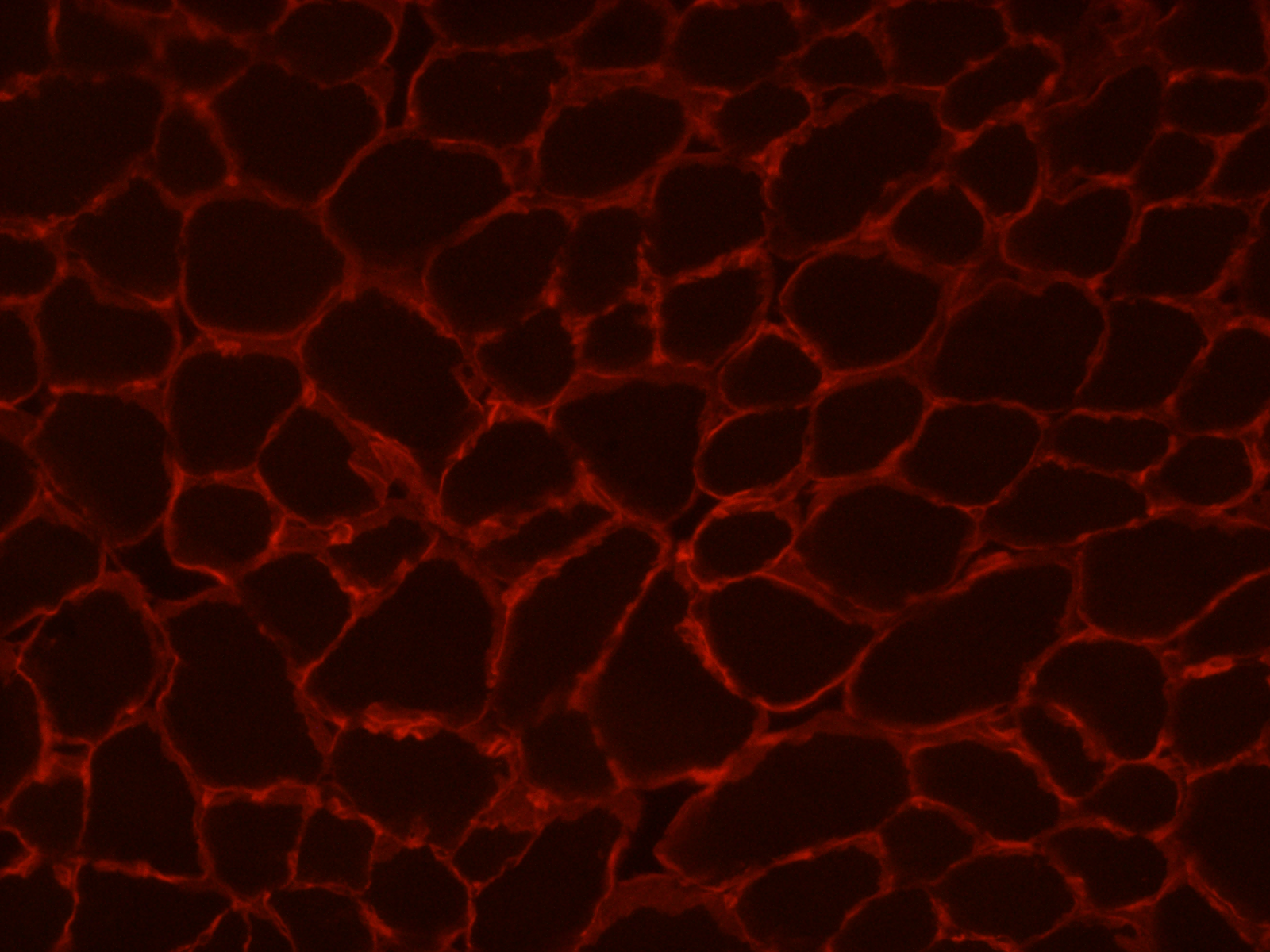

Supplement: Figure 3—source data 1. [file elife-81858-fig3-data1.zip › Figure 3-source data 1/fig3.c/WT-M/_58050.tif]

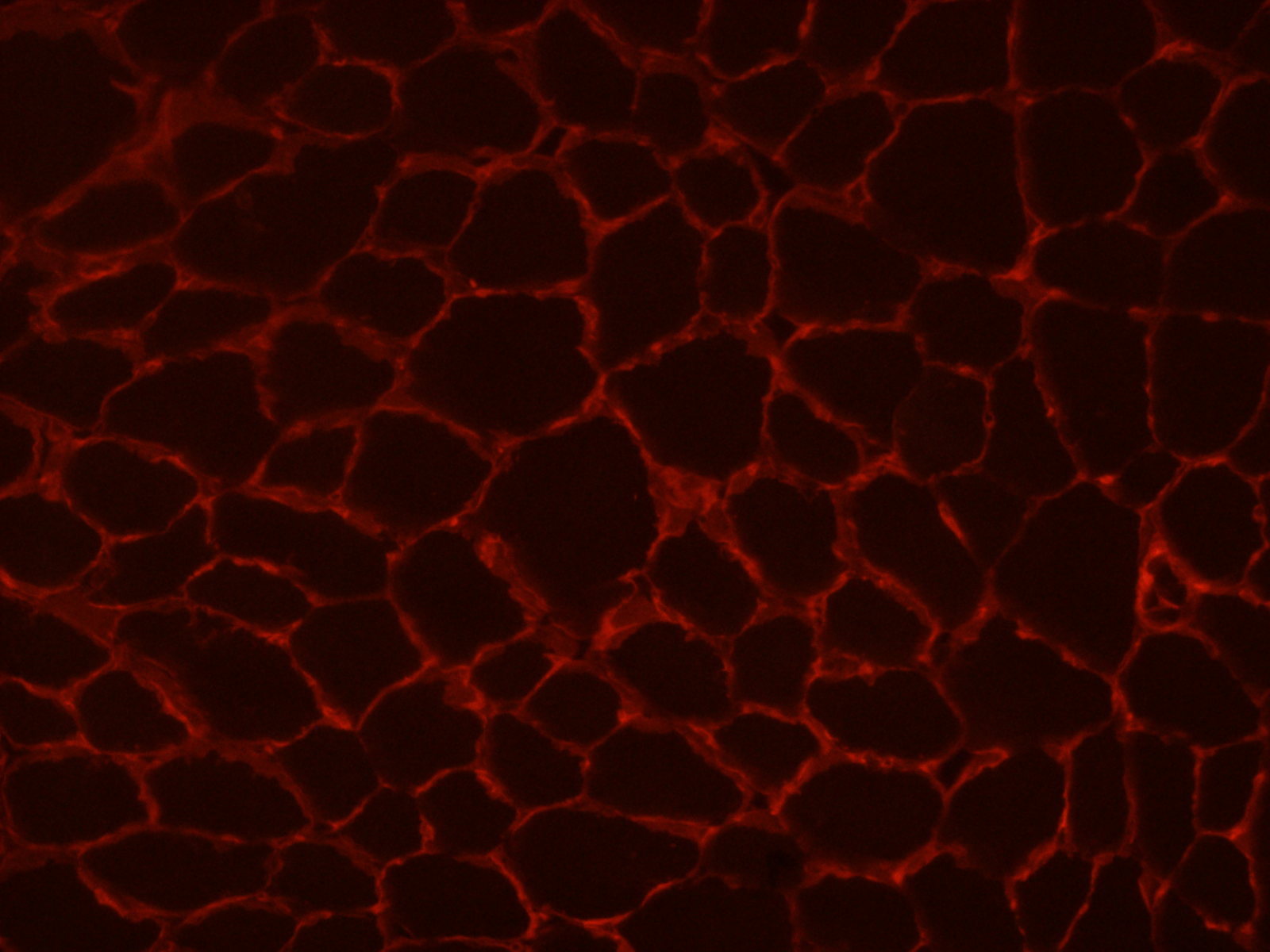

Supplement: Figure 3—source data 1. [file elife-81858-fig3-data1.zip › Figure 3-source data 1/fig3.c/WT-M/_58054.tif]

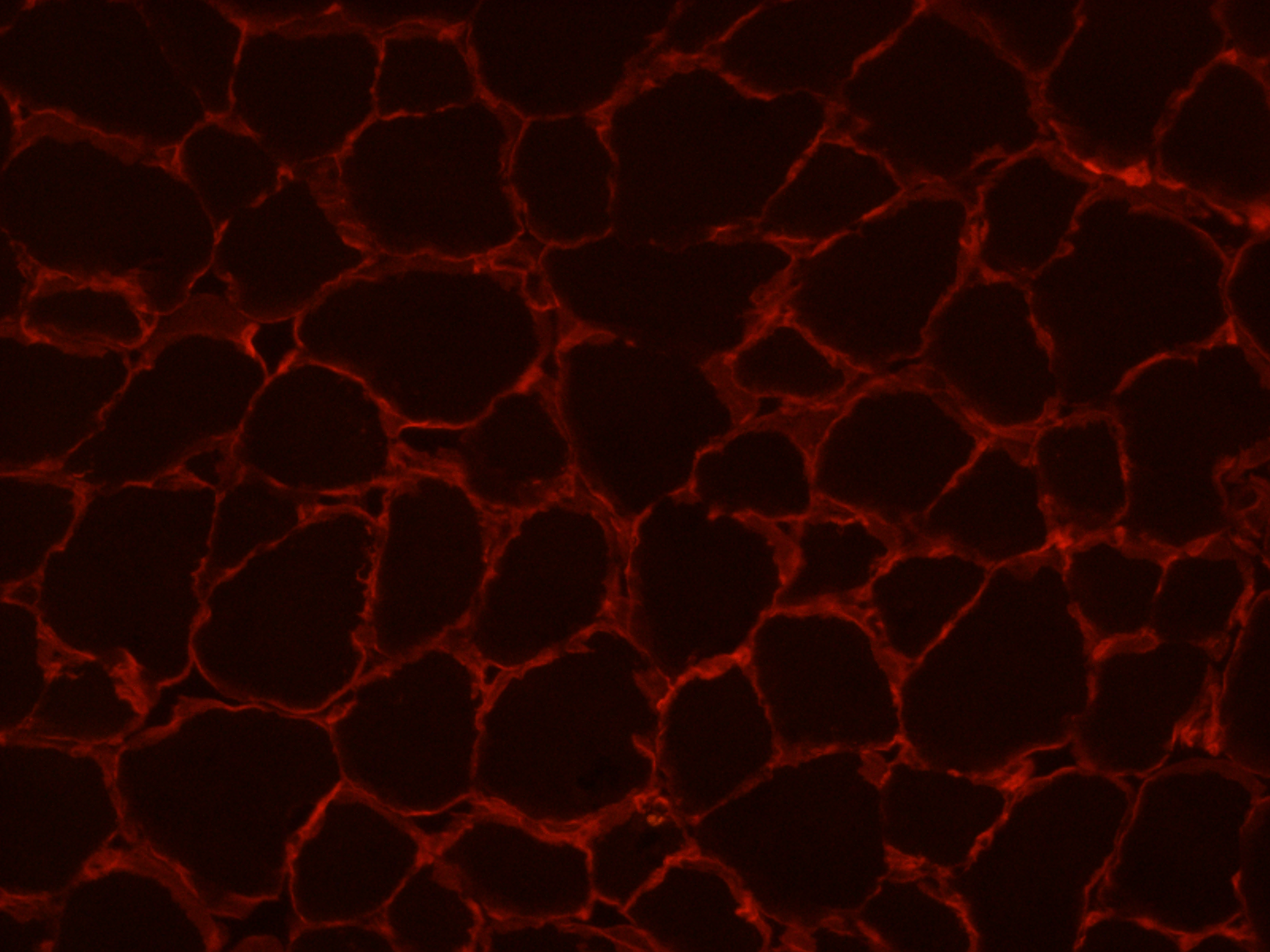

Supplement: Figure 3—source data 1. [file elife-81858-fig3-data1.zip › Figure 3-source data 1/fig3.c/WT-M/_58059.tif]

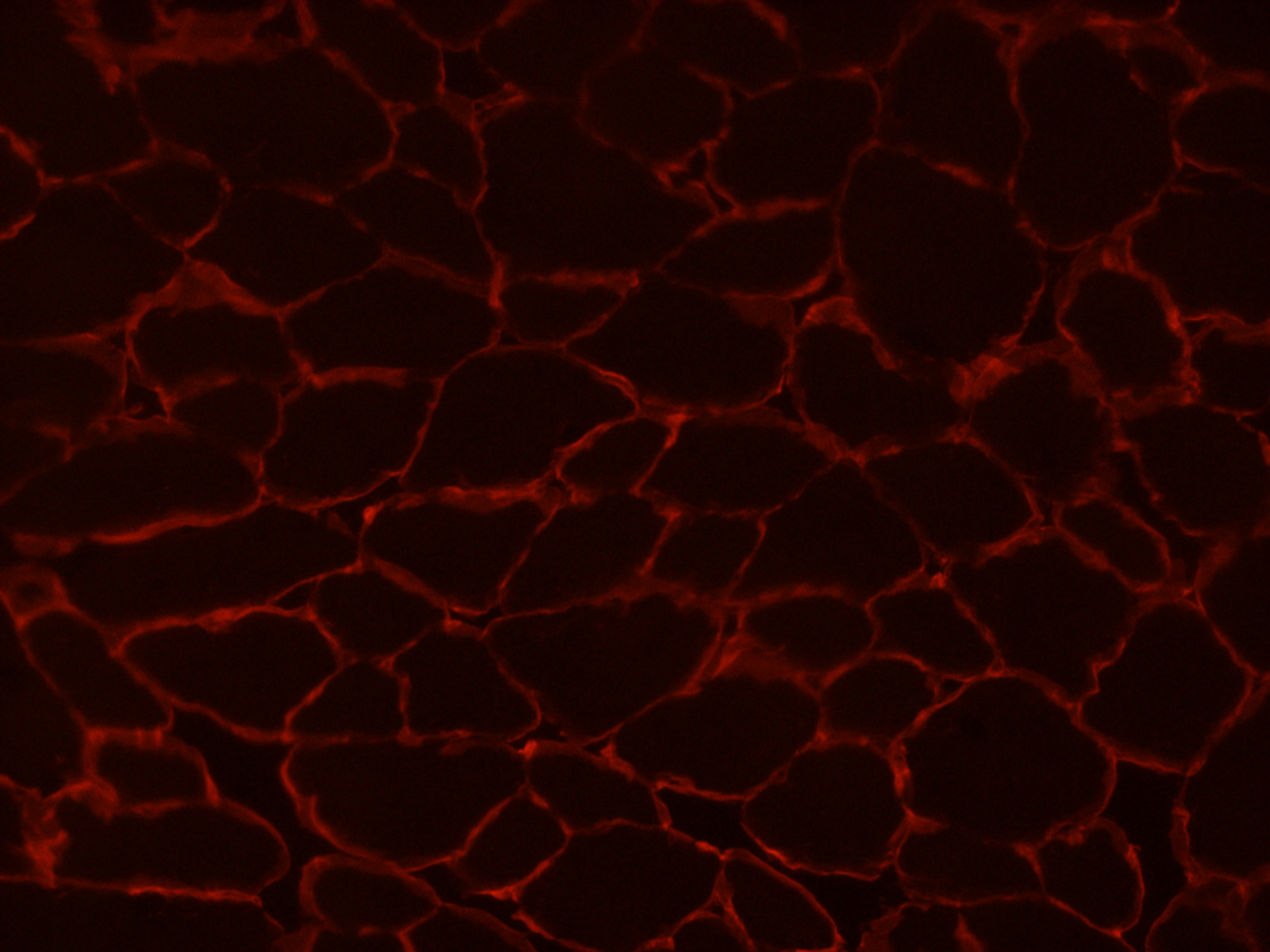

Supplement: Figure 3—source data 1. [file elife-81858-fig3-data1.zip › Figure 3-source data 1/fig3.c/WT-M/_58060.tif]

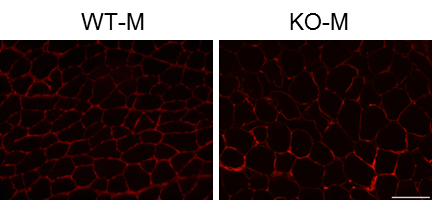

Supplement: Figure 3—source data 1. [file elife-81858-fig3-data1.zip › Figure 3-source data 1/fig3.c/fig3.c.tif]

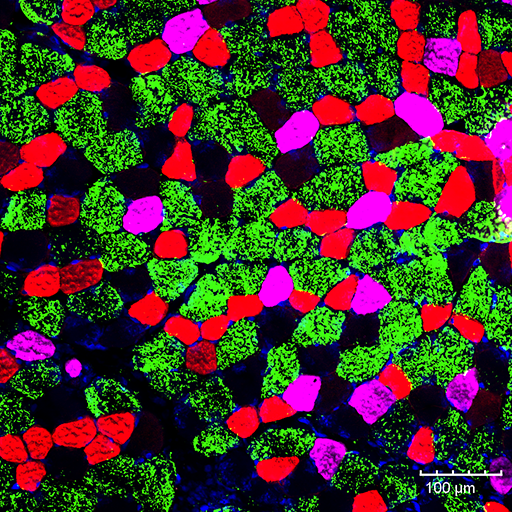

Supplement: Figure 3—source data 1. [file elife-81858-fig3-data1.zip › Figure 3-source data 1/fig3.e/KO-M/KO-M1.tif]

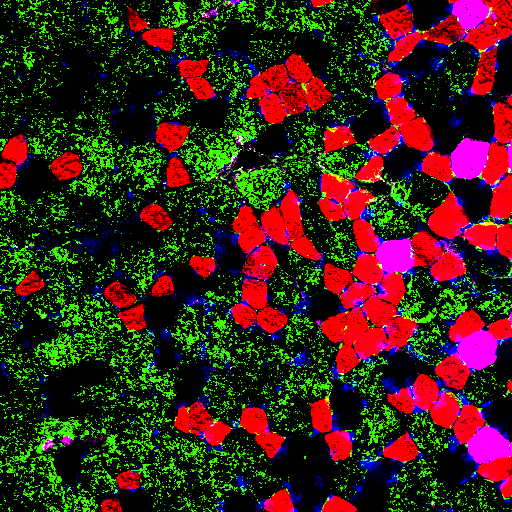

Supplement: Figure 3—source data 1. [file elife-81858-fig3-data1.zip › Figure 3-source data 1/fig3.e/KO-M/KO-M2.tif]

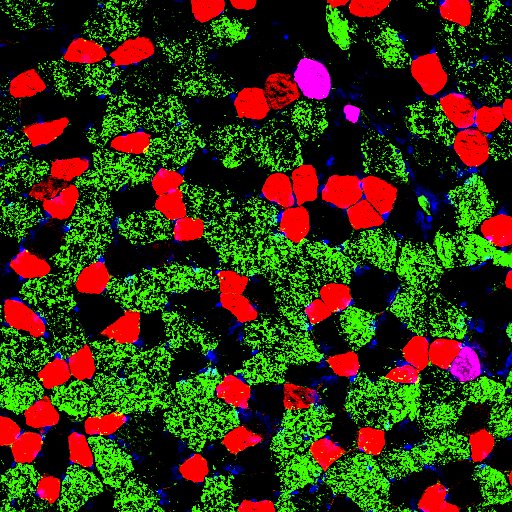

Supplement: Figure 3—source data 1. [file elife-81858-fig3-data1.zip › Figure 3-source data 1/fig3.e/KO-M/KO-M3.tif]

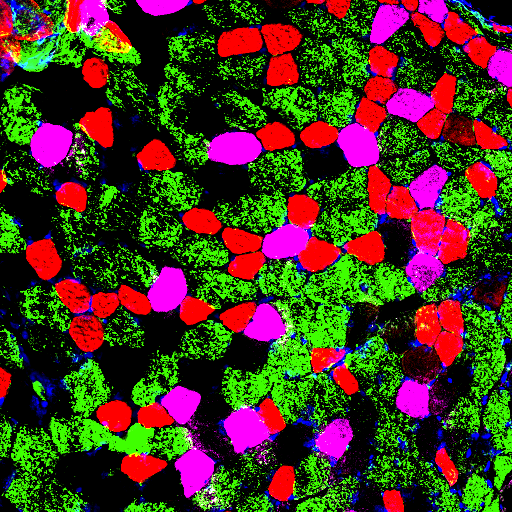

Supplement: Figure 3—source data 1. [file elife-81858-fig3-data1.zip › Figure 3-source data 1/fig3.e/KO-M/KO-M4.tif]

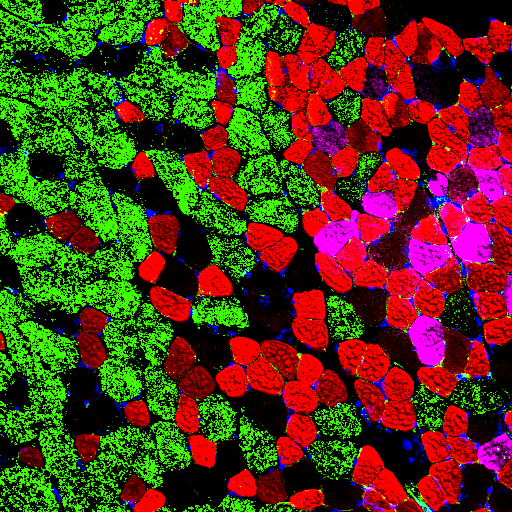

Supplement: Figure 3—source data 1. [file elife-81858-fig3-data1.zip › Figure 3-source data 1/fig3.e/KO-M/KO-M5.tif]

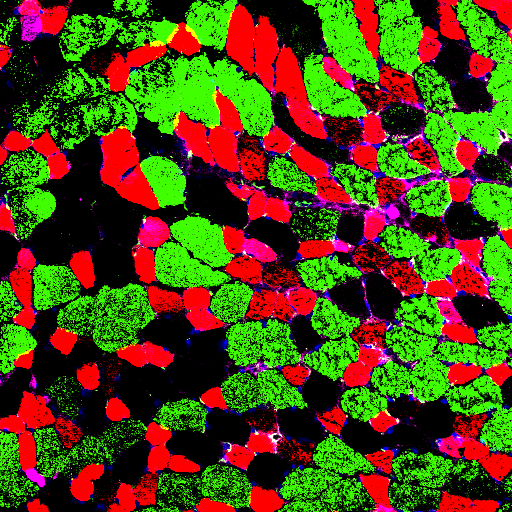

Supplement: Figure 3—source data 1. [file elife-81858-fig3-data1.zip › Figure 3-source data 1/fig3.e/KO-M/KO-M6.tif]

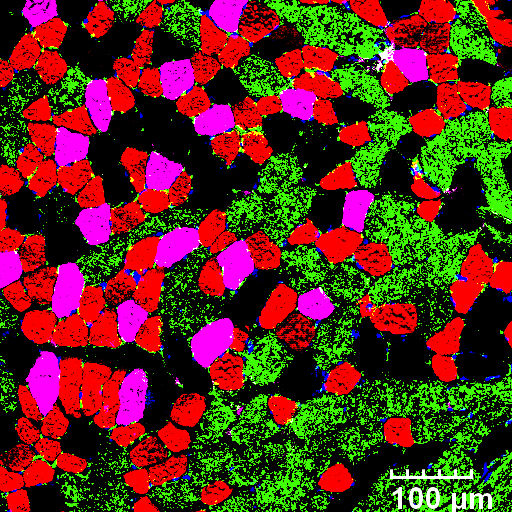

Supplement: Figure 3—source data 1. [file elife-81858-fig3-data1.zip › Figure 3-source data 1/fig3.e/WT-M/WT-M1.tif]

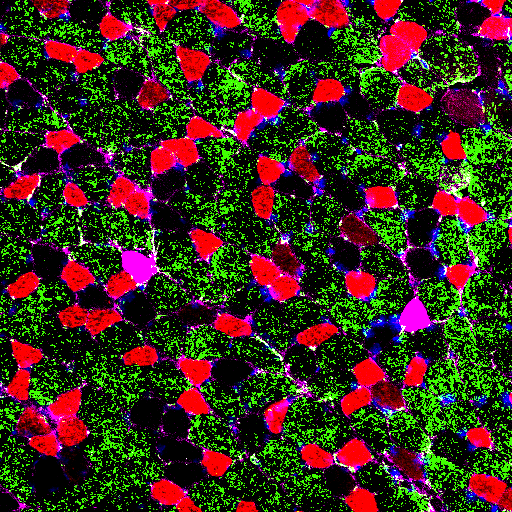

Supplement: Figure 3—source data 1. [file elife-81858-fig3-data1.zip › Figure 3-source data 1/fig3.e/WT-M/WT-M2.tif]

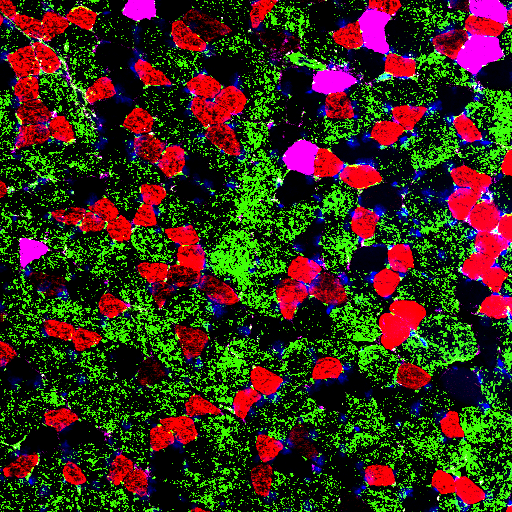

Supplement: Figure 3—source data 1. [file elife-81858-fig3-data1.zip › Figure 3-source data 1/fig3.e/WT-M/WT-M3.tif]

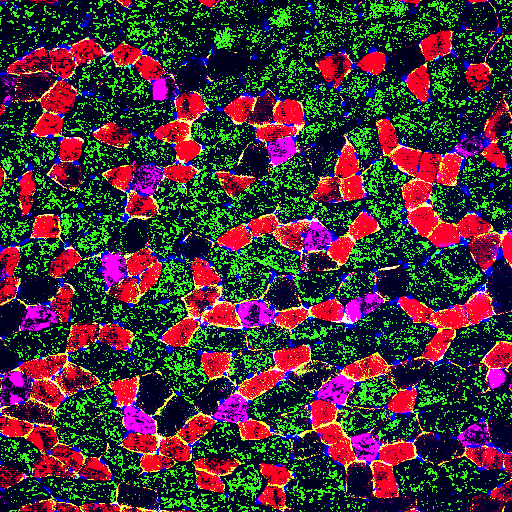

Supplement: Figure 3—source data 1. [file elife-81858-fig3-data1.zip › Figure 3-source data 1/fig3.e/WT-M/WT-M4.tif]

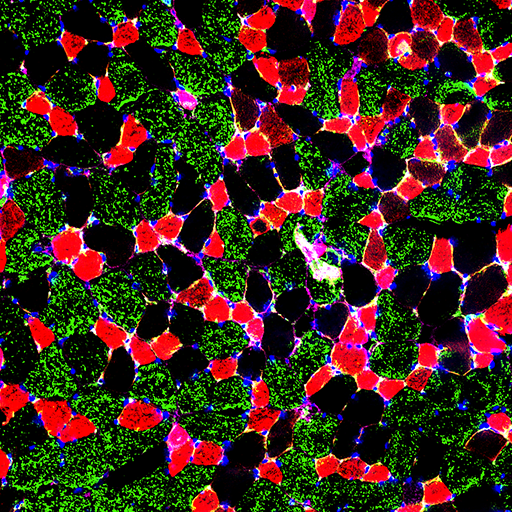

Supplement: Figure 3—source data 1. [file elife-81858-fig3-data1.zip › Figure 3-source data 1/fig3.e/WT-M/WT-M5.tif]

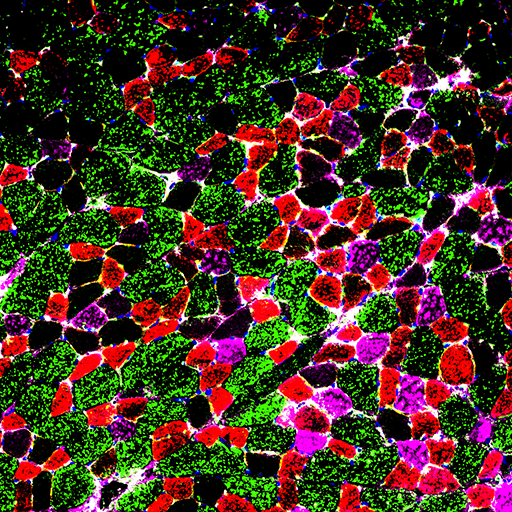

Supplement: Figure 3—source data 1. [file elife-81858-fig3-data1.zip › Figure 3-source data 1/fig3.e/WT-M/WT-M6.tif]

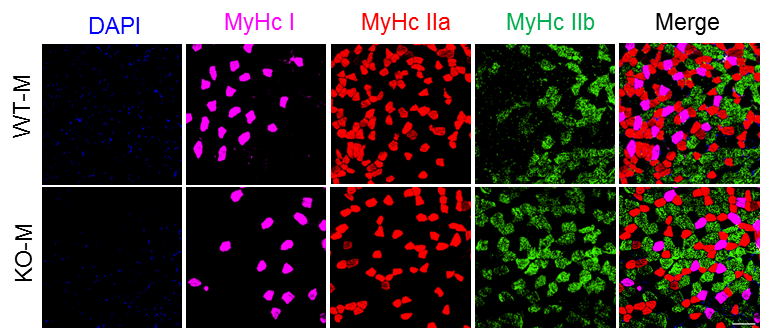

Supplement: Figure 3—source data 1. [file elife-81858-fig3-data1.zip › Figure 3-source data 1/fig3.e/fig3.e.tif]

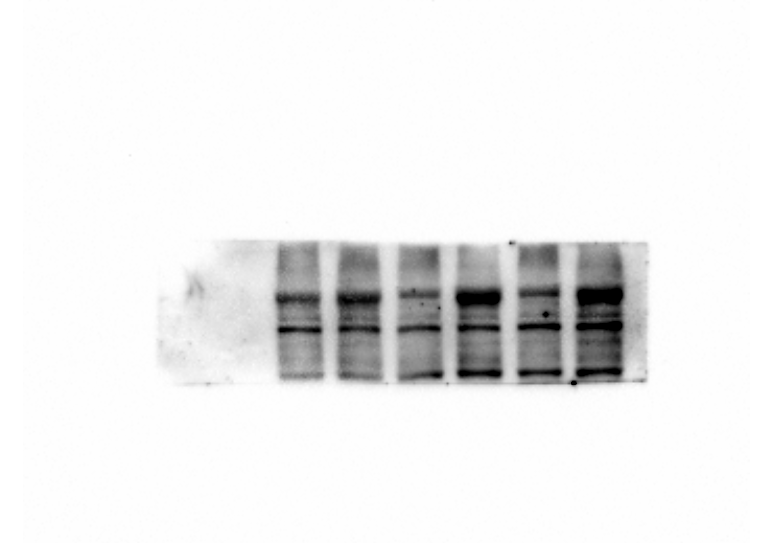

Supplement: Figure 3—source data 2. [file elife-81858-fig3-data2.zip › Figure 3-source data 2/fig3f.Myhc I.tif]

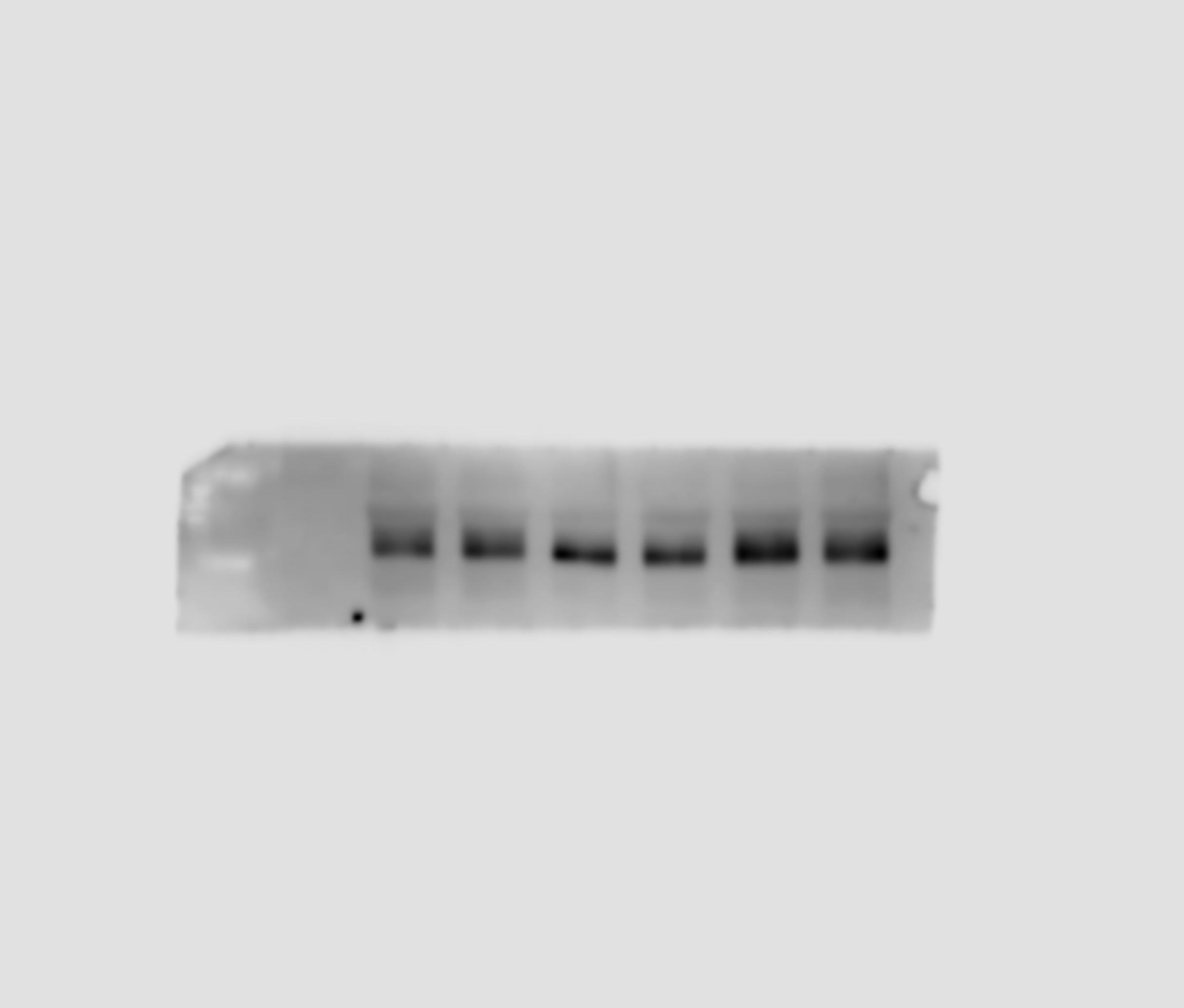

Supplement: Figure 3—source data 2. [file elife-81858-fig3-data2.zip › Figure 3-source data 2/fig3f.MyhcIIa.tif]

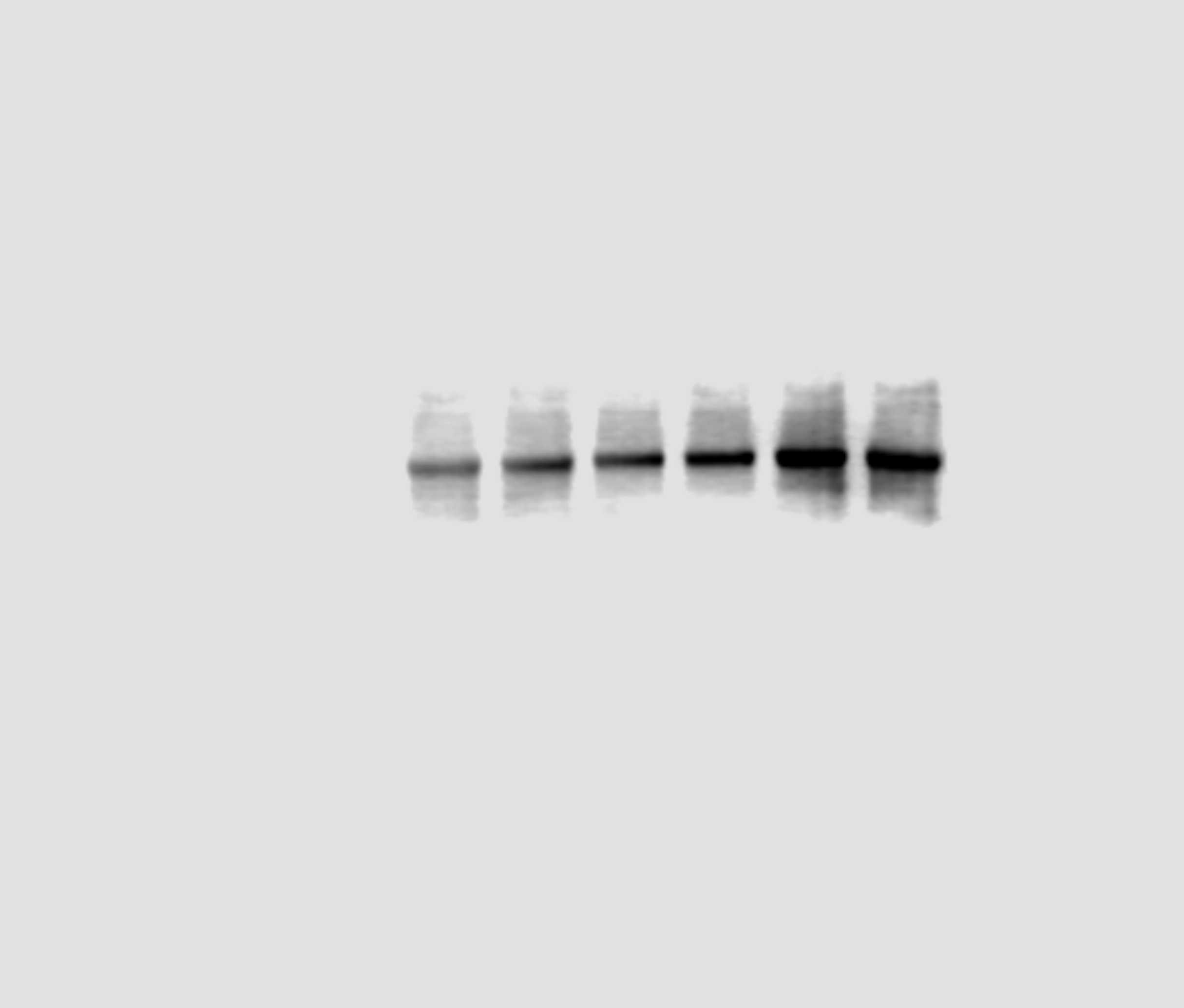

Supplement: Figure 3—source data 2. [file elife-81858-fig3-data2.zip › Figure 3-source data 2/fig3f.MyhcIIb.tif]

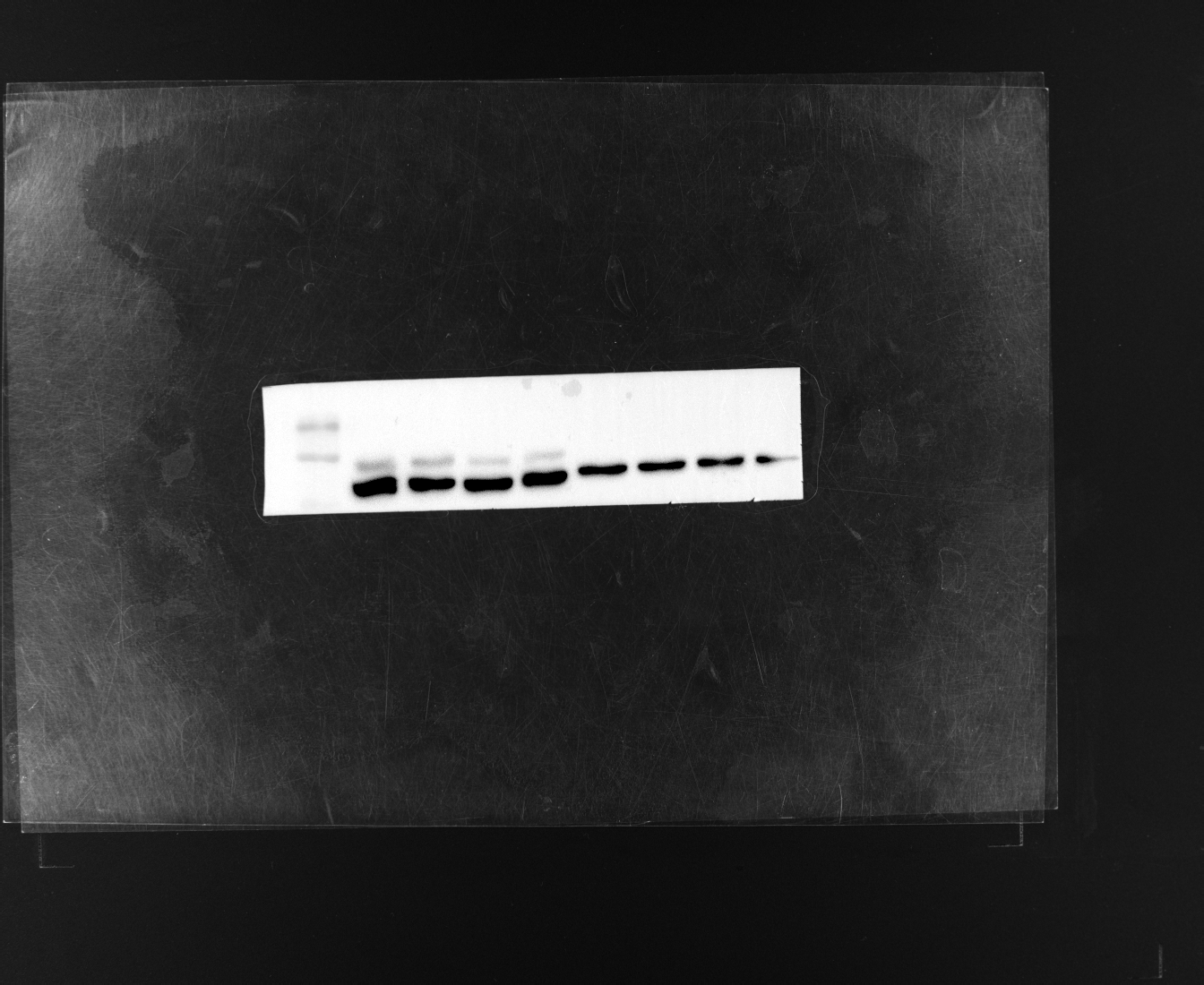

Supplement: Figure 3—source data 2. [file elife-81858-fig3-data2.zip › Figure 3-source data 2/fig3f.Myod.tif]

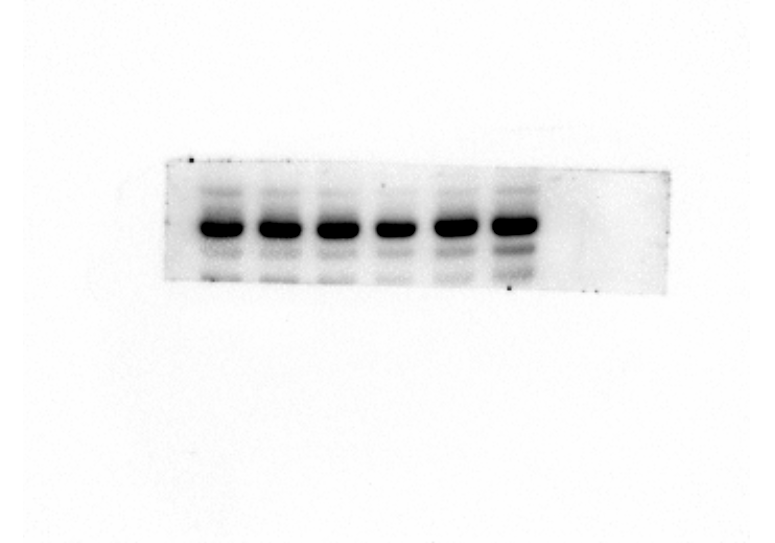

Supplement: Figure 3—source data 2. [file elife-81858-fig3-data2.zip › Figure 3-source data 2/fig3f.actin.tif]

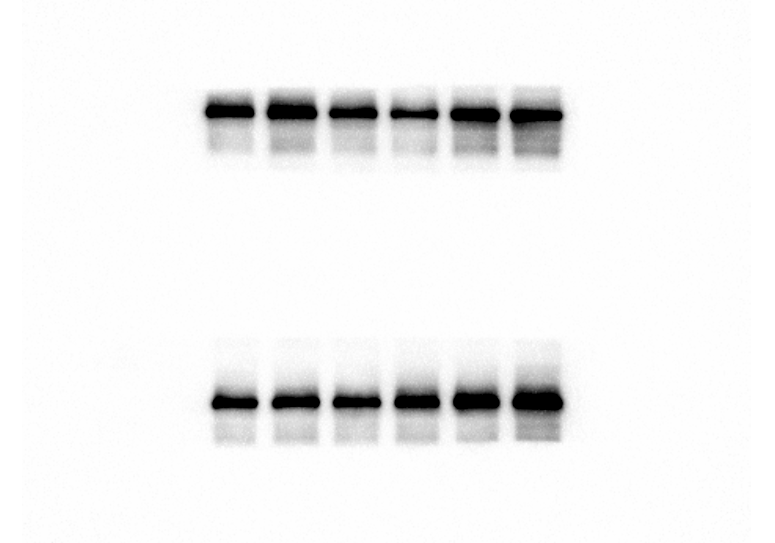

Supplement: Figure 3—source data 2. [file elife-81858-fig3-data2.zip › Figure 3-source data 2/fig3g.HK2.tif]

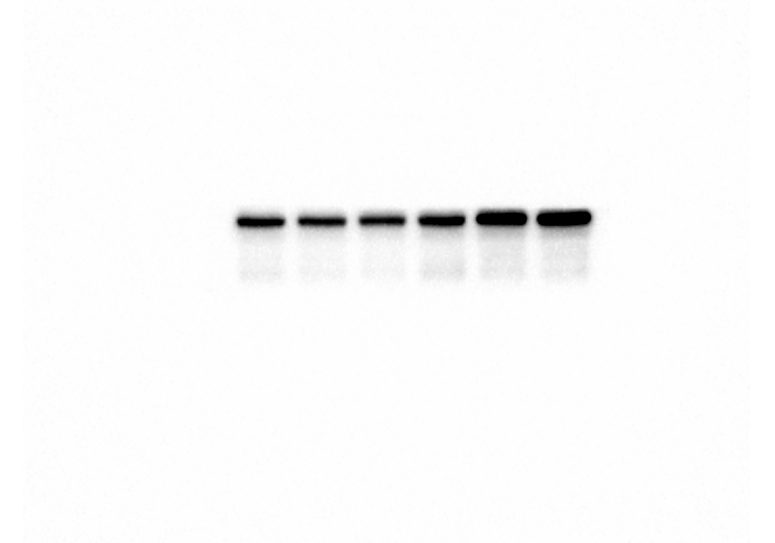

Supplement: Figure 3—source data 2. [file elife-81858-fig3-data2.zip › Figure 3-source data 2/fig3g.PFK1.tif]

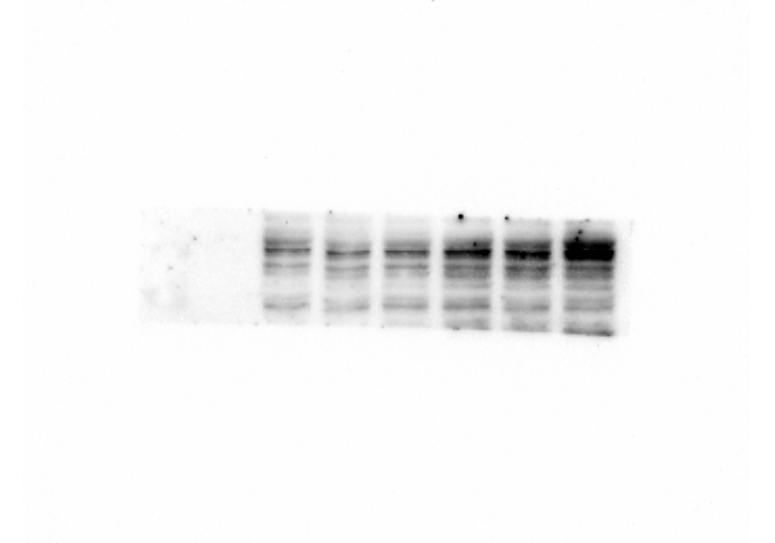

Supplement: Figure 3—source data 2. [file elife-81858-fig3-data2.zip › Figure 3-source data 2/fig3g.PKM2.tif]

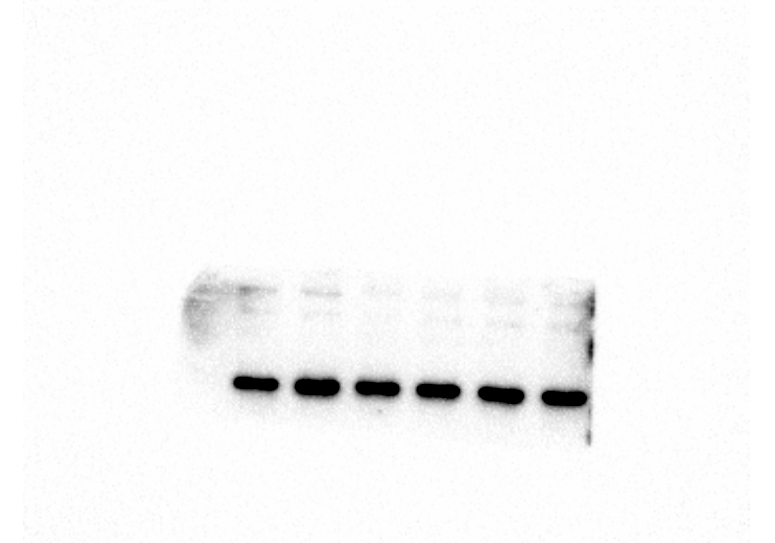

Supplement: Figure 3—source data 2. [file elife-81858-fig3-data2.zip › Figure 3-source data 2/fig3g.actin.tif]

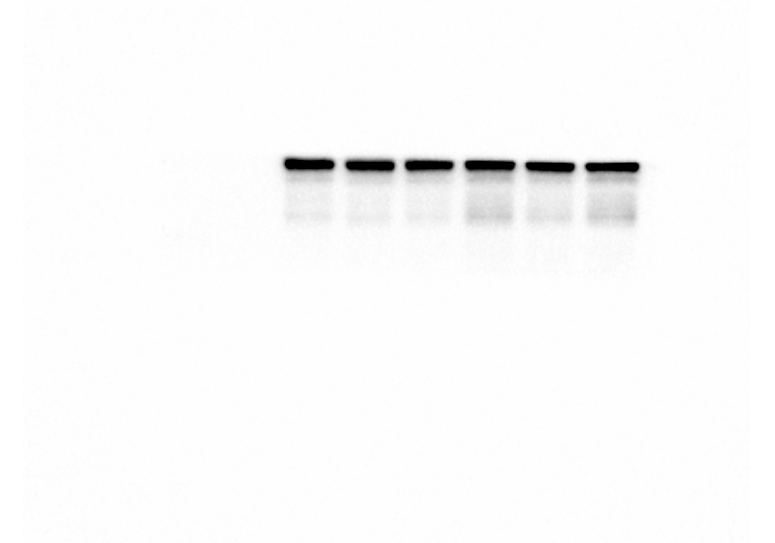

Supplement: Figure 3—source data 2. [file elife-81858-fig3-data2.zip › Figure 3-source data 2/fig3h.actin.tif]

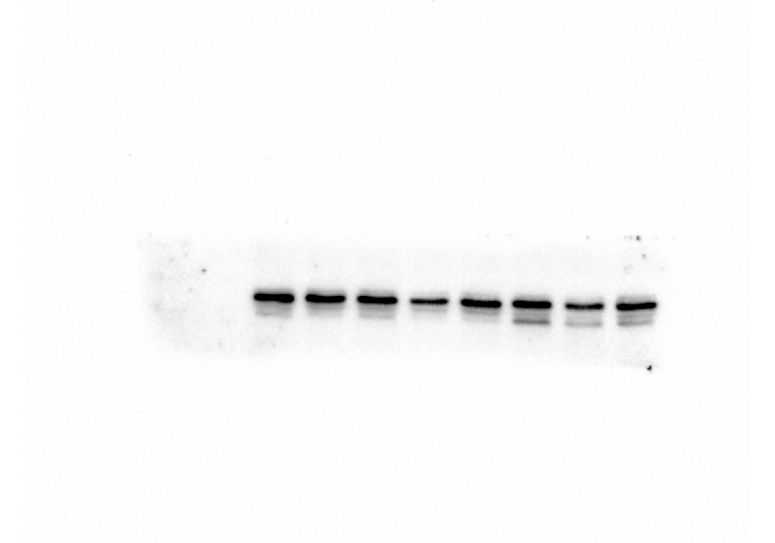

Supplement: Figure 3—source data 2. [file elife-81858-fig3-data2.zip › Figure 3-source data 2/fig3h.akt1.tif]

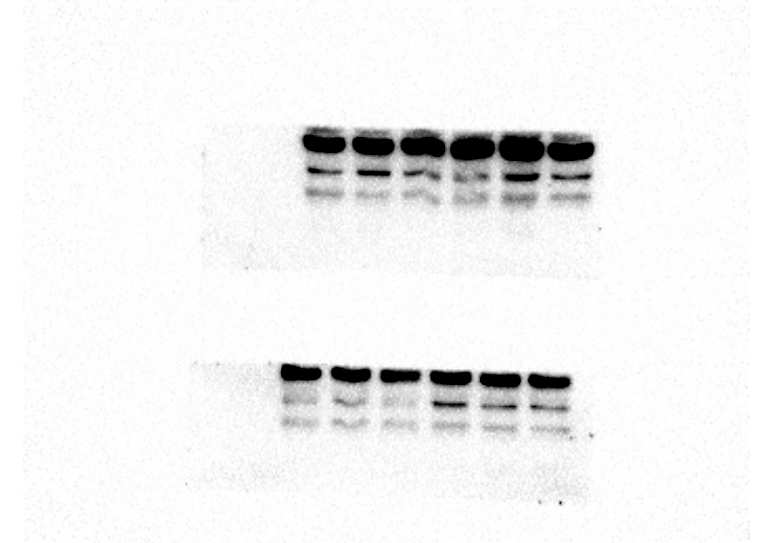

Supplement: Figure 3—source data 2. [file elife-81858-fig3-data2.zip › Figure 3-source data 2/fig3h.mTOR.tif]

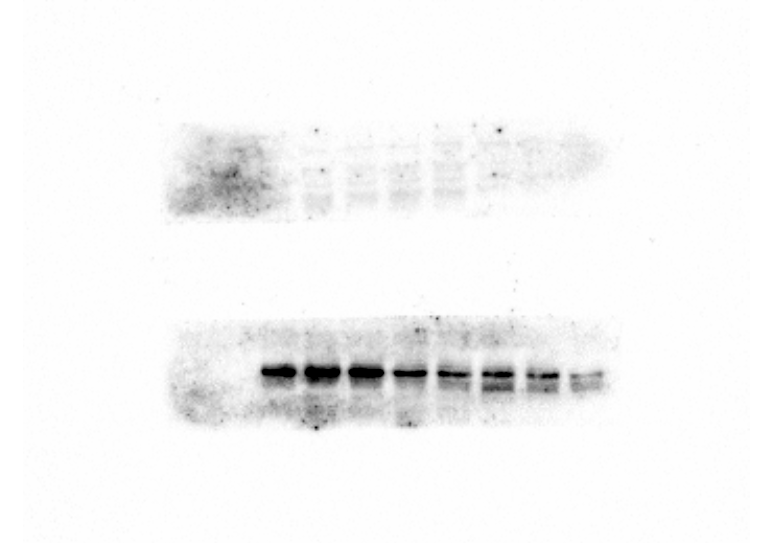

Supplement: Figure 3—source data 2. [file elife-81858-fig3-data2.zip › Figure 3-source data 2/fig3h.p-akt1.tif]

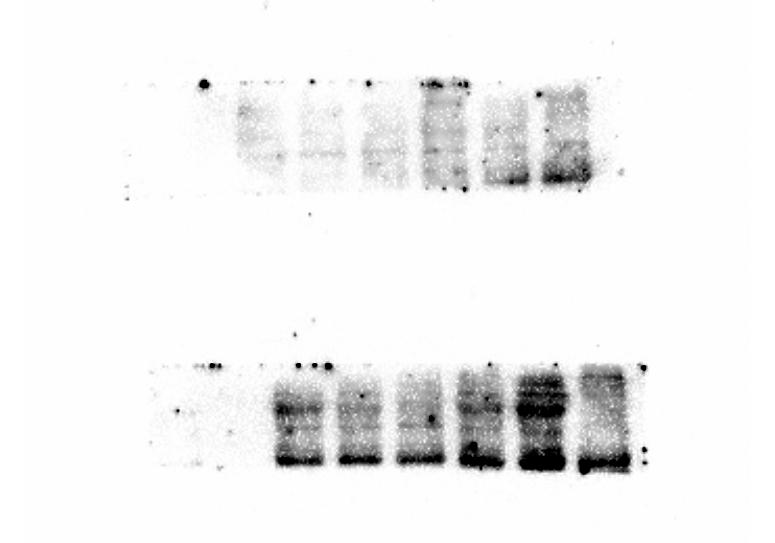

Supplement: Figure 3—source data 2. [file elife-81858-fig3-data2.zip › Figure 3-source data 2/fig3h.p-mTOR.tif]

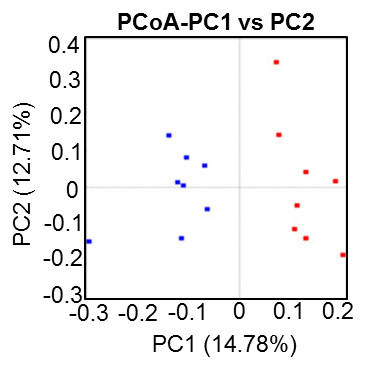

Supplement: Figure 4—source data 1. [file elife-81858-fig4-data1.zip › Figure 4-source data 1/fig4.a/fig4.a.tif]

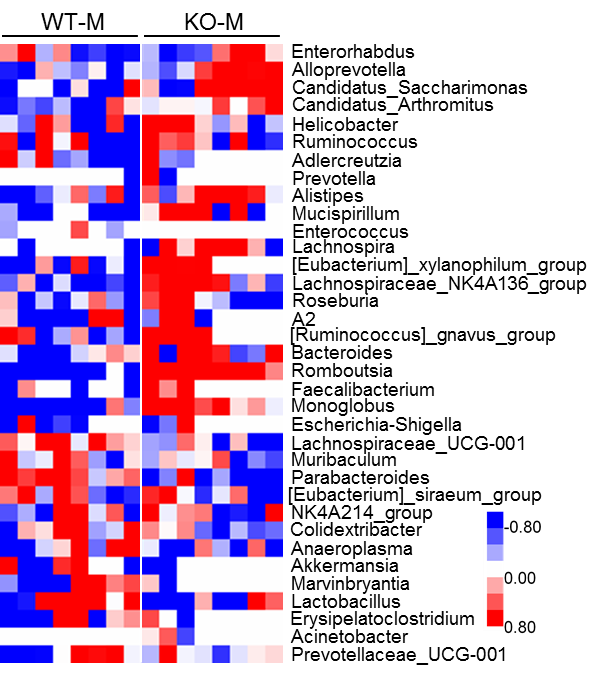

Supplement: Figure 4—source data 1. [file elife-81858-fig4-data1.zip › Figure 4-source data 1/fig4.c/fig4.c.tif]

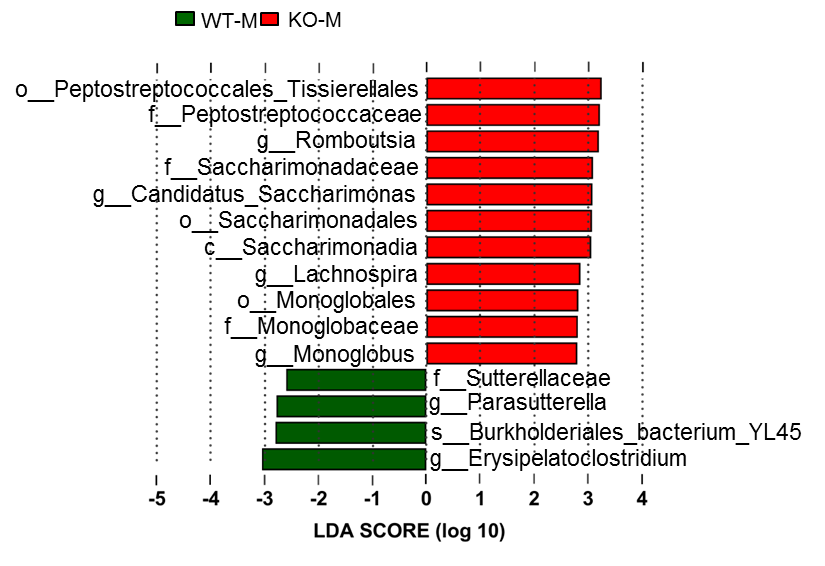

Supplement: Figure 4—source data 1. [file elife-81858-fig4-data1.zip › Figure 4-source data 1/fig4.d/LDA-WT-Mvs.KO-M.tif]

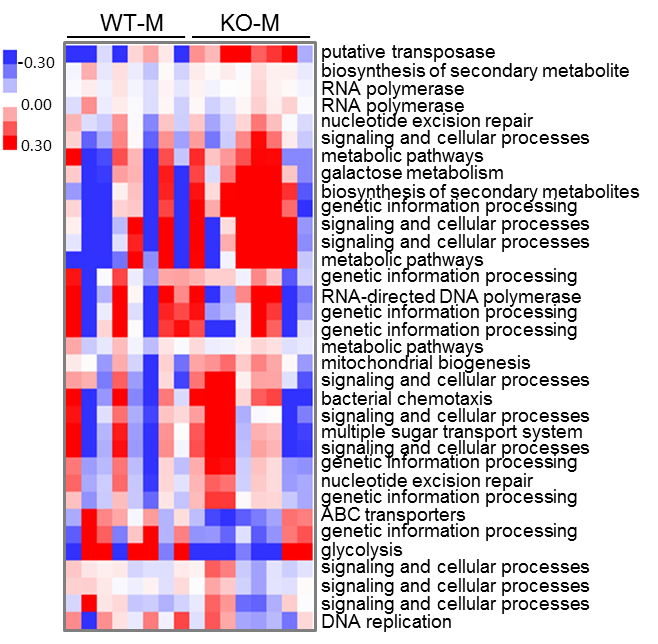

Supplement: Figure 4—source data 1. [file elife-81858-fig4-data1.zip › Figure 4-source data 1/fig4.e/fig4.e.tif]

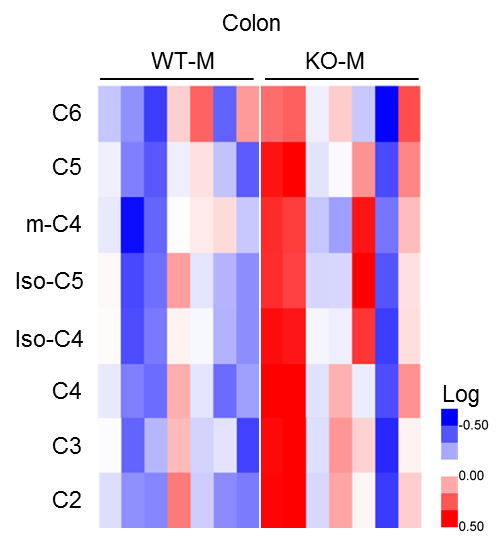

Supplement: Figure 5—source data 1. [file elife-81858-fig5-data1.zip › Figure 5-source data 1/fig5.d/Heatmap-WT-Mvs.KO-M.tif]

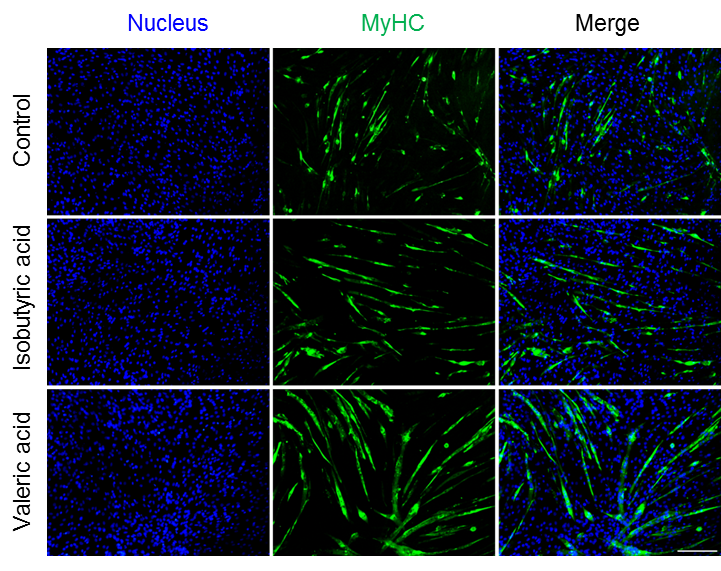

Supplement: Figure 6—source data 1. [file elife-81858-fig6-data1.zip › Figure 6-source data 1/fig6.a/fig6.a.tif]

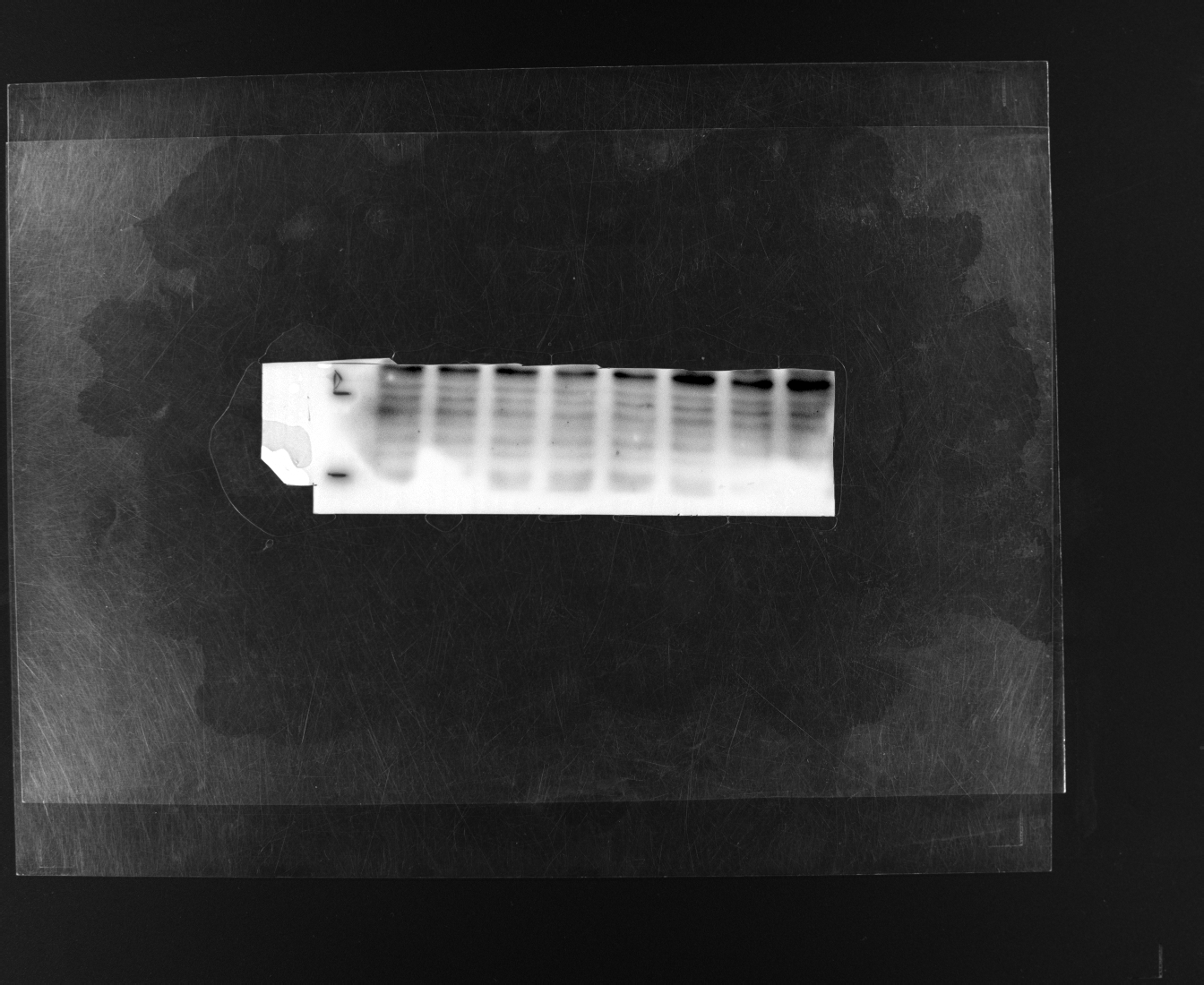

Supplement: Figure 6—source data 2. [file elife-81858-fig6-data2.zip › Figure 6-source data 2/fig6b.MyoD.tif]

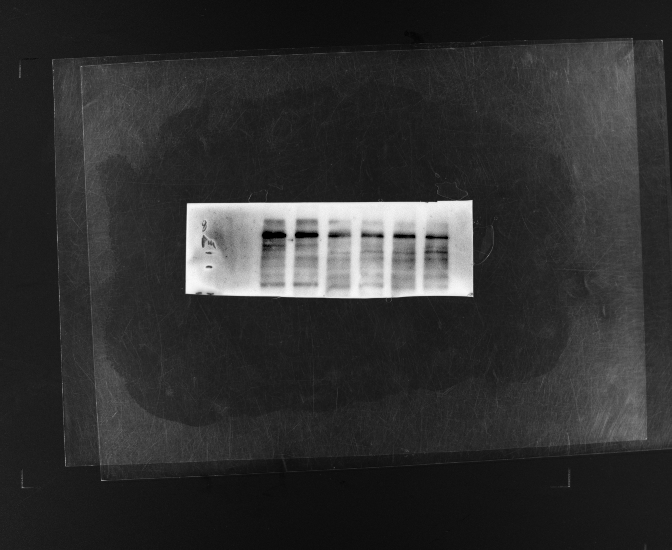

Supplement: Figure 6—source data 2. [file elife-81858-fig6-data2.zip › Figure 6-source data 2/fig6b.MyoG.tif]

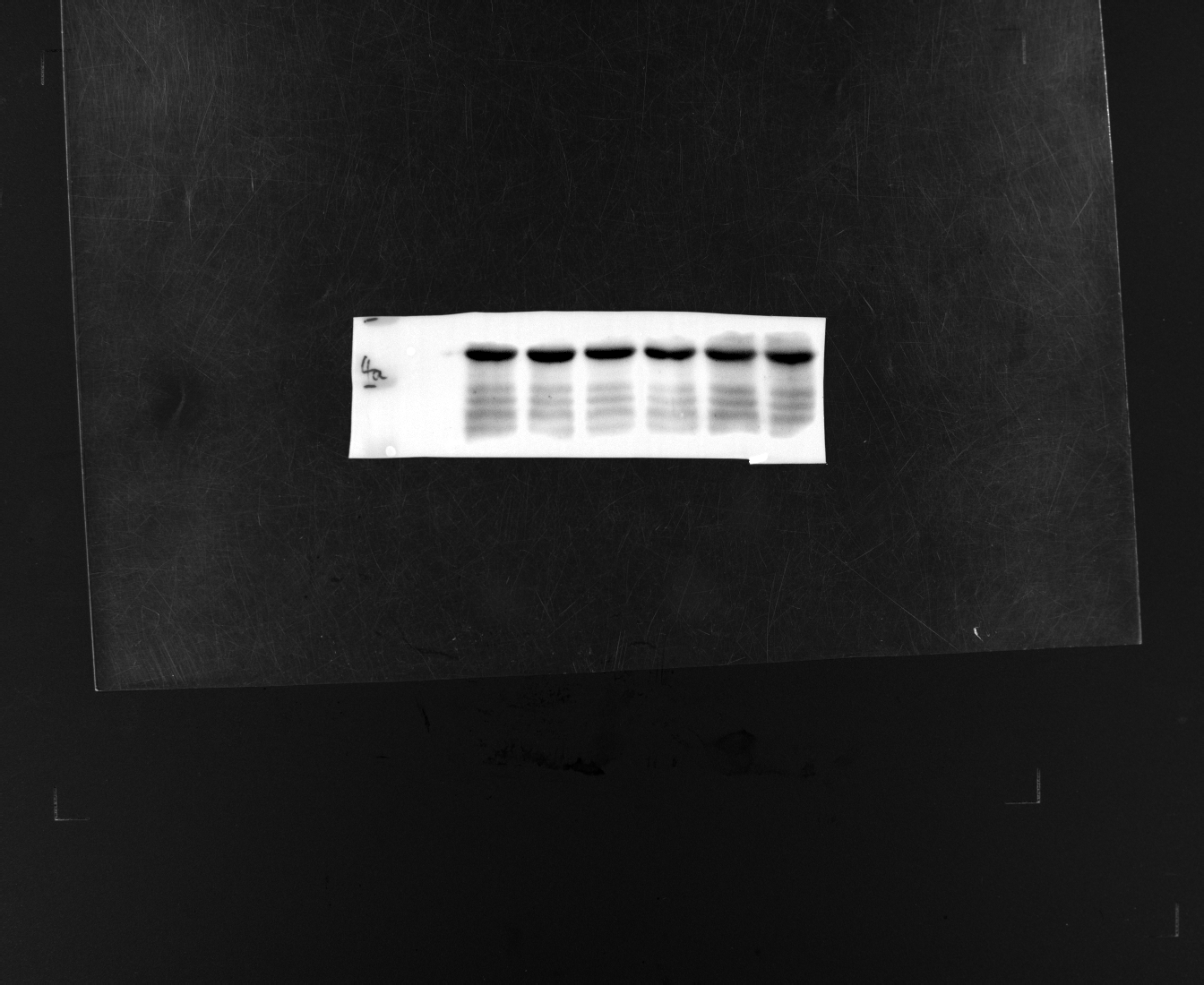

Supplement: Figure 6—source data 2. [file elife-81858-fig6-data2.zip › Figure 6-source data 2/fig6b.actin.tif]

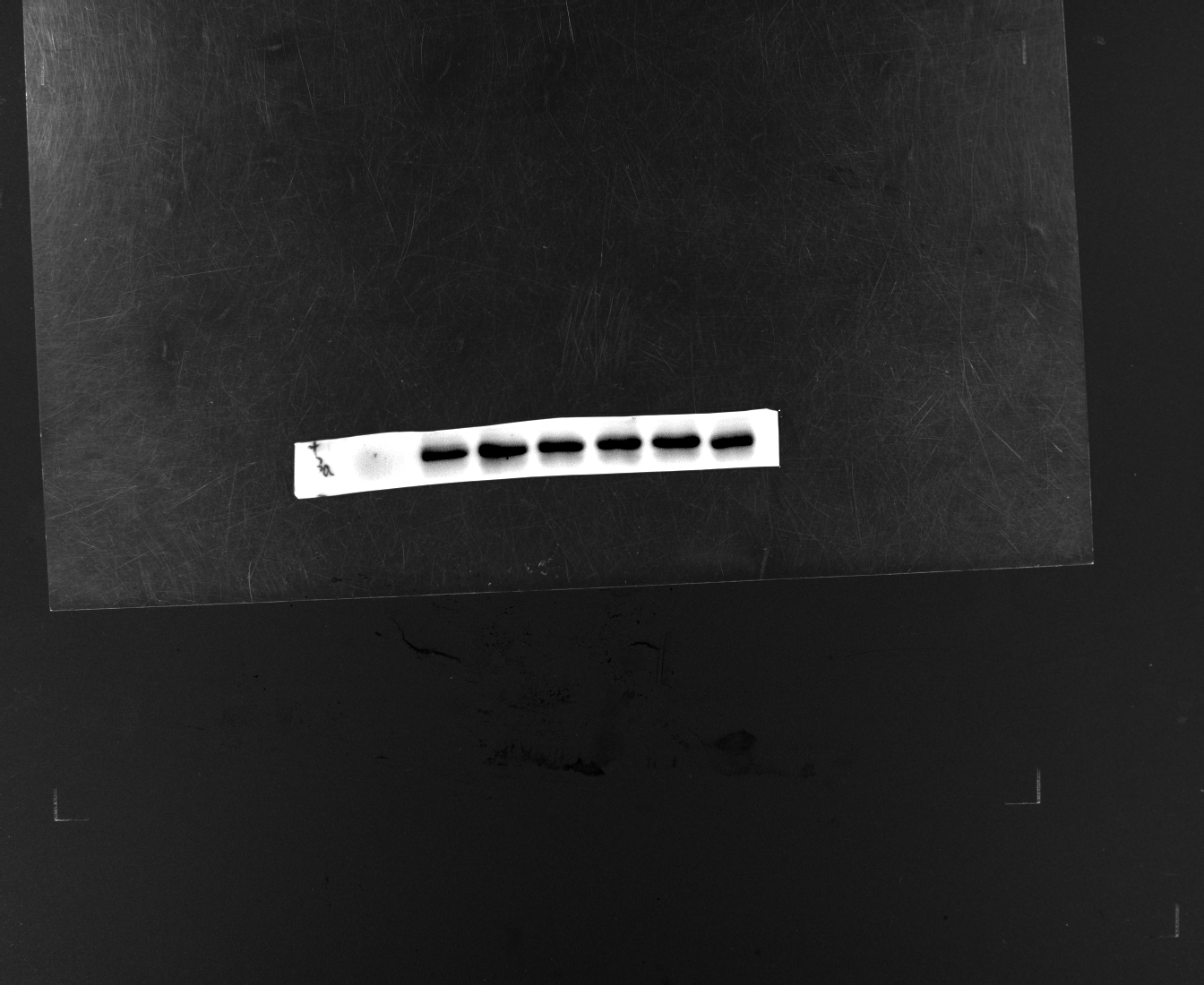

Supplement: Figure 6—source data 2. [file elife-81858-fig6-data2.zip › Figure 6-source data 2/fig6c.actin.tif]

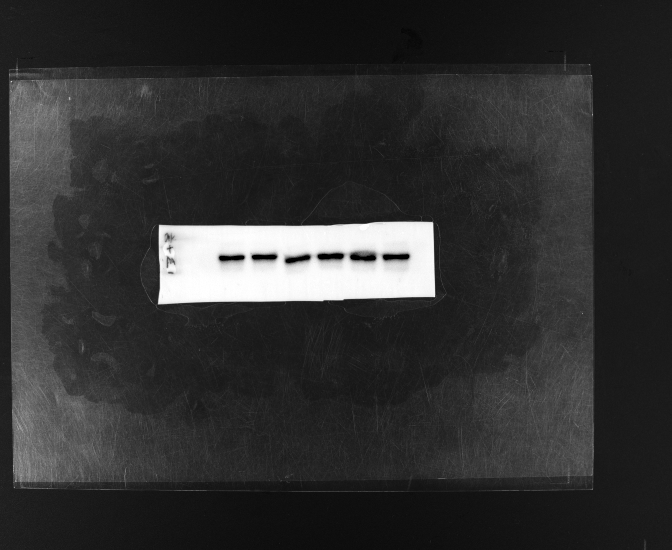

Supplement: Figure 6—source data 2. [file elife-81858-fig6-data2.zip › Figure 6-source data 2/fig6c.akt1.tif]

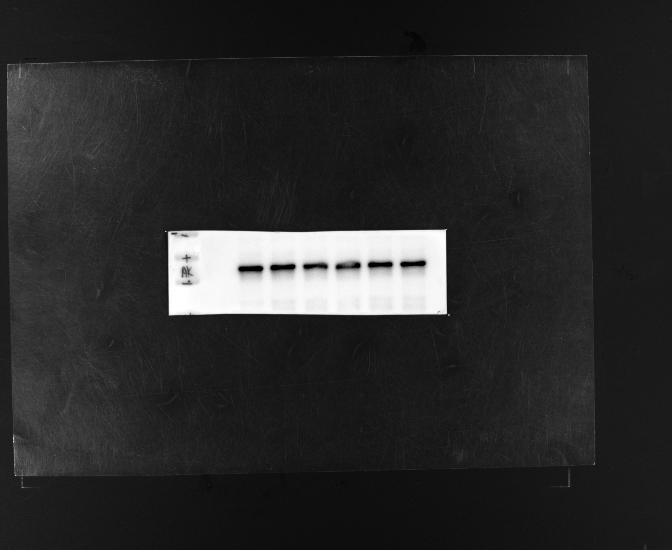

Supplement: Figure 6—source data 2. [file elife-81858-fig6-data2.zip › Figure 6-source data 2/fig6c.mTOR.tif]

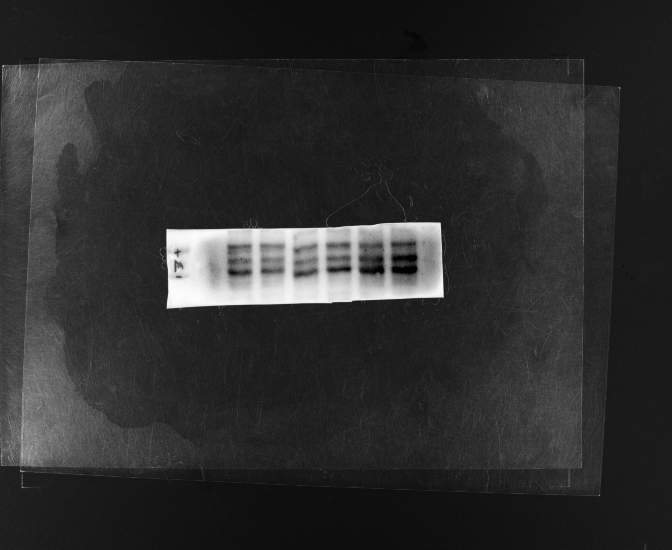

Supplement: Figure 6—source data 2. [file elife-81858-fig6-data2.zip › Figure 6-source data 2/fig6c.p-akt1.tif]

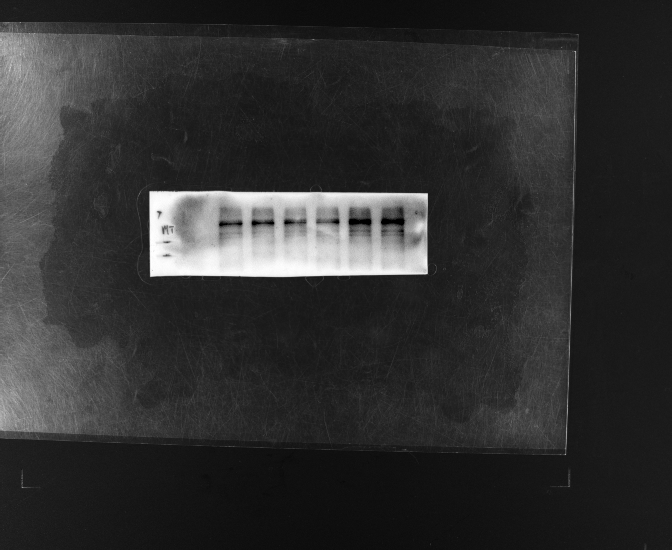

Supplement: Figure 6—source data 2. [file elife-81858-fig6-data2.zip › Figure 6-source data 2/fig6c.p-mTOR.tif]
