# Supplementary material for: Fecal transplant from myostatin deletion pigs positively impacts the gut-muscle axis
Source: eLife. 2023 Apr 11;12:e81858. doi: 10.7554/eLife.81858 (PMC10121221; doi:10.7554/eLife.81858)

Figure 1C source data

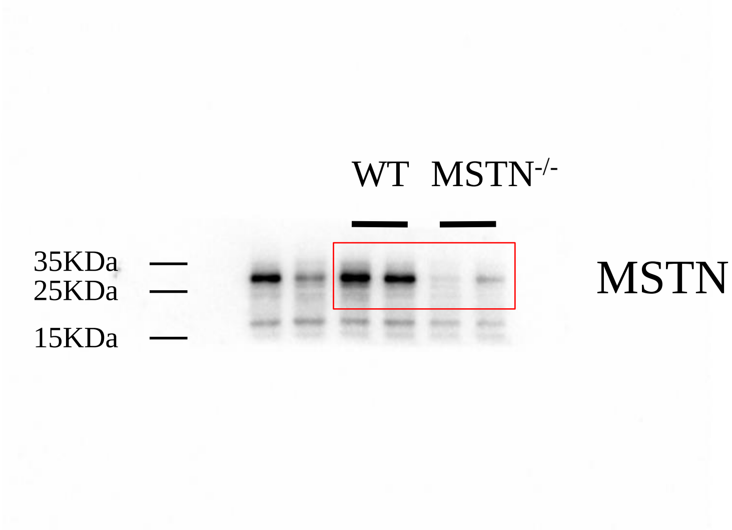

MSTN

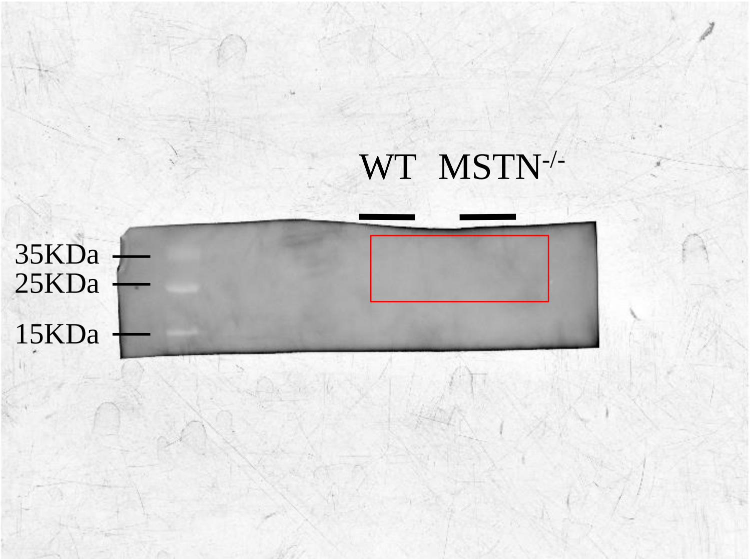

Figure 1C source data

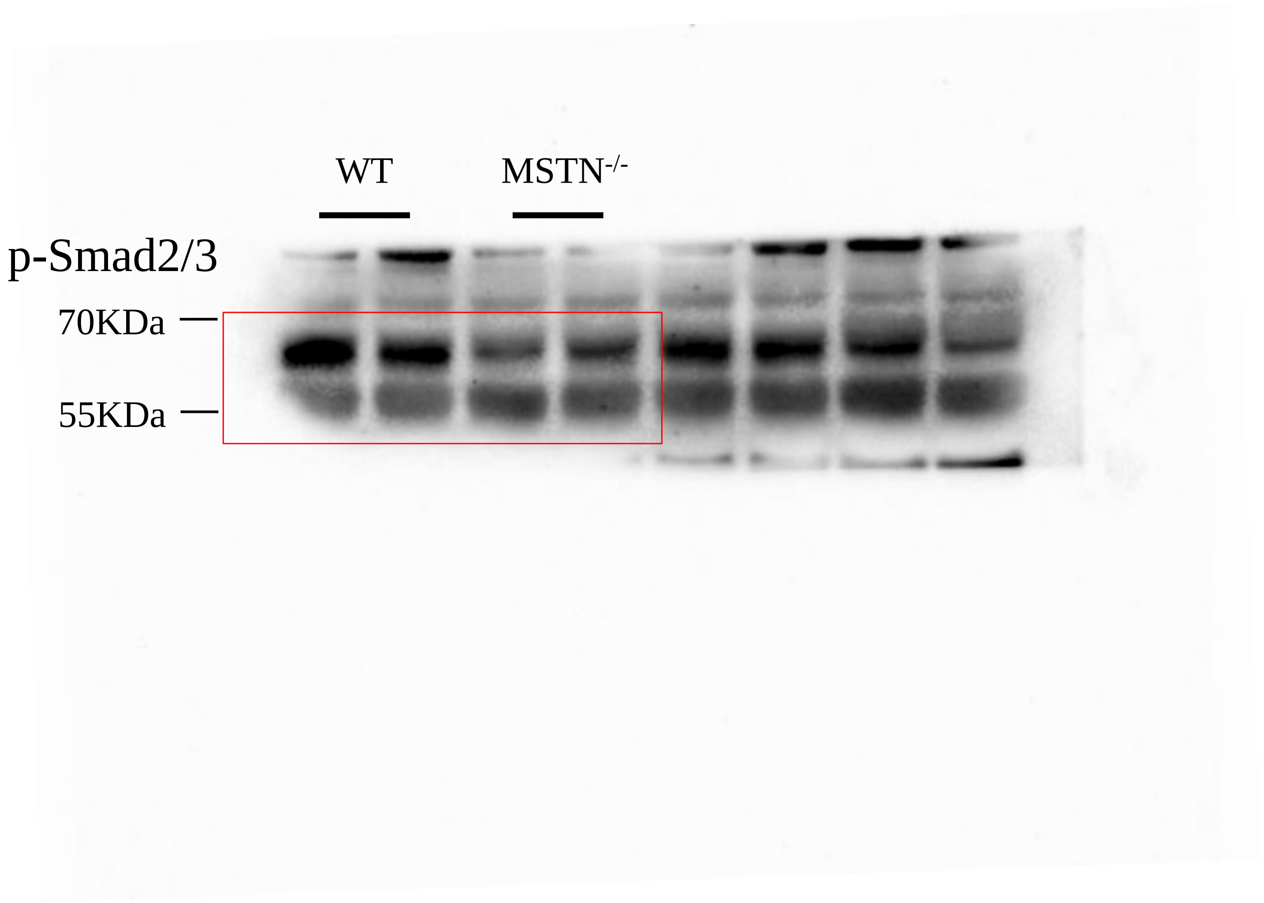

Figure 1C source data

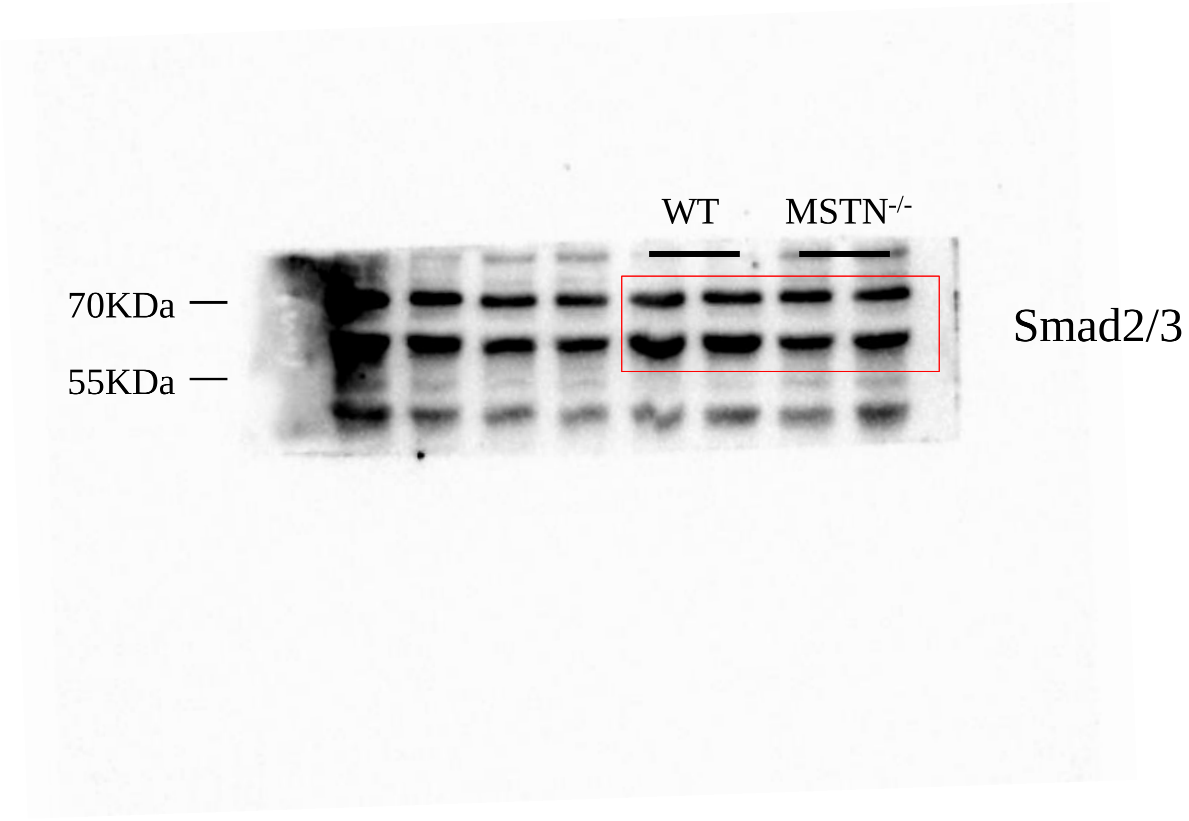

Figure 1C source data

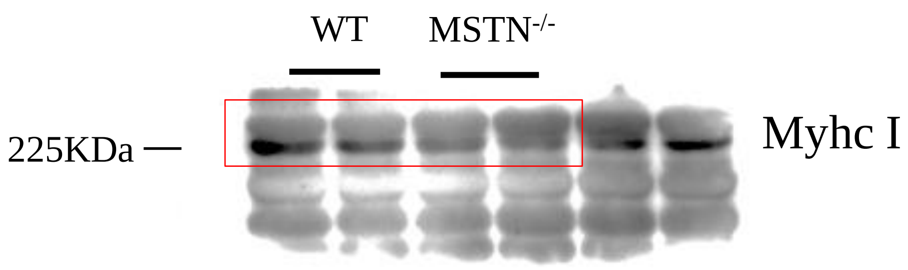

Figure 1C source data

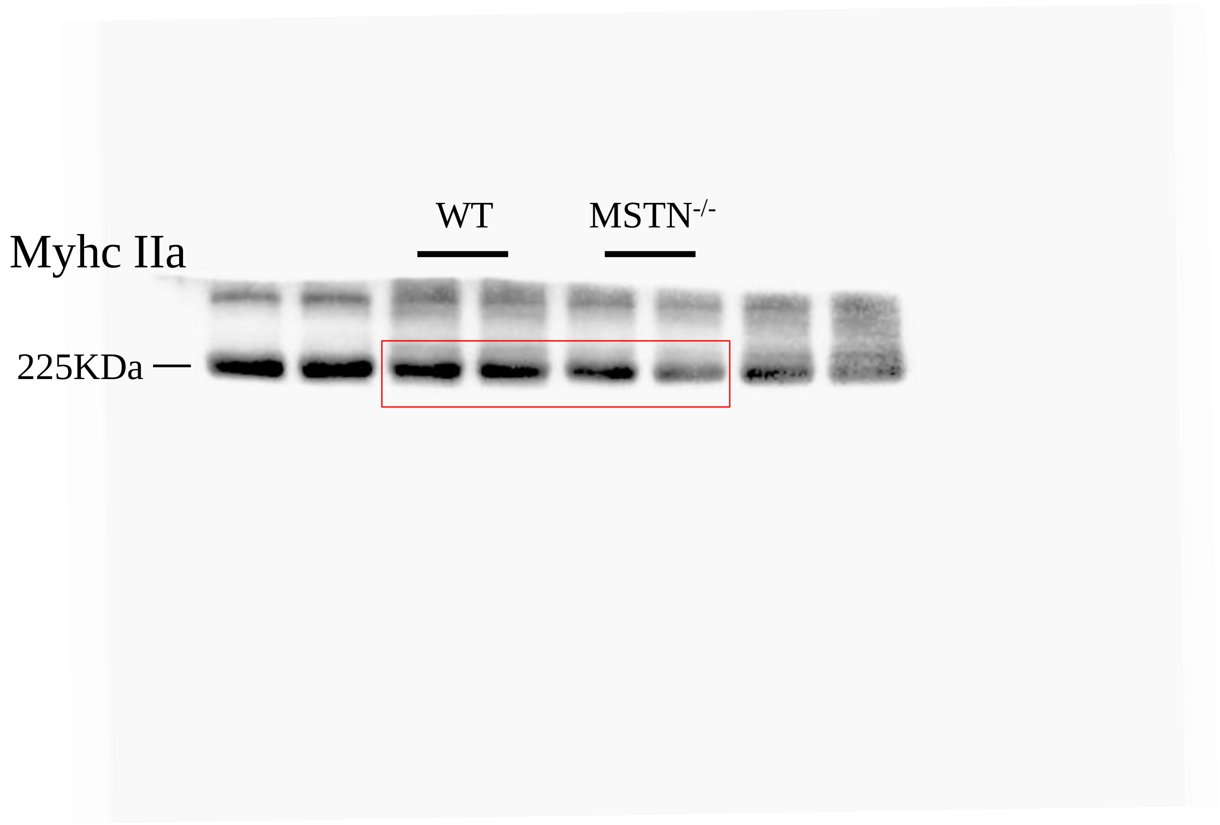

Figure 1C source data

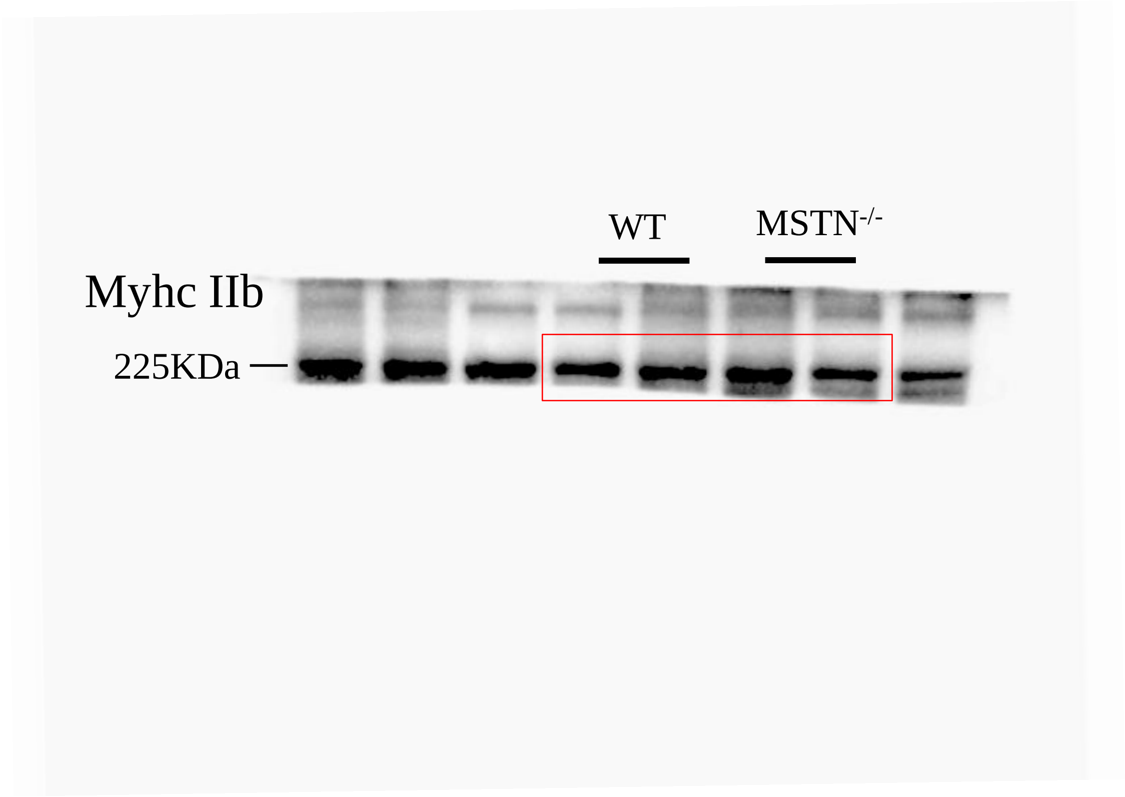

Figure 1C source data

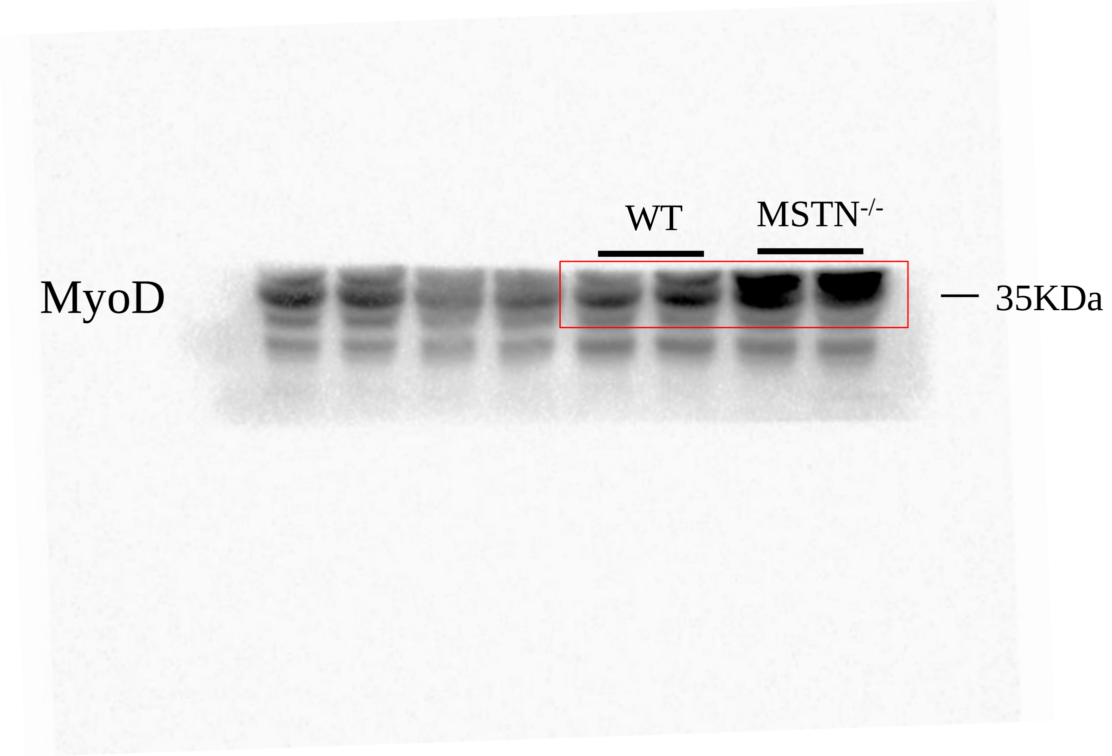

Figure 1C source data

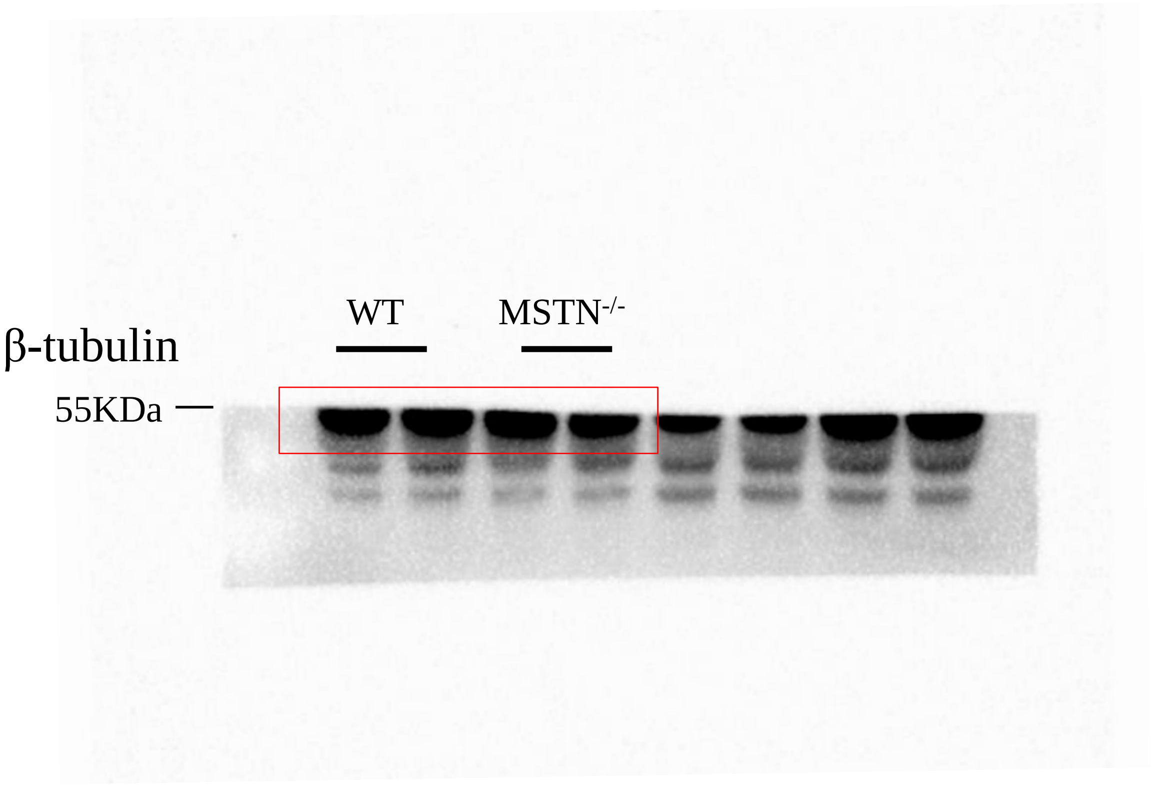

Figure 1D source data

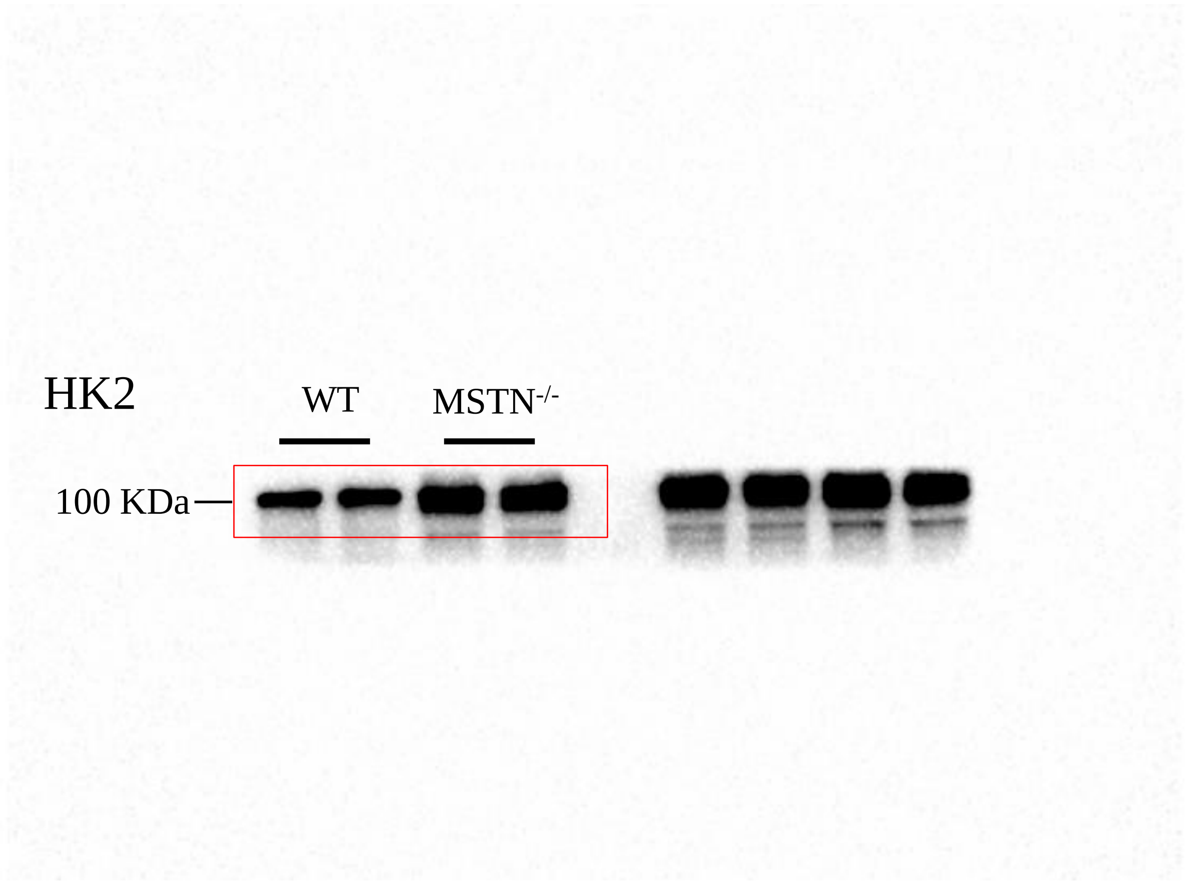

Figure 1D source data

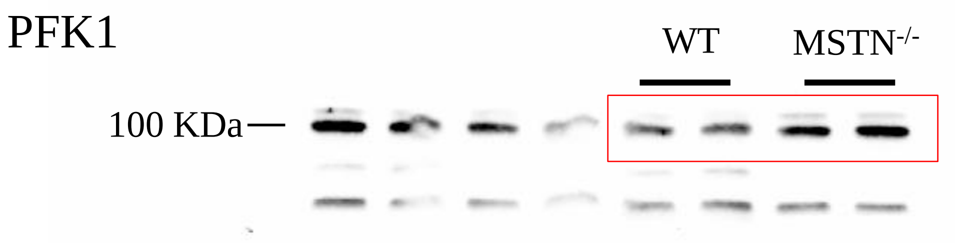

Figure 1D source data

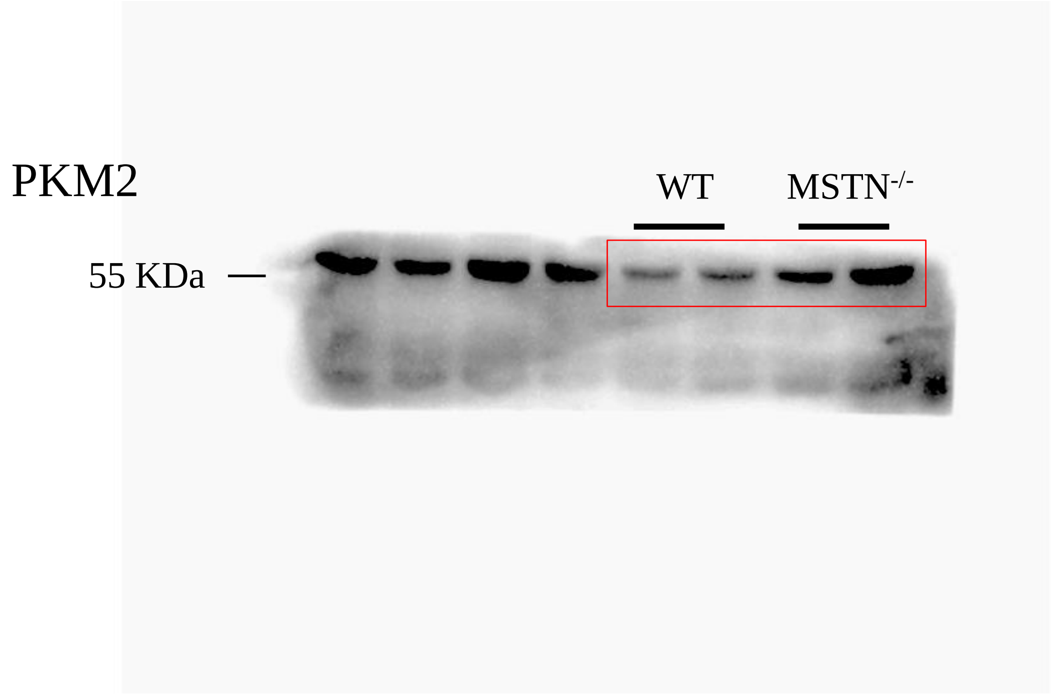

Figure 1D source data

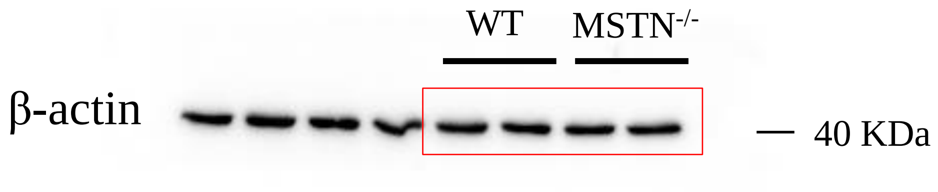

Supplement: Figure 1—source data 2. [file elife-81858-fig1-data2.zip › Figure 1-source data 2/Raw western blot images for Figure 1.pdf]
