# Supplementary material for: Fecal transplant from myostatin deletion pigs positively impacts the gut-muscle axis
Source: eLife. 2023 Apr 11;12:e81858. doi: 10.7554/eLife.81858 (PMC10121221; doi:10.7554/eLife.81858)

Figure 2B source data

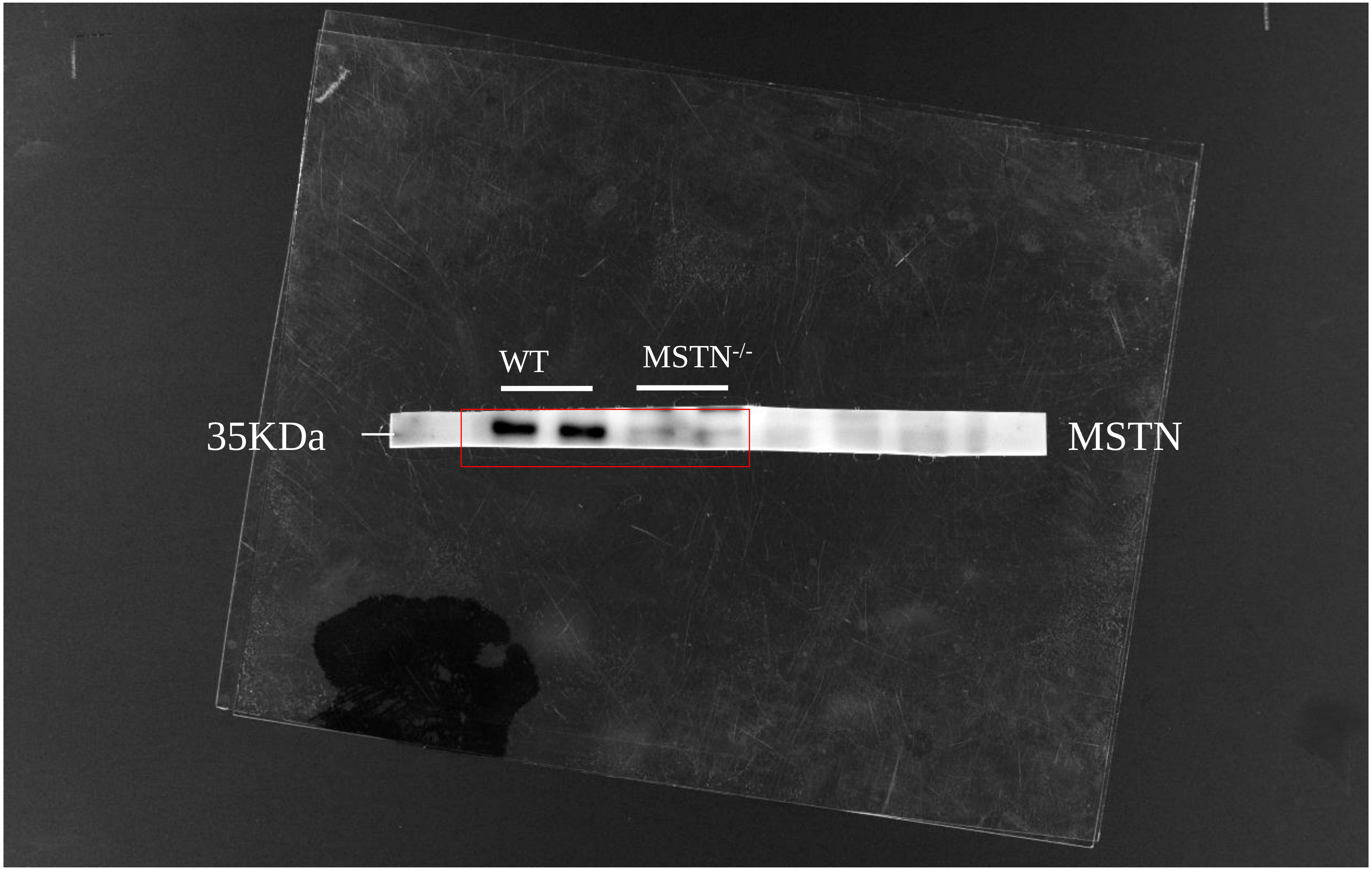

Figure 2B source data

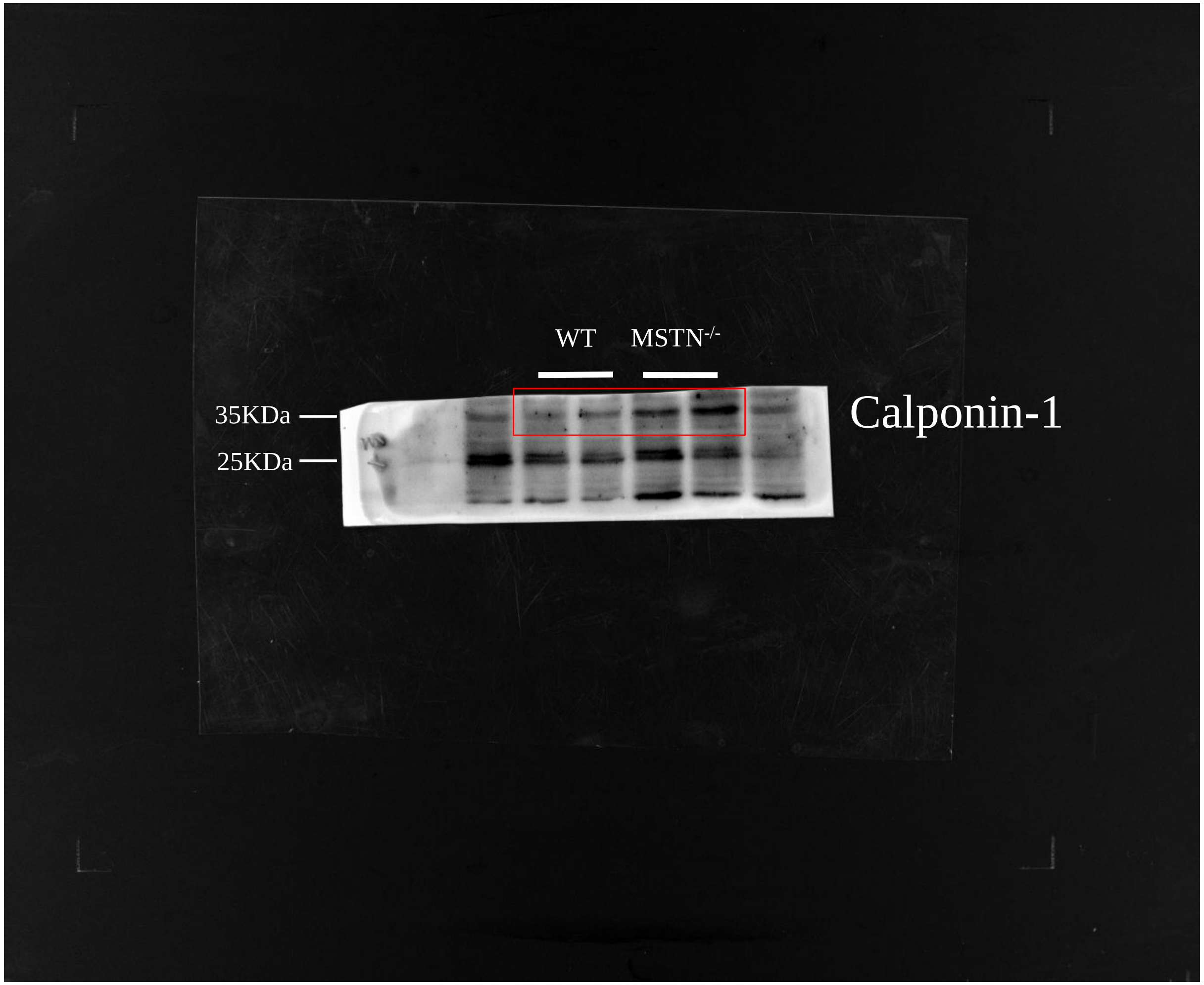

Figure 2B source data

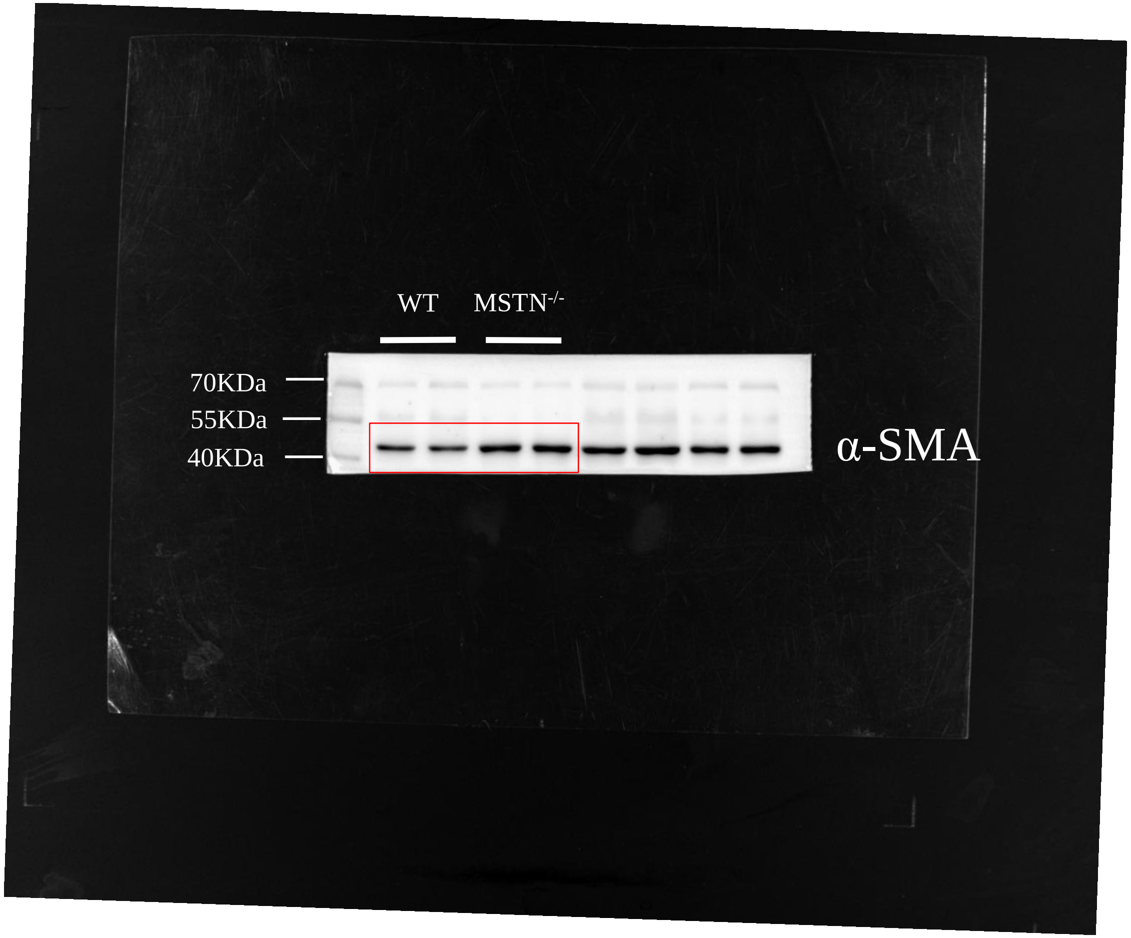

Figure 2B source data

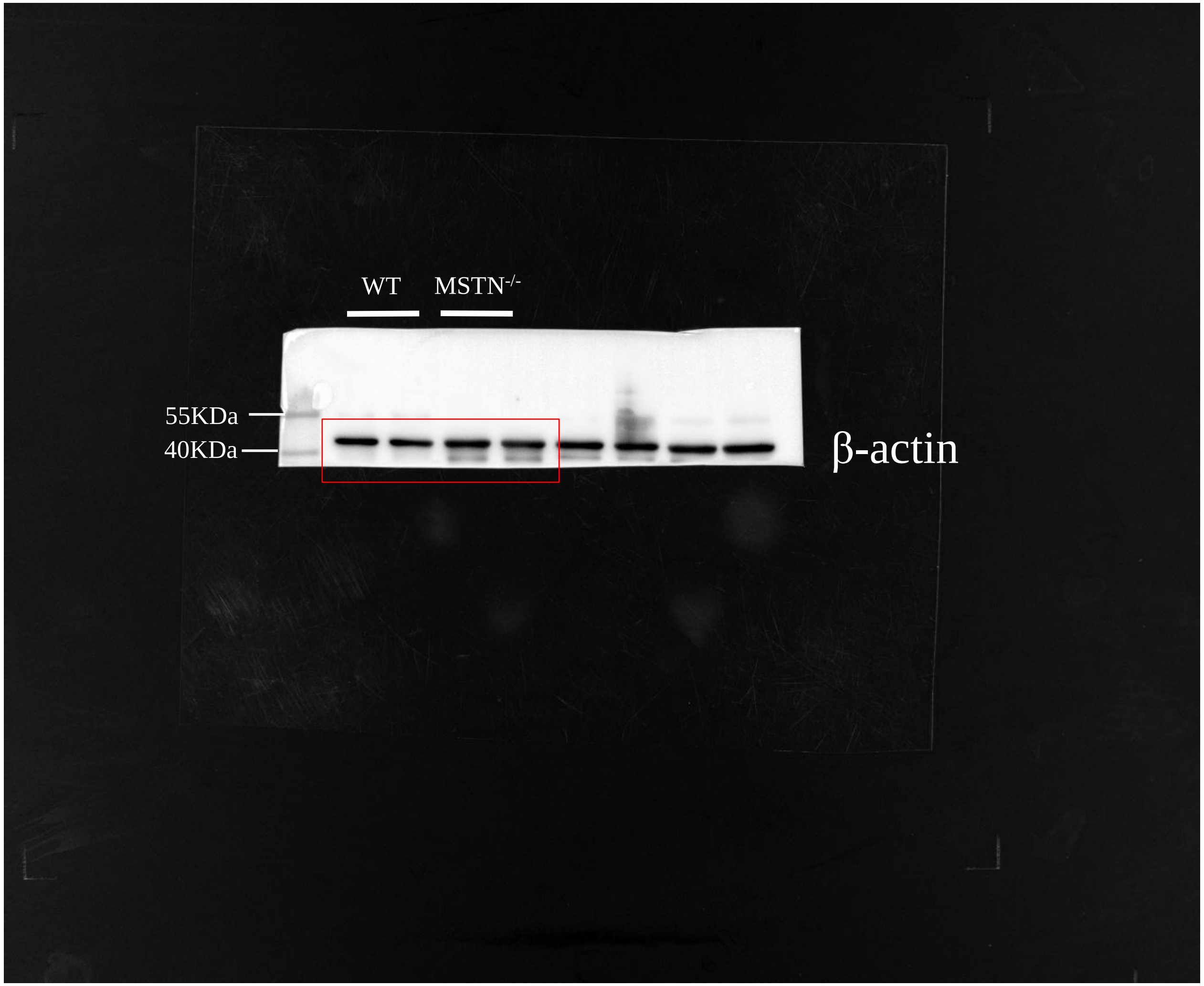

Figure 2D source data

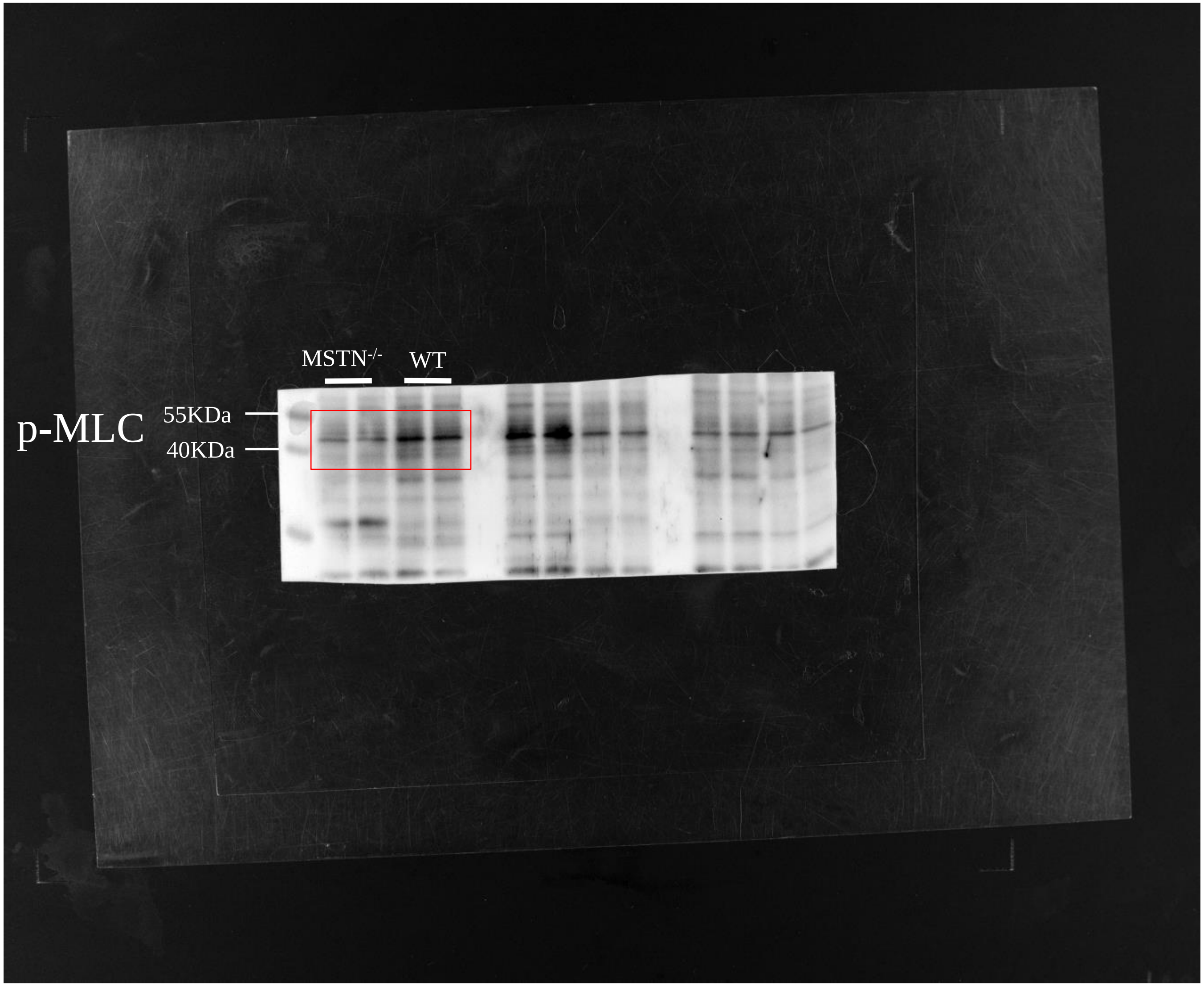

Figure 2D source data

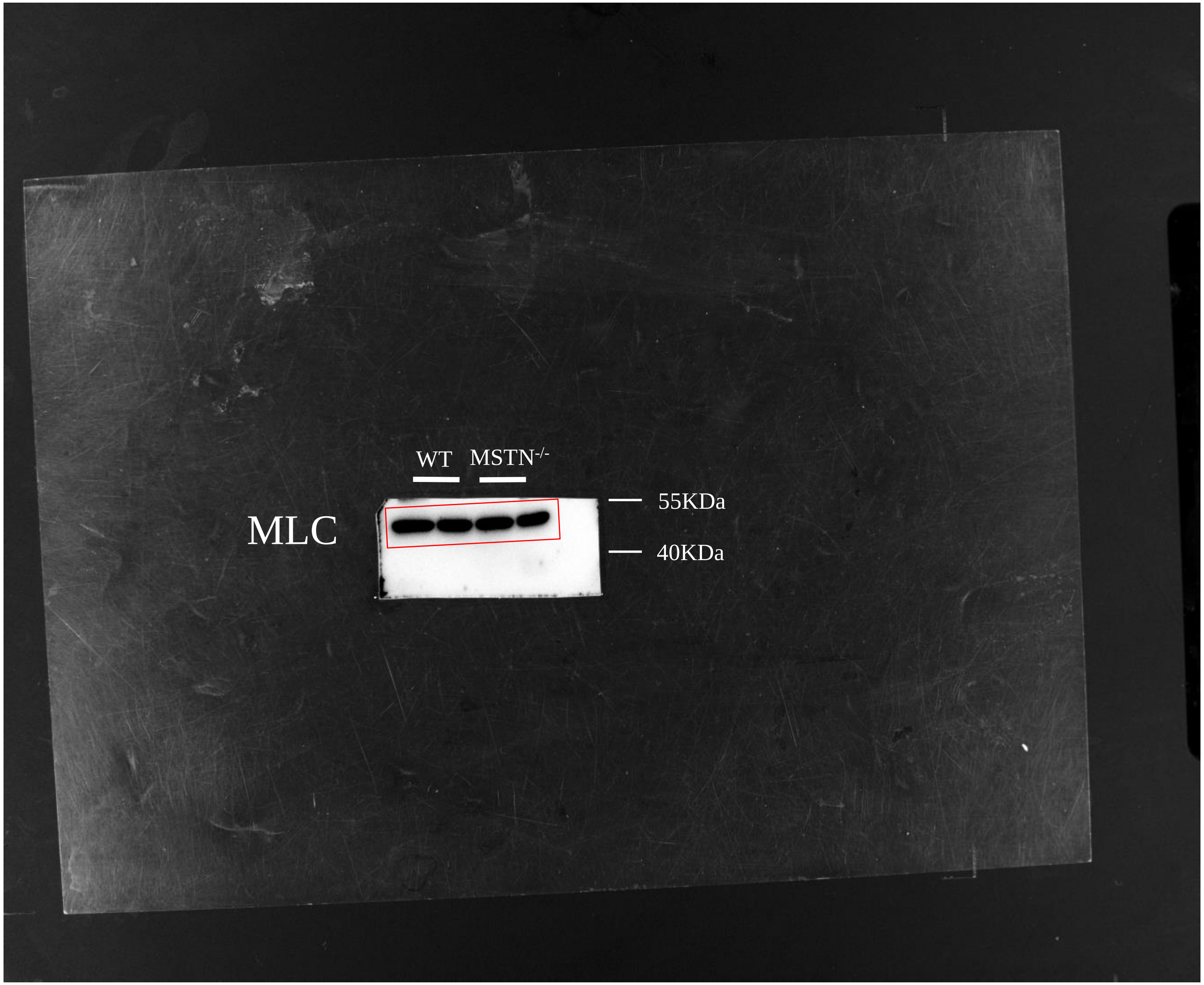

Figure 2D source data

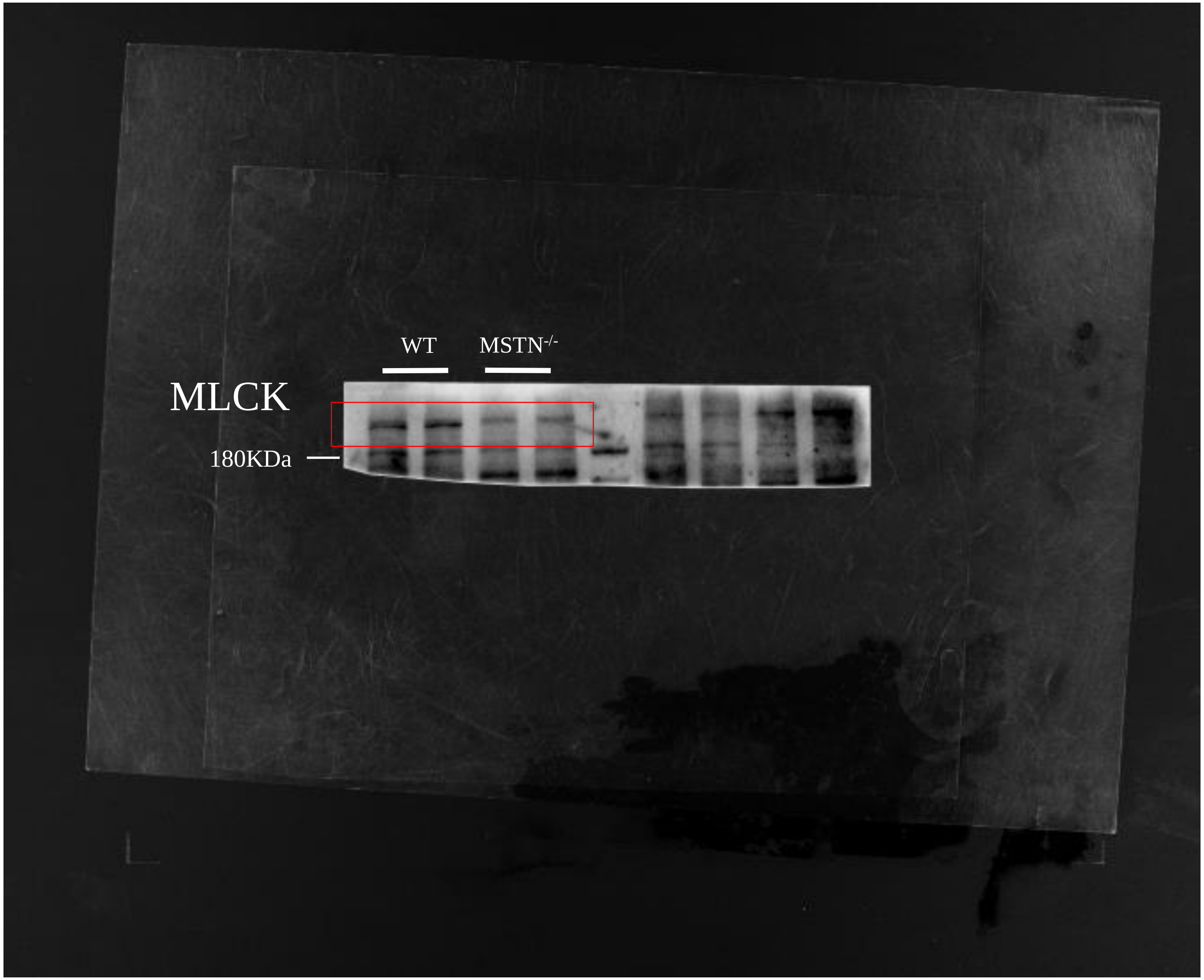

Figure 2D source data

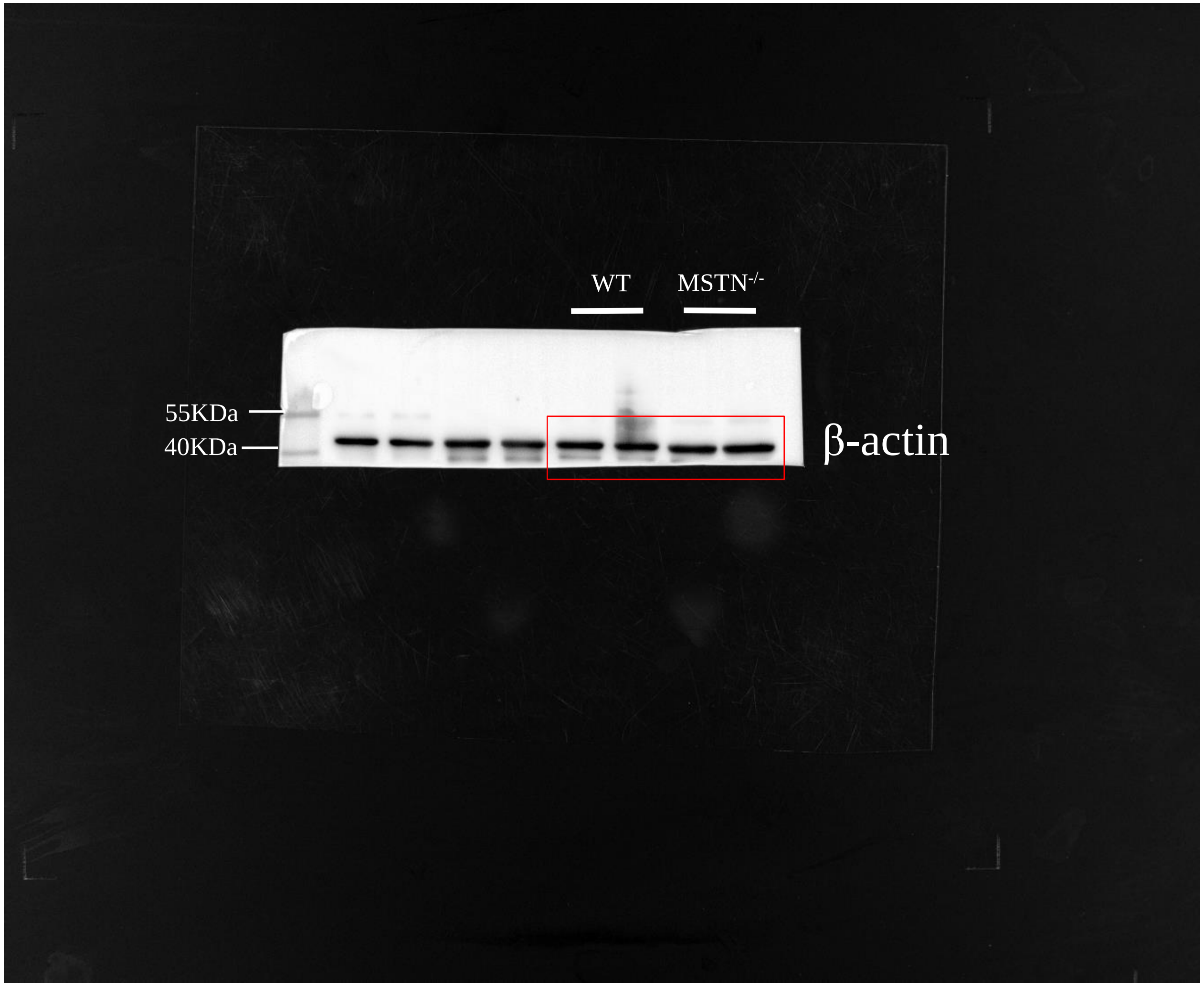

Figure 2E source data

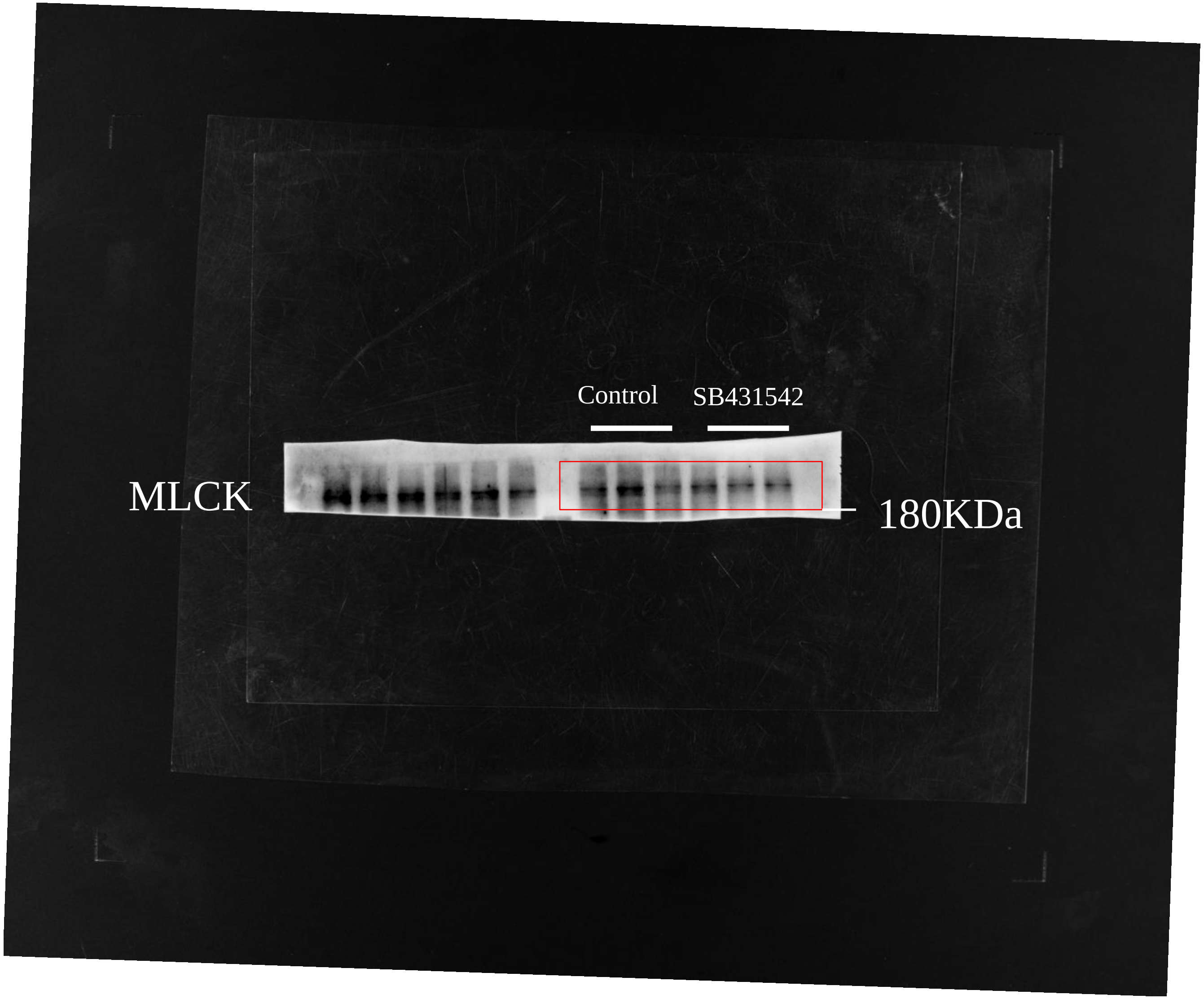

Figure 2E source data

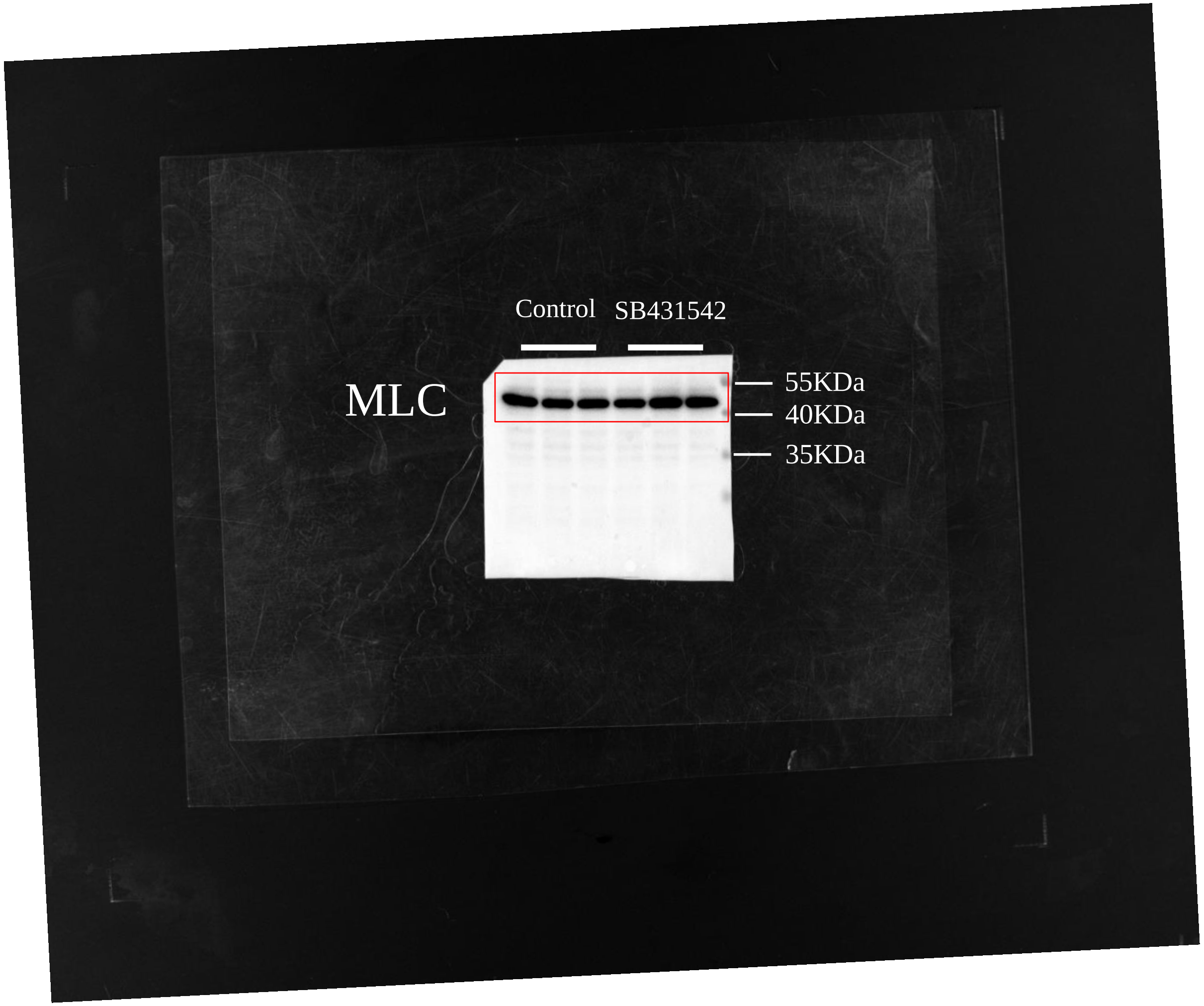

Figure 2E source data

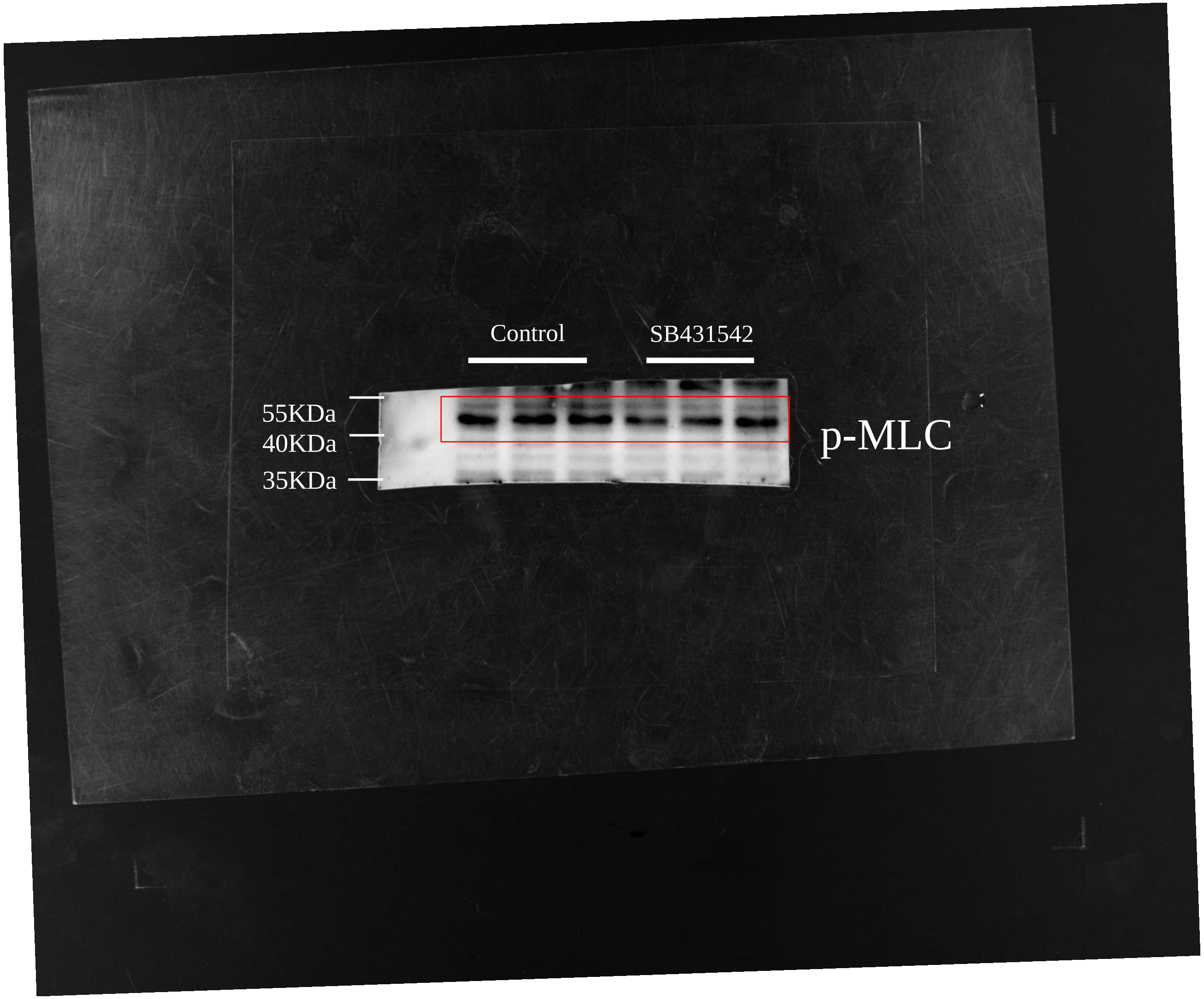

Figure 2E source data

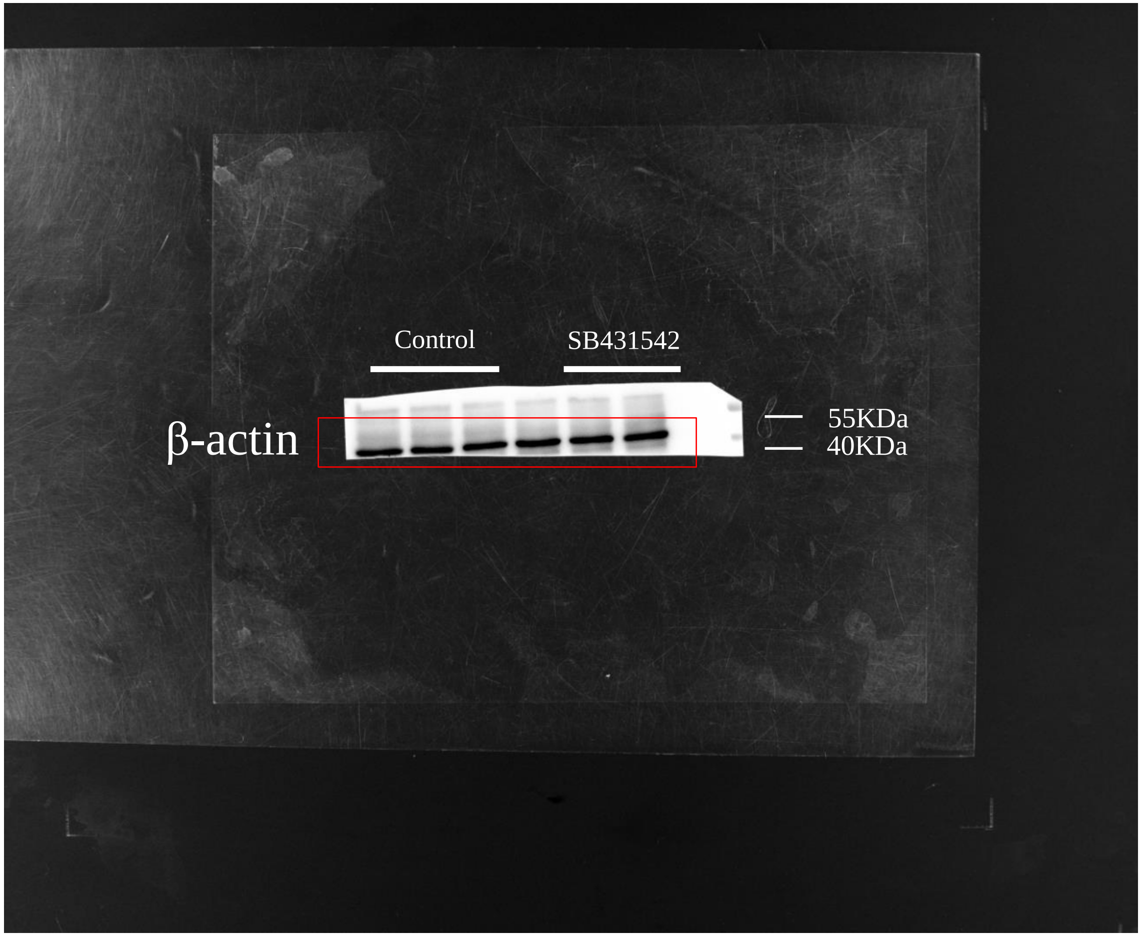

Supplement: Figure 2—source data 2. [file elife-81858-fig2-data2.zip › Figure 2-source data 2/Raw western blot images for Figure 2.pdf]
