# Supplementary material for: Fecal transplant from myostatin deletion pigs positively impacts the gut-muscle axis
Source: eLife. 2023 Apr 11;12:e81858. doi: 10.7554/eLife.81858 (PMC10121221; doi:10.7554/eLife.81858)

Figure 3F source data

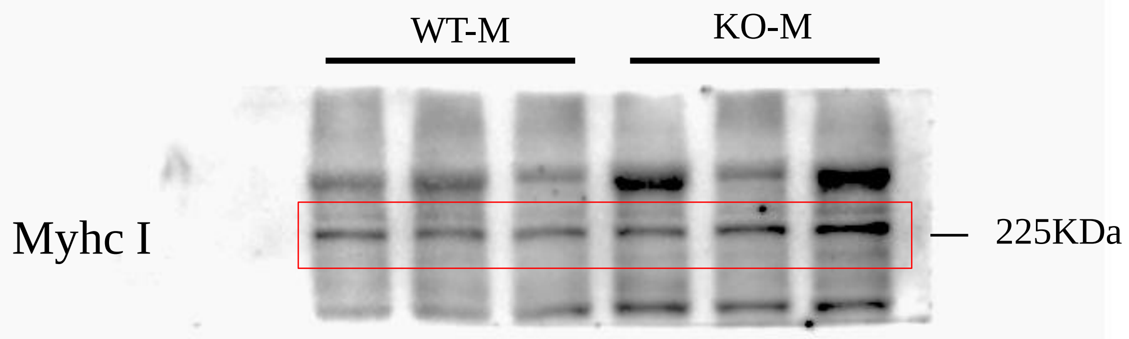

Figure 3F source data

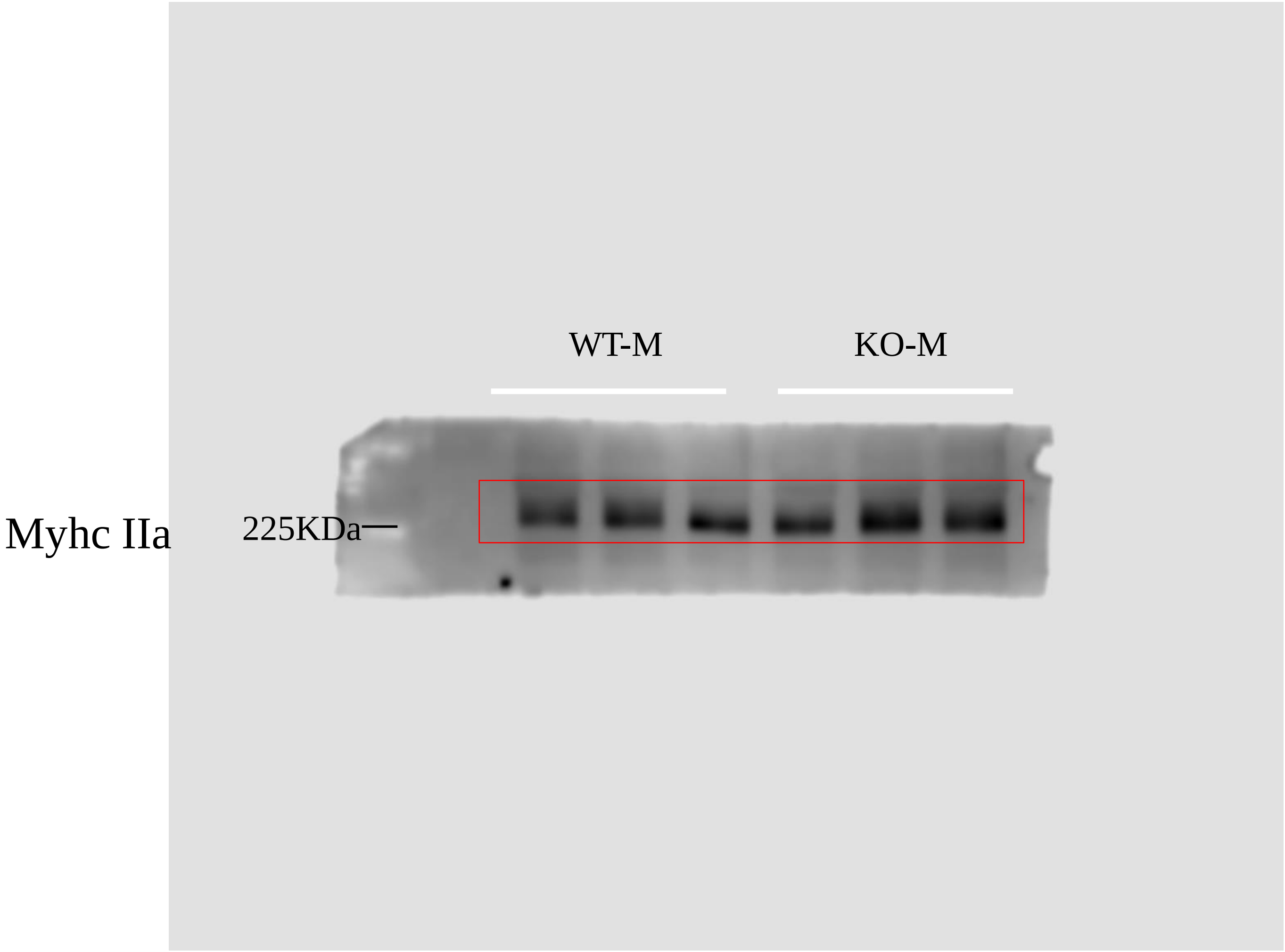

Figure 3F source data

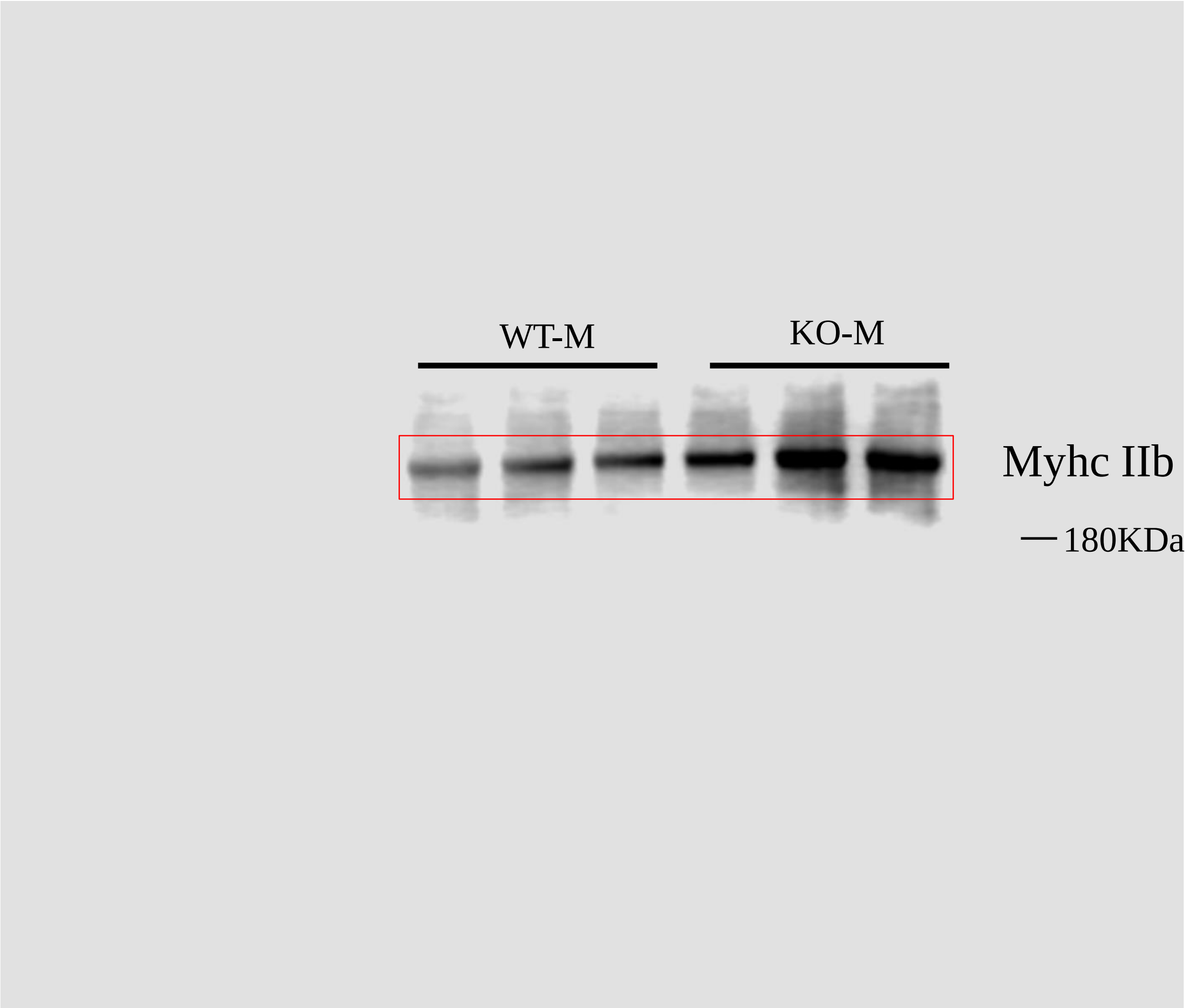

Figure 3F source data

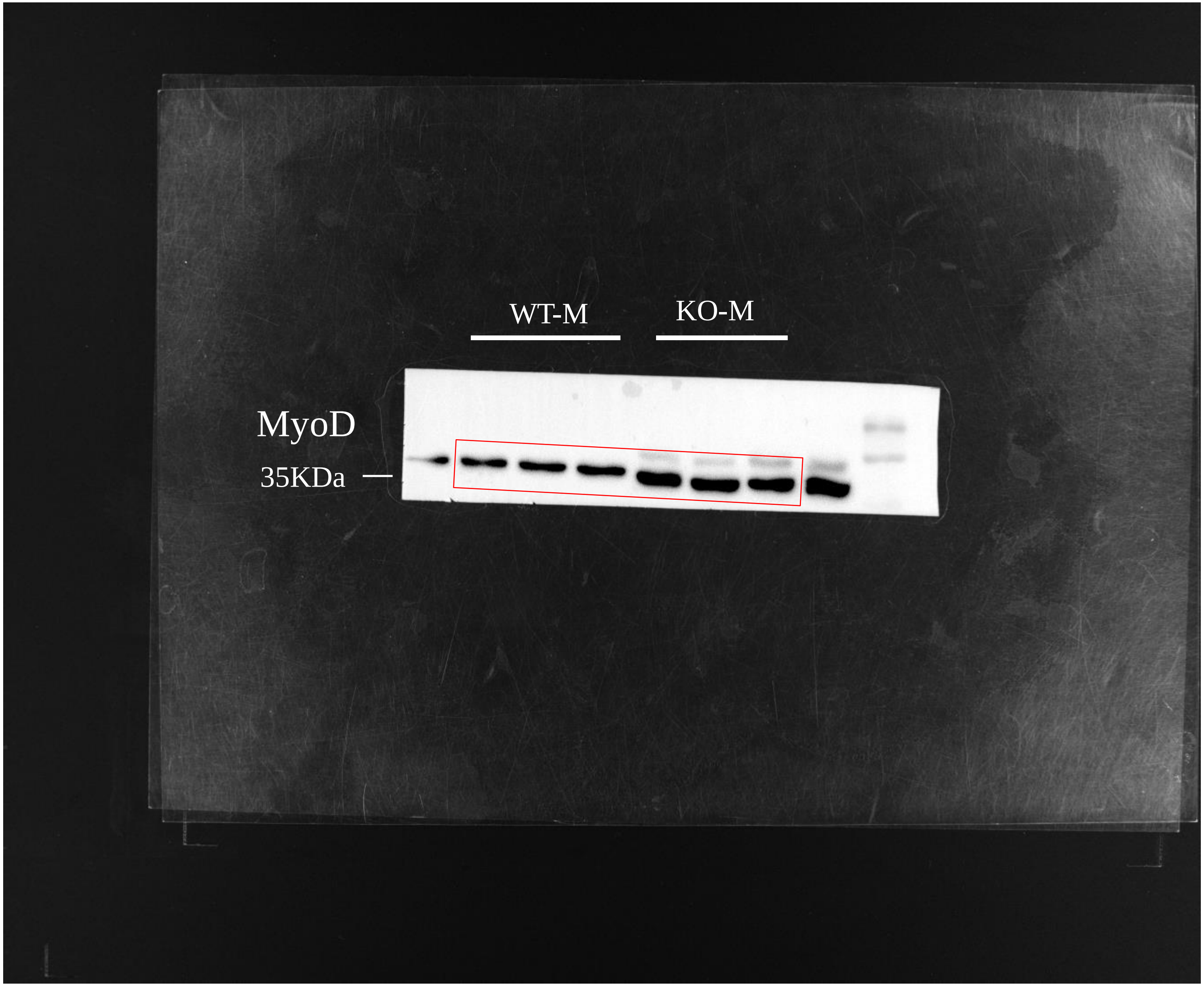

Figure 3F source data

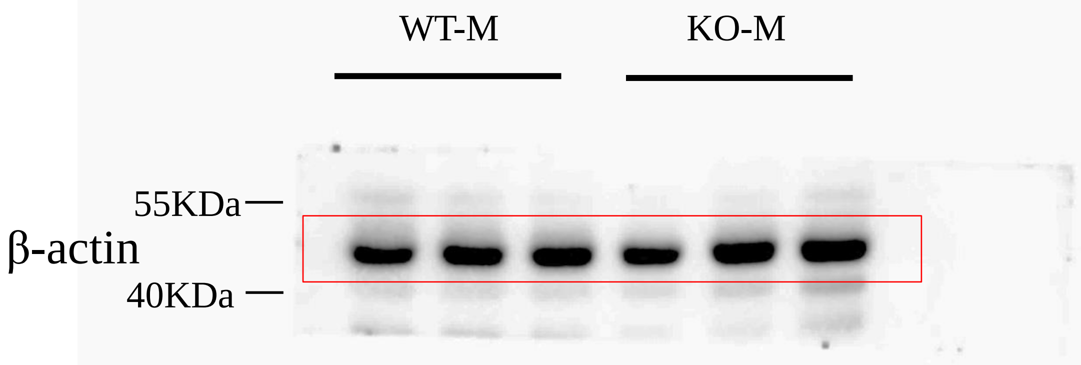

Figure 3G source data

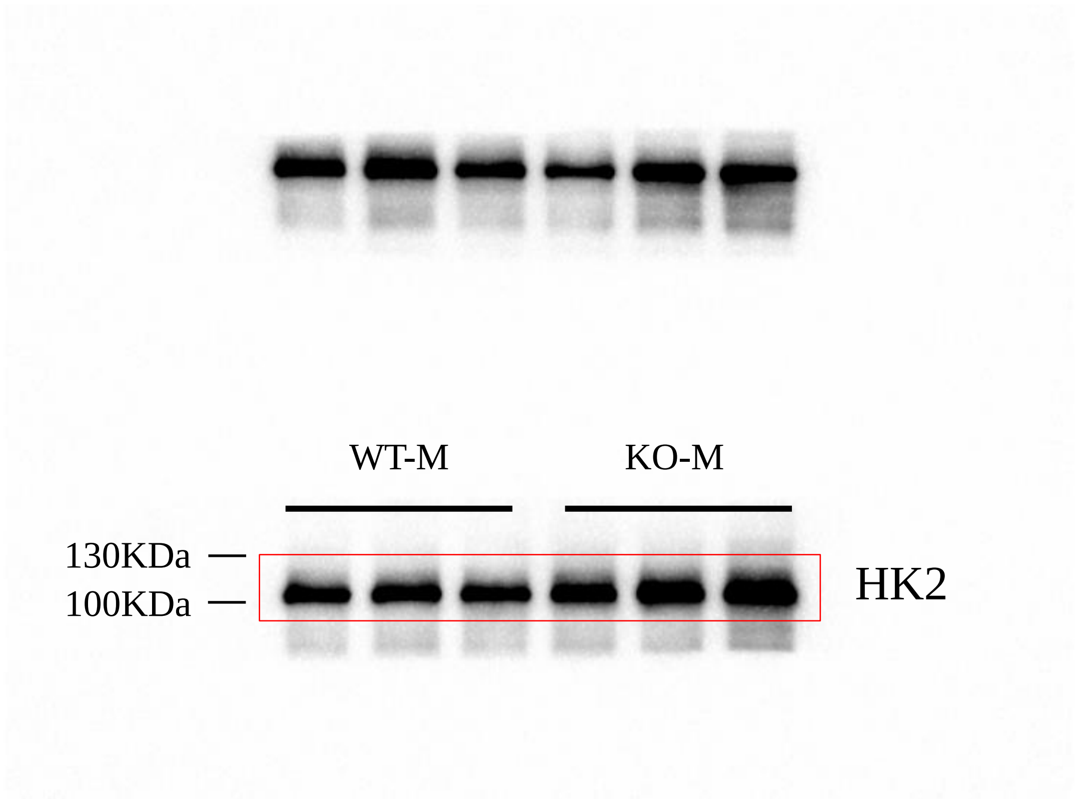

Figure 3G source data

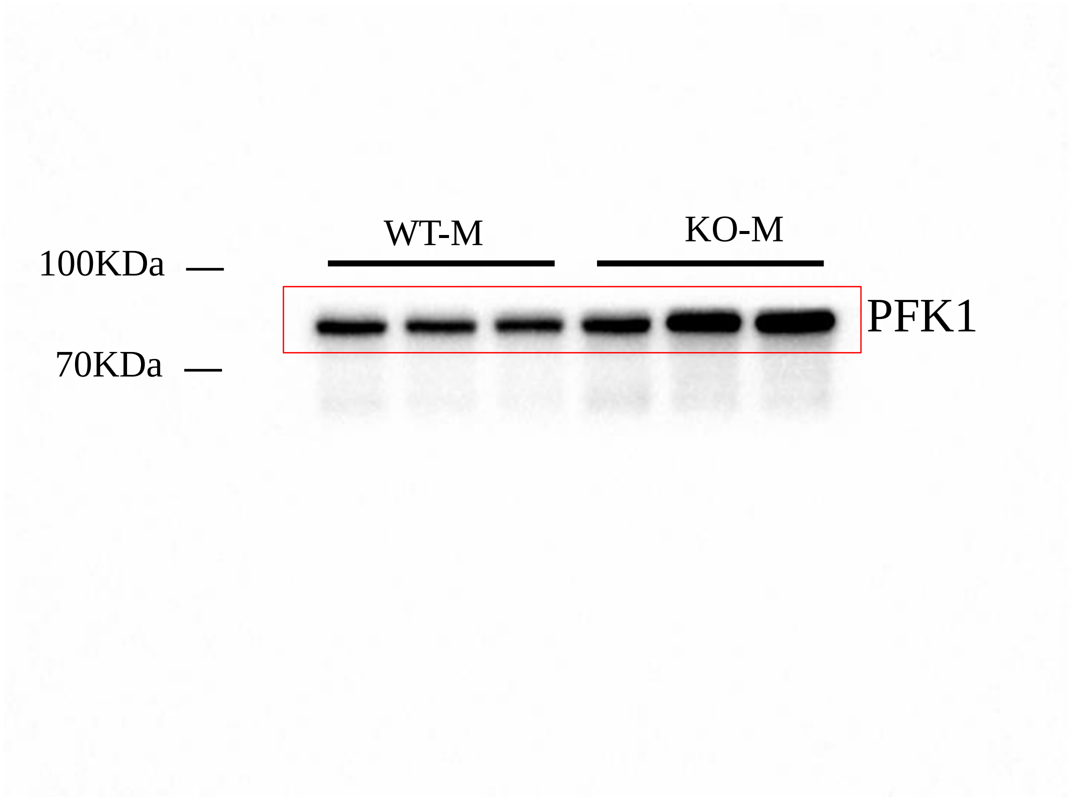

Figure 3G source data

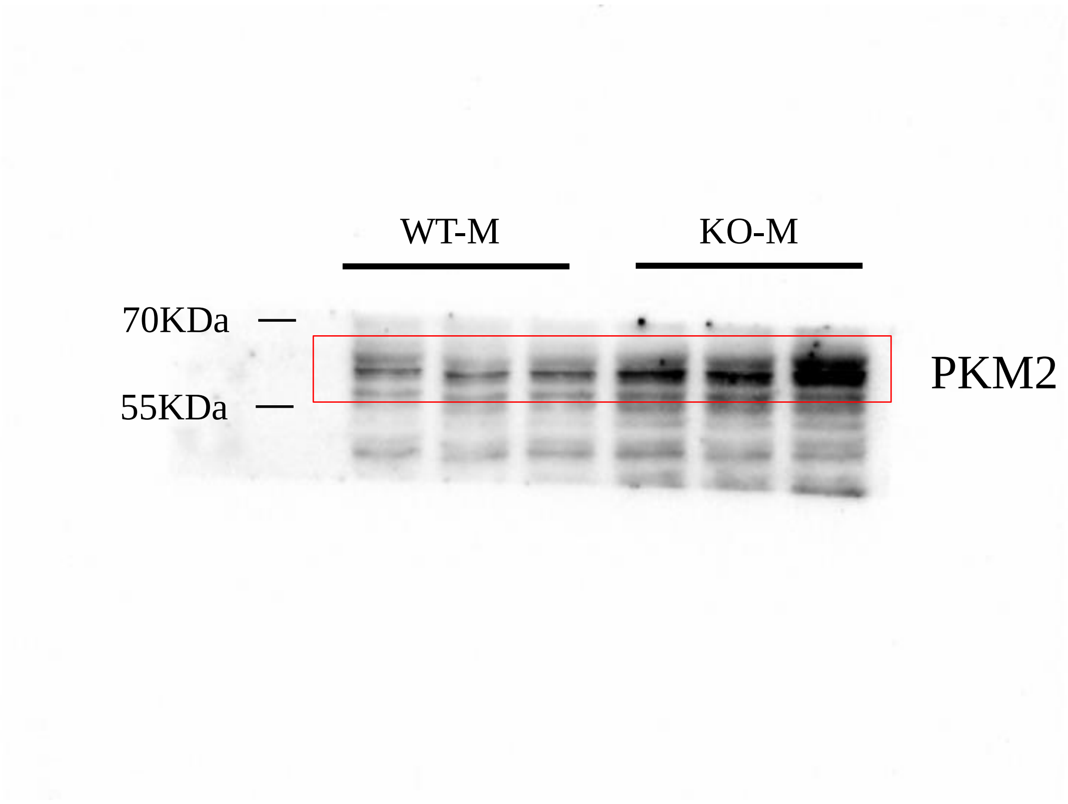

Figure 3G source data

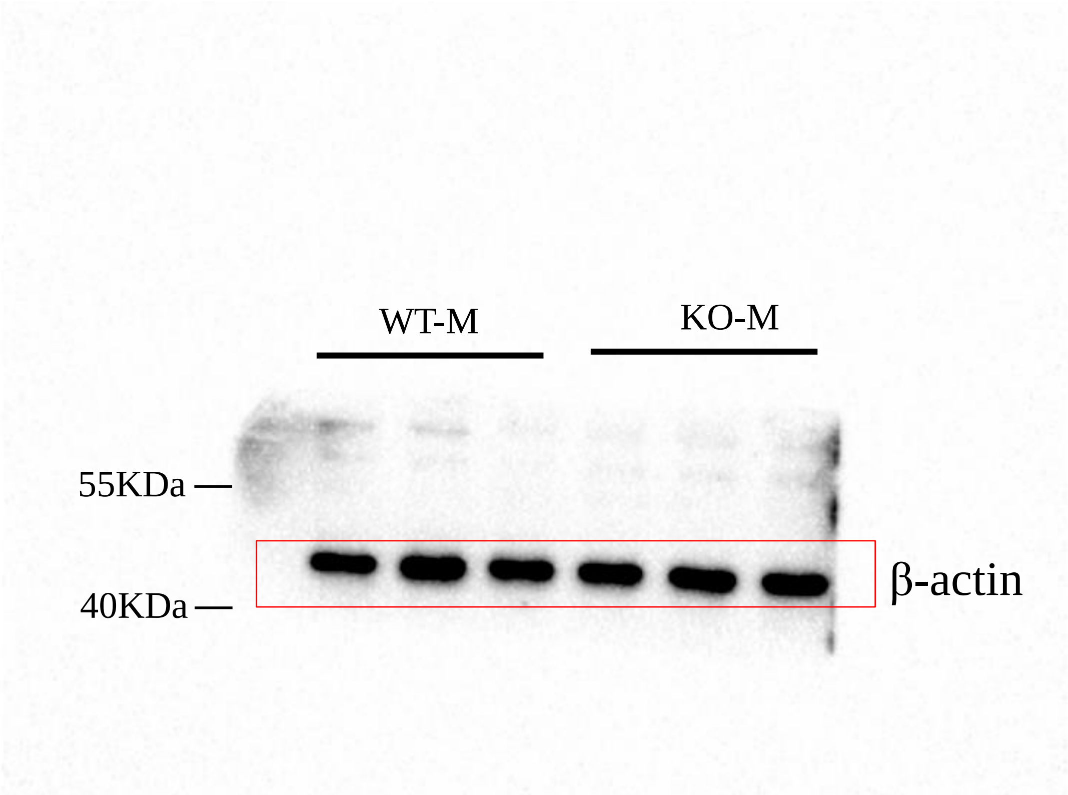

Figure 3H source data

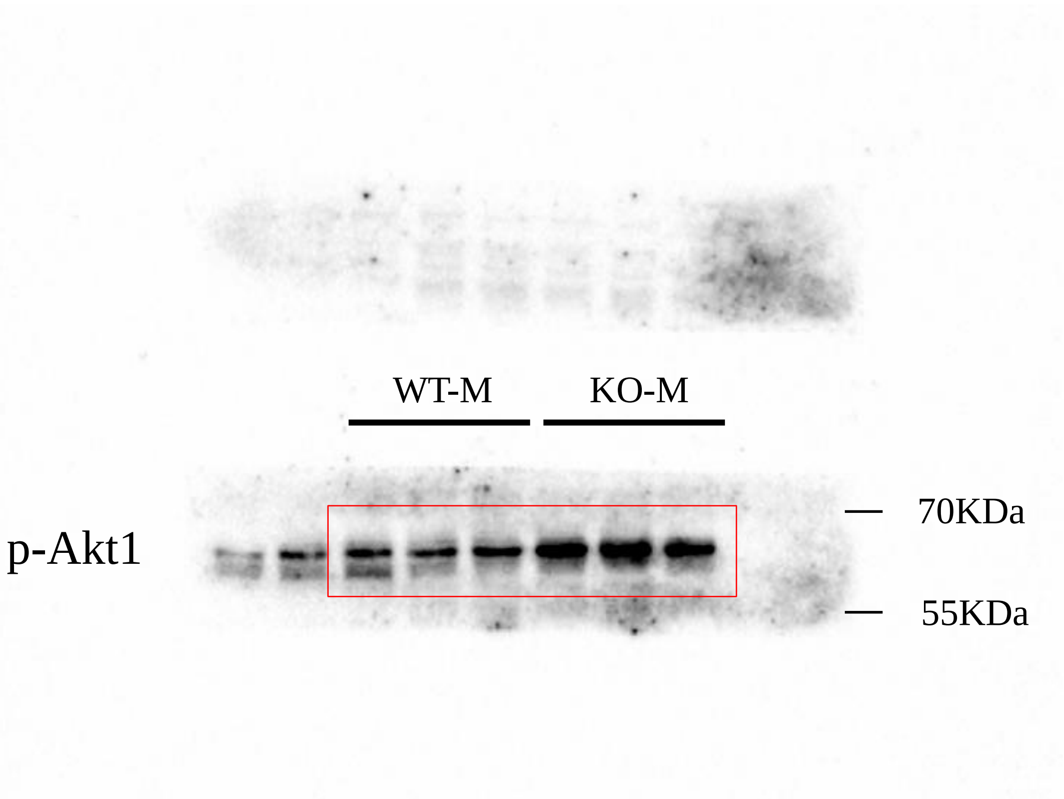

Figure 3H source data

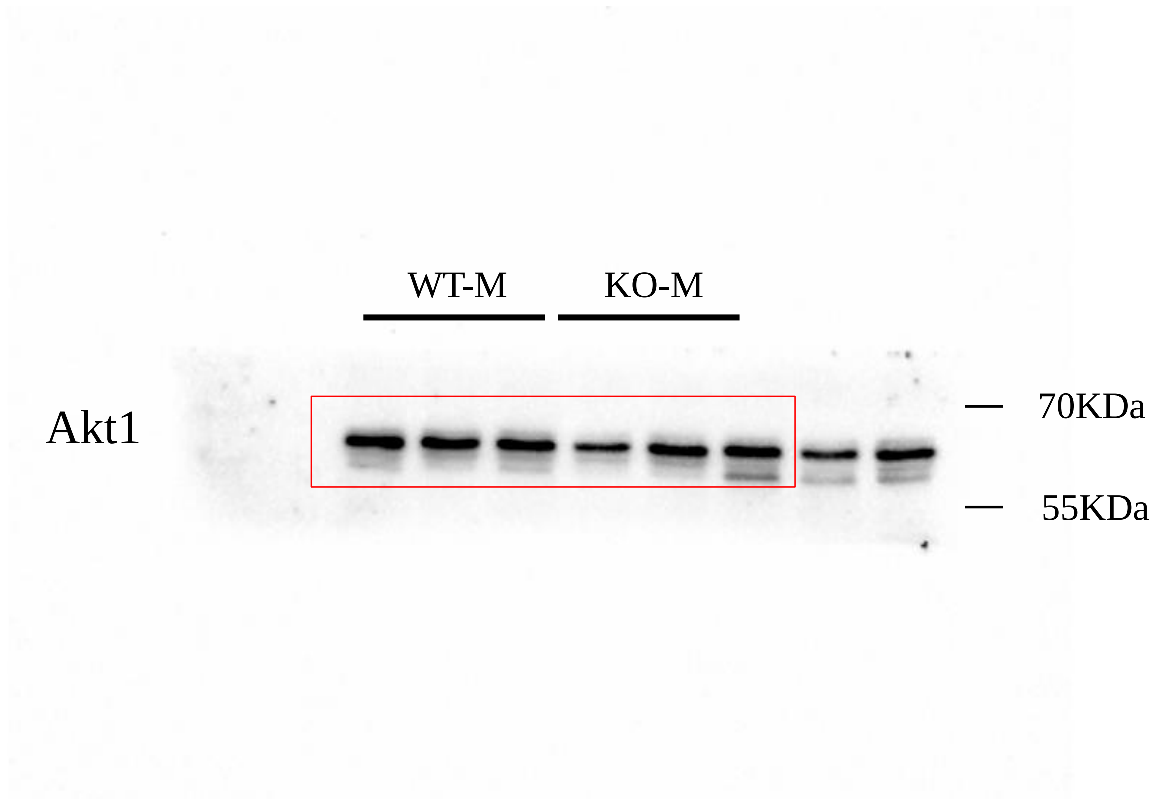

Figure 3H source data

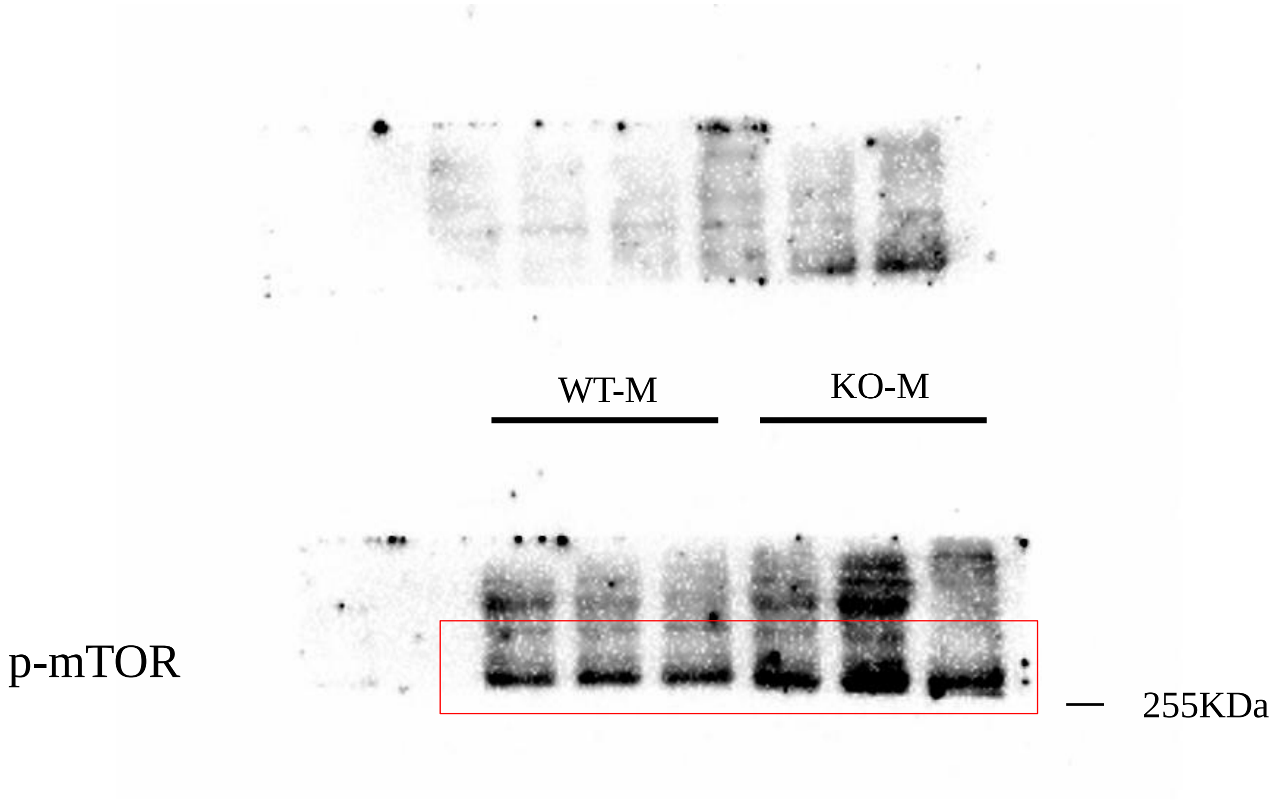

Figure 3H source data

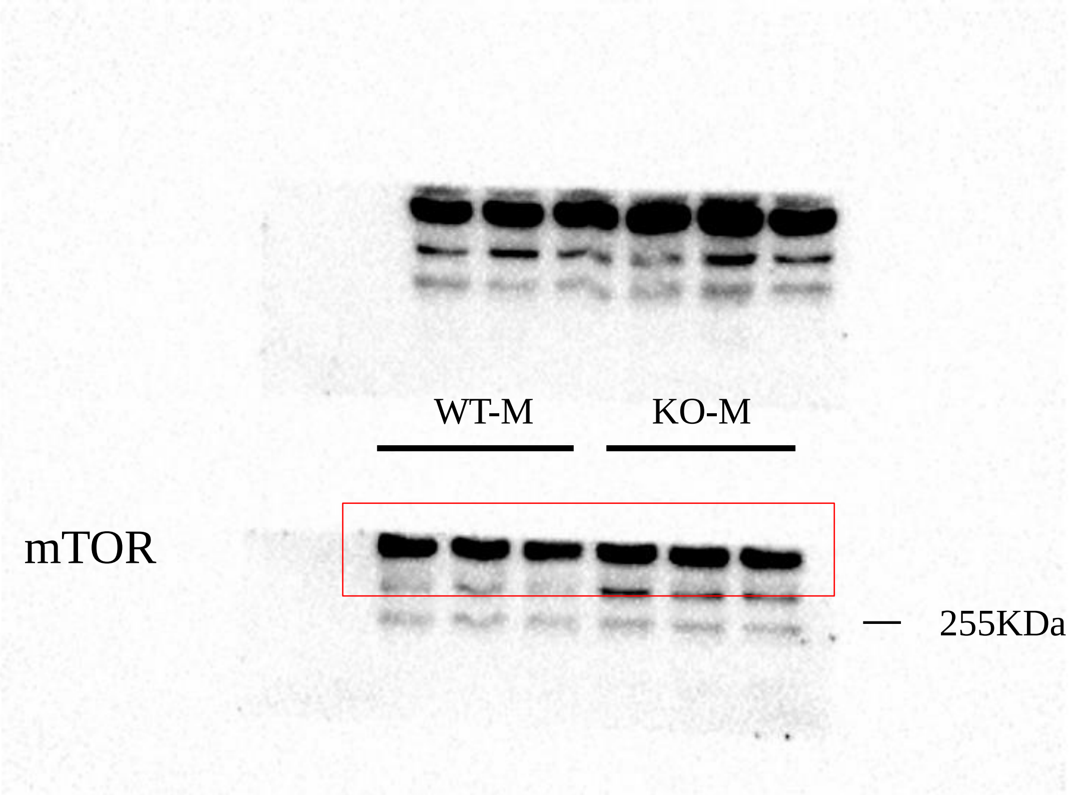

Figure 3H source data

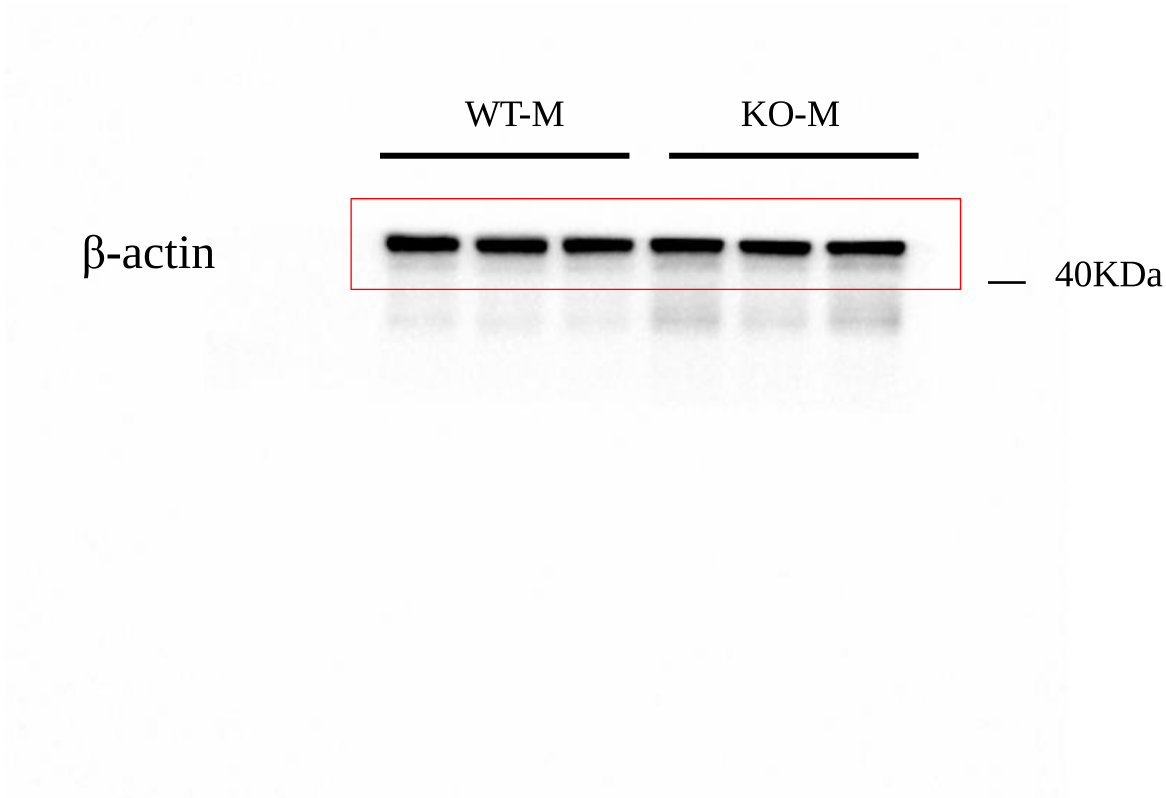

Supplement: Figure 3—source data 2. [file elife-81858-fig3-data2.zip › Figure 3-source data 2/Raw western blot images for Figure 3.pdf]
