# Supplementary figures and images for: Fecal transplant from myostatin deletion pigs positively impacts the gut-muscle axis (part 2 of 2)
Source: eLife. 2023 Apr 11;12:e81858. doi: 10.7554/eLife.81858 (PMC10121221; doi:10.7554/eLife.81858)

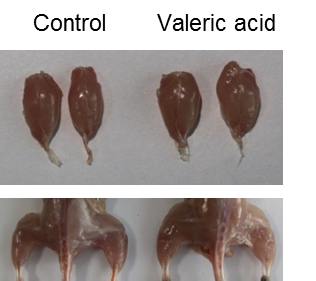

Supplement: Figure 7—source data 1. [file elife-81858-fig7-data1.zip › Figure 7-source data 1/fig7.a/fig7.a.tif]

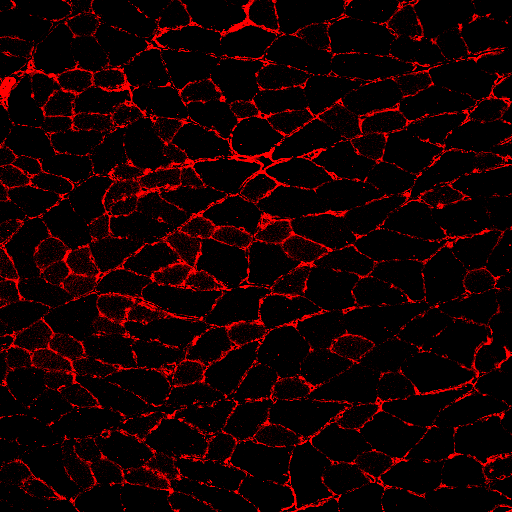

Supplement: Figure 7—source data 1. [file elife-81858-fig7-data1.zip › Figure 7-source data 1/fig7.c/SCFA-laimin/Control/Con1.tif]

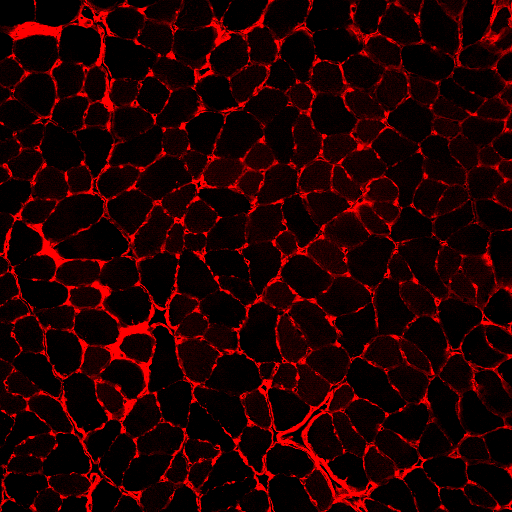

Supplement: Figure 7—source data 1. [file elife-81858-fig7-data1.zip › Figure 7-source data 1/fig7.c/SCFA-laimin/Control/Con2.tif]

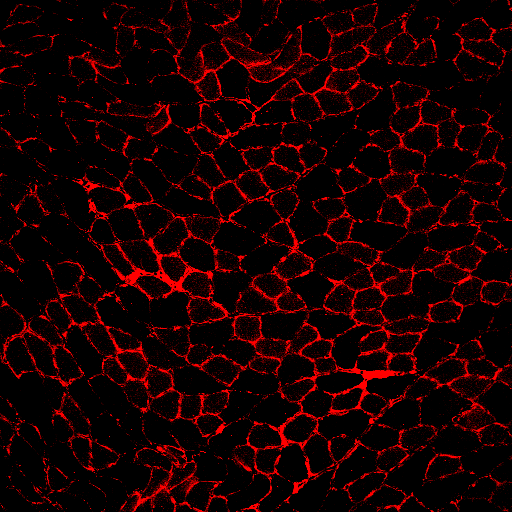

Supplement: Figure 7—source data 1. [file elife-81858-fig7-data1.zip › Figure 7-source data 1/fig7.c/SCFA-laimin/Control/Con3.tif]

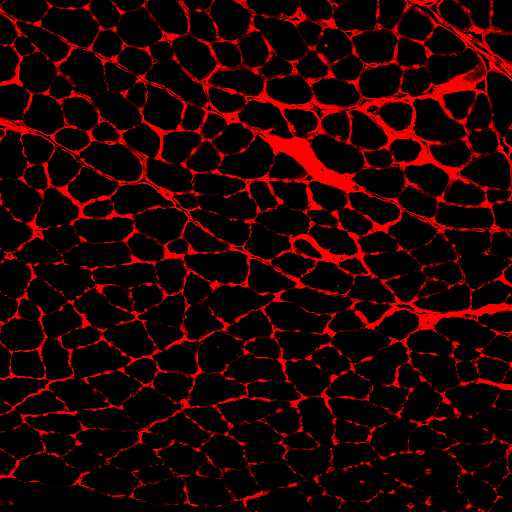

Supplement: Figure 7—source data 1. [file elife-81858-fig7-data1.zip › Figure 7-source data 1/fig7.c/SCFA-laimin/Control/Con4.tif]

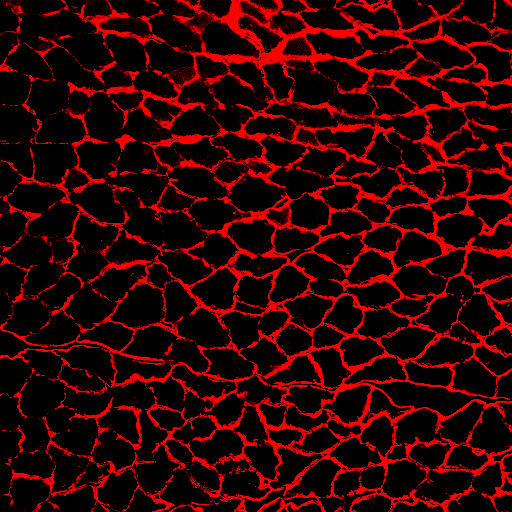

Supplement: Figure 7—source data 1. [file elife-81858-fig7-data1.zip › Figure 7-source data 1/fig7.c/SCFA-laimin/Control/Con5.tif]

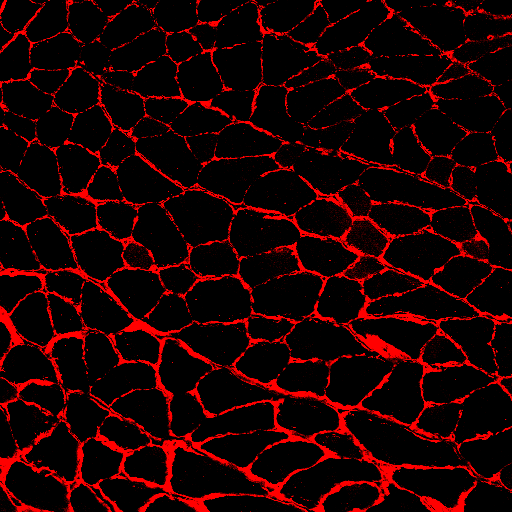

Supplement: Figure 7—source data 1. [file elife-81858-fig7-data1.zip › Figure 7-source data 1/fig7.c/SCFA-laimin/Val/Val1.tif]

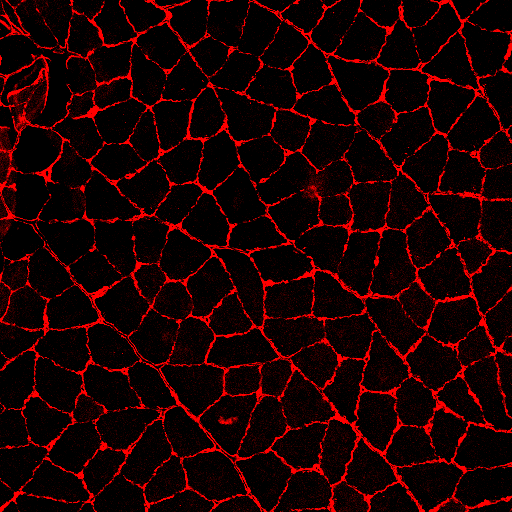

Supplement: Figure 7—source data 1. [file elife-81858-fig7-data1.zip › Figure 7-source data 1/fig7.c/SCFA-laimin/Val/Val2.tif]

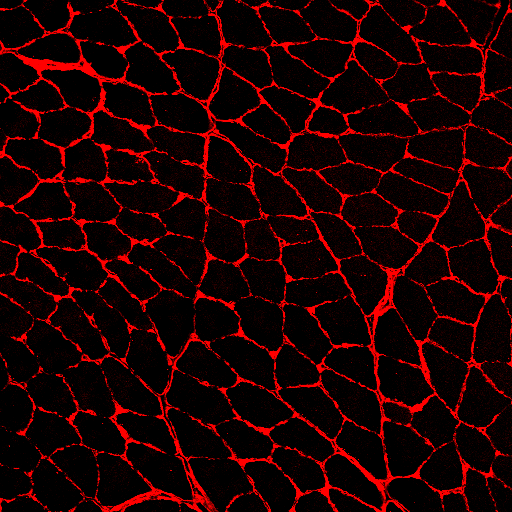

Supplement: Figure 7—source data 1. [file elife-81858-fig7-data1.zip › Figure 7-source data 1/fig7.c/SCFA-laimin/Val/Val3.tif]

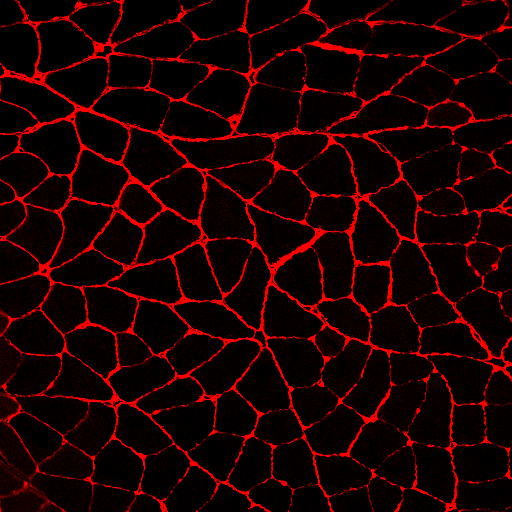

Supplement: Figure 7—source data 1. [file elife-81858-fig7-data1.zip › Figure 7-source data 1/fig7.c/SCFA-laimin/Val/Val4.tif]

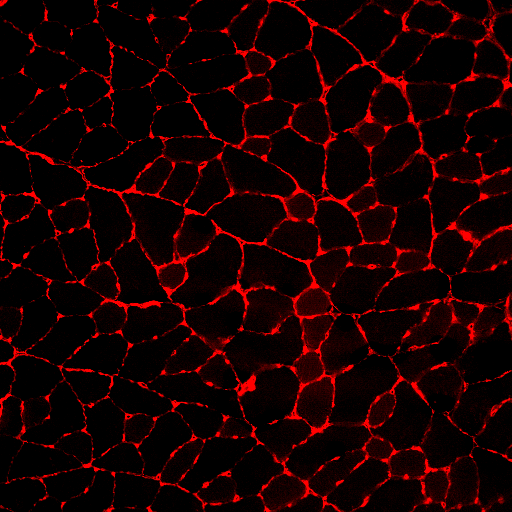

Supplement: Figure 7—source data 1. [file elife-81858-fig7-data1.zip › Figure 7-source data 1/fig7.c/SCFA-laimin/Val/Val5.tif]

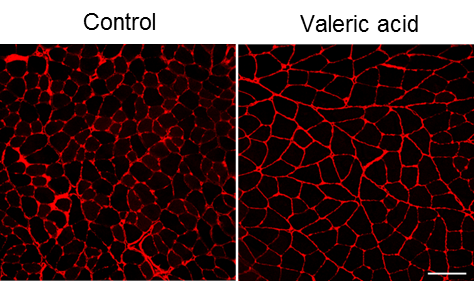

Supplement: Figure 7—source data 1. [file elife-81858-fig7-data1.zip › Figure 7-source data 1/fig7.c/fig7.c.tif]

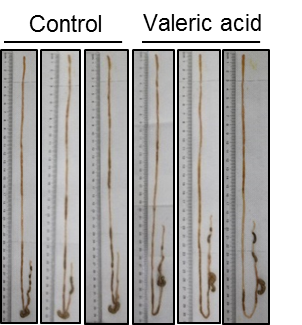

Supplement: Figure 7—source data 1. [file elife-81858-fig7-data1.zip › Figure 7-source data 1/fig7.h/fig7.h.tif]

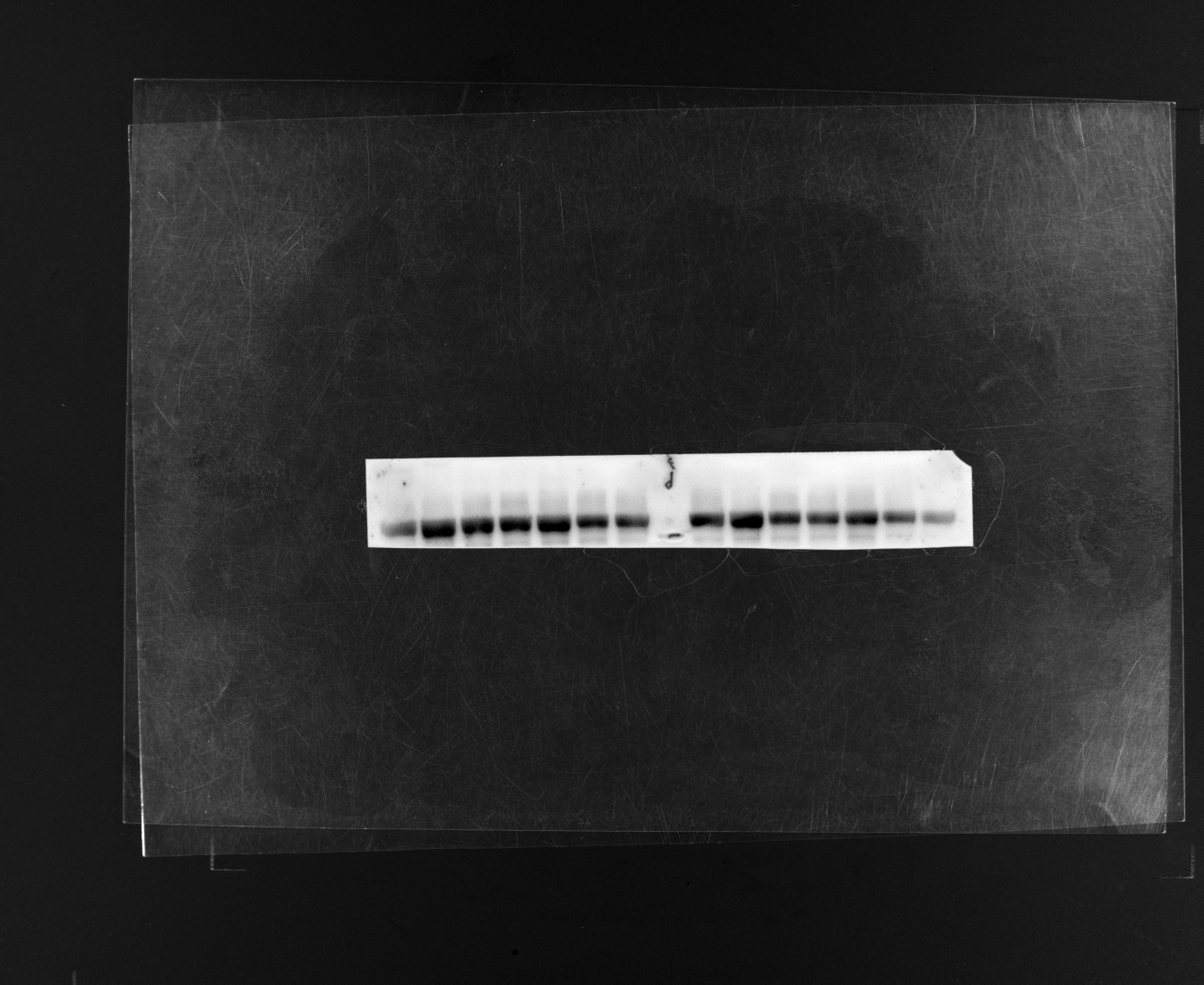

Supplement: Figure 7—source data 2. [file elife-81858-fig7-data2.zip › Figure 7-source data 2/fig7d.MyhcI.tif]

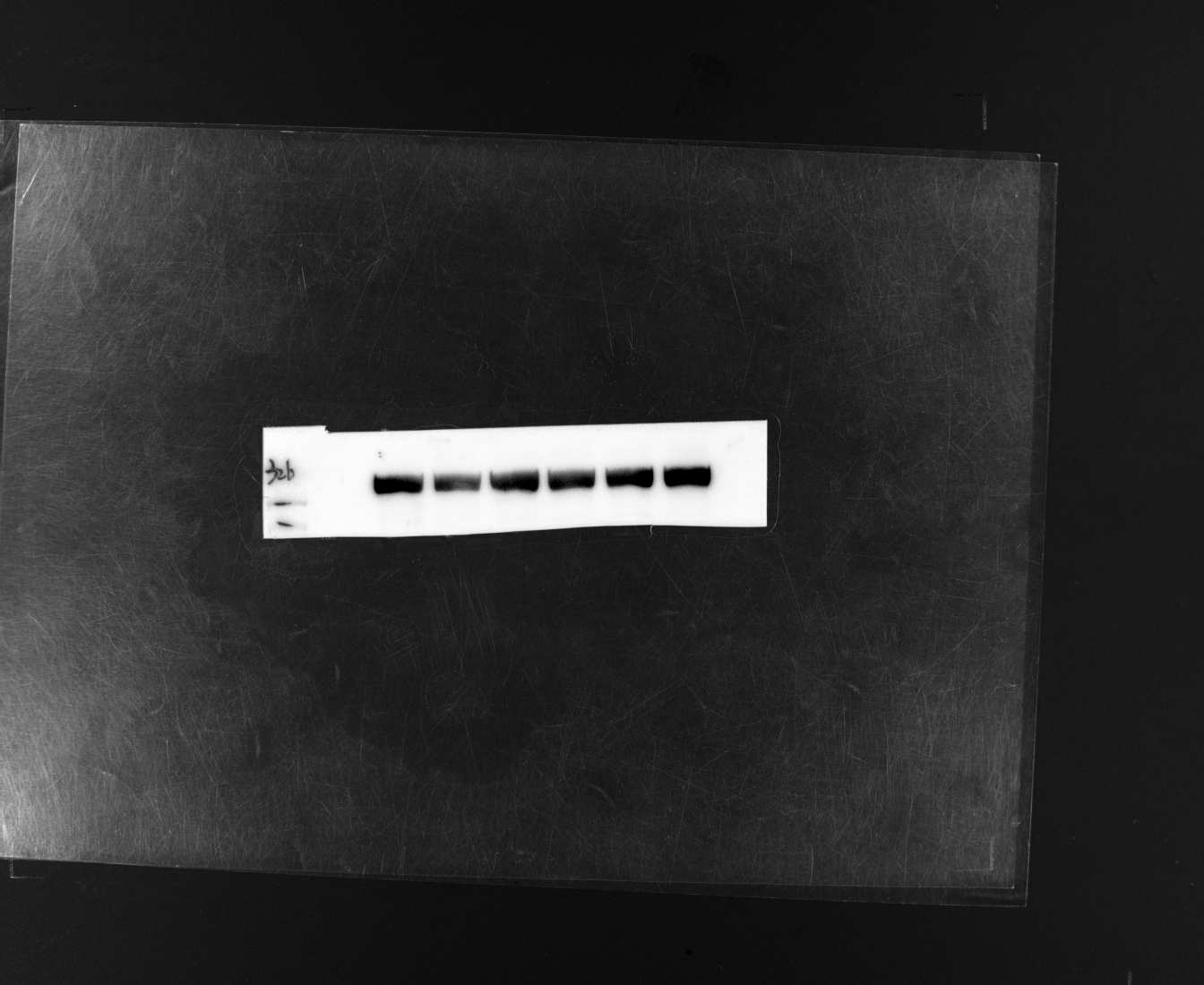

Supplement: Figure 7—source data 2. [file elife-81858-fig7-data2.zip › Figure 7-source data 2/fig7d.MyhcIIa.tif]

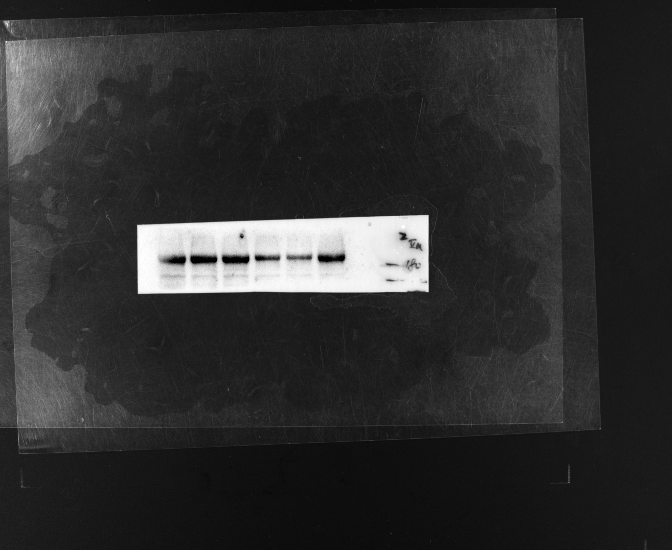

Supplement: Figure 7—source data 2. [file elife-81858-fig7-data2.zip › Figure 7-source data 2/fig7d.MyhcIIb.tif]

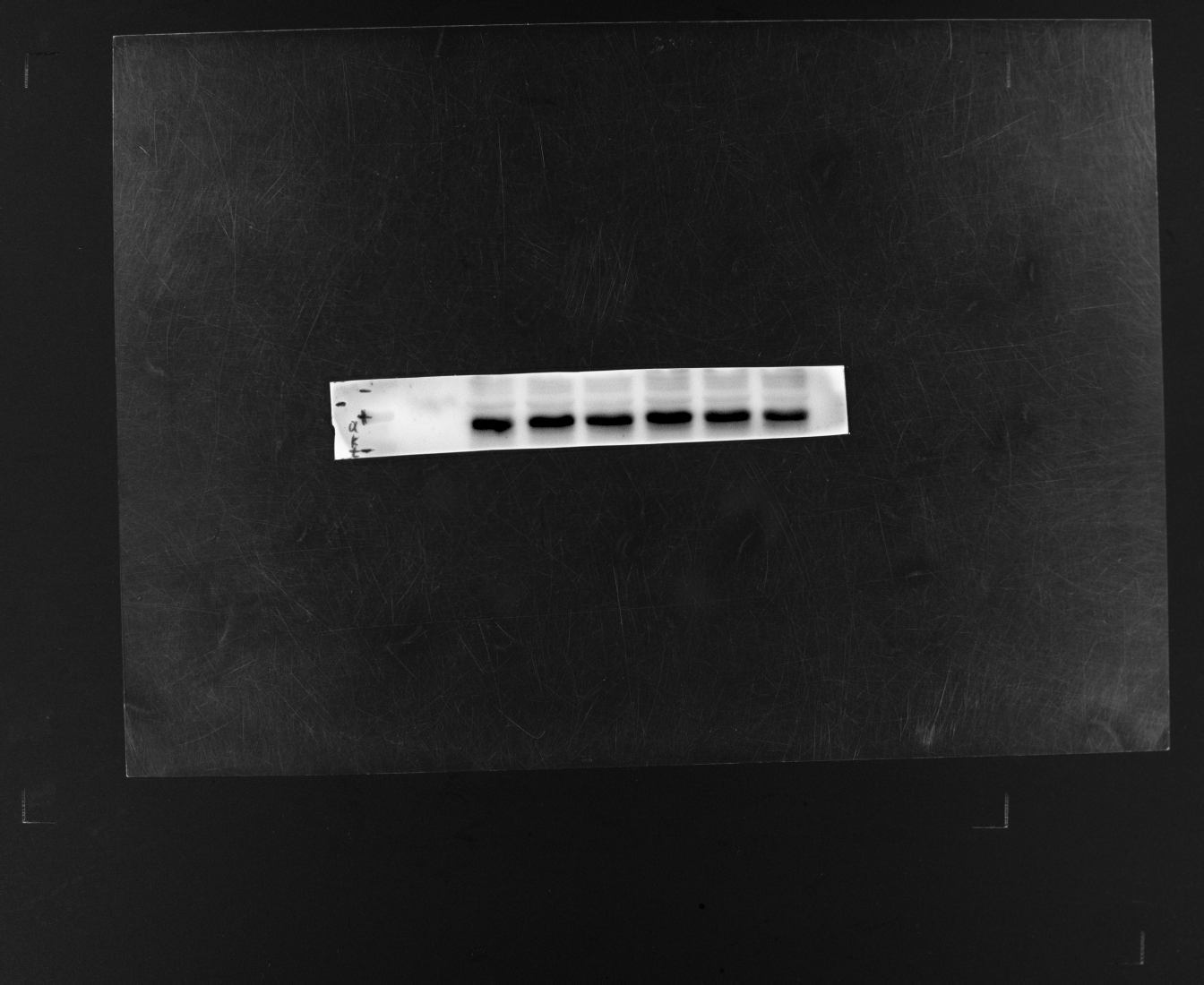

Supplement: Figure 7—source data 2. [file elife-81858-fig7-data2.zip › Figure 7-source data 2/fig7d.actin.tif]

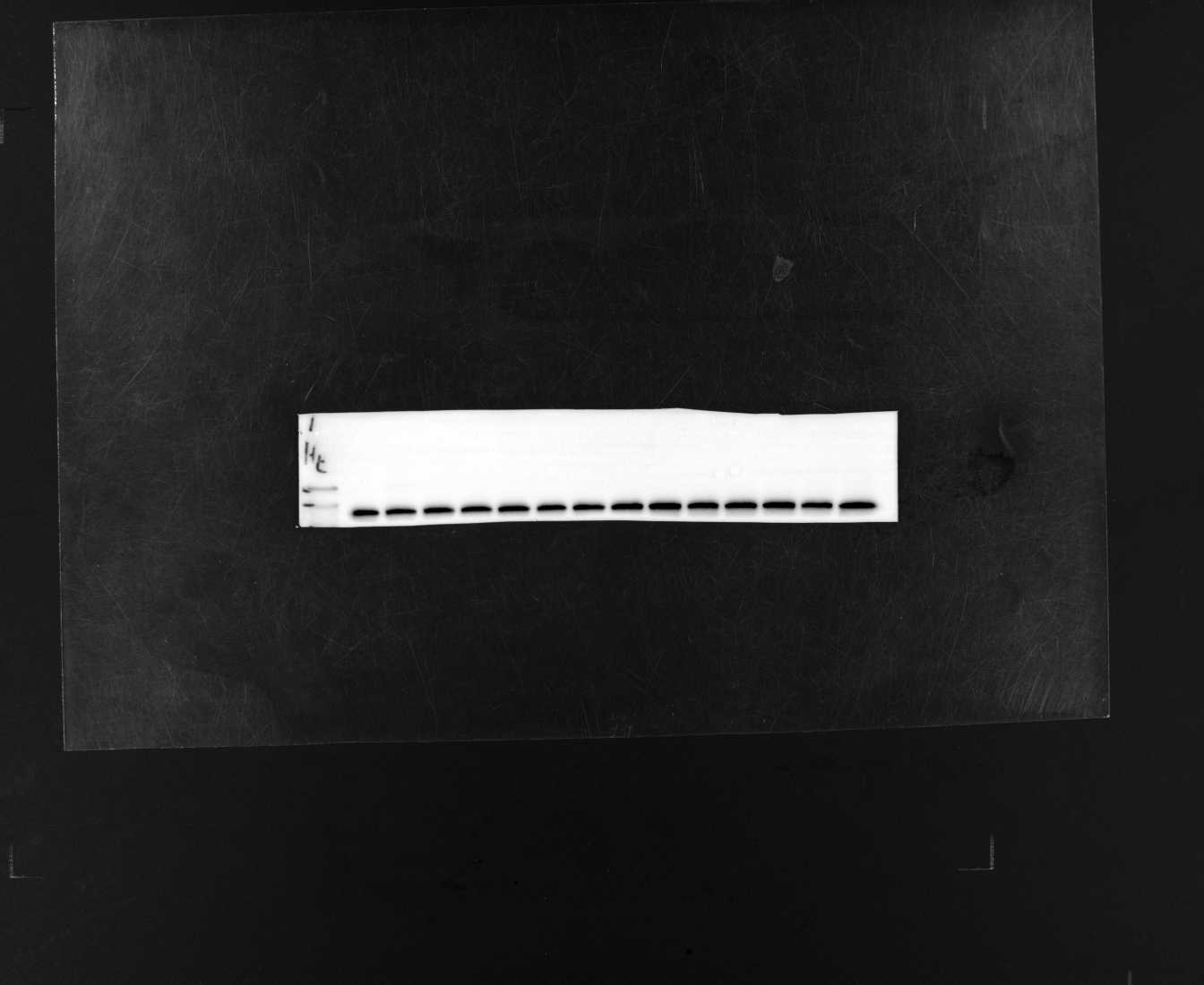

Supplement: Figure 7—source data 2. [file elife-81858-fig7-data2.zip › Figure 7-source data 2/fig7e.HK2.tif]

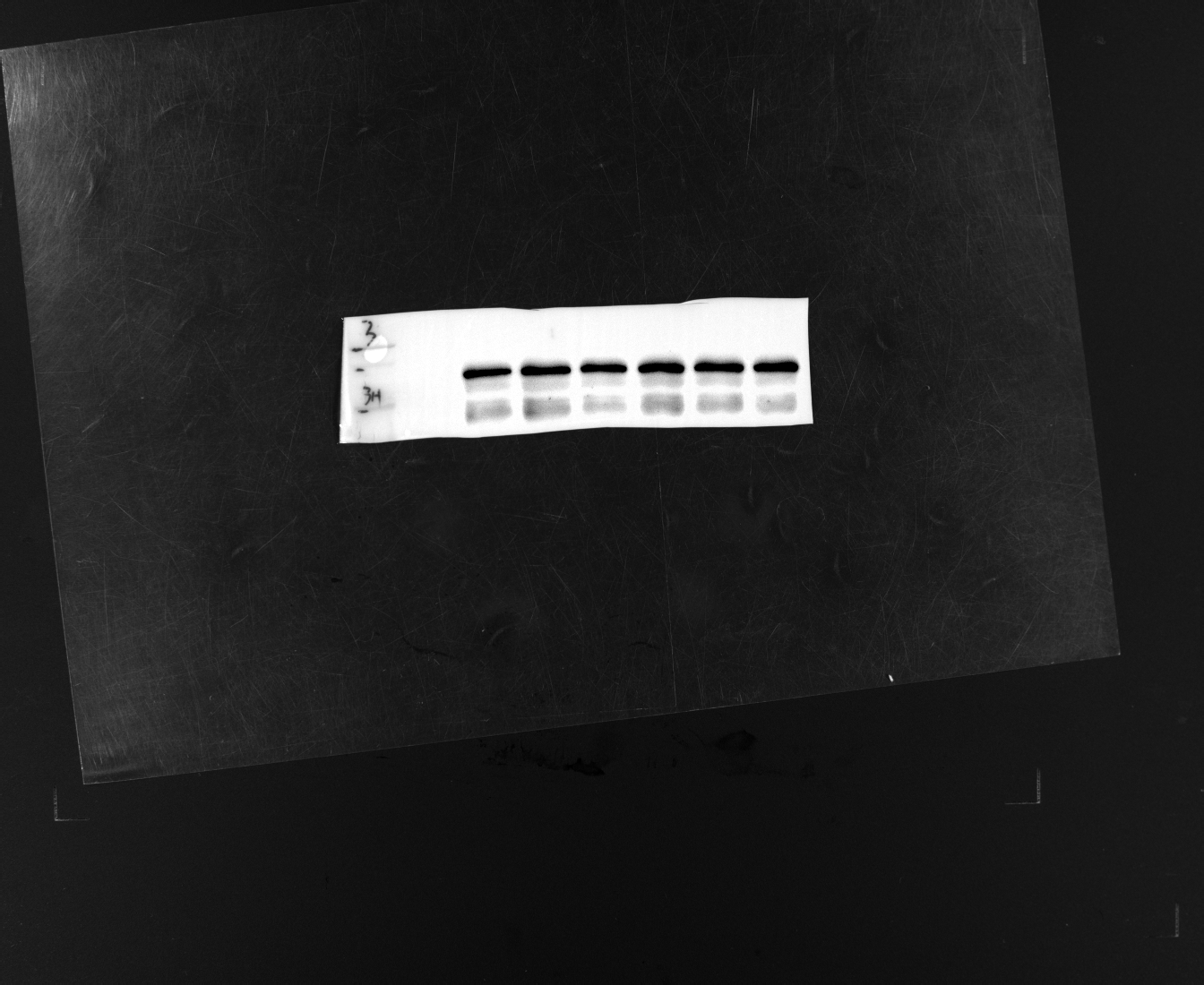

Supplement: Figure 7—source data 2. [file elife-81858-fig7-data2.zip › Figure 7-source data 2/fig7e.PFK1.tif]

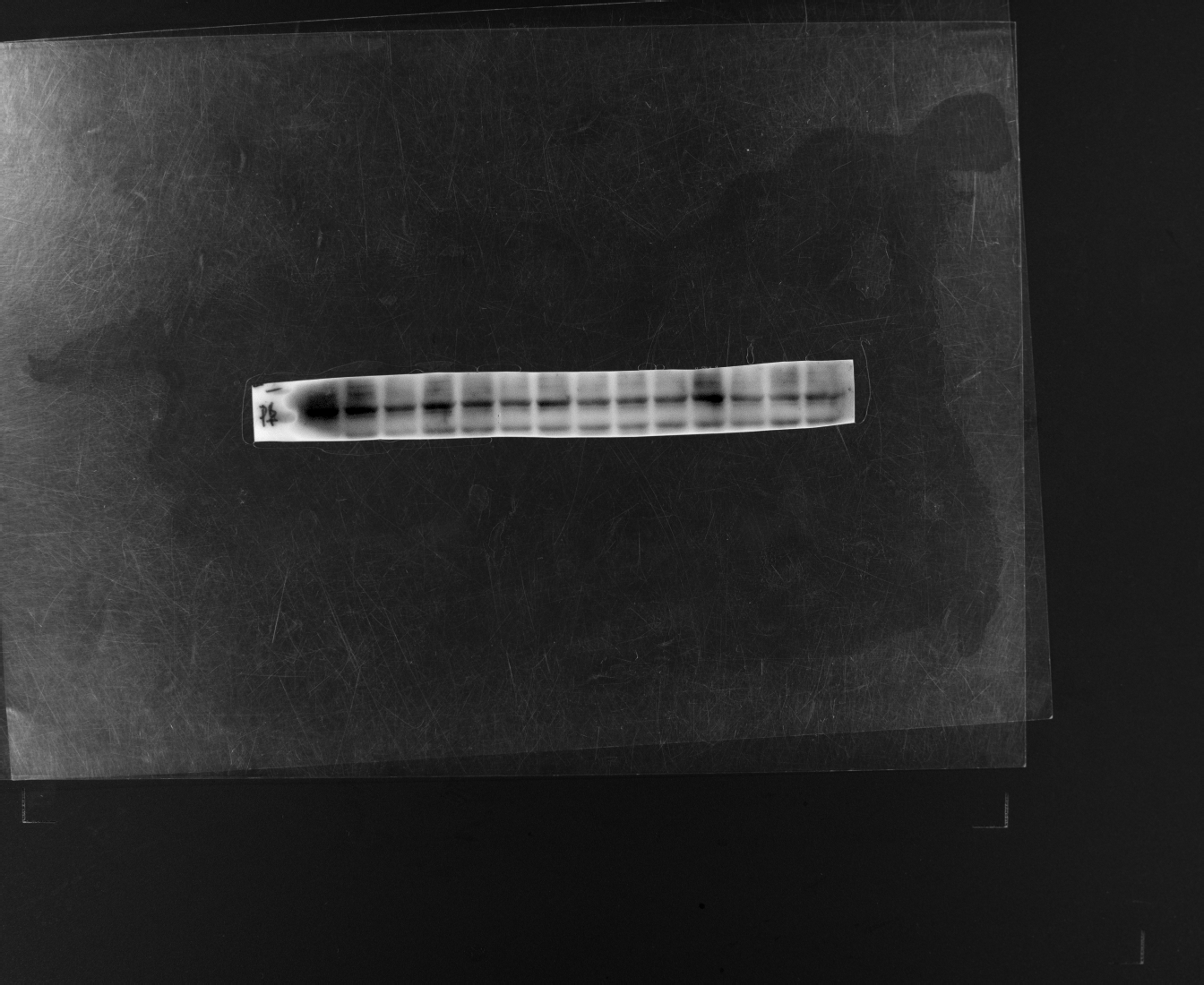

Supplement: Figure 7—source data 2. [file elife-81858-fig7-data2.zip › Figure 7-source data 2/fig7e.PKM2.tif]

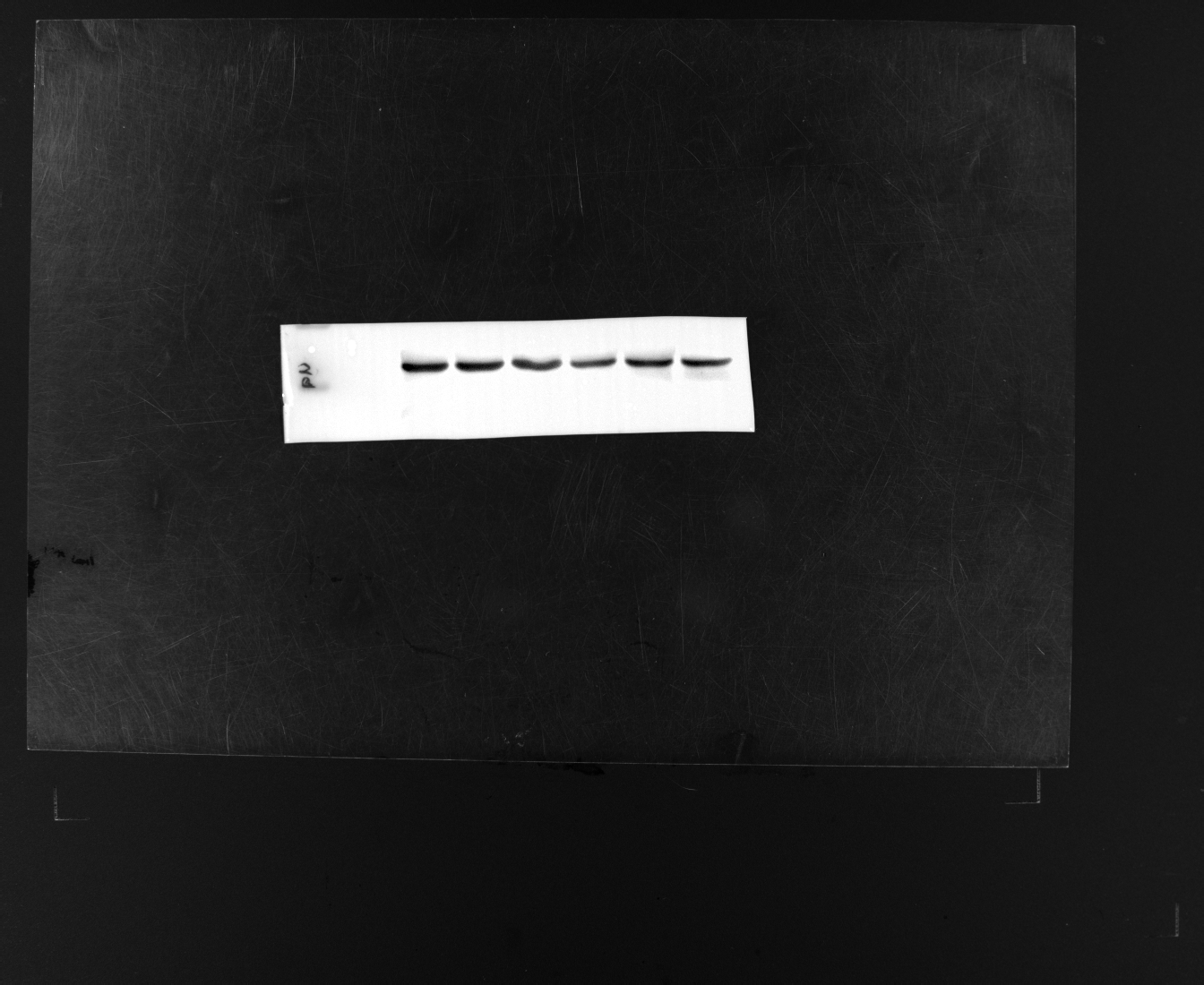

Supplement: Figure 7—source data 2. [file elife-81858-fig7-data2.zip › Figure 7-source data 2/fig7e.actin.tif]

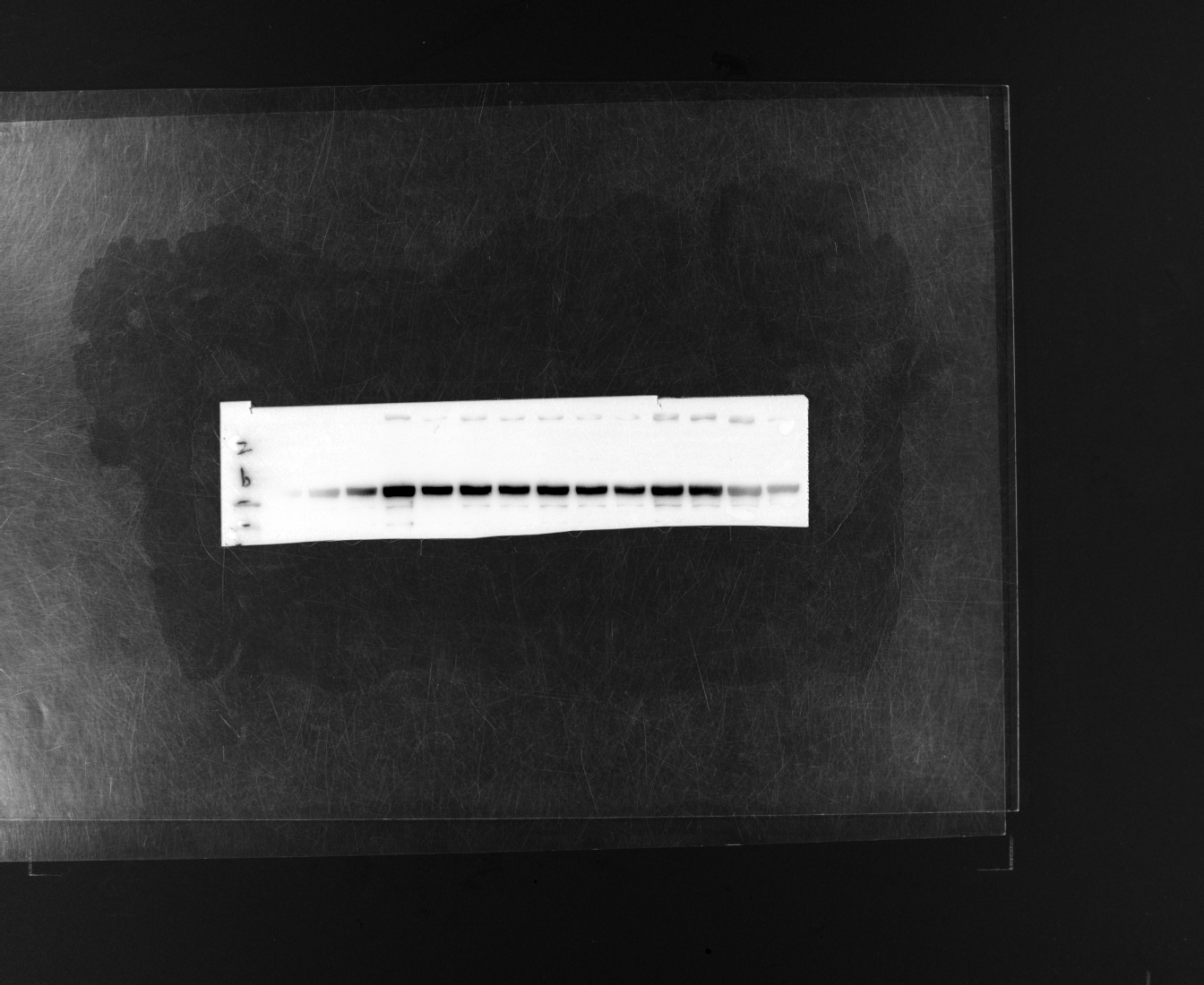

Supplement: Figure 7—source data 2. [file elife-81858-fig7-data2.zip › Figure 7-source data 2/fig7f.actin.tif]

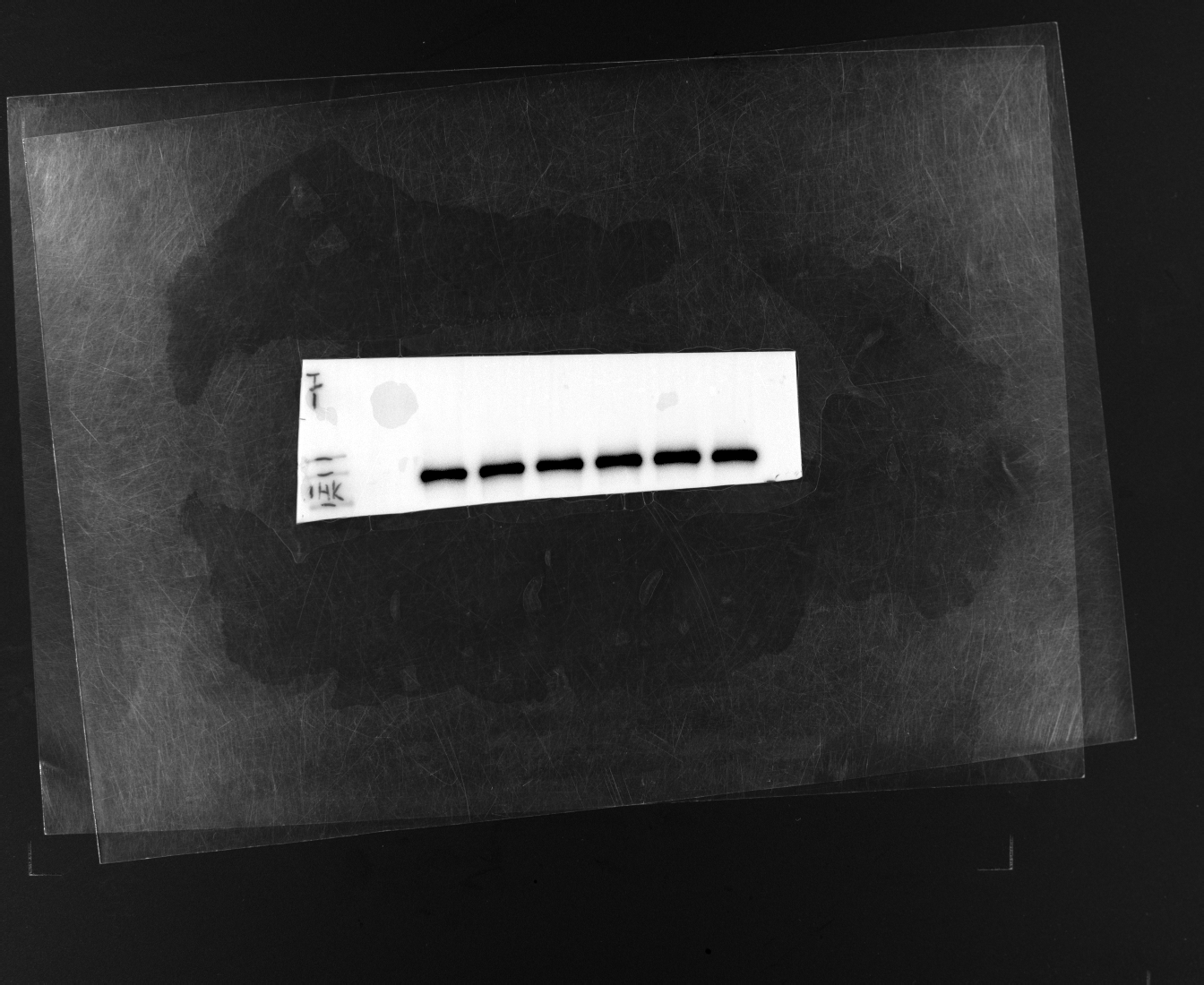

Supplement: Figure 7—source data 2. [file elife-81858-fig7-data2.zip › Figure 7-source data 2/fig7f.akt1.tif]

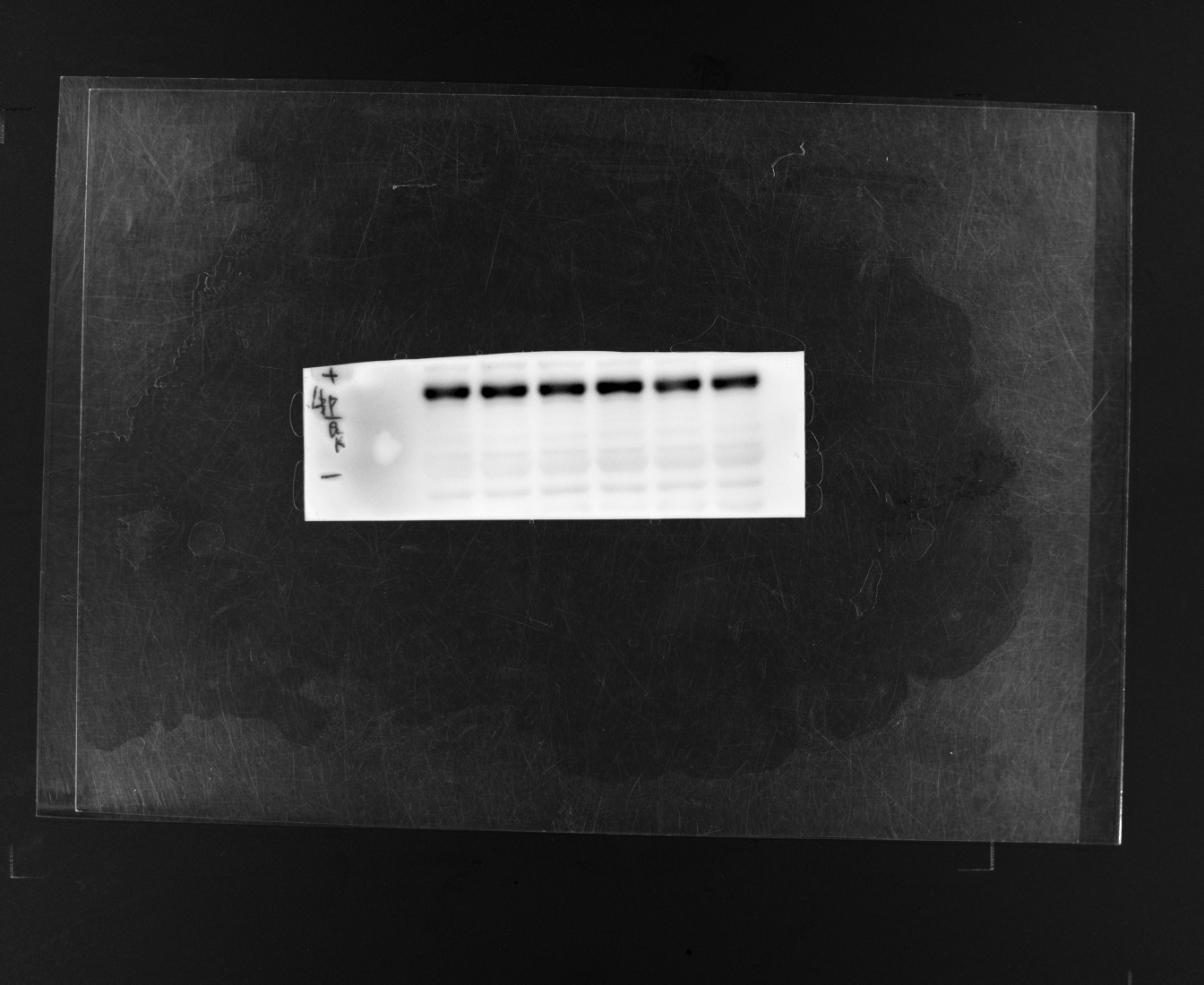

Supplement: Figure 7—source data 2. [file elife-81858-fig7-data2.zip › Figure 7-source data 2/fig7f.mTOR.tif]

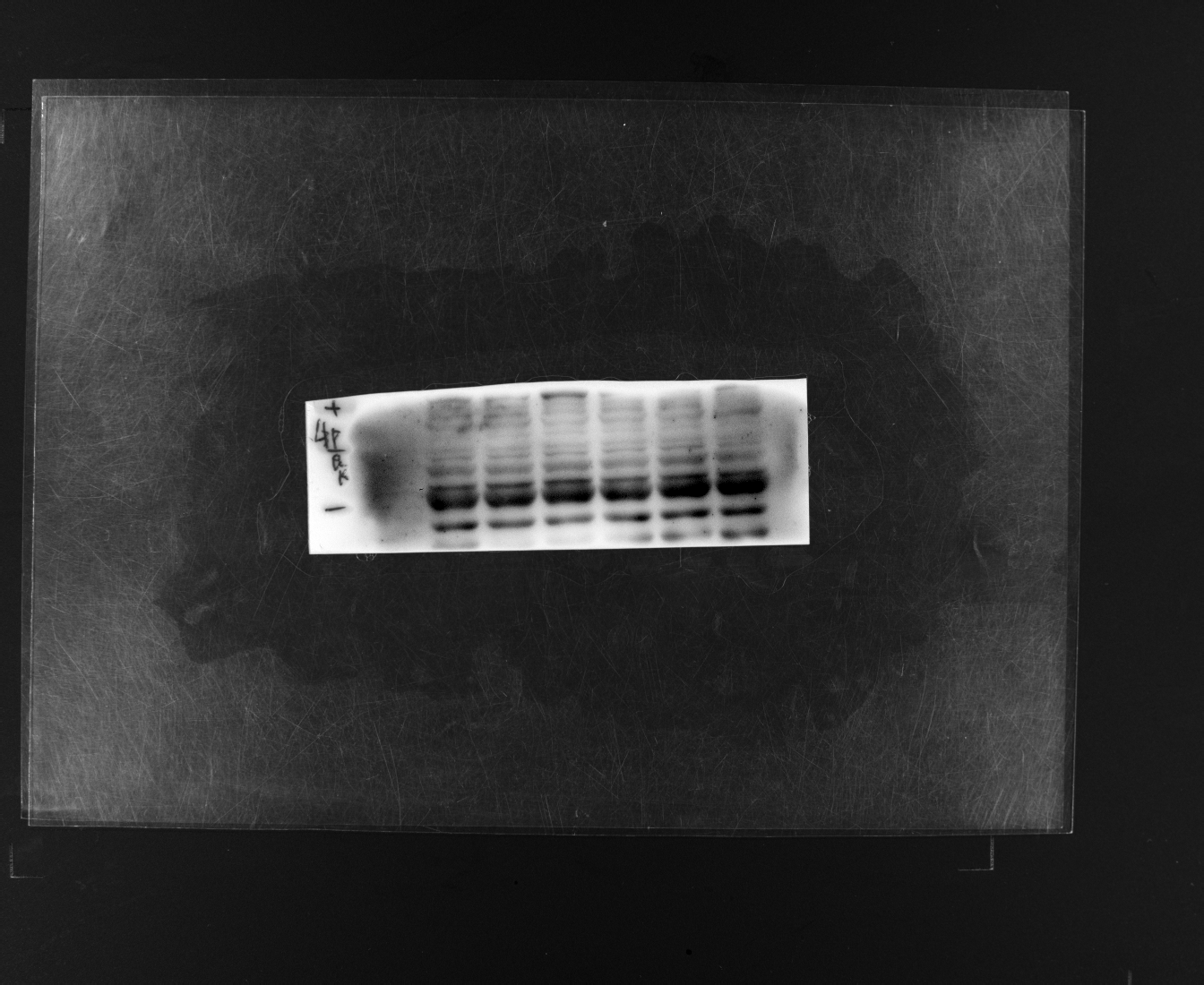

Supplement: Figure 7—source data 2. [file elife-81858-fig7-data2.zip › Figure 7-source data 2/fig7f.p-akt1.tif]

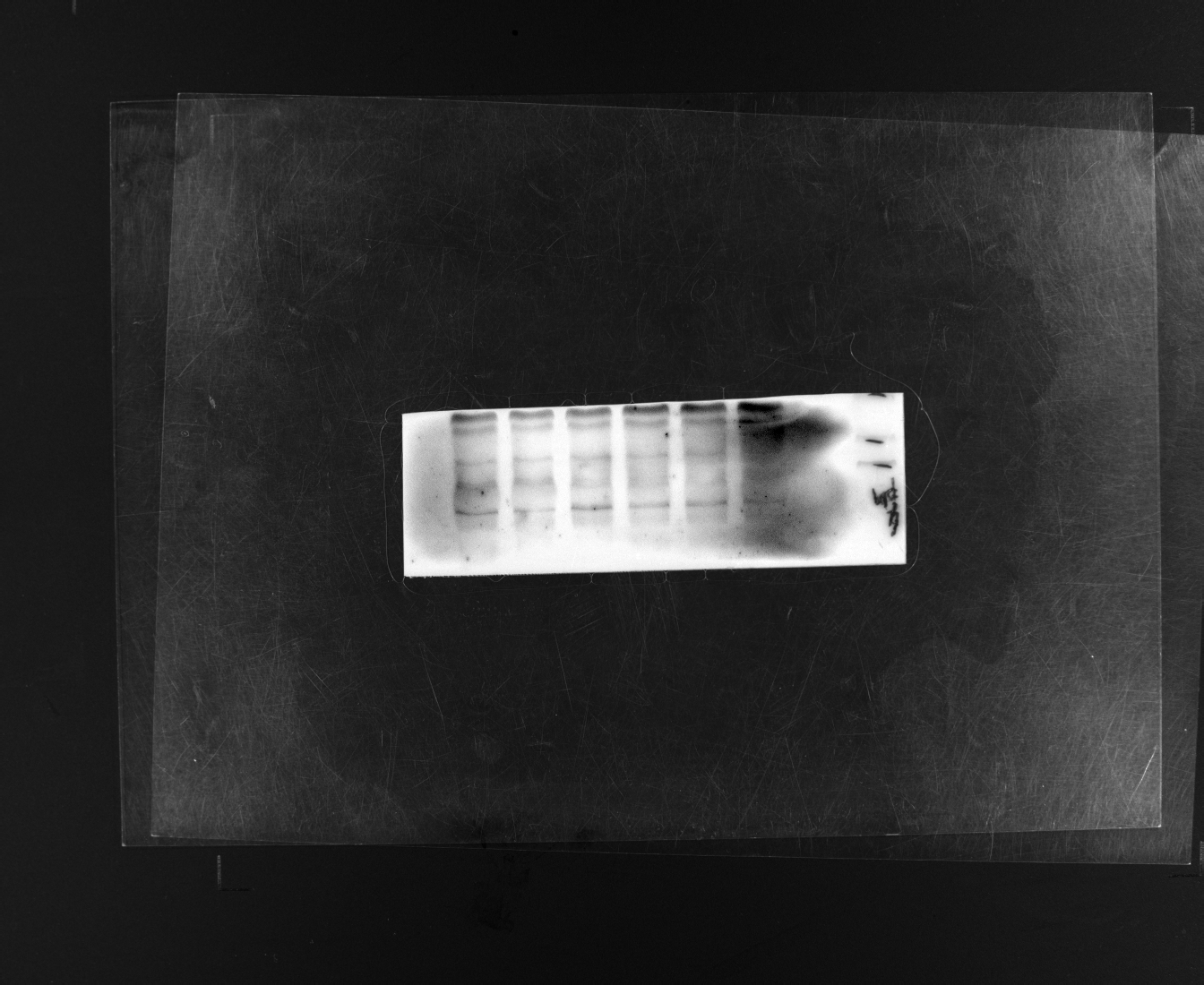

Supplement: Figure 7—source data 2. [file elife-81858-fig7-data2.zip › Figure 7-source data 2/fig7f.p-mTOR.tif]

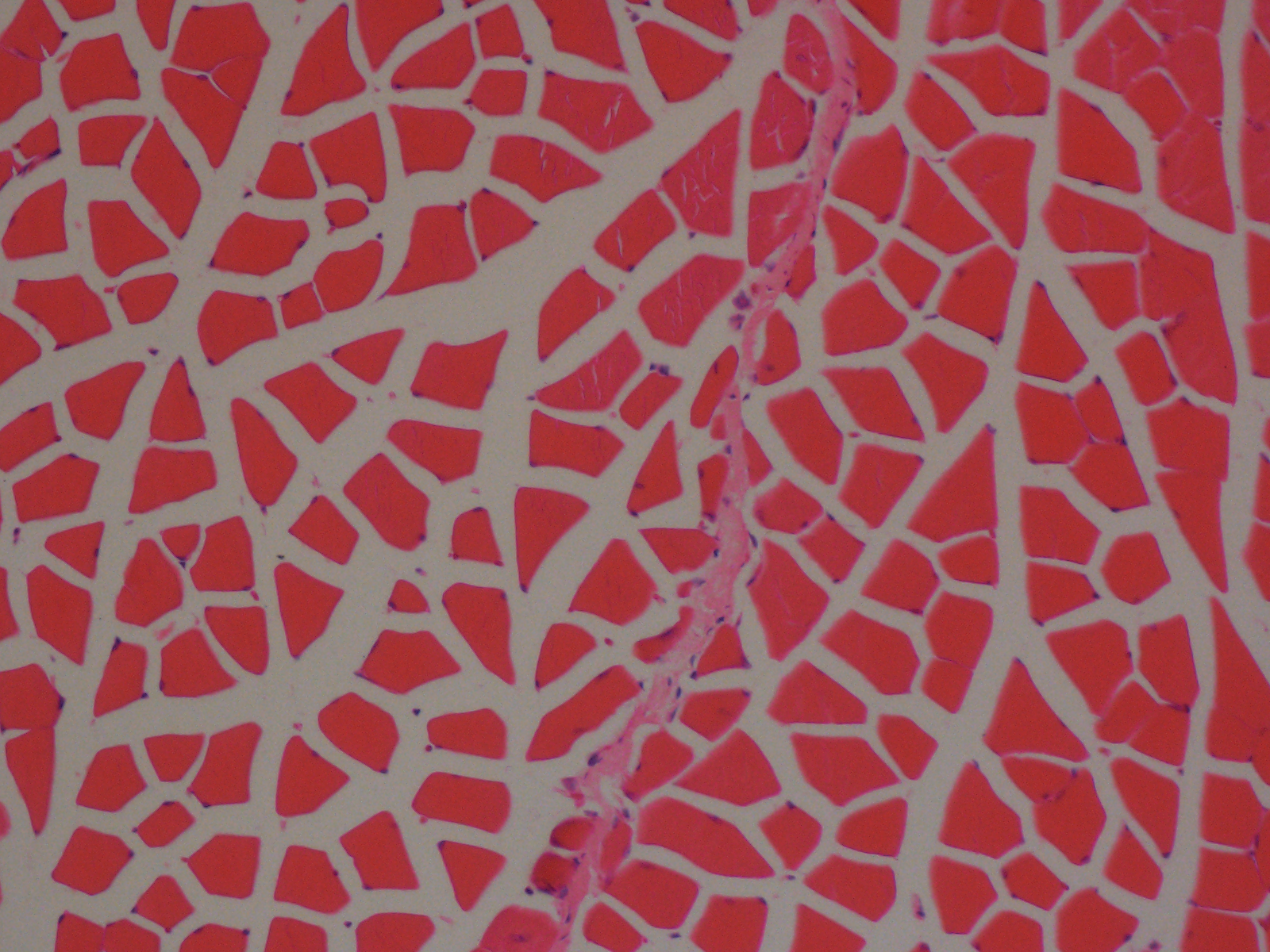

Supplement: Figure 8—source data 1. [file elife-81858-fig8-data1.zip › Figure 8-source data 1/fig8.b/Inhibitor/In (1).tif]

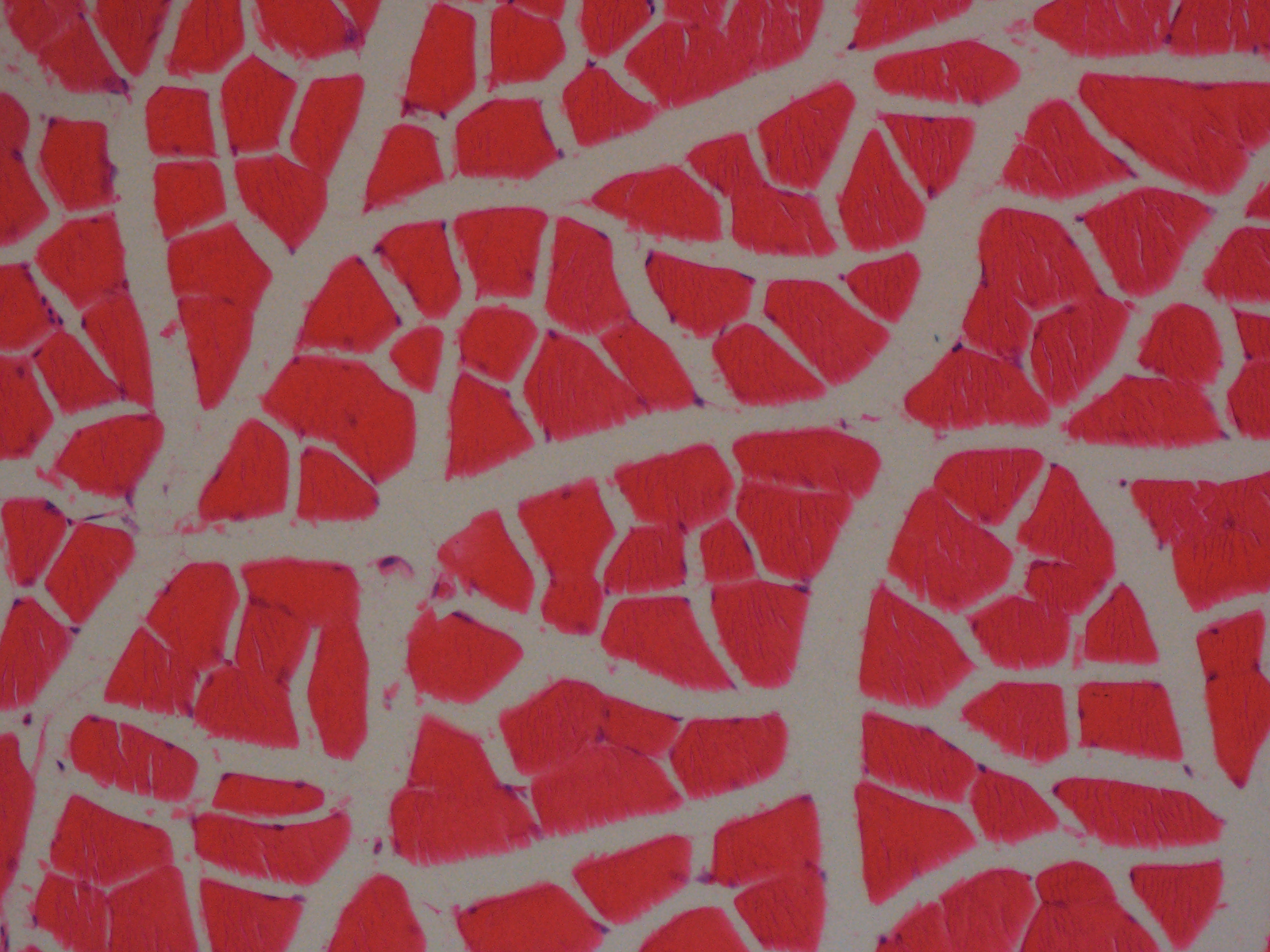

Supplement: Figure 8—source data 1. [file elife-81858-fig8-data1.zip › Figure 8-source data 1/fig8.b/Inhibitor/In (2).tif]

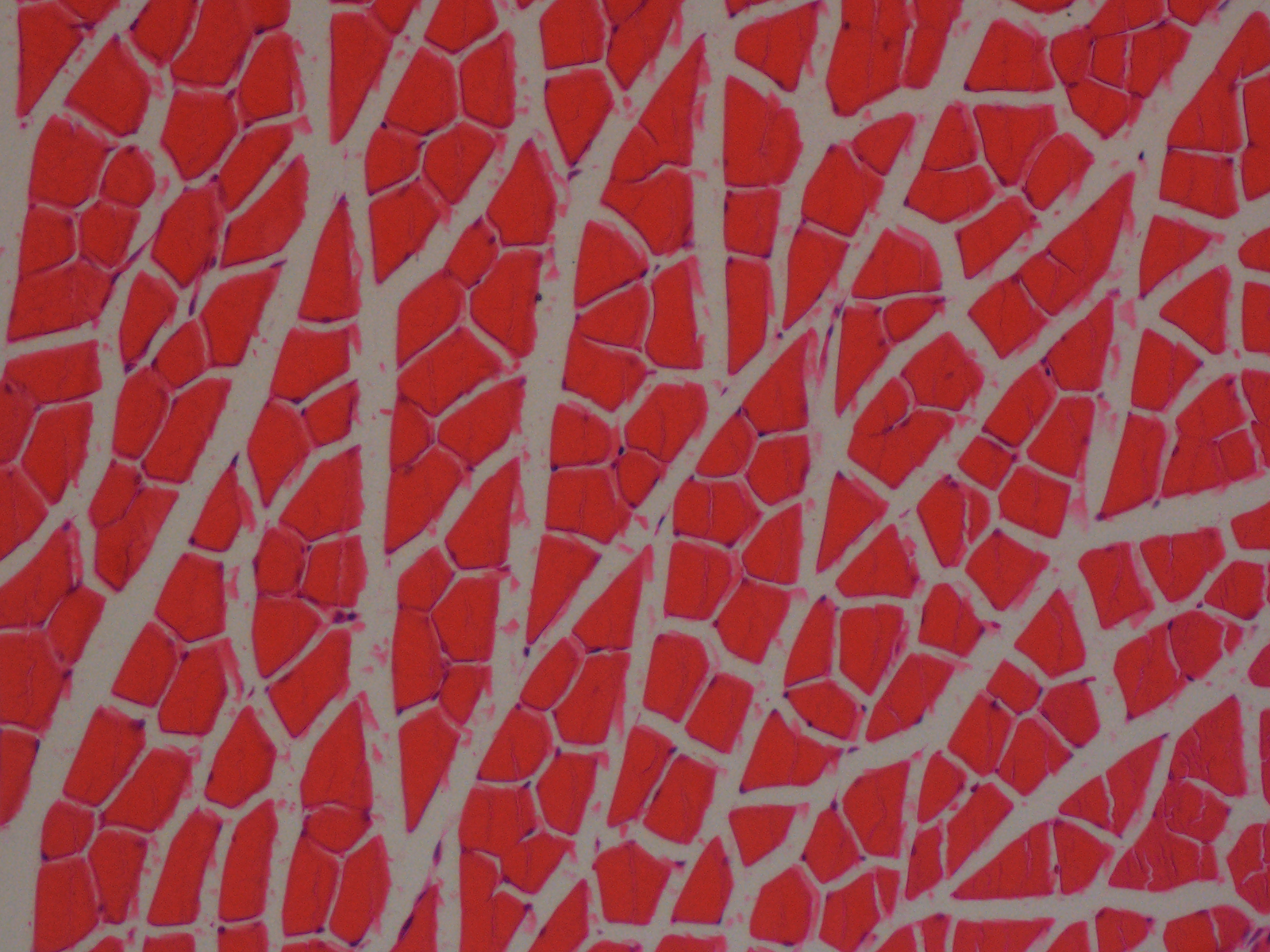

Supplement: Figure 8—source data 1. [file elife-81858-fig8-data1.zip › Figure 8-source data 1/fig8.b/Inhibitor/In (3).tif]

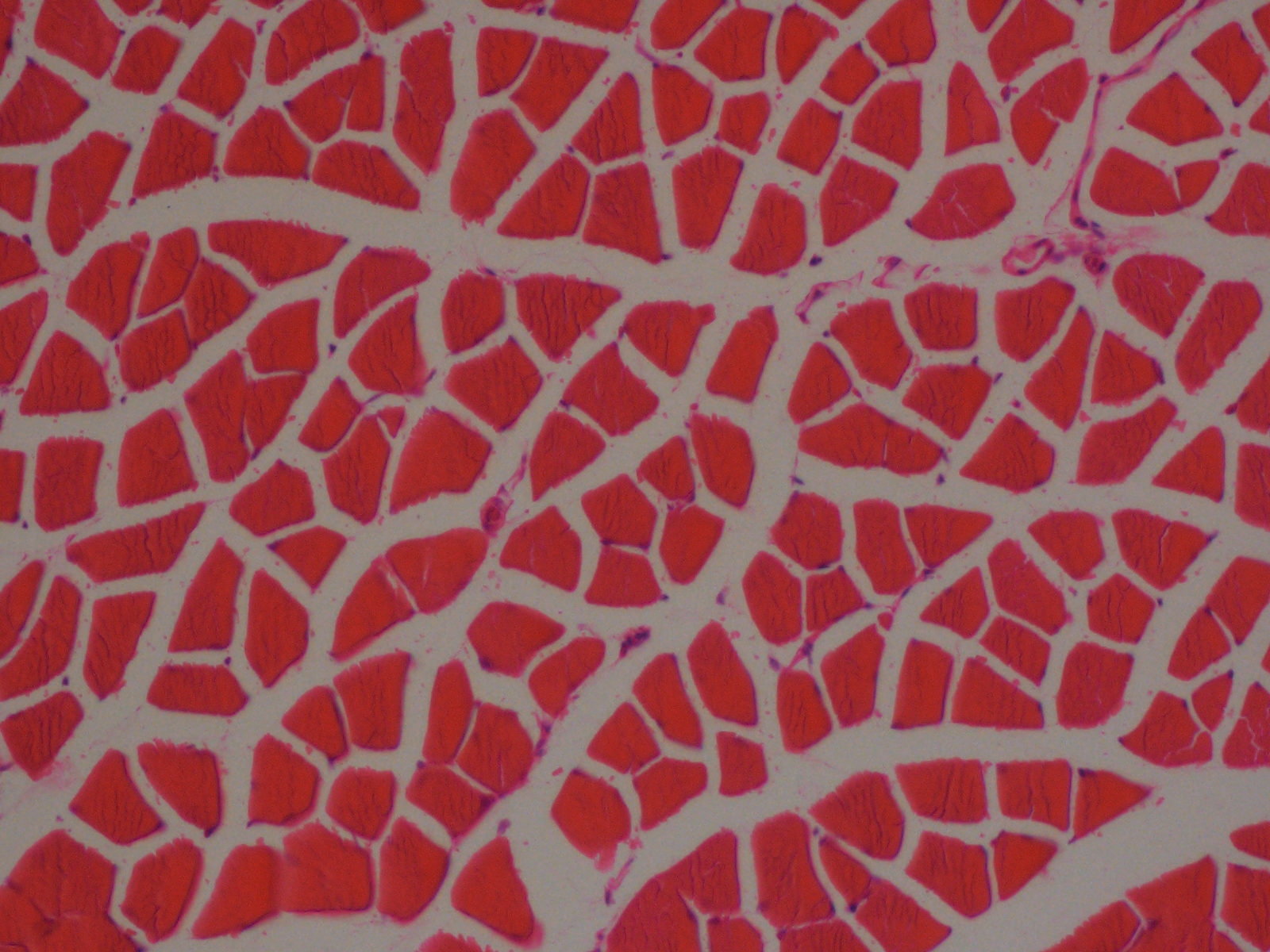

Supplement: Figure 8—source data 1. [file elife-81858-fig8-data1.zip › Figure 8-source data 1/fig8.b/Inhibitor/In (4).tif]

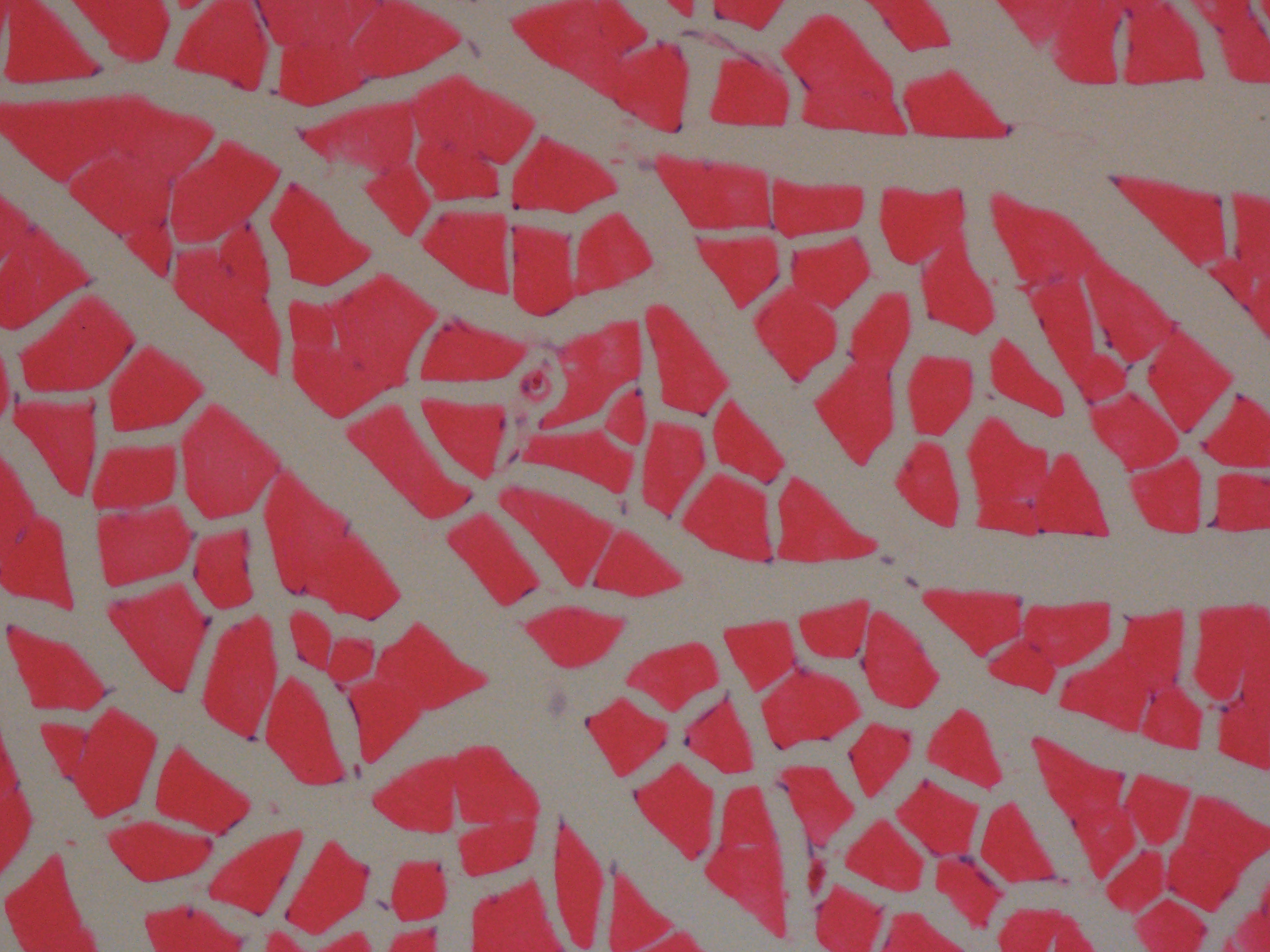

Supplement: Figure 8—source data 1. [file elife-81858-fig8-data1.zip › Figure 8-source data 1/fig8.b/Inhibitor/In (5).tif]

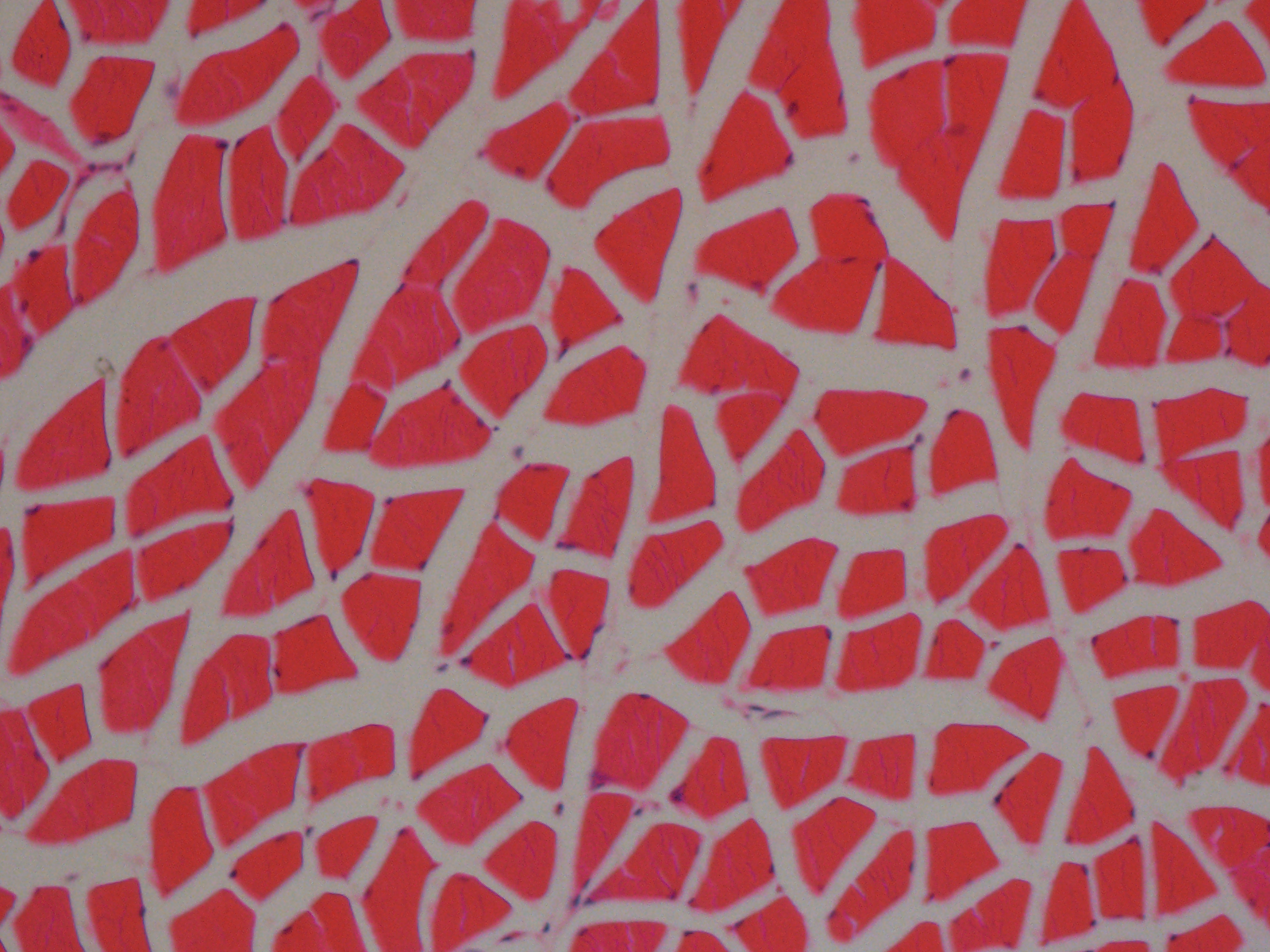

Supplement: Figure 8—source data 1. [file elife-81858-fig8-data1.zip › Figure 8-source data 1/fig8.b/Inhibitor+Val/In+Val (1).tif]

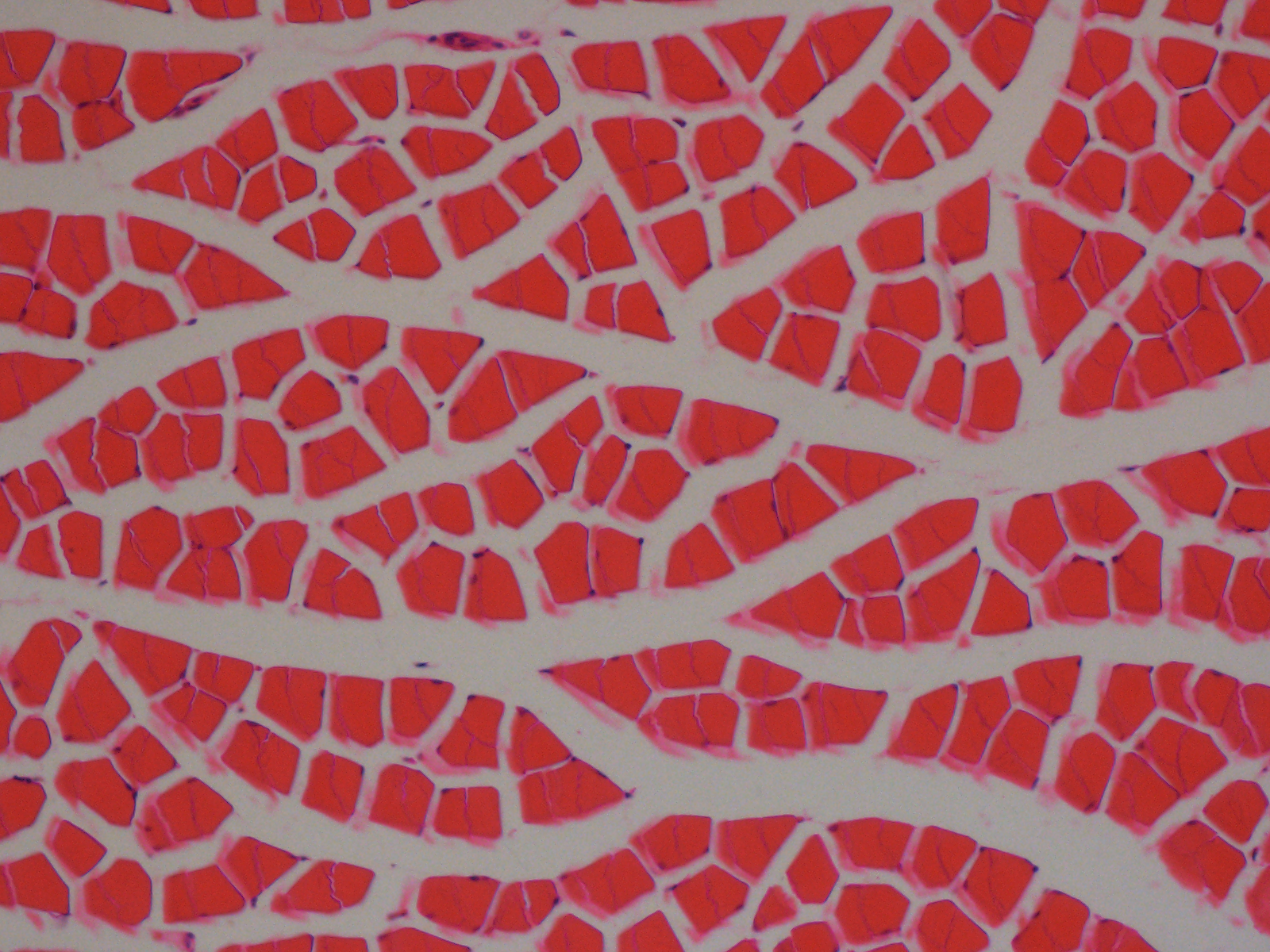

Supplement: Figure 8—source data 1. [file elife-81858-fig8-data1.zip › Figure 8-source data 1/fig8.b/Inhibitor+Val/In+Val (2).tif]

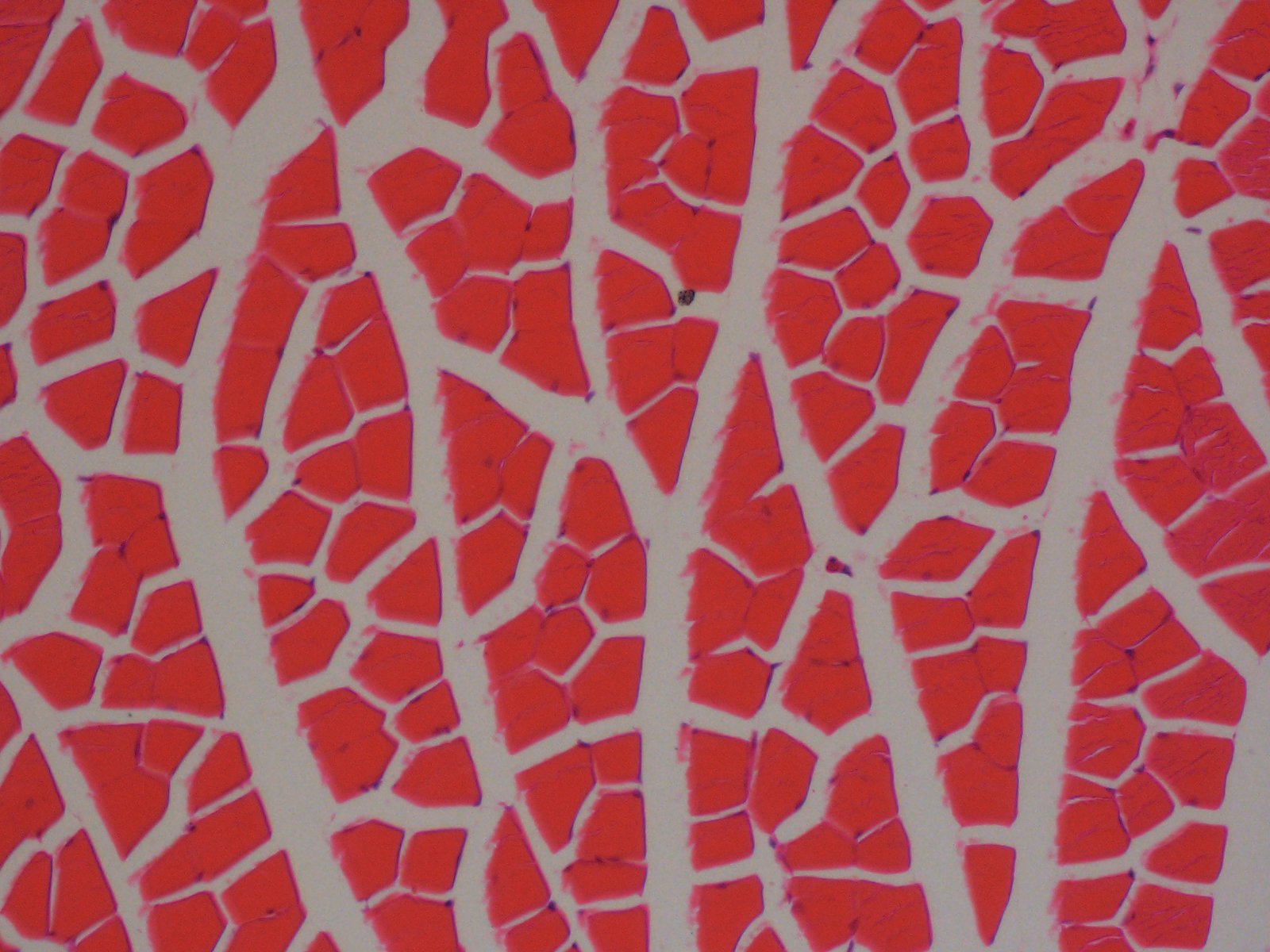

Supplement: Figure 8—source data 1. [file elife-81858-fig8-data1.zip › Figure 8-source data 1/fig8.b/Inhibitor+Val/In+Val (3).tif]

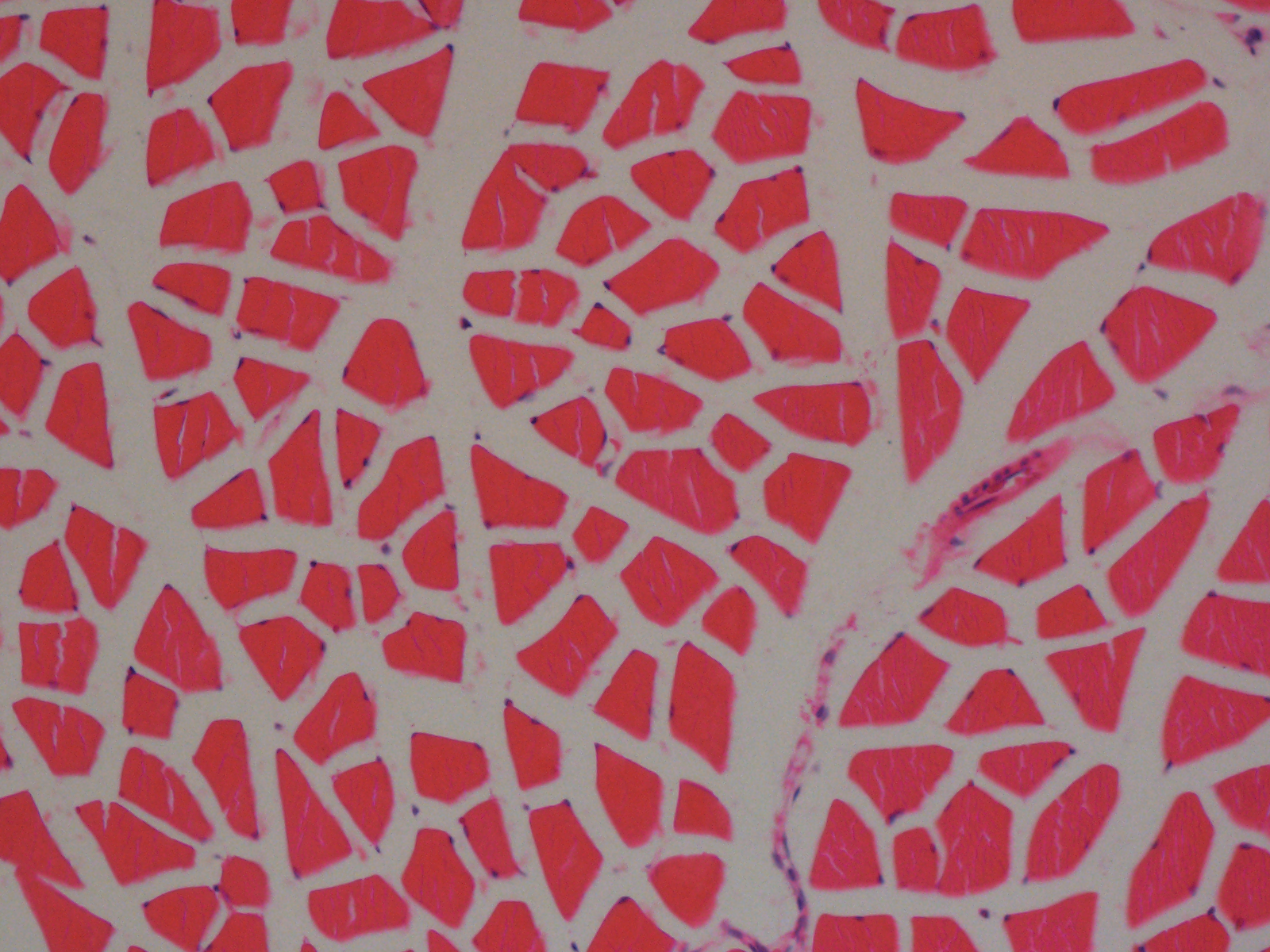

Supplement: Figure 8—source data 1. [file elife-81858-fig8-data1.zip › Figure 8-source data 1/fig8.b/Inhibitor+Val/In+Val (4).tif]

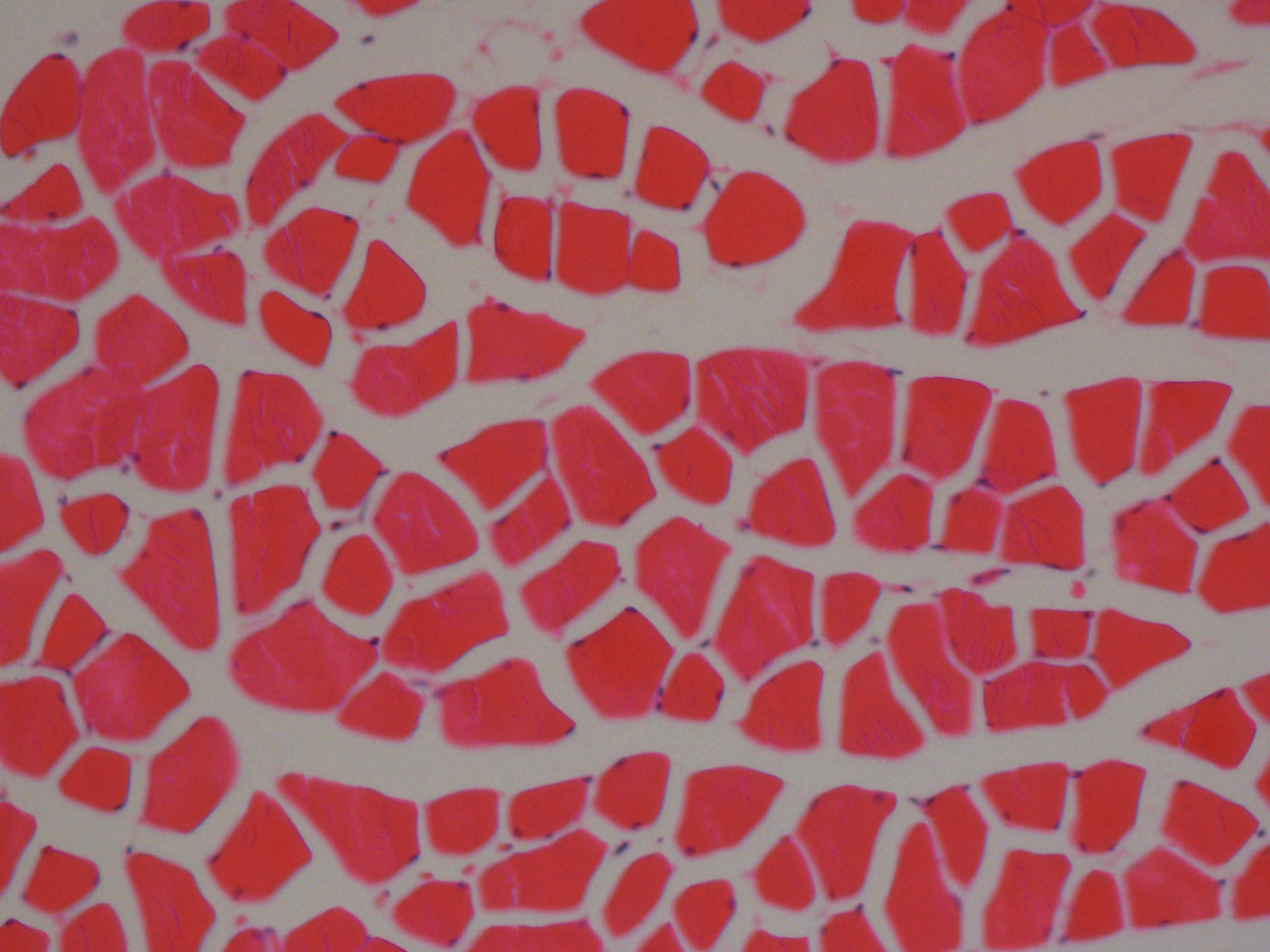

Supplement: Figure 8—source data 1. [file elife-81858-fig8-data1.zip › Figure 8-source data 1/fig8.b/Inhibitor+Val/In+Val (5).tif]

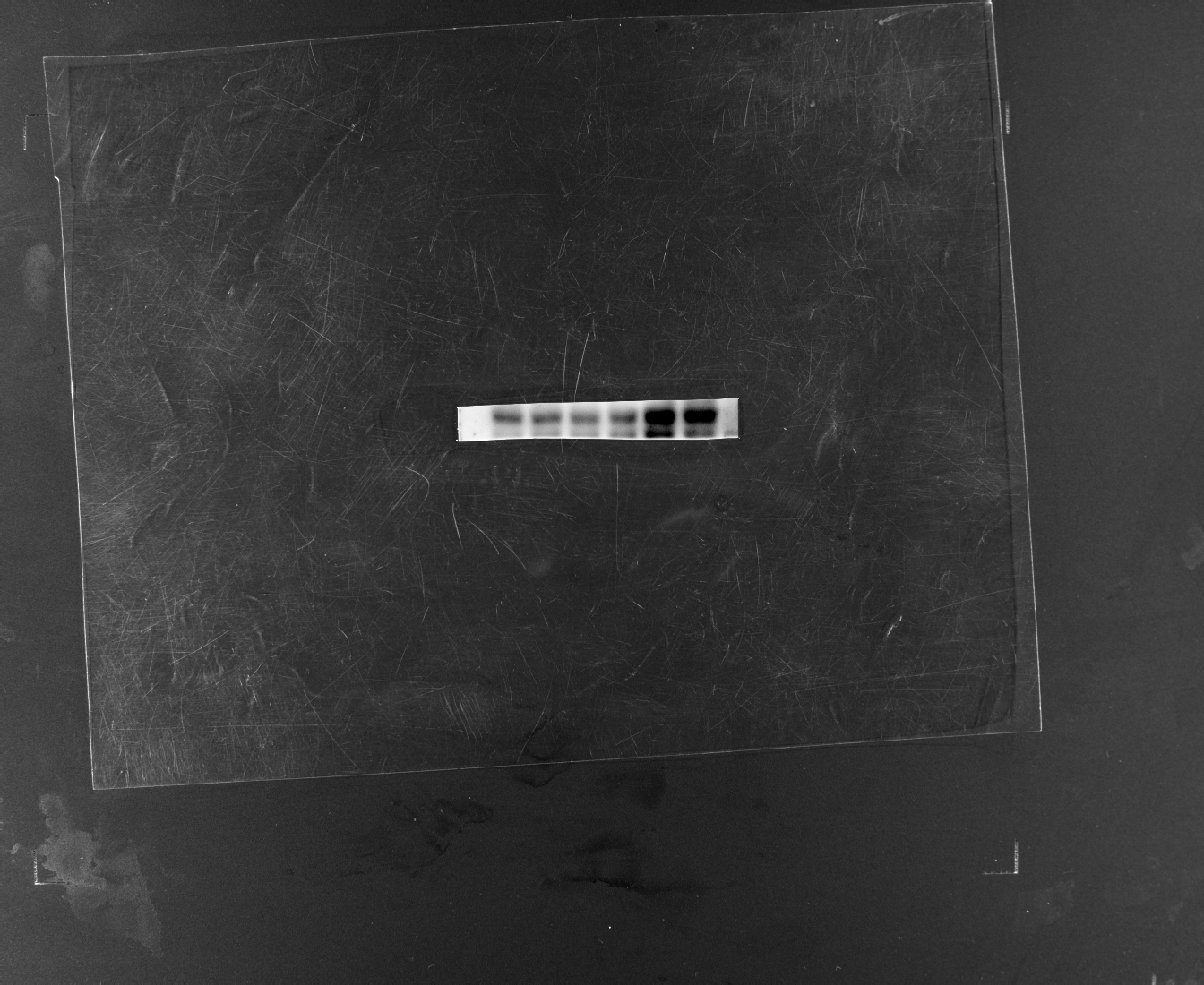

Supplement: Figure 8—source data 2. [file elife-81858-fig8-data2.zip › Figure 8-source data 2/fig8d.GPR43.tif]

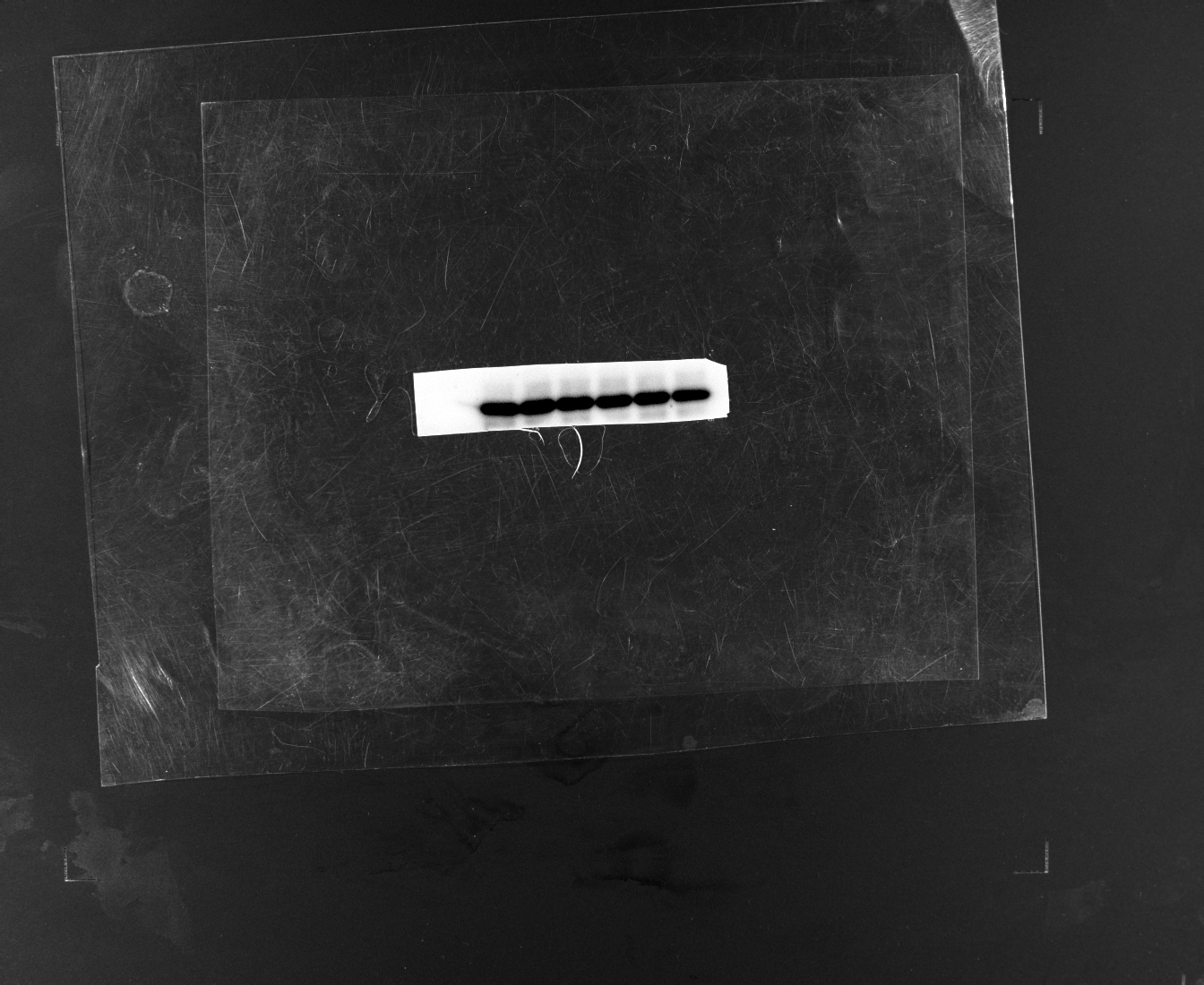

Supplement: Figure 8—source data 2. [file elife-81858-fig8-data2.zip › Figure 8-source data 2/fig8d.actin.tif]

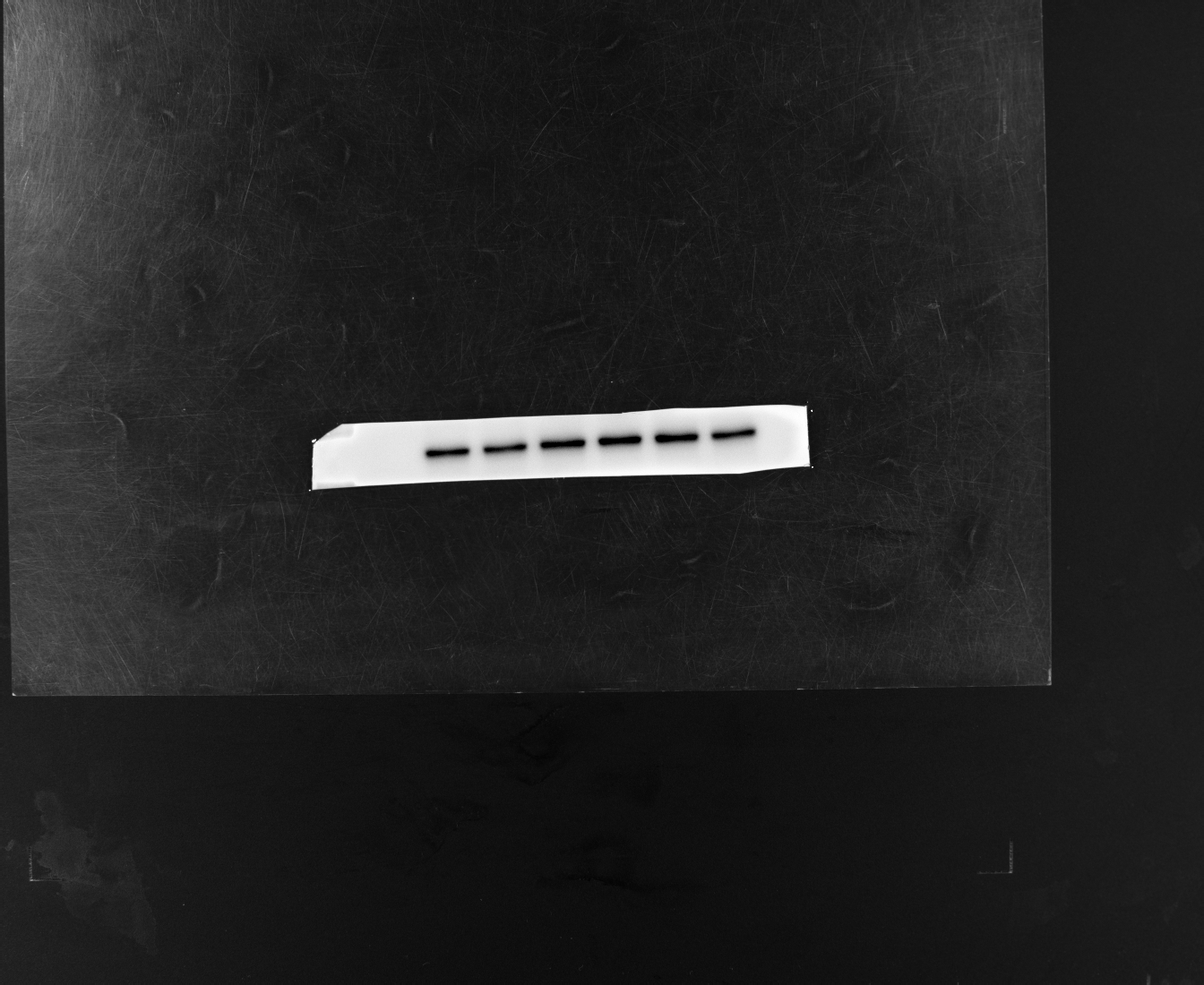

Supplement: Figure 8—source data 2. [file elife-81858-fig8-data2.zip › Figure 8-source data 2/fig8d.akt1.tif]

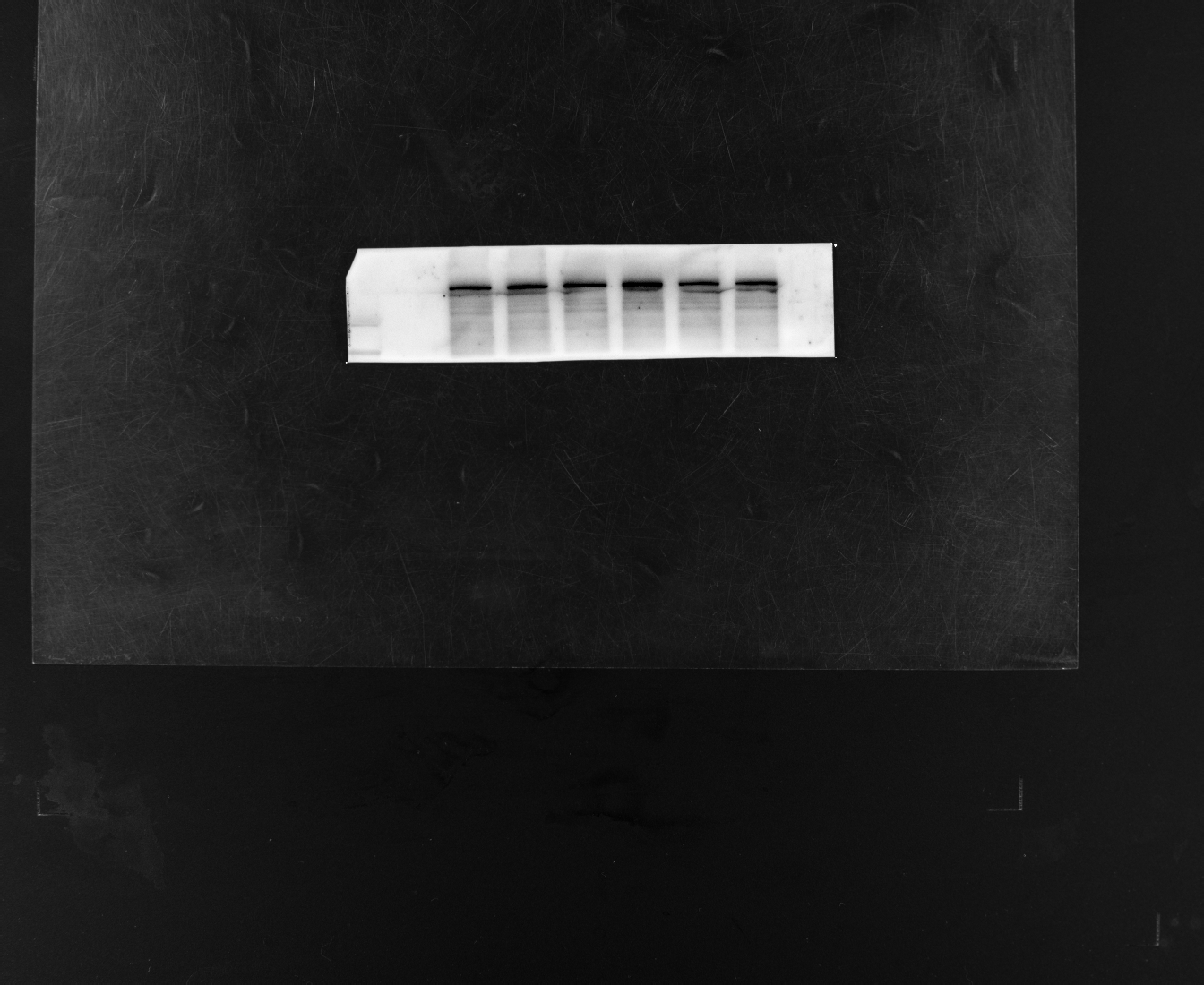

Supplement: Figure 8—source data 2. [file elife-81858-fig8-data2.zip › Figure 8-source data 2/fig8d.mTOR.tif]

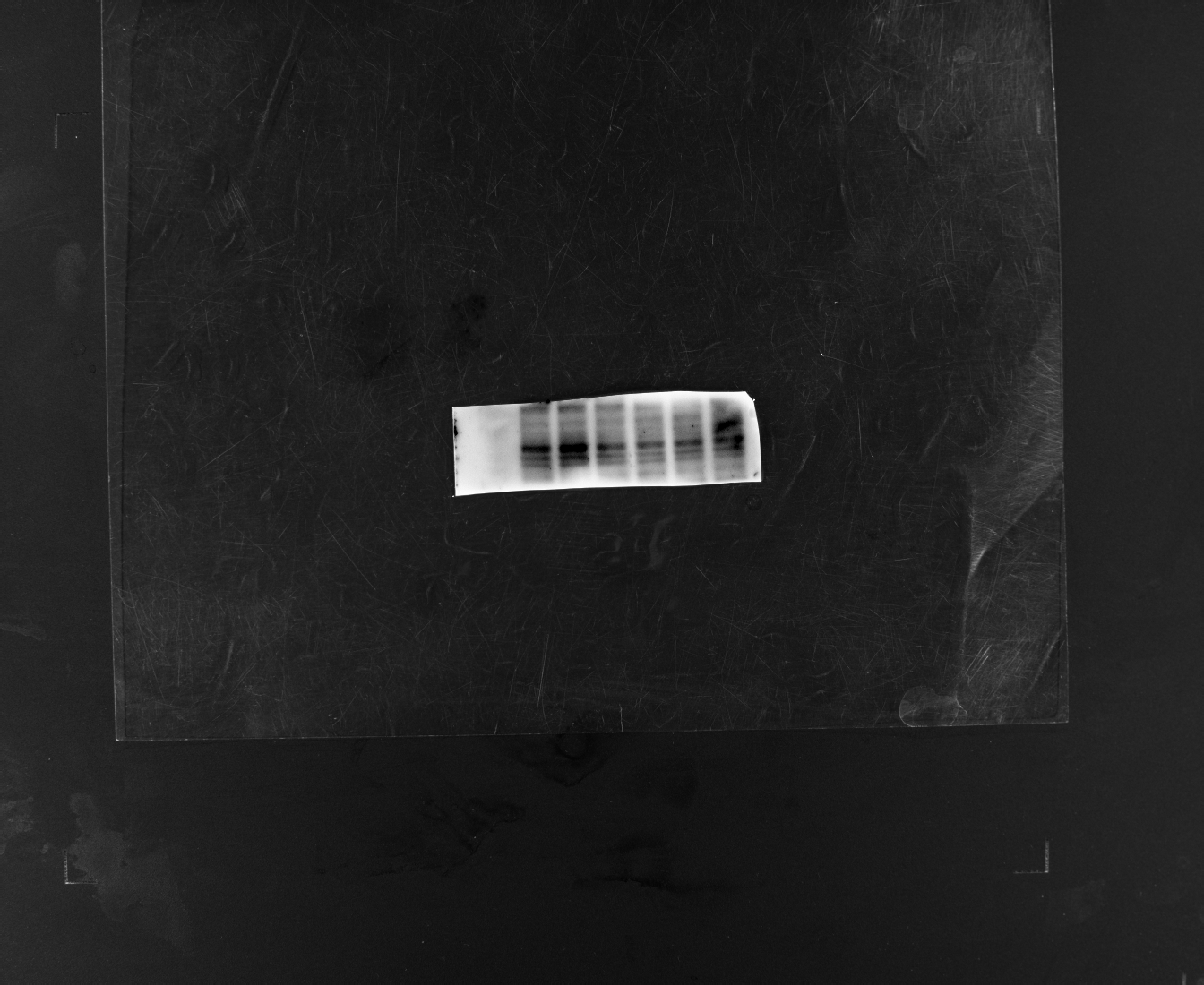

Supplement: Figure 8—source data 2. [file elife-81858-fig8-data2.zip › Figure 8-source data 2/fig8d.pakt1.tif]

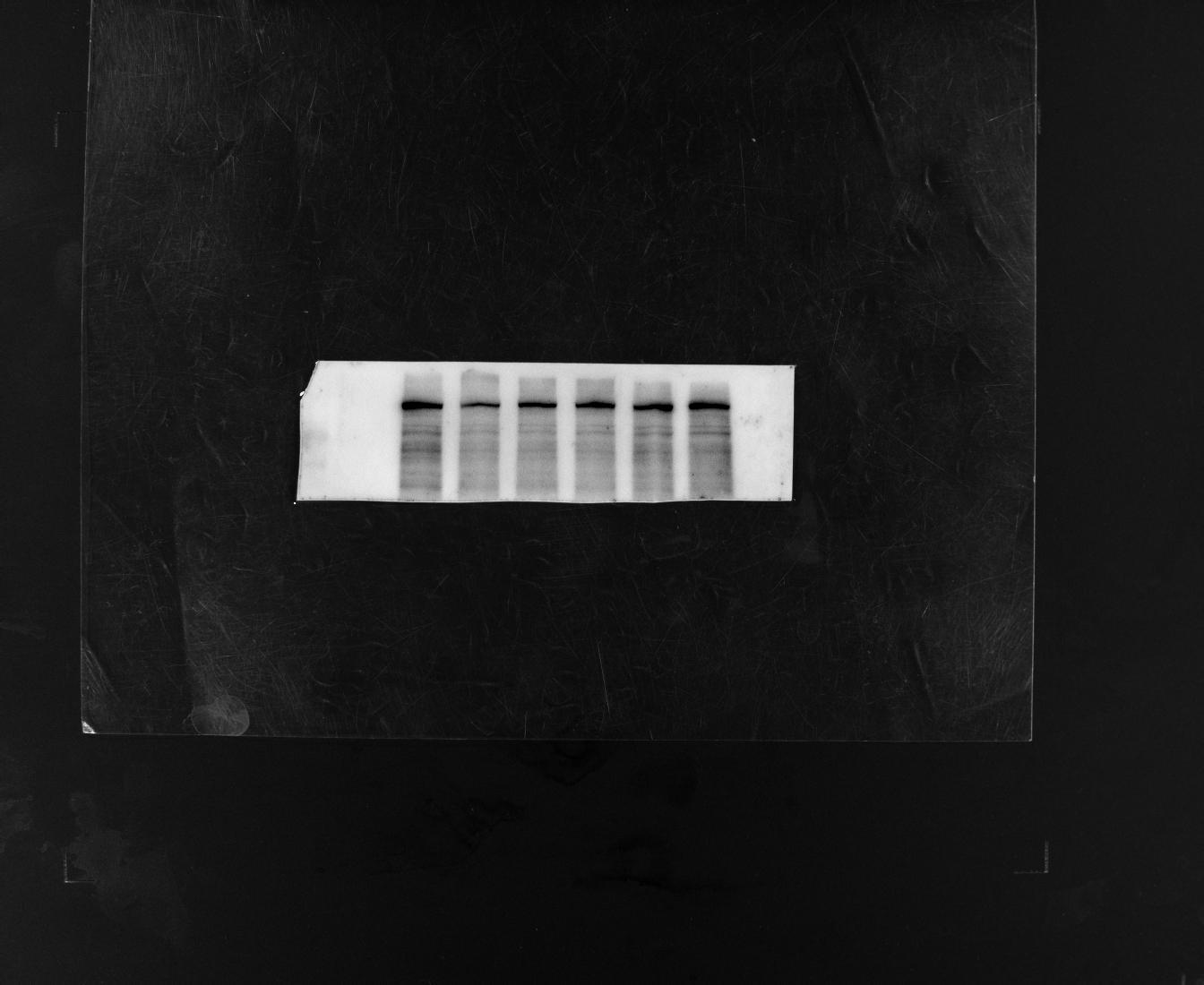

Supplement: Figure 8—source data 2. [file elife-81858-fig8-data2.zip › Figure 8-source data 2/fig8d.pmTOR.tif]

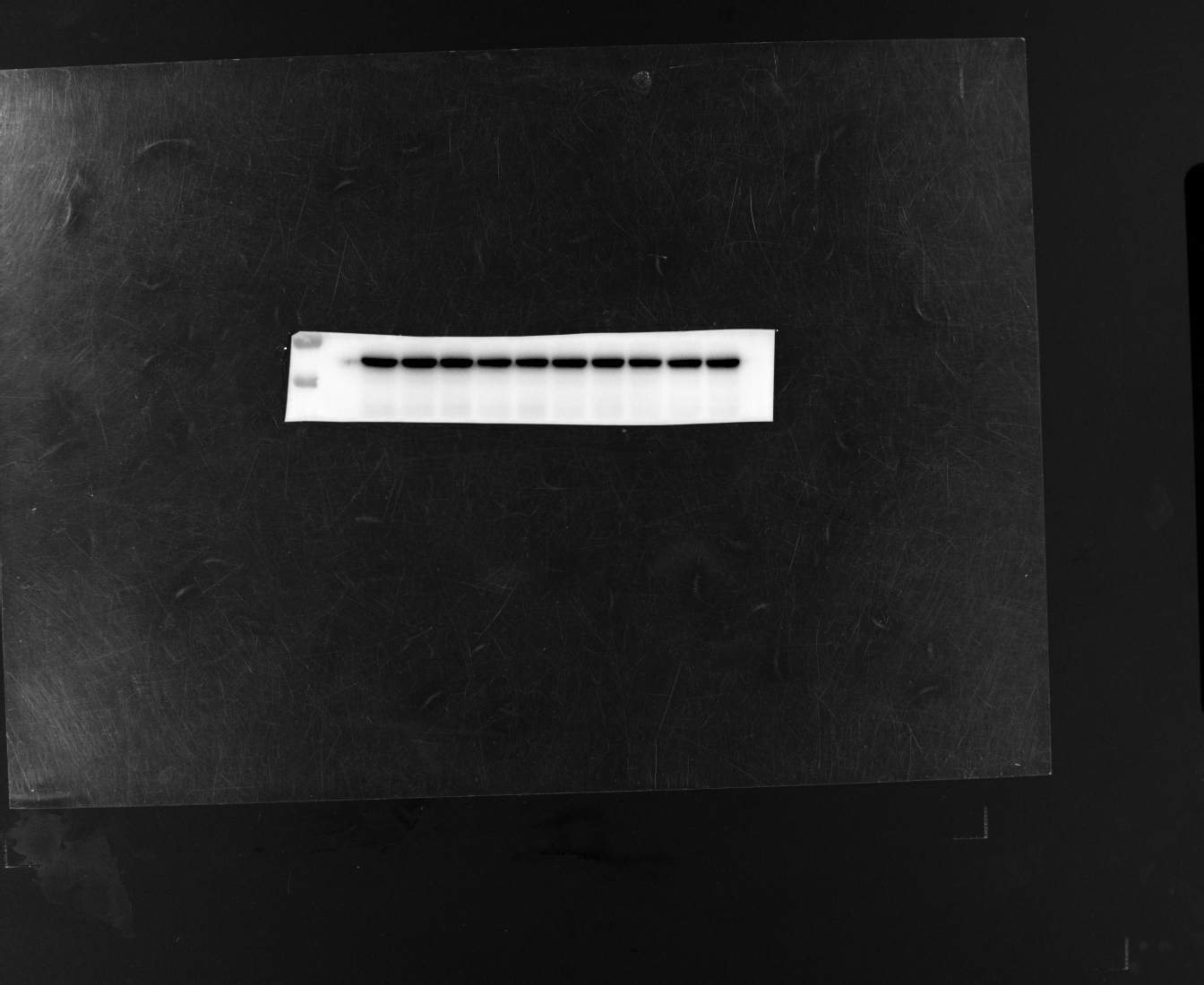

Supplement: Figure 8—source data 2. [file elife-81858-fig8-data2.zip › Figure 8-source data 2/fig8f.actin.tif]

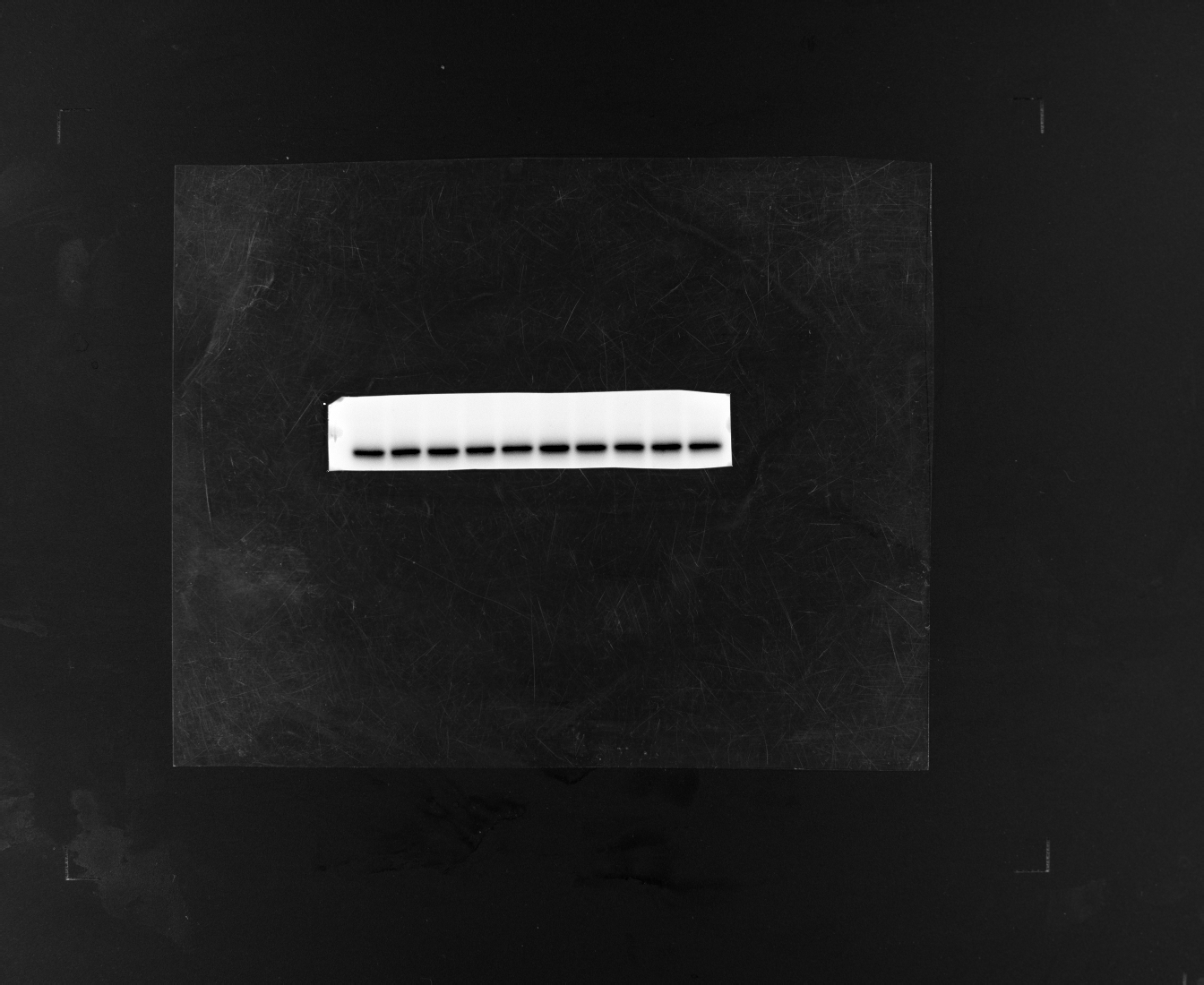

Supplement: Figure 8—source data 2. [file elife-81858-fig8-data2.zip › Figure 8-source data 2/fig8f.akt1.tif]

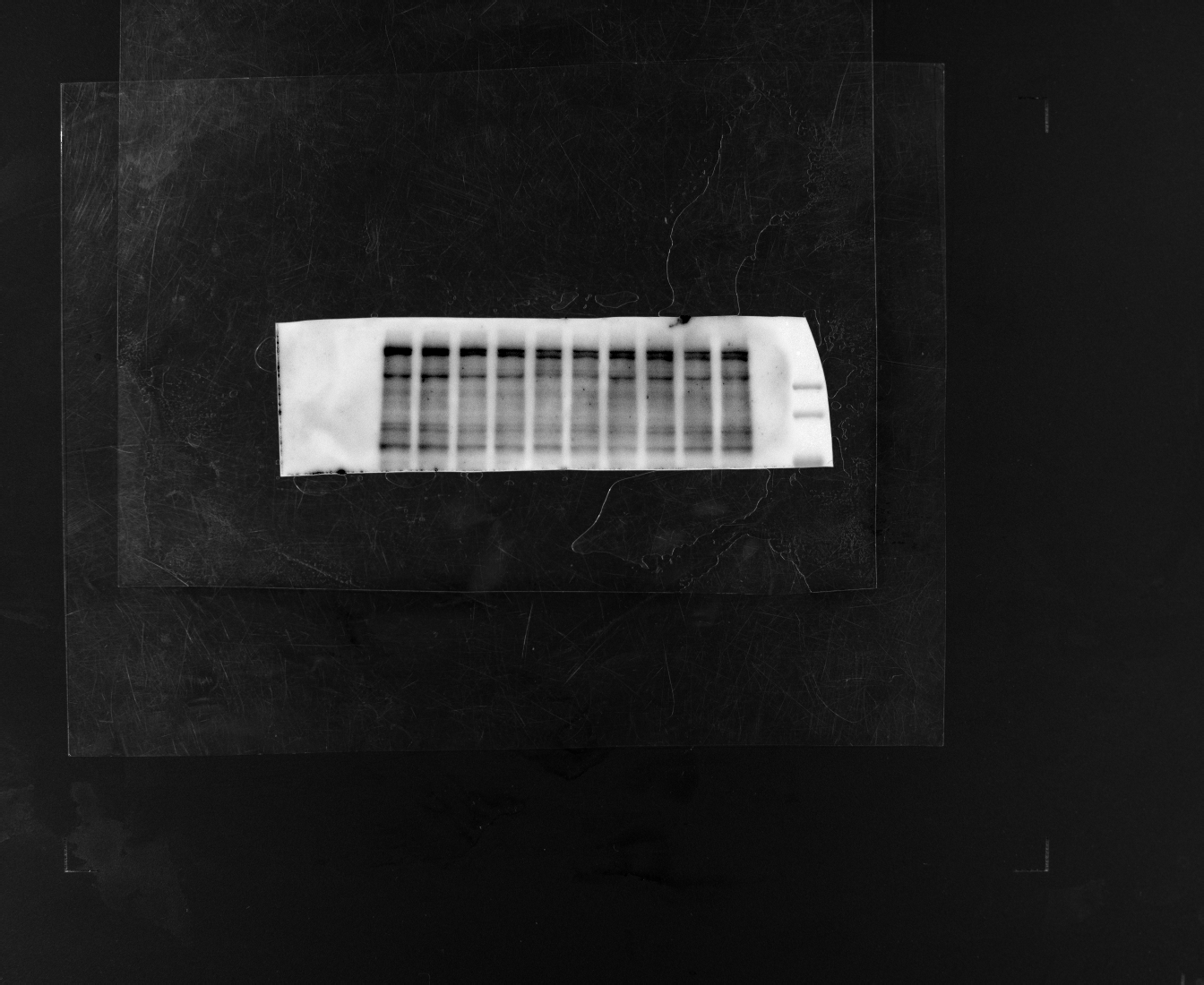

Supplement: Figure 8—source data 2. [file elife-81858-fig8-data2.zip › Figure 8-source data 2/fig8f.mTOR.tif]

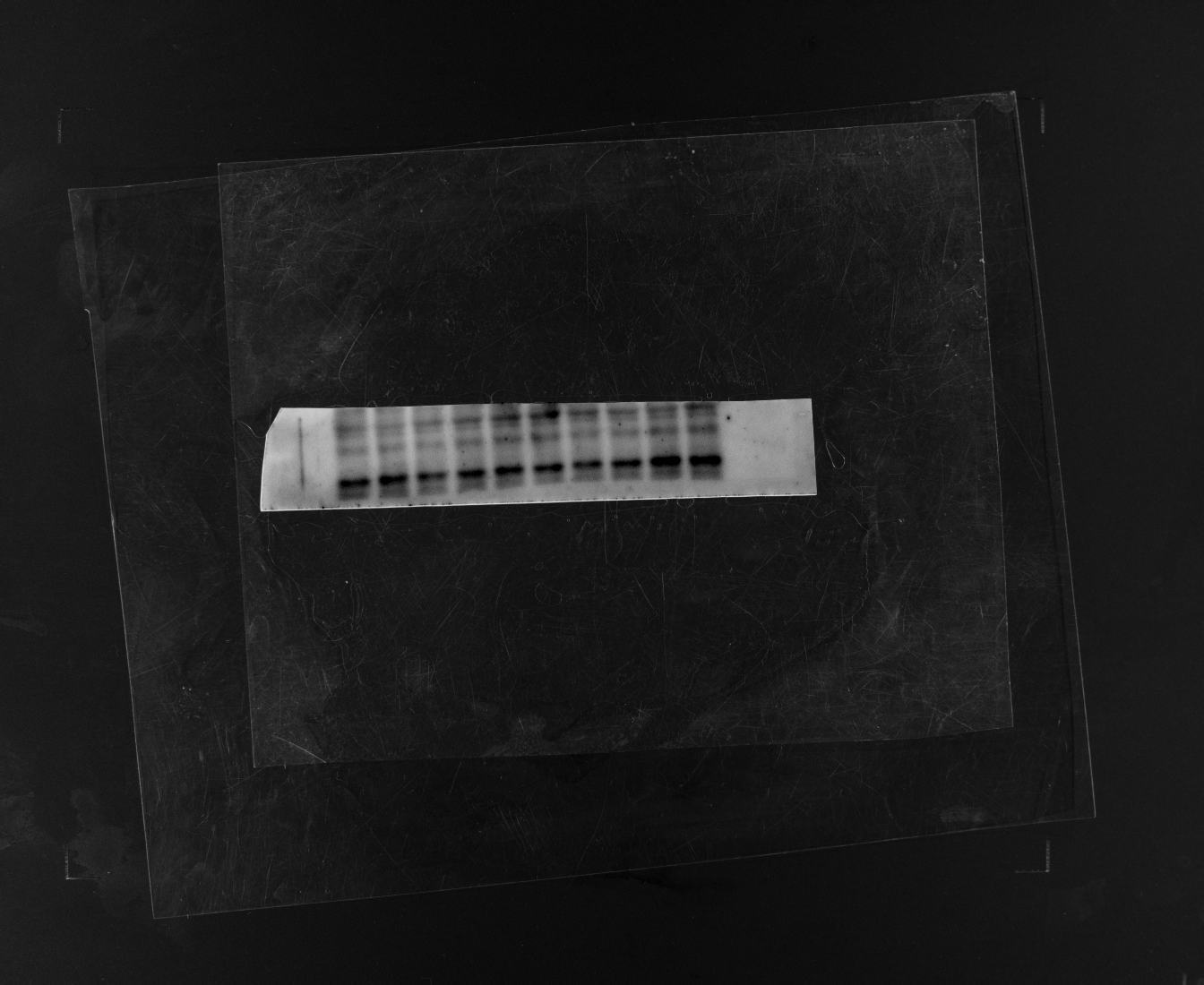

Supplement: Figure 8—source data 2. [file elife-81858-fig8-data2.zip › Figure 8-source data 2/fig8f.myod.tif]

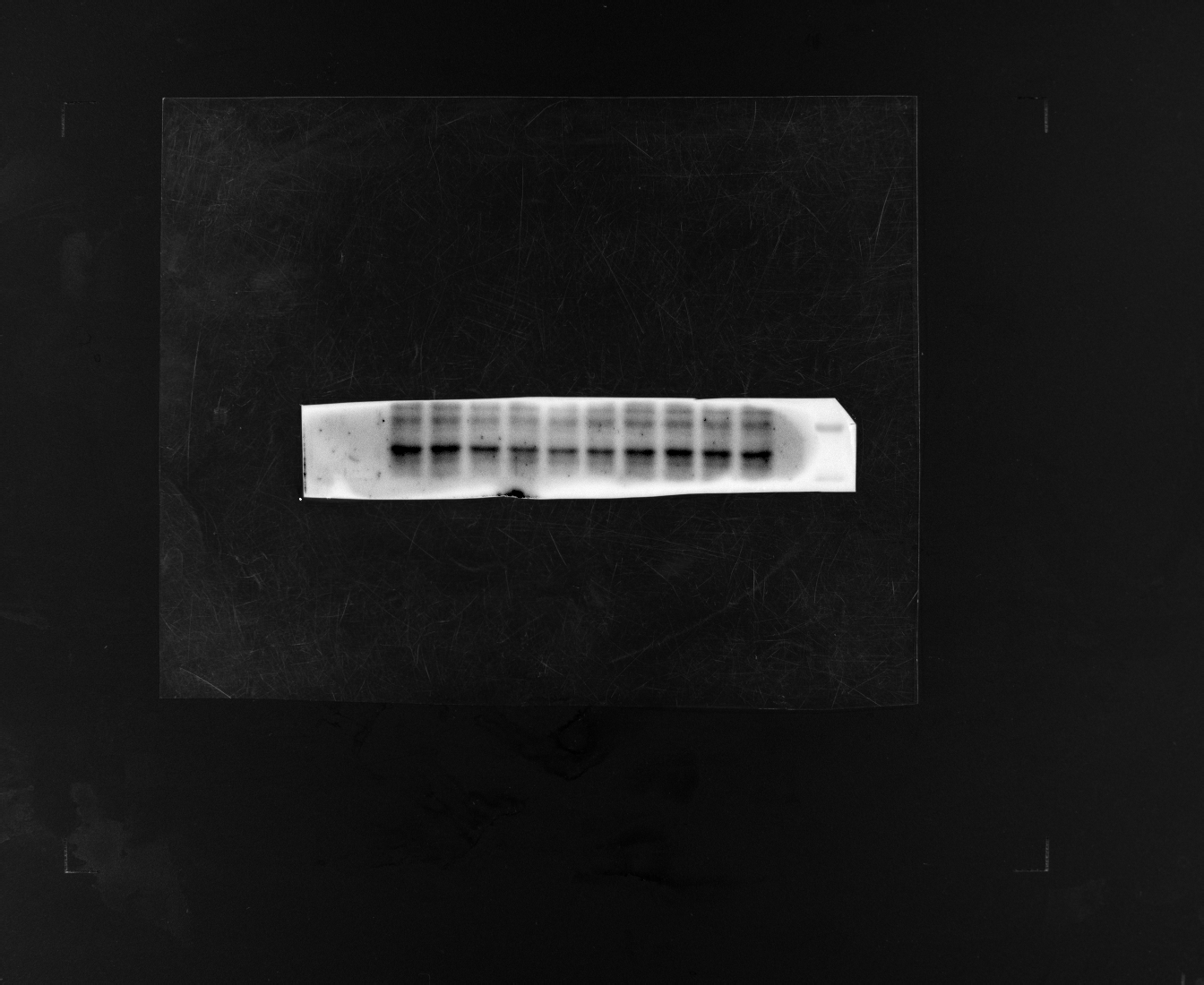

Supplement: Figure 8—source data 2. [file elife-81858-fig8-data2.zip › Figure 8-source data 2/fig8f.myog.tif]

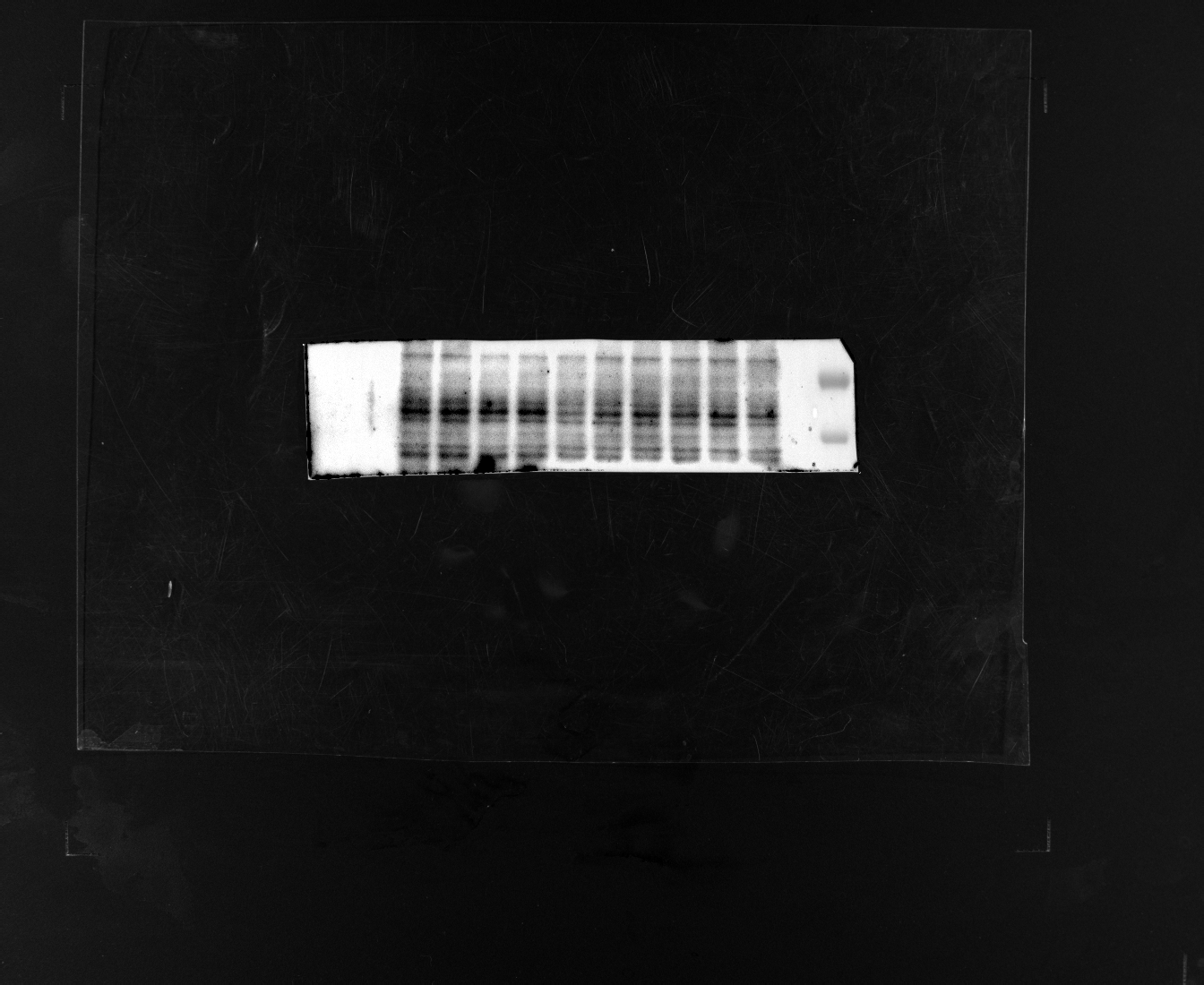

Supplement: Figure 8—source data 2. [file elife-81858-fig8-data2.zip › Figure 8-source data 2/fig8f.pakt1.tif]

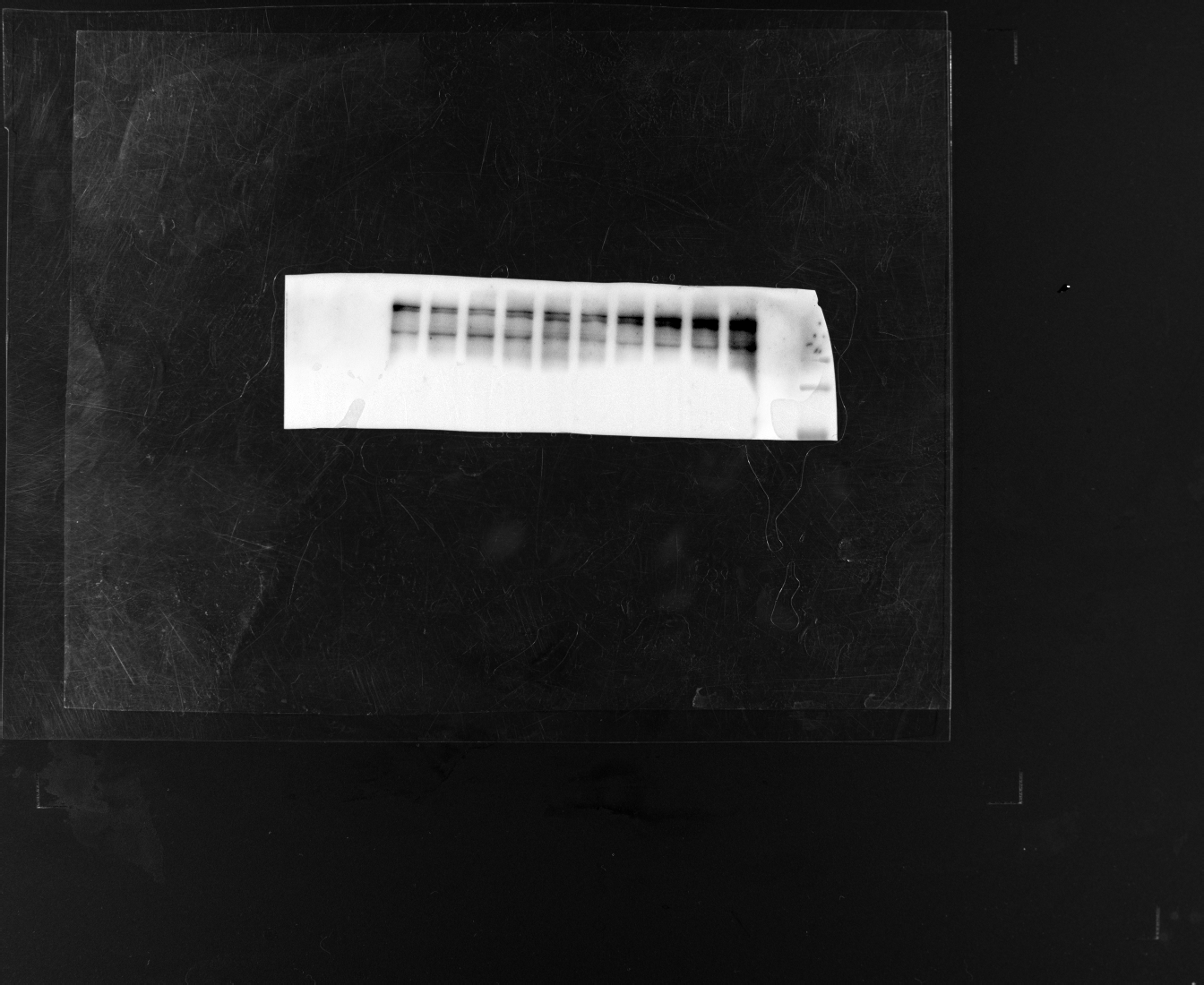

Supplement: Figure 8—source data 2. [file elife-81858-fig8-data2.zip › Figure 8-source data 2/fig8f.pmTOR.tif]

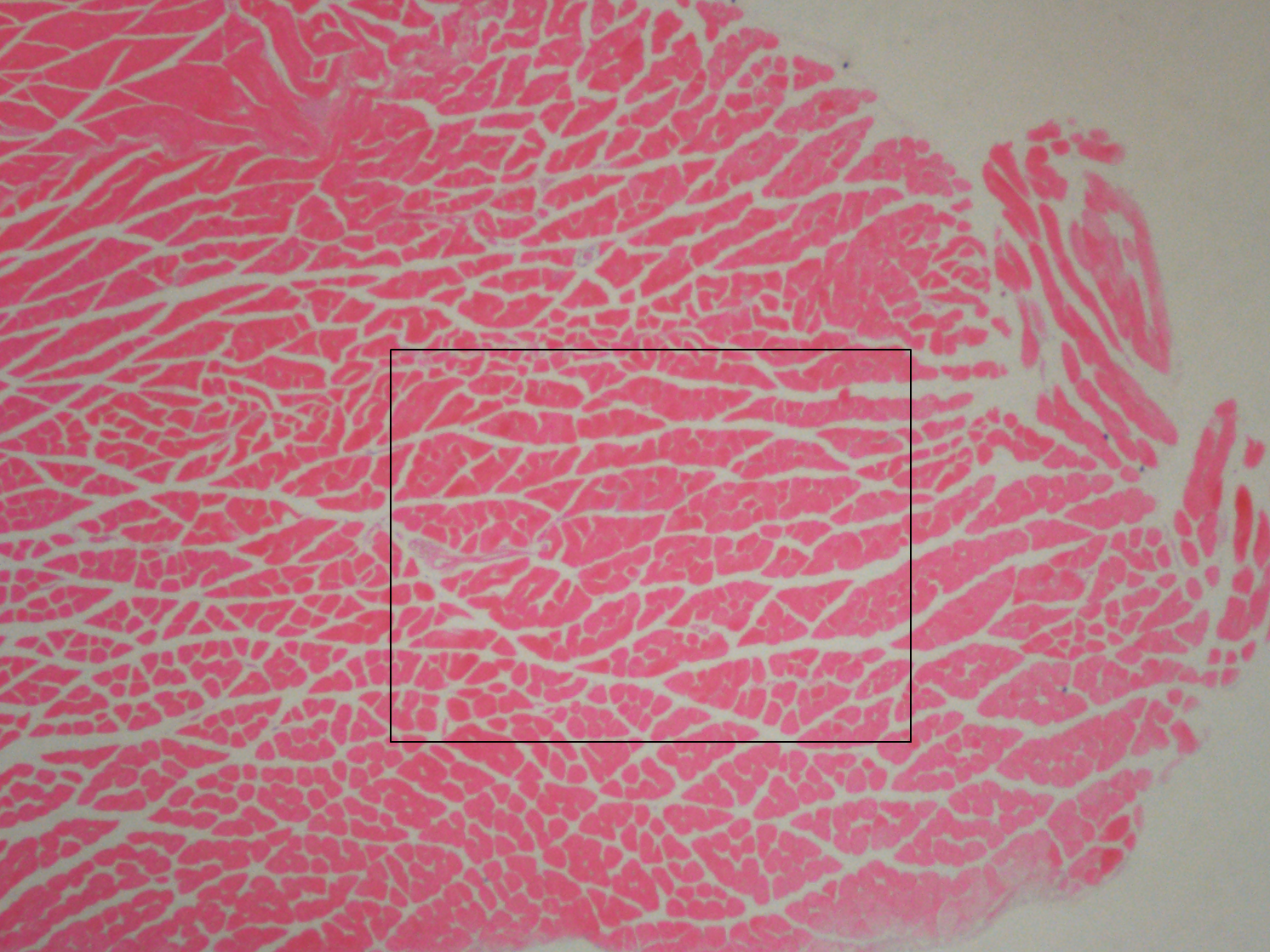

Supplement: Figure 9—source data 1. [file elife-81858-fig9-data1.zip › Figure 9-source data 1/fig9.a/Con-GA/GA-Con (1).tif]

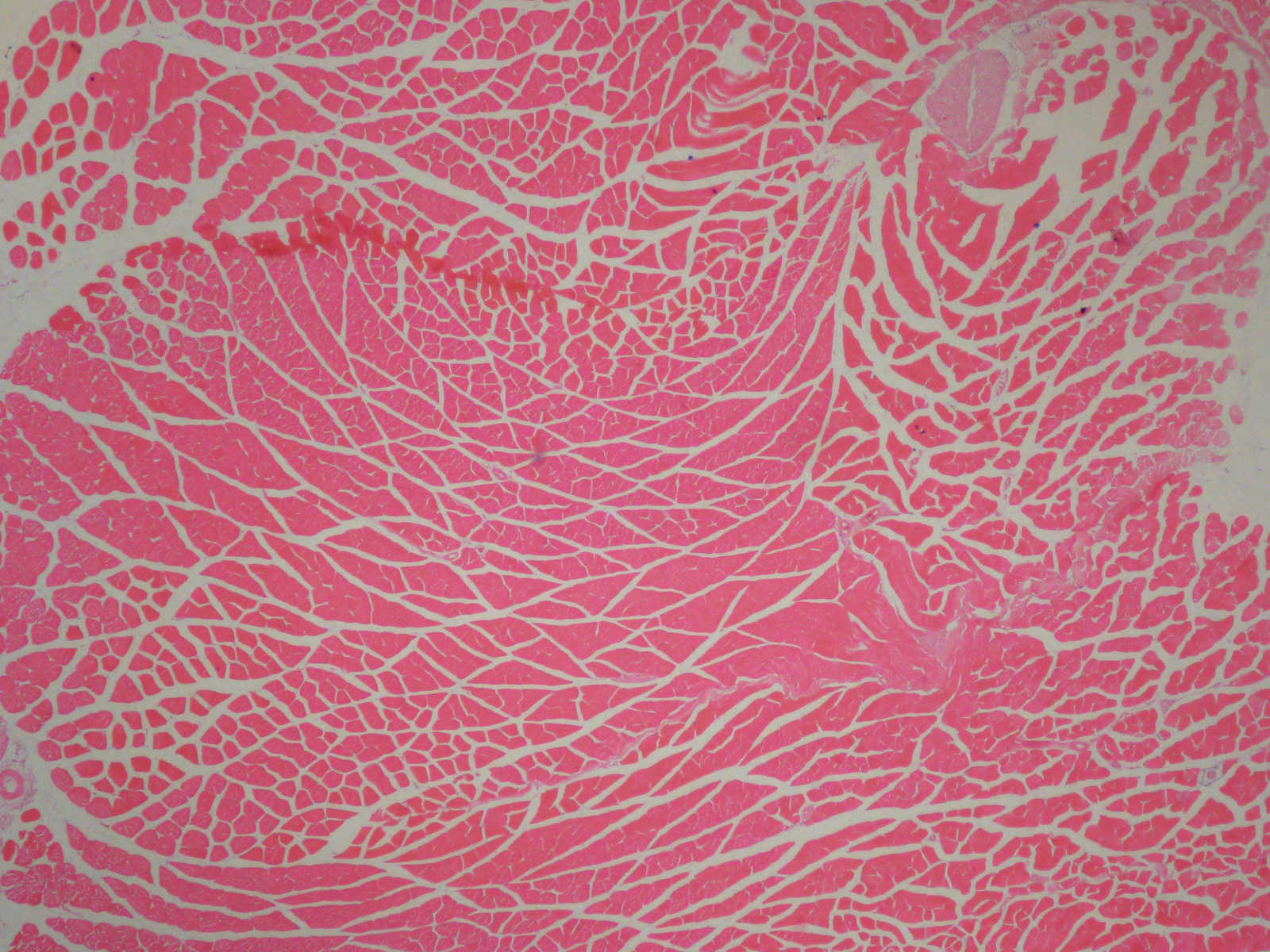

Supplement: Figure 9—source data 1. [file elife-81858-fig9-data1.zip › Figure 9-source data 1/fig9.a/Con-GA/GA-Con (2).tif]

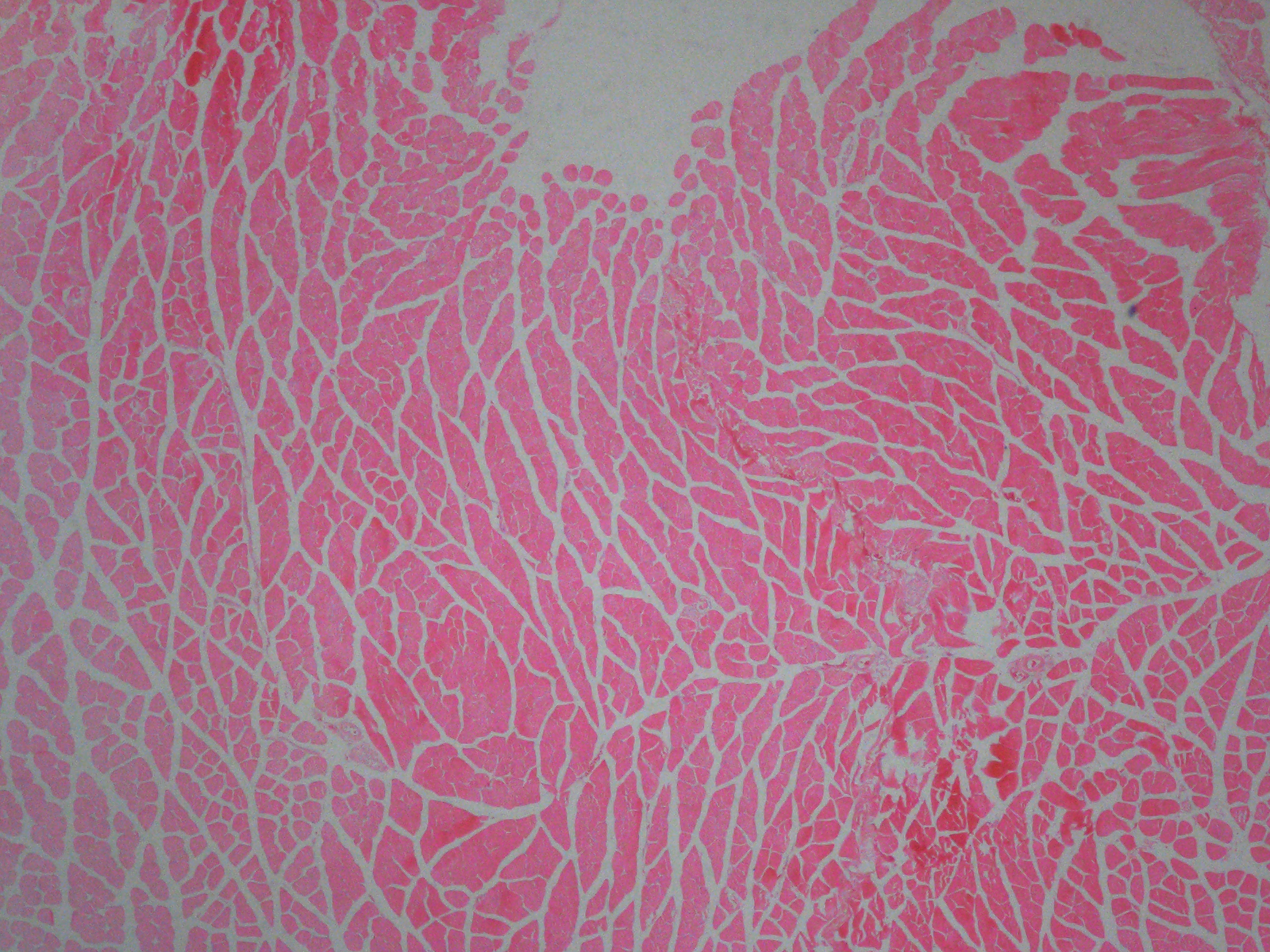

Supplement: Figure 9—source data 1. [file elife-81858-fig9-data1.zip › Figure 9-source data 1/fig9.a/Con-GA/GA-Con (3).tif]

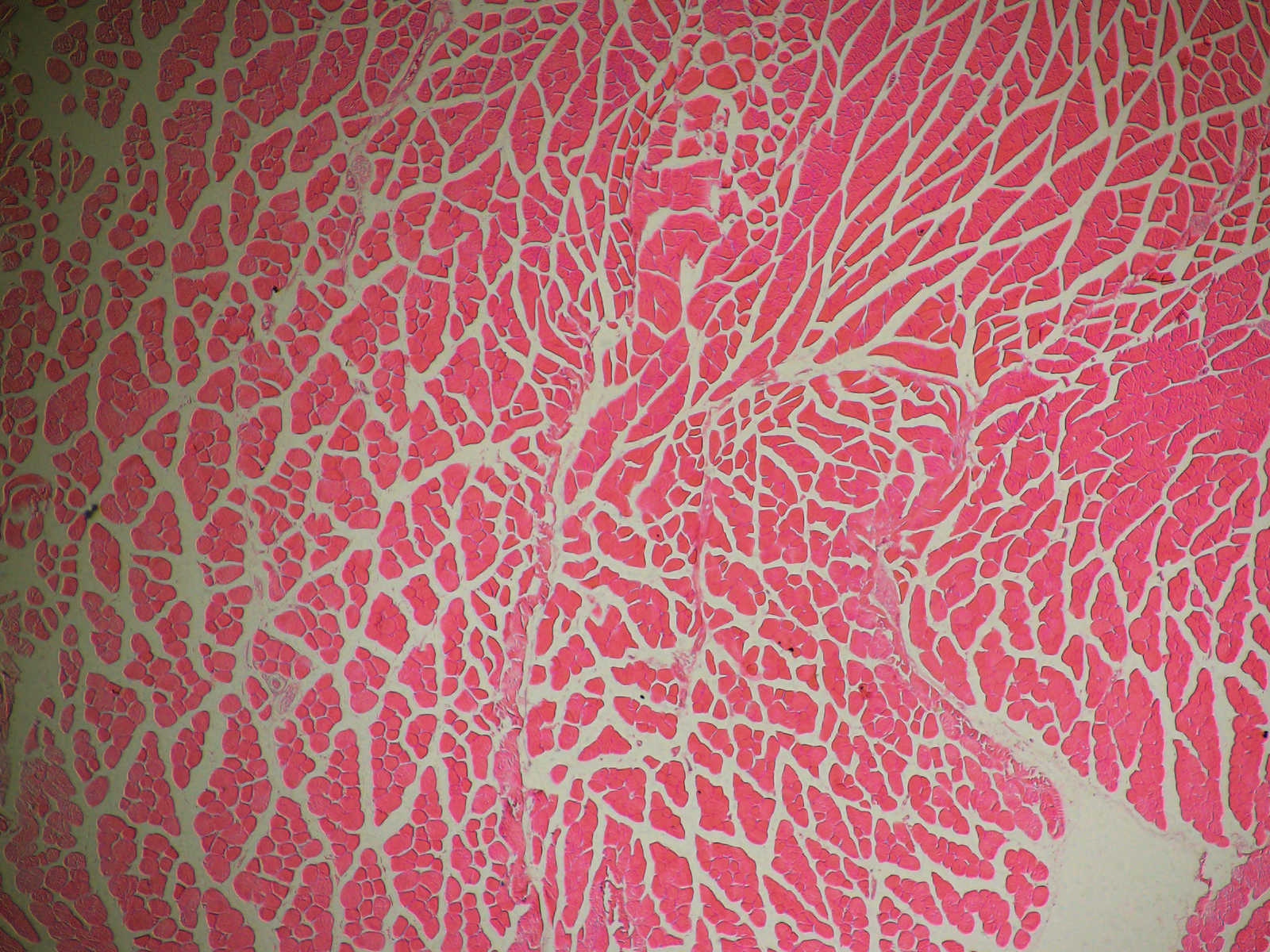

Supplement: Figure 9—source data 1. [file elife-81858-fig9-data1.zip › Figure 9-source data 1/fig9.a/Con-GA/GA-Con (4).tif]

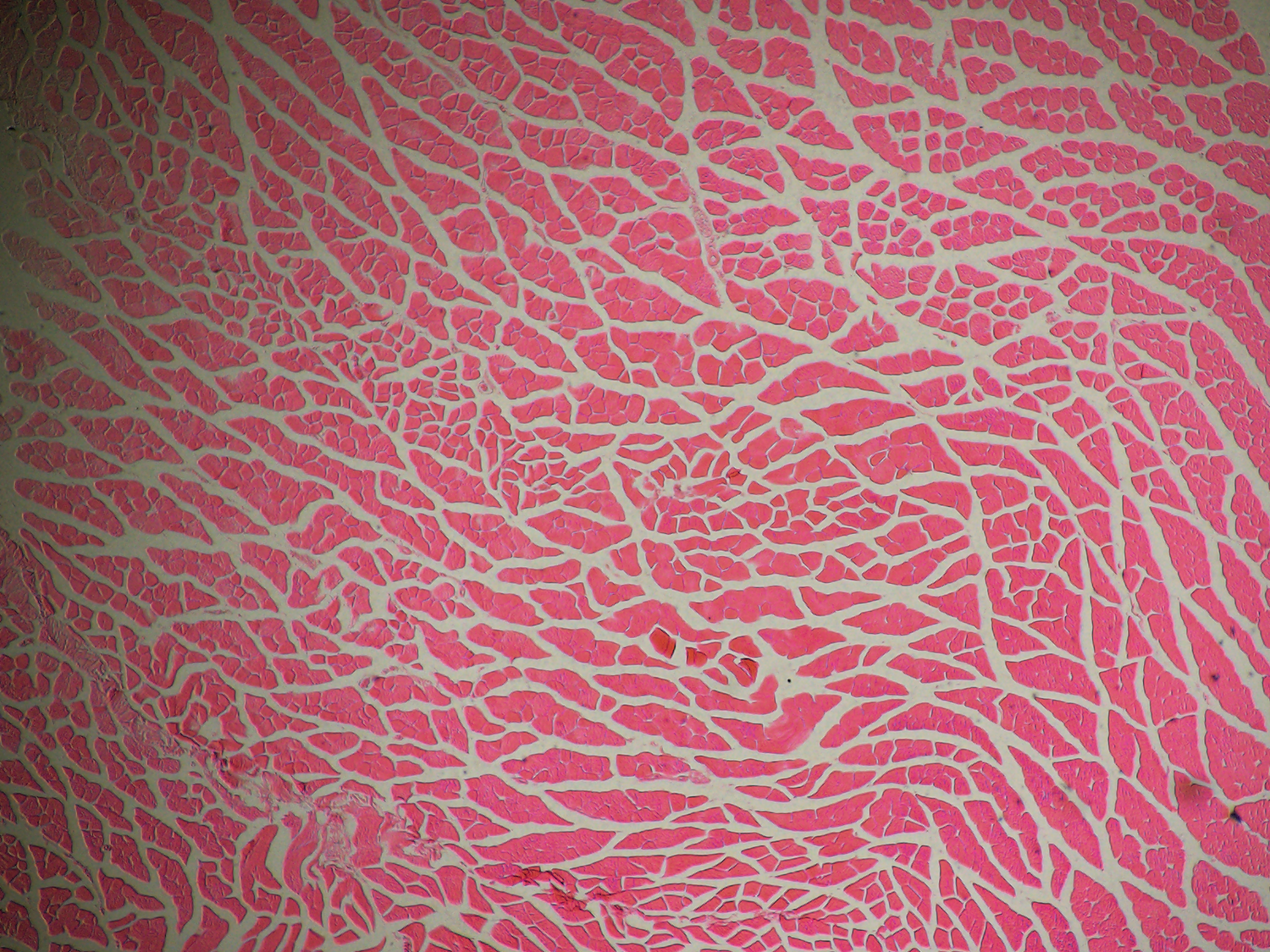

Supplement: Figure 9—source data 1. [file elife-81858-fig9-data1.zip › Figure 9-source data 1/fig9.a/Con-GA/GA-Con (5).tif]

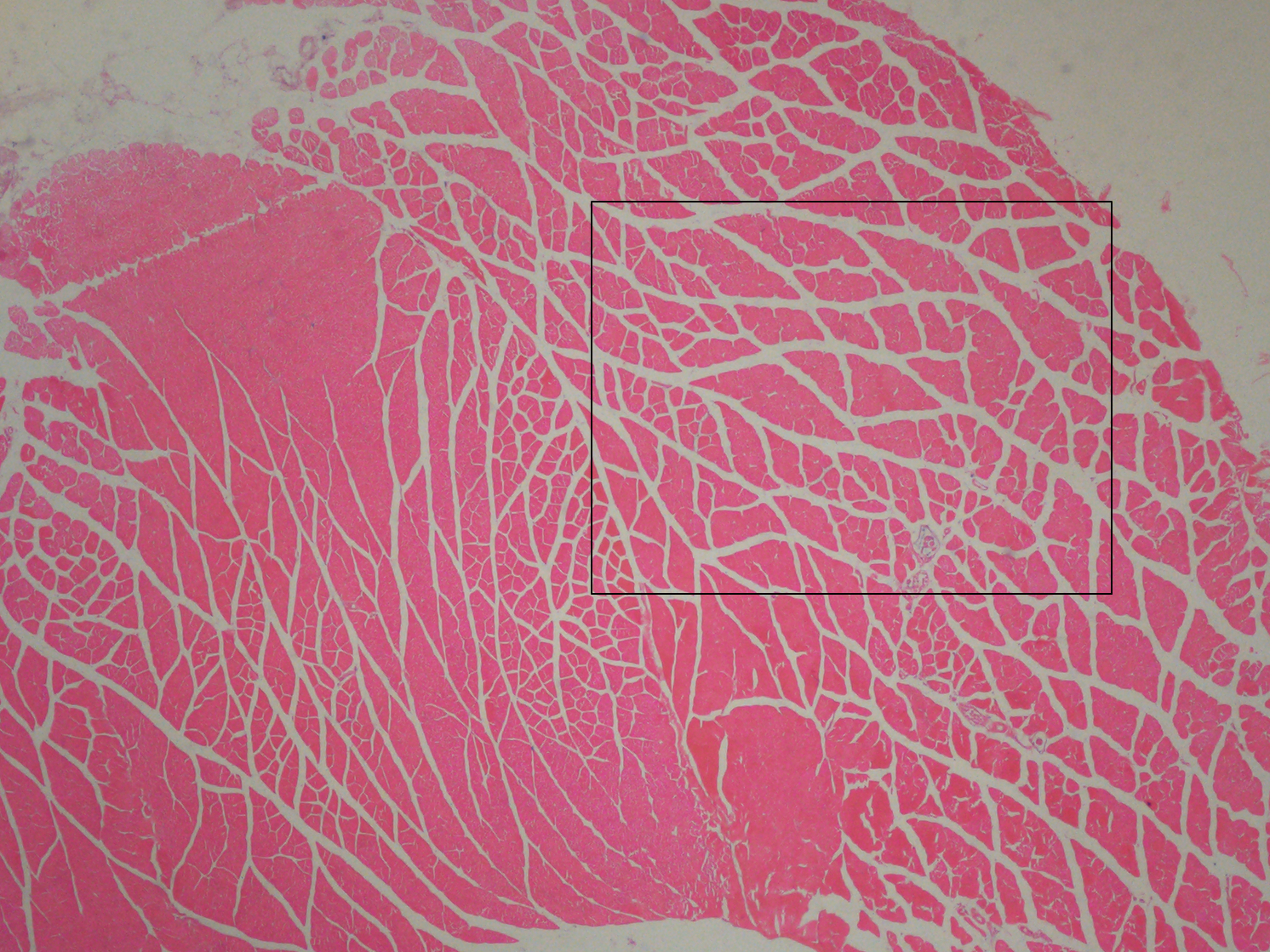

Supplement: Figure 9—source data 1. [file elife-81858-fig9-data1.zip › Figure 9-source data 1/fig9.a/Dex+Val-GA/Dex+Val-GA (1).tif]

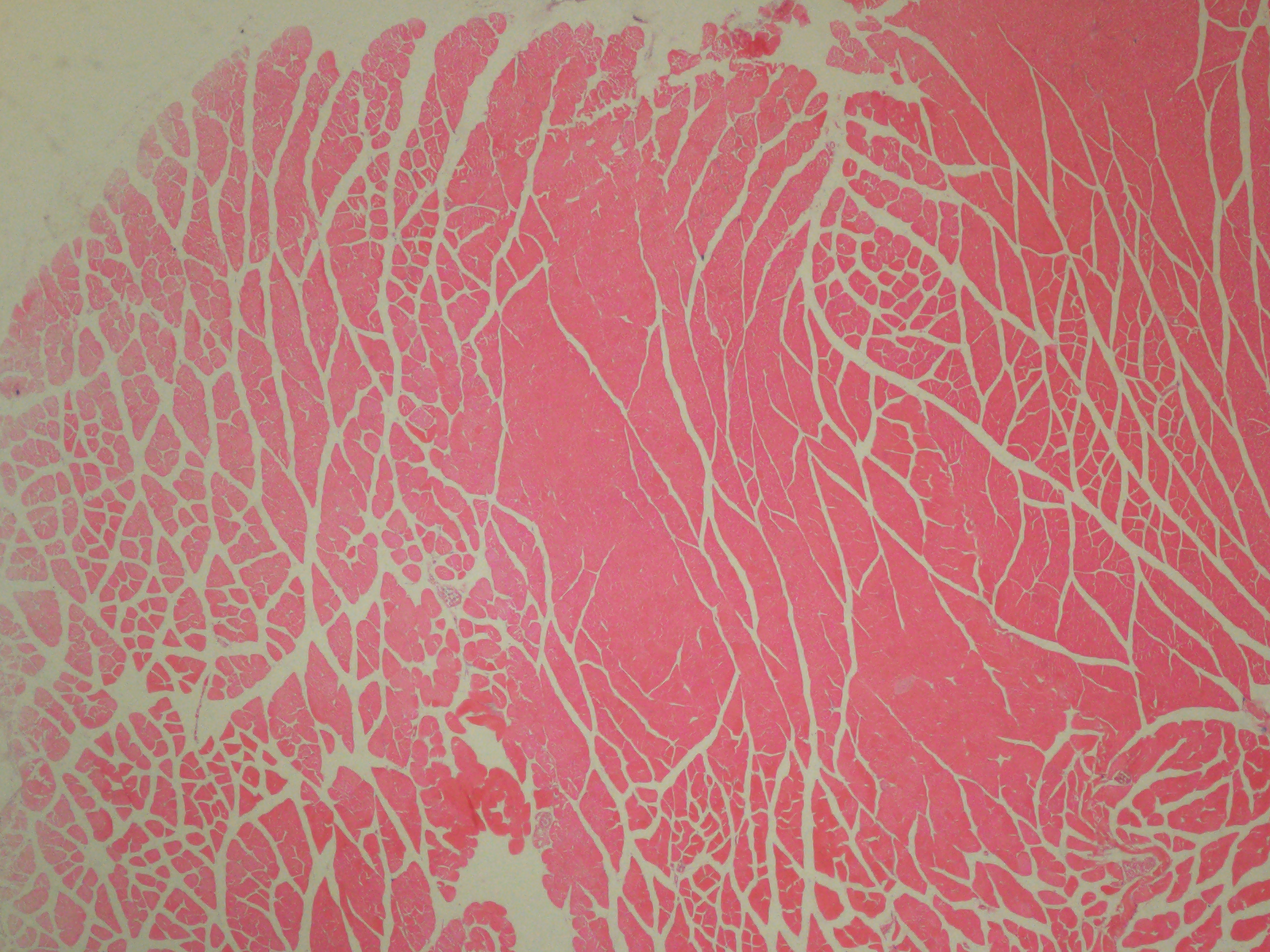

Supplement: Figure 9—source data 1. [file elife-81858-fig9-data1.zip › Figure 9-source data 1/fig9.a/Dex+Val-GA/Dex+Val-GA (2).tif]

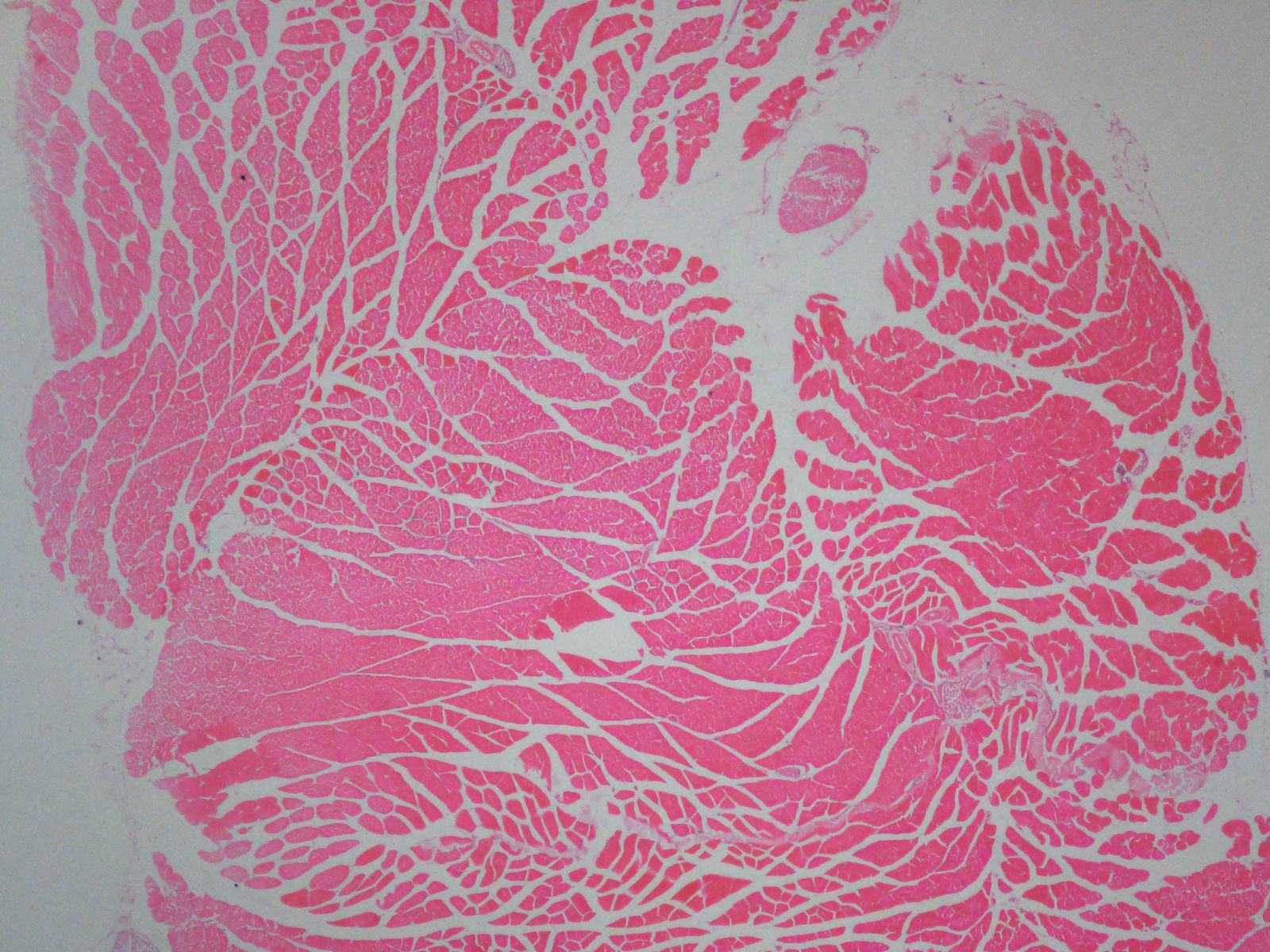

Supplement: Figure 9—source data 1. [file elife-81858-fig9-data1.zip › Figure 9-source data 1/fig9.a/Dex+Val-GA/Dex+Val-GA (3).tif]

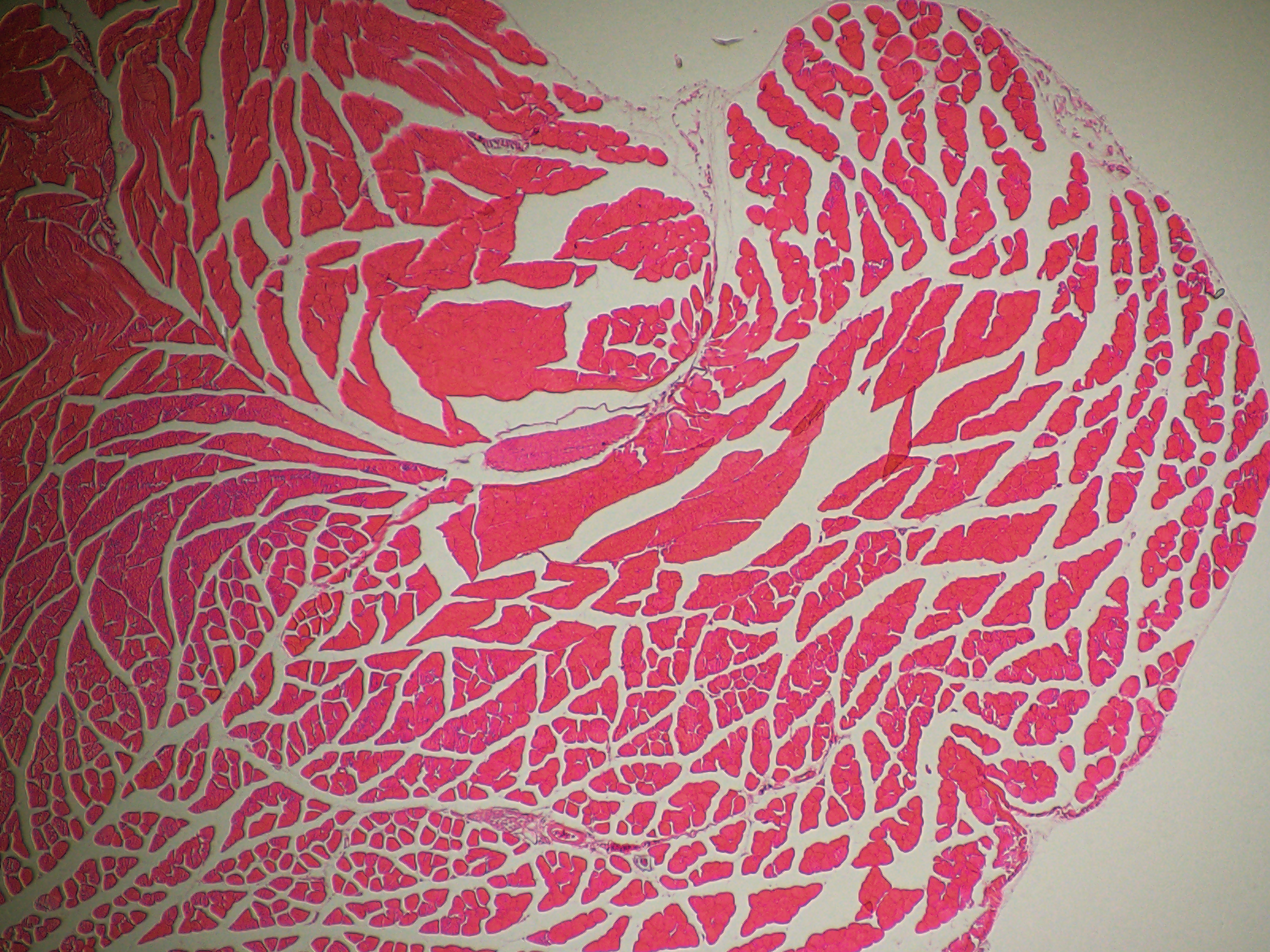

Supplement: Figure 9—source data 1. [file elife-81858-fig9-data1.zip › Figure 9-source data 1/fig9.a/Dex+Val-GA/Dex+Val-GA (4).tif]

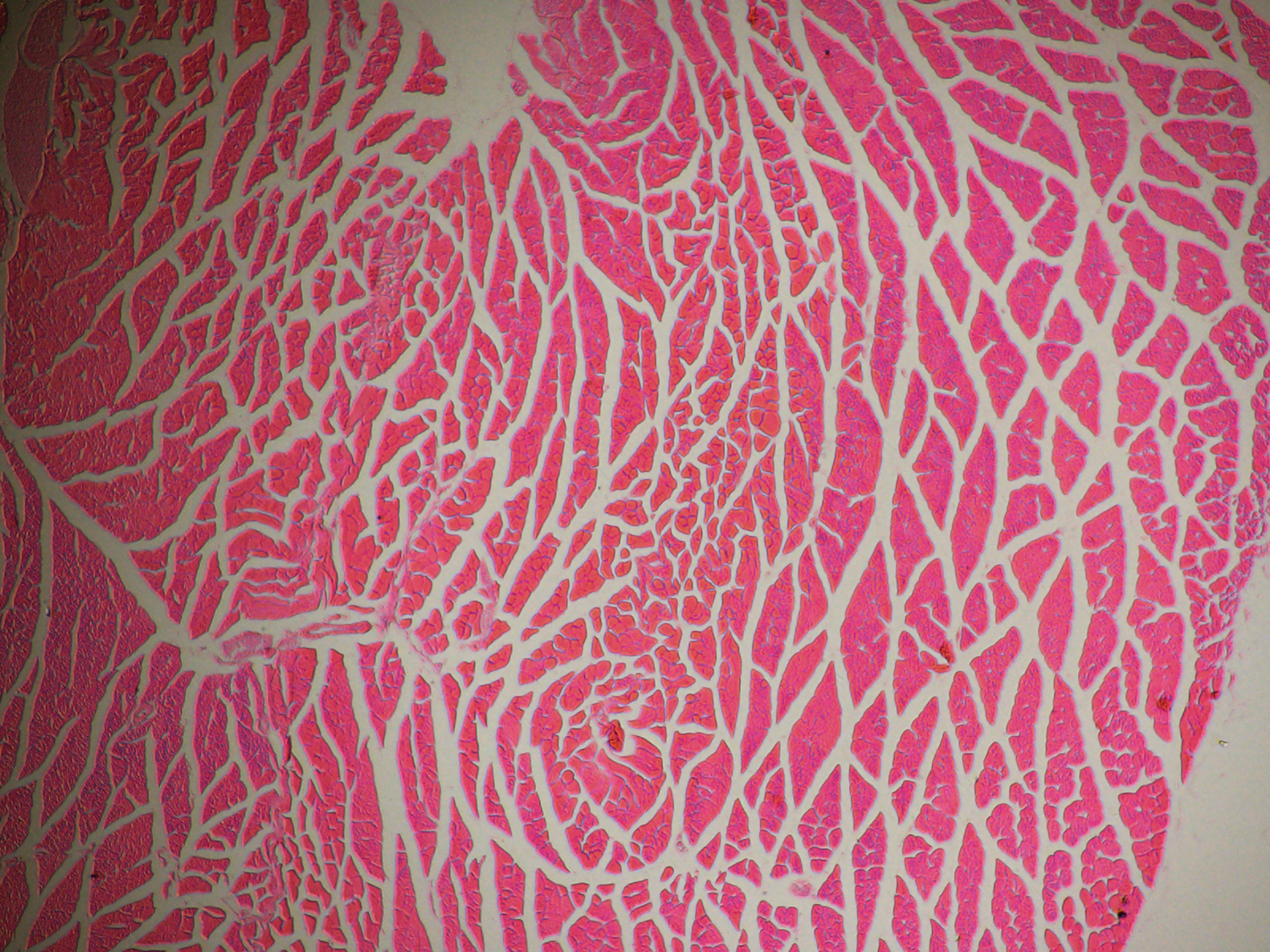

Supplement: Figure 9—source data 1. [file elife-81858-fig9-data1.zip › Figure 9-source data 1/fig9.a/Dex+Val-GA/Dex+Val-GA (5).tif]

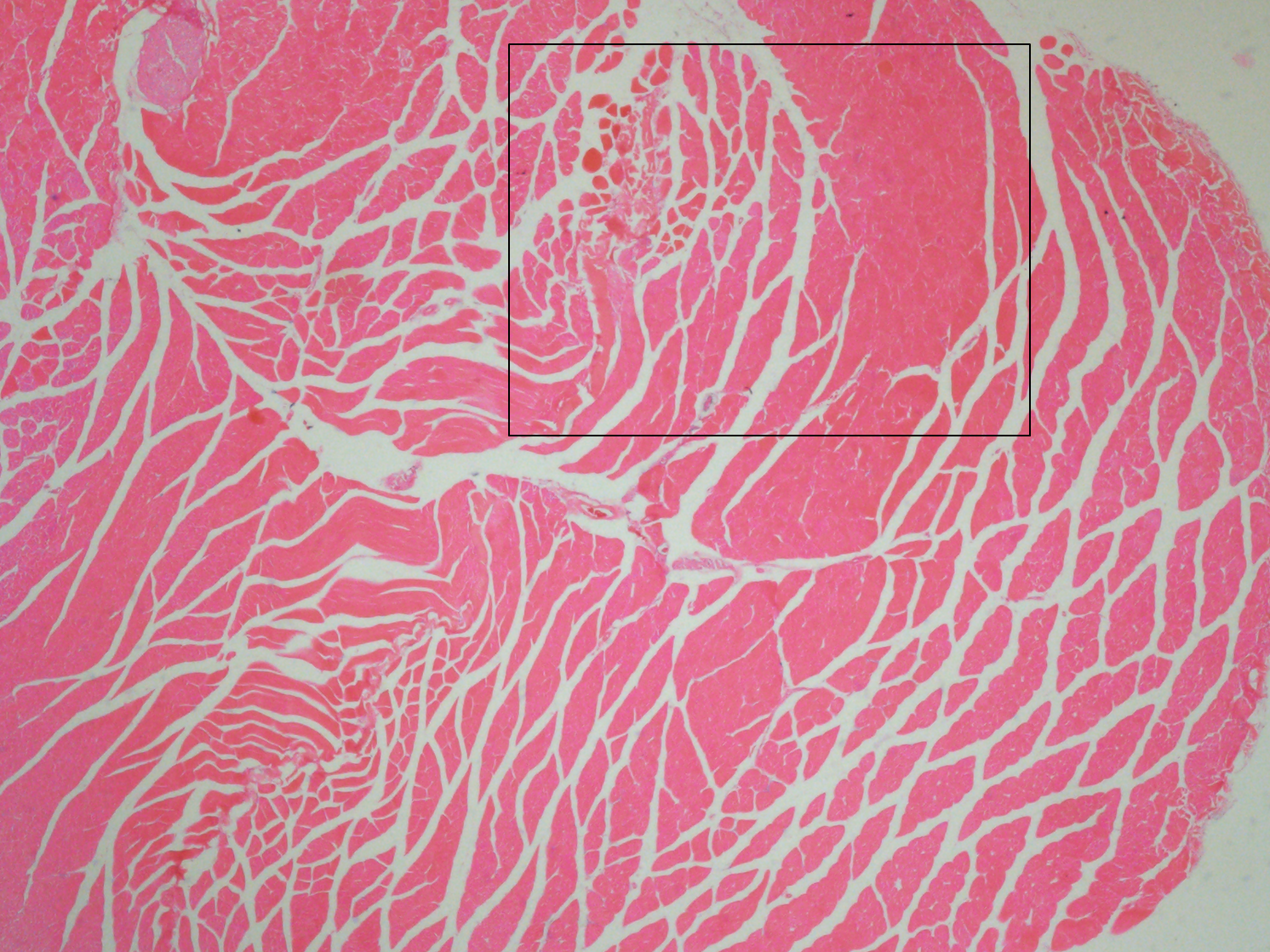

Supplement: Figure 9—source data 1. [file elife-81858-fig9-data1.zip › Figure 9-source data 1/fig9.a/Dex-GA/Dex-GA (1).tif]

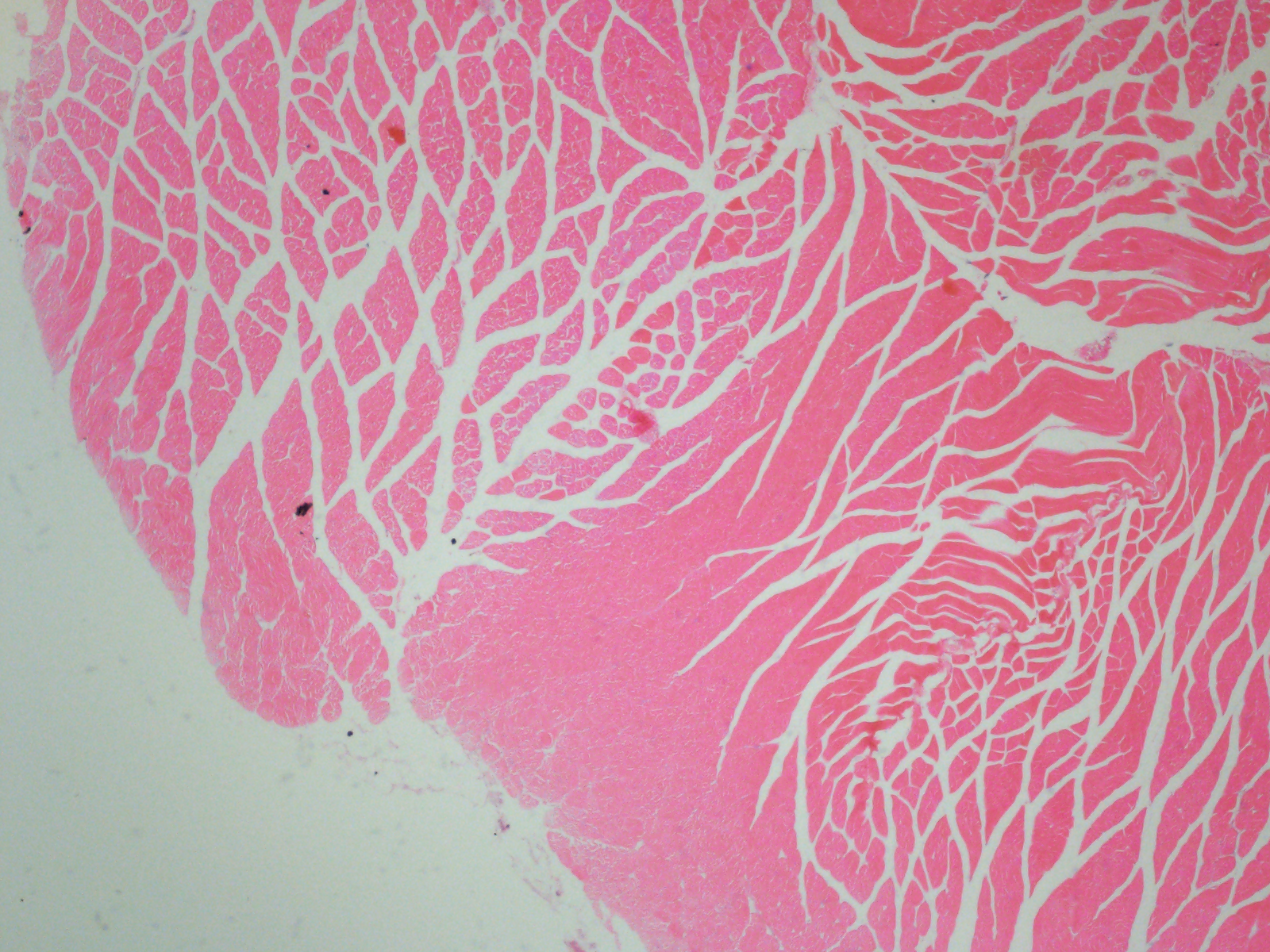

Supplement: Figure 9—source data 1. [file elife-81858-fig9-data1.zip › Figure 9-source data 1/fig9.a/Dex-GA/Dex-GA (2).tif]

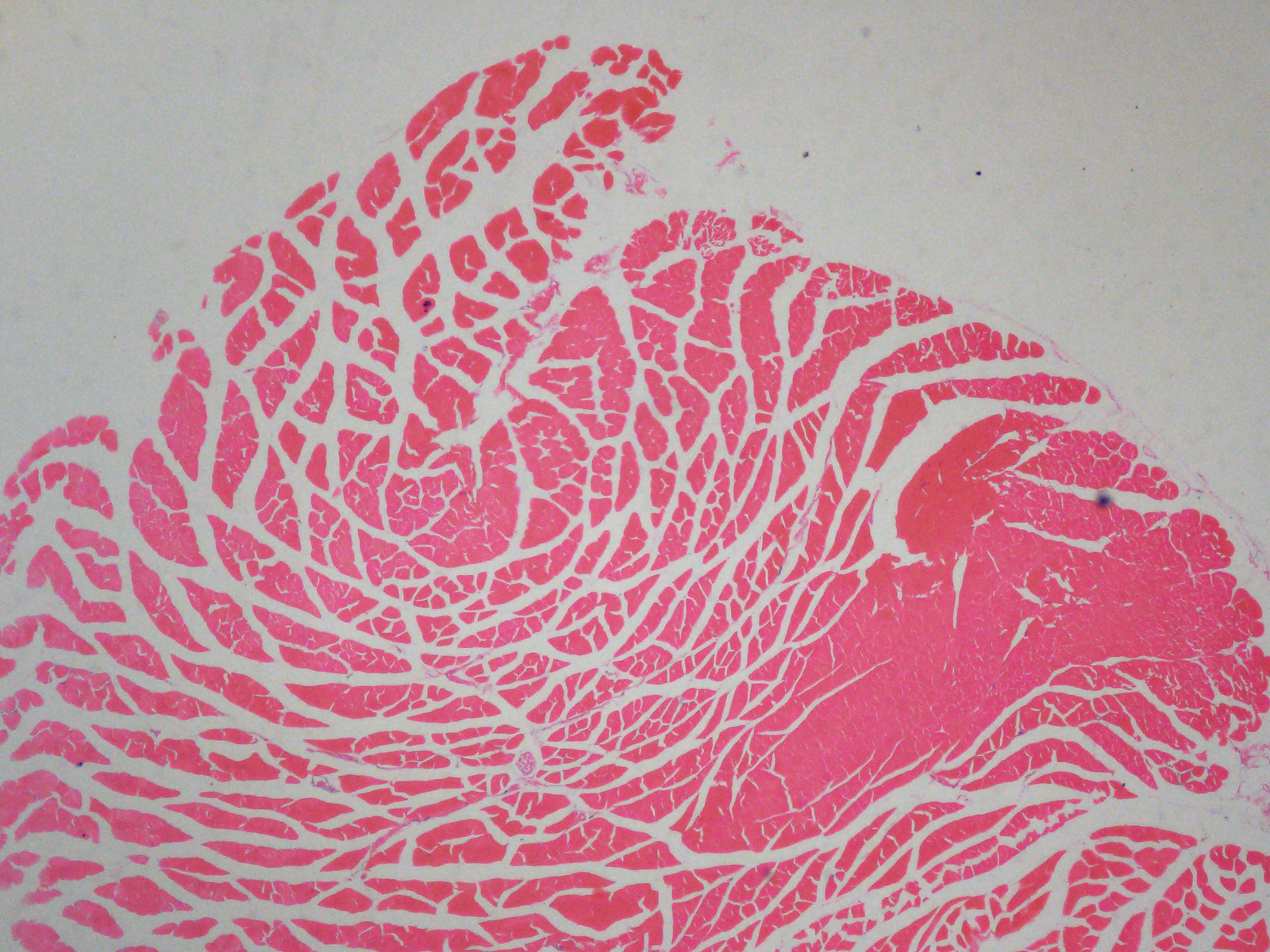

Supplement: Figure 9—source data 1. [file elife-81858-fig9-data1.zip › Figure 9-source data 1/fig9.a/Dex-GA/Dex-GA (3).tif]

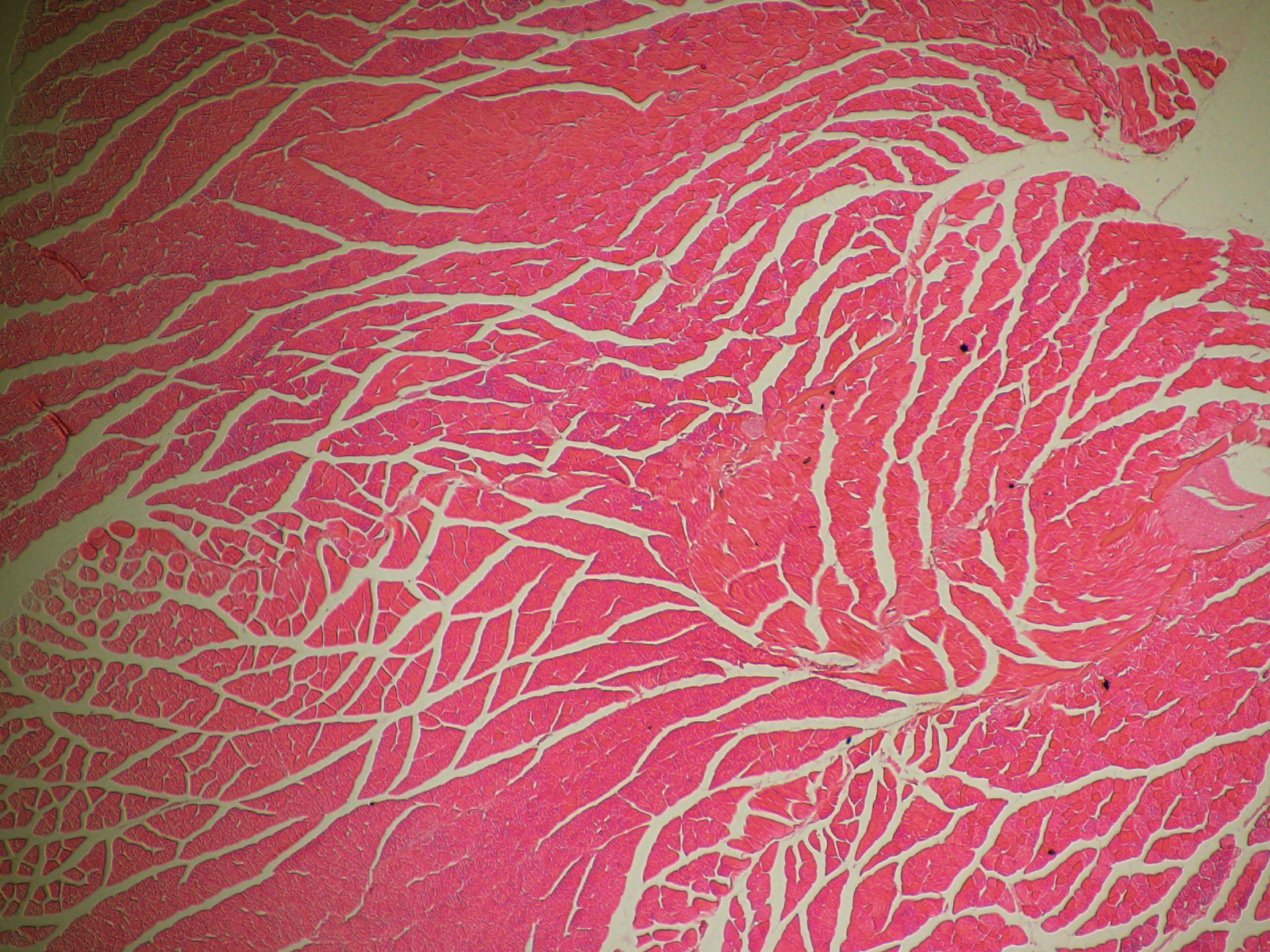

Supplement: Figure 9—source data 1. [file elife-81858-fig9-data1.zip › Figure 9-source data 1/fig9.a/Dex-GA/Dex-GA (4).tif]

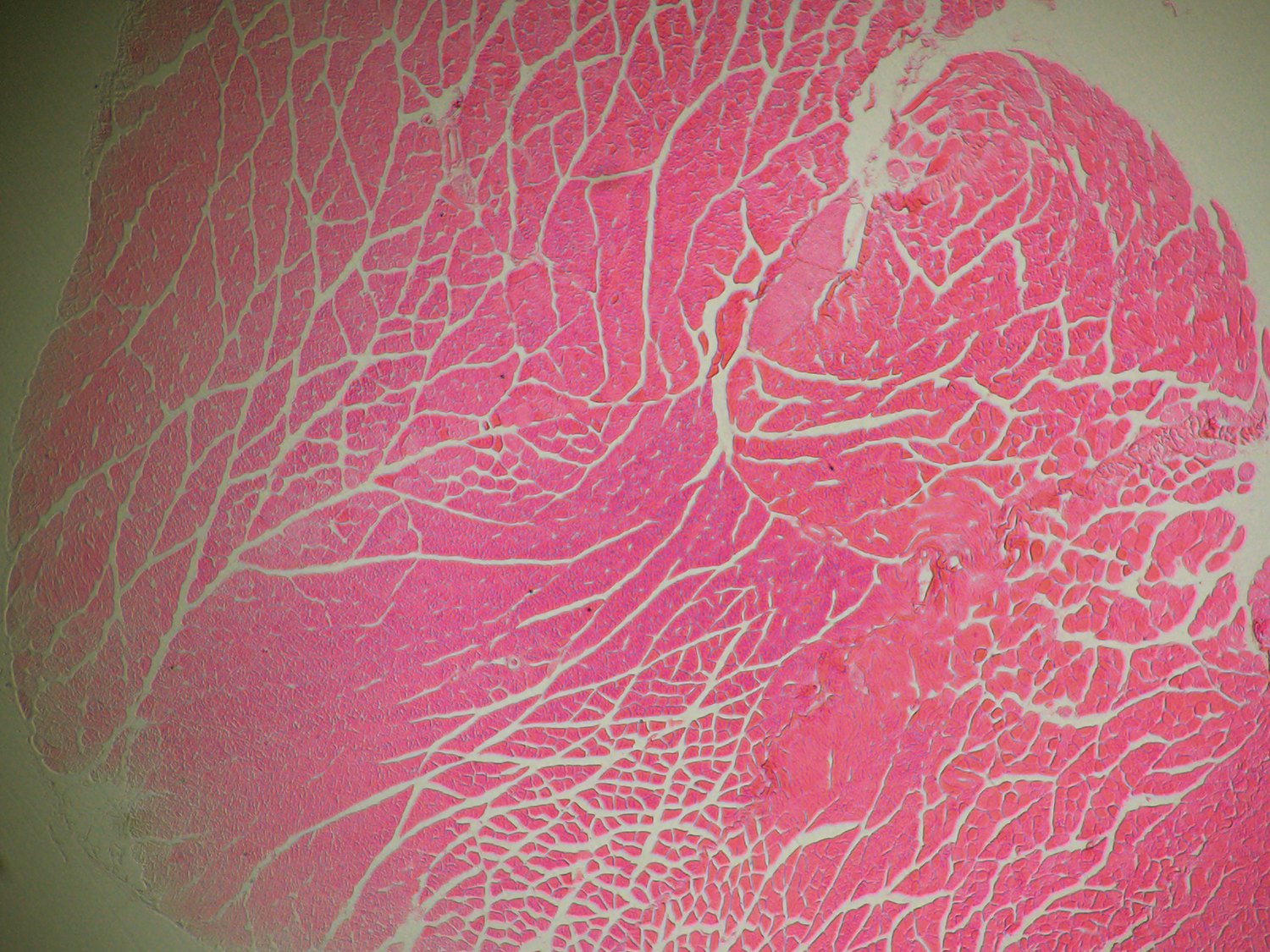

Supplement: Figure 9—source data 1. [file elife-81858-fig9-data1.zip › Figure 9-source data 1/fig9.a/Dex-GA/Dex-GA (5).tif]

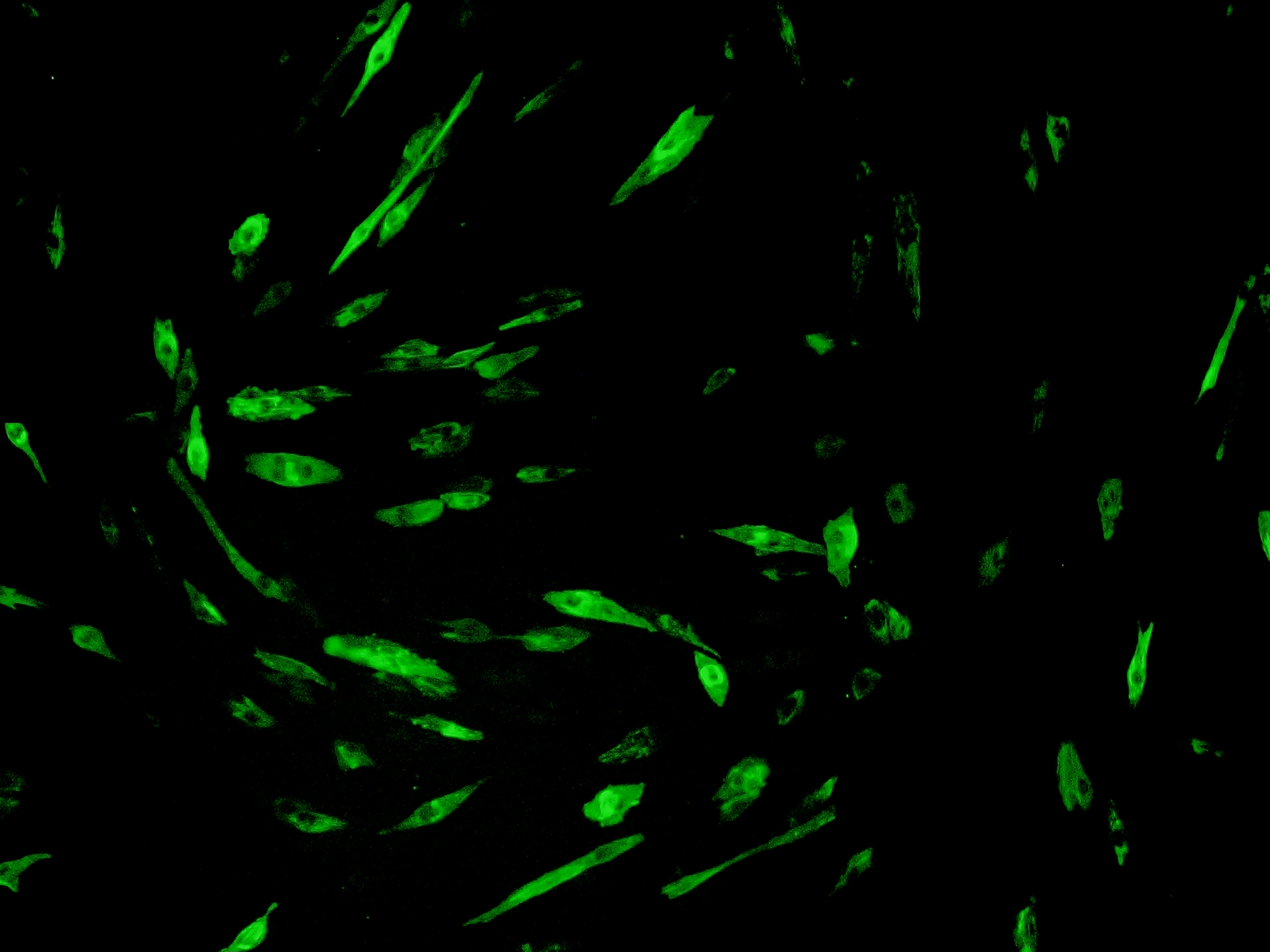

Supplement: Figure 9—source data 2. [file elife-81858-fig9-data2.zip › Figure 9-source data 2/fig9.d/Control/Con (1).tif]

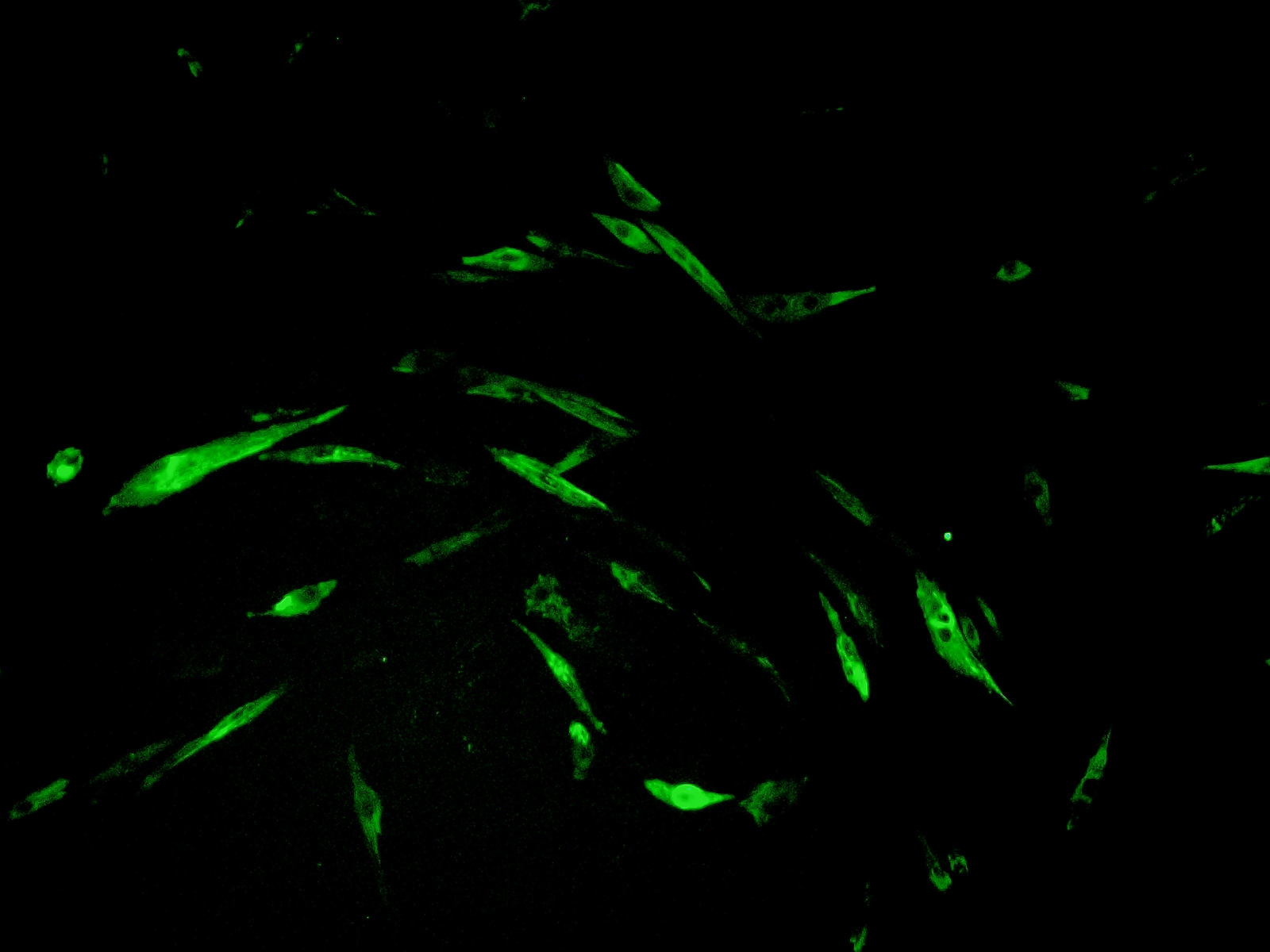

Supplement: Figure 9—source data 2. [file elife-81858-fig9-data2.zip › Figure 9-source data 2/fig9.d/Control/Con (2).tif]

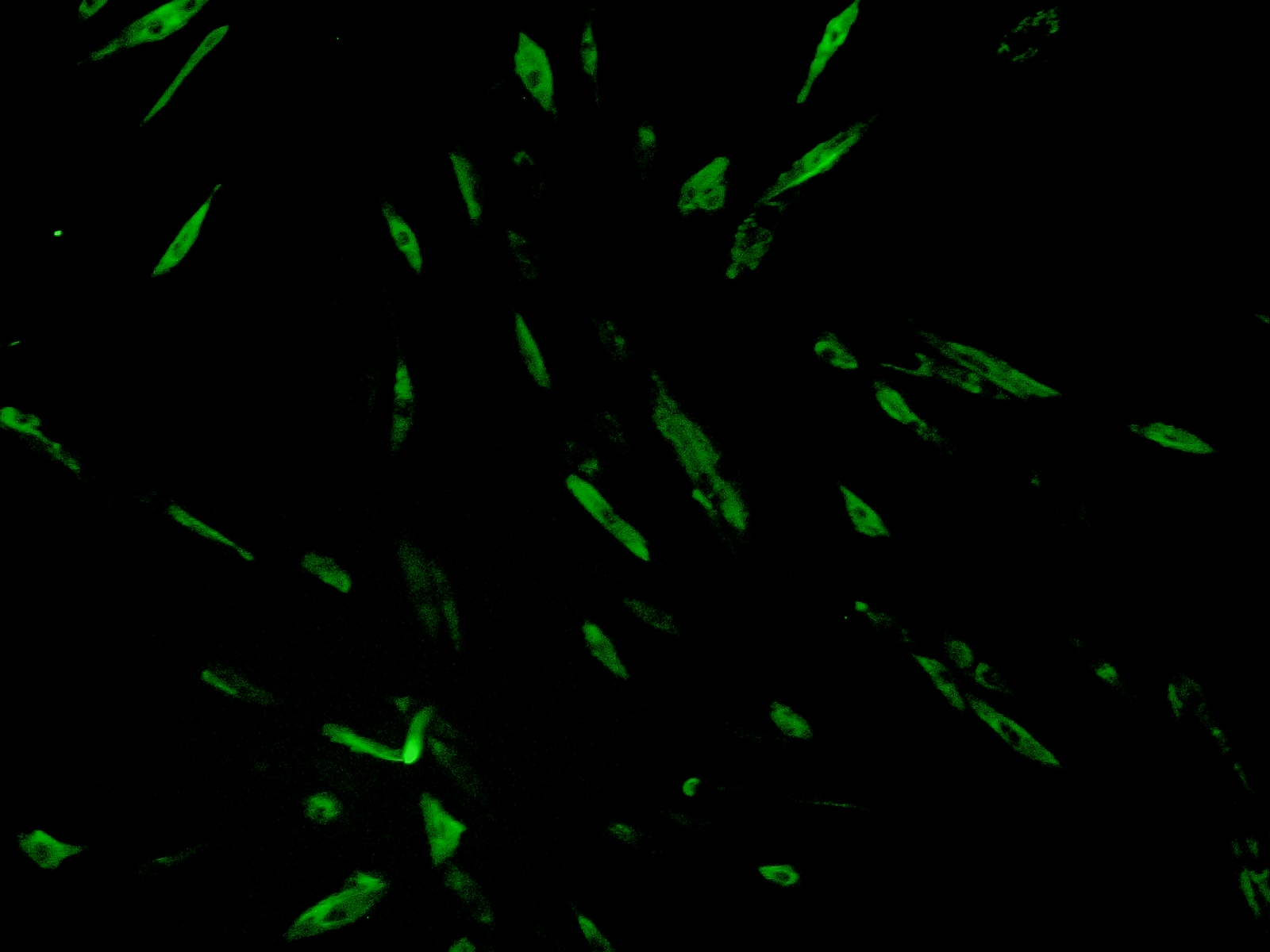

Supplement: Figure 9—source data 2. [file elife-81858-fig9-data2.zip › Figure 9-source data 2/fig9.d/Control/Con (3).tif]

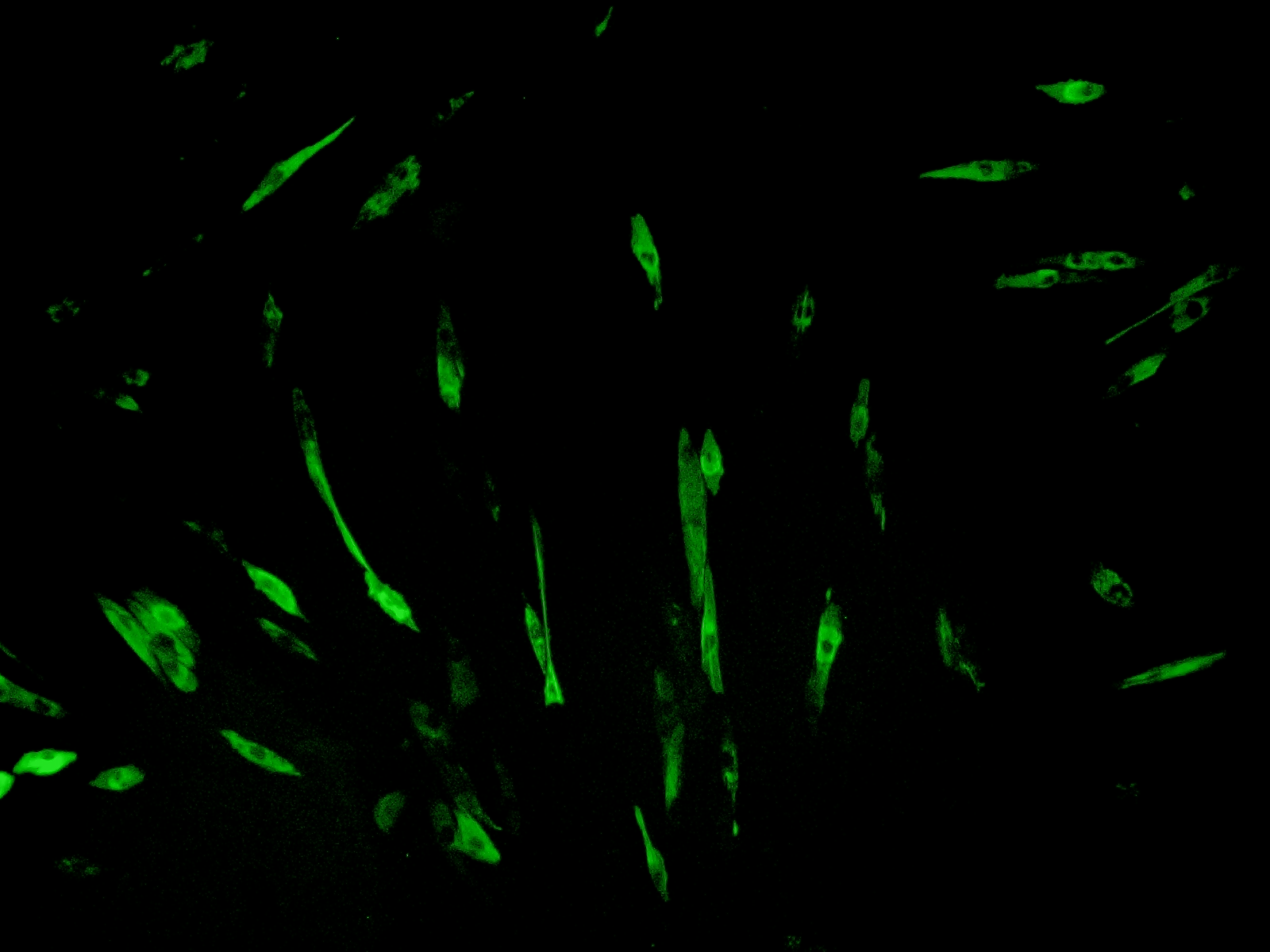

Supplement: Figure 9—source data 2. [file elife-81858-fig9-data2.zip › Figure 9-source data 2/fig9.d/Control/Con (4).tif]

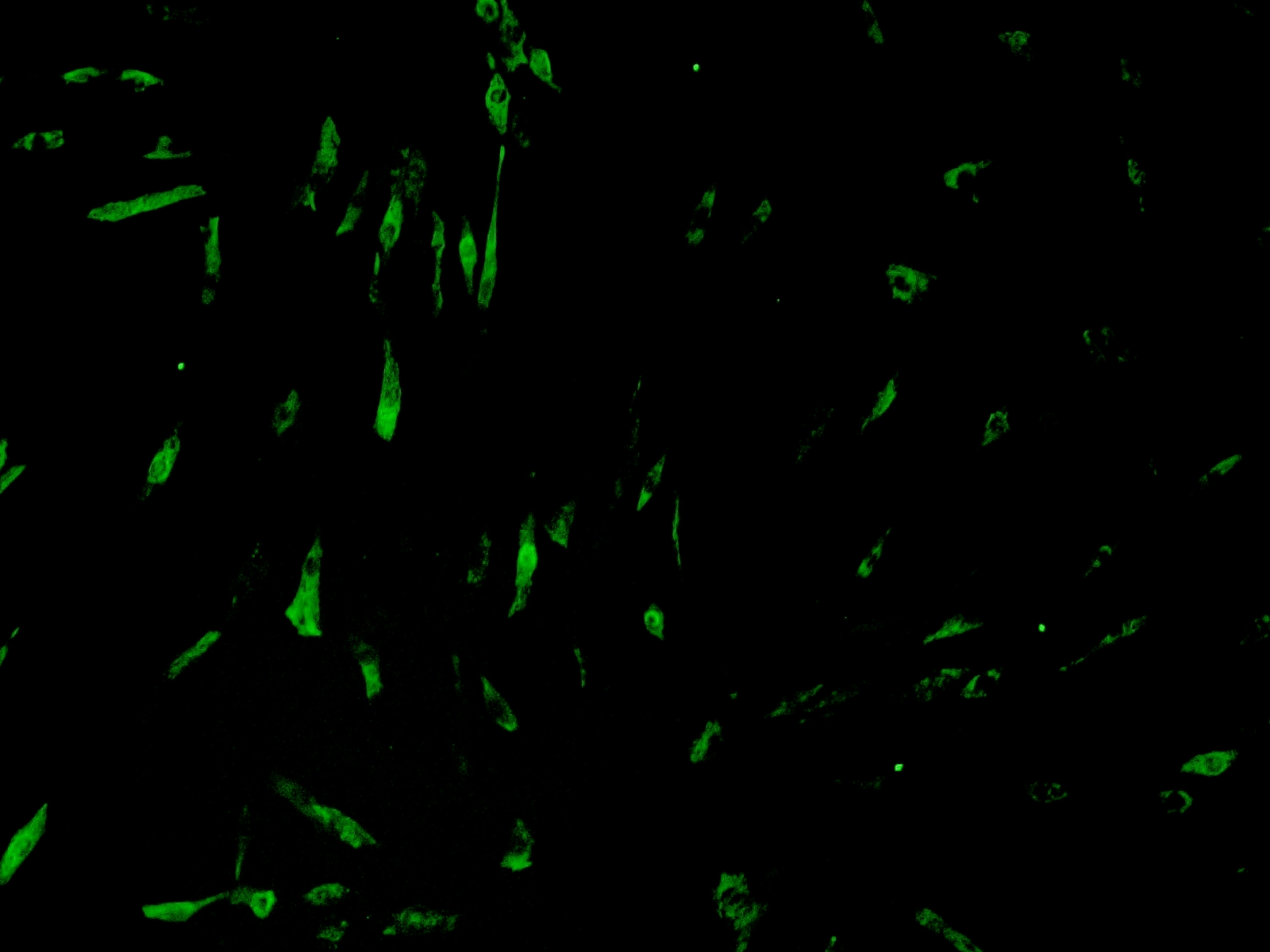

Supplement: Figure 9—source data 2. [file elife-81858-fig9-data2.zip › Figure 9-source data 2/fig9.d/Dex/Dex (1).tif]

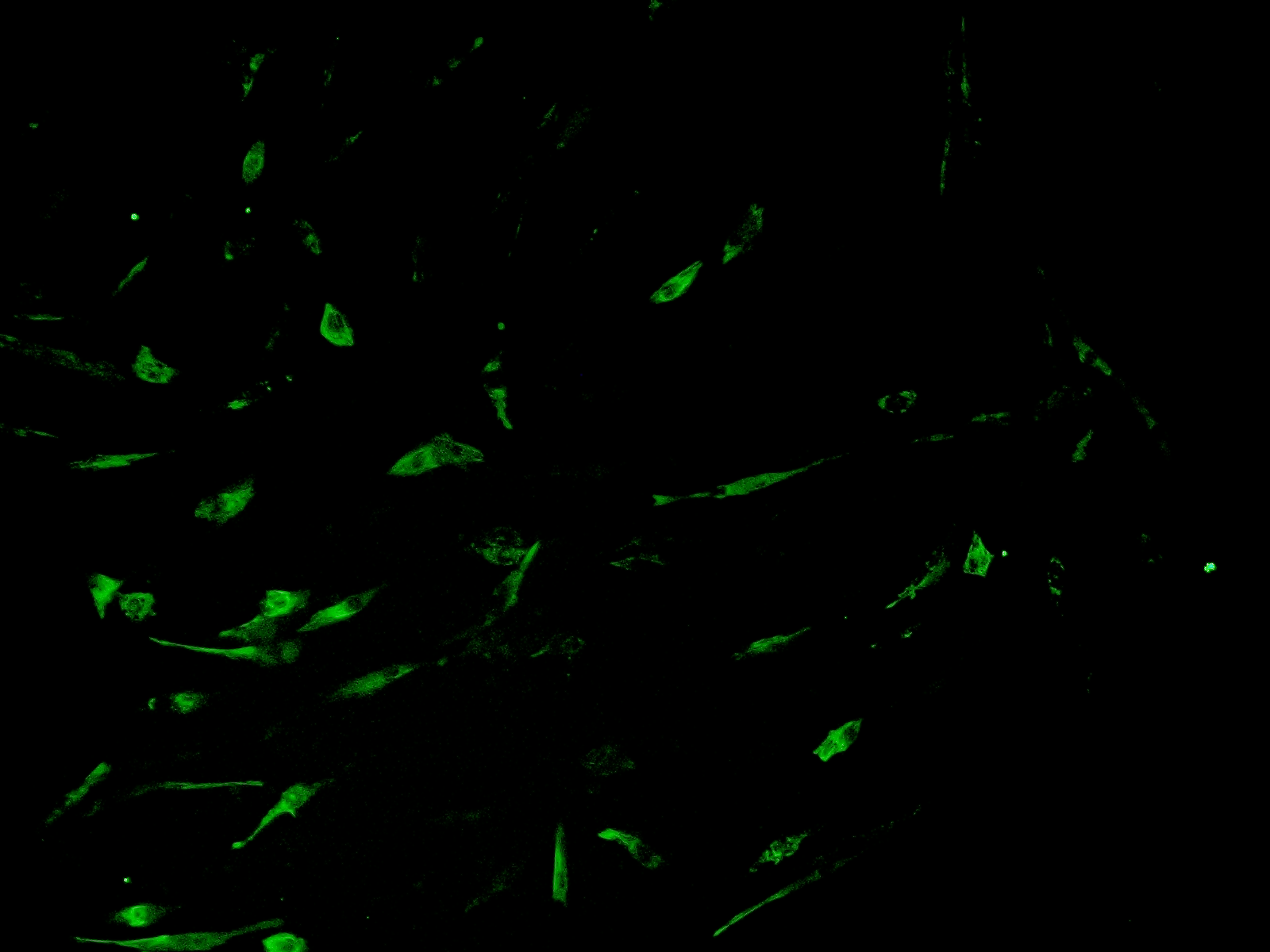

Supplement: Figure 9—source data 2. [file elife-81858-fig9-data2.zip › Figure 9-source data 2/fig9.d/Dex/Dex (2).tif]

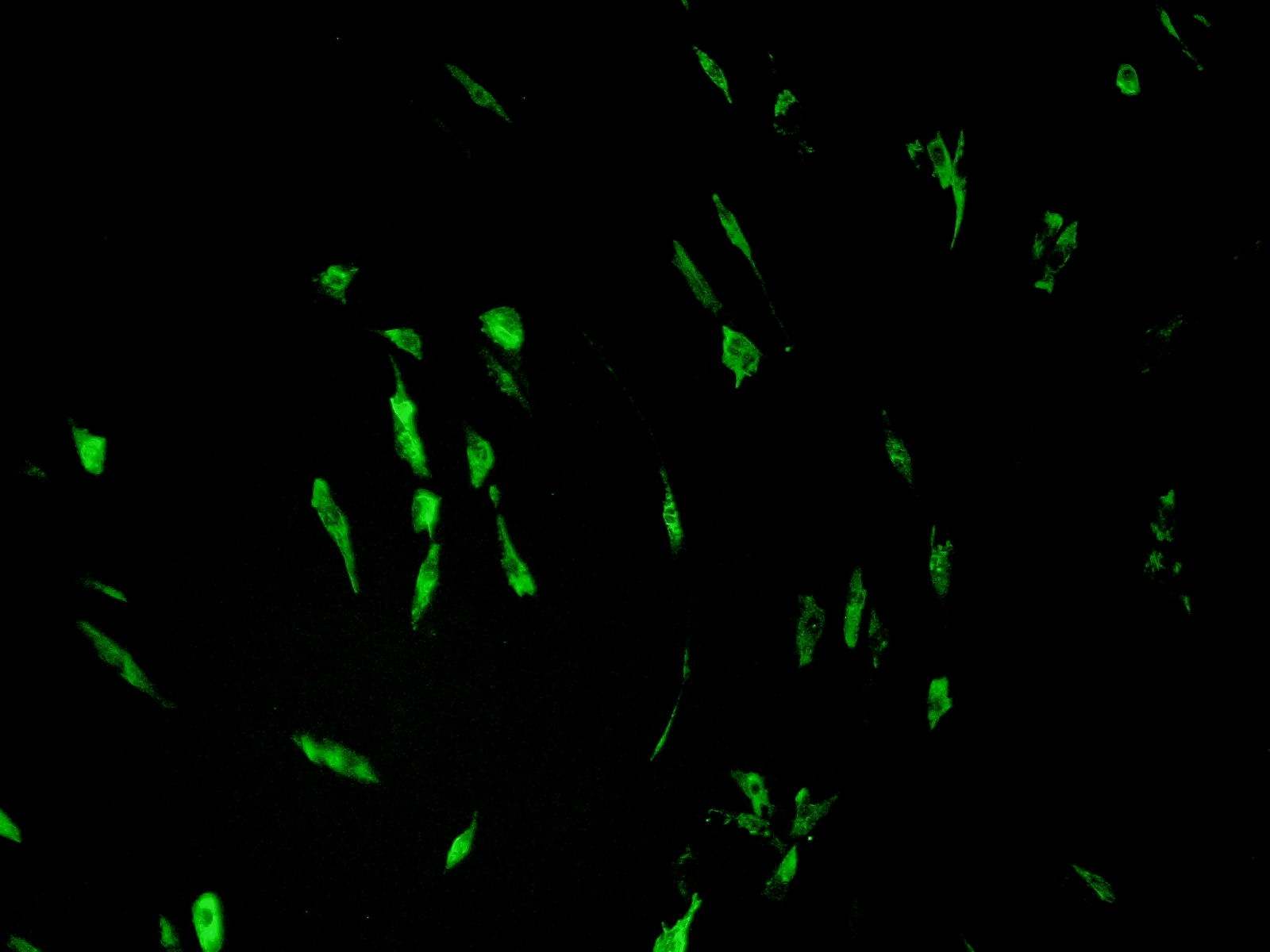

Supplement: Figure 9—source data 2. [file elife-81858-fig9-data2.zip › Figure 9-source data 2/fig9.d/Dex/Dex (3).tif]

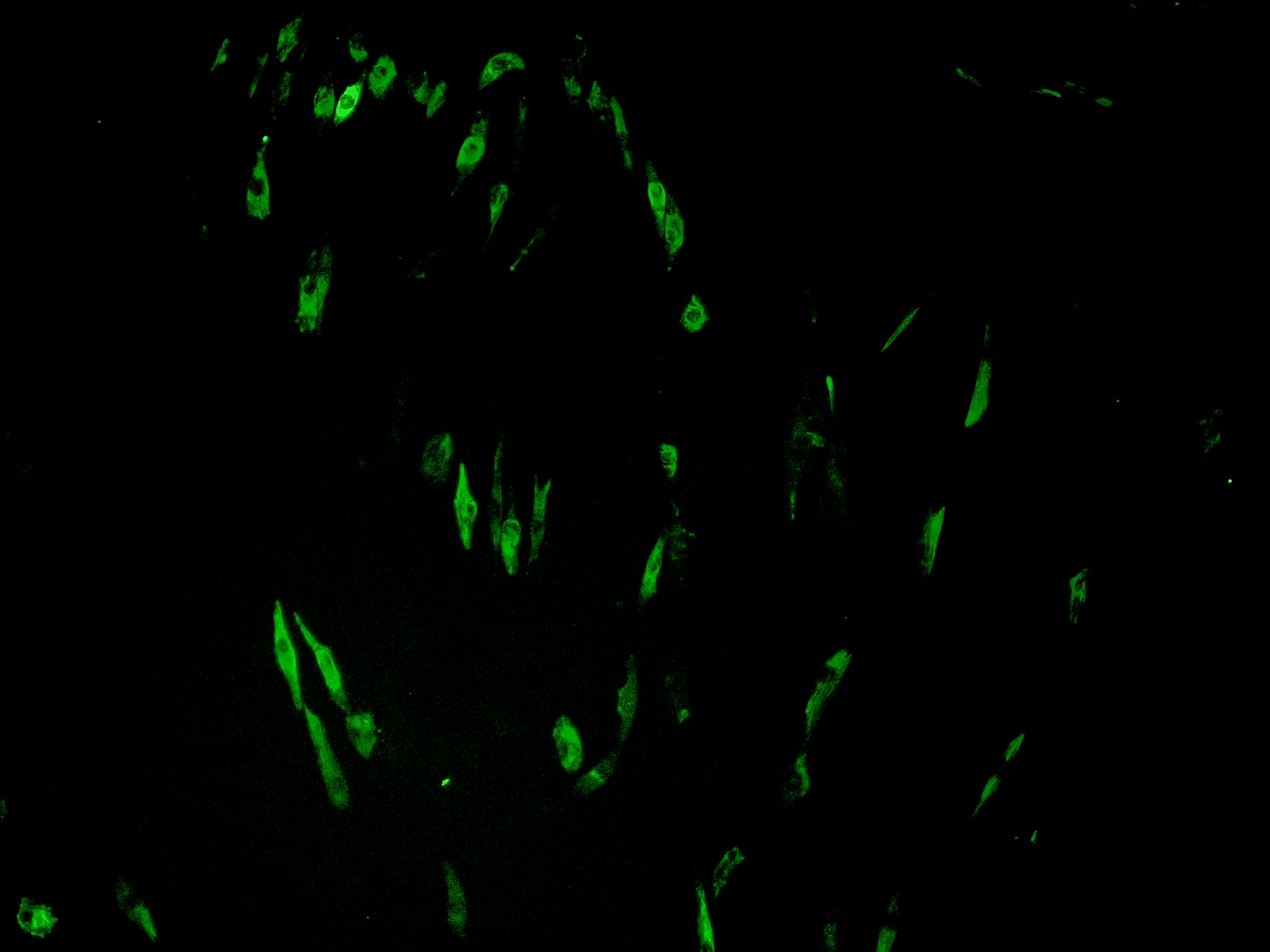

Supplement: Figure 9—source data 2. [file elife-81858-fig9-data2.zip › Figure 9-source data 2/fig9.d/Dex/Dex (4).tif]

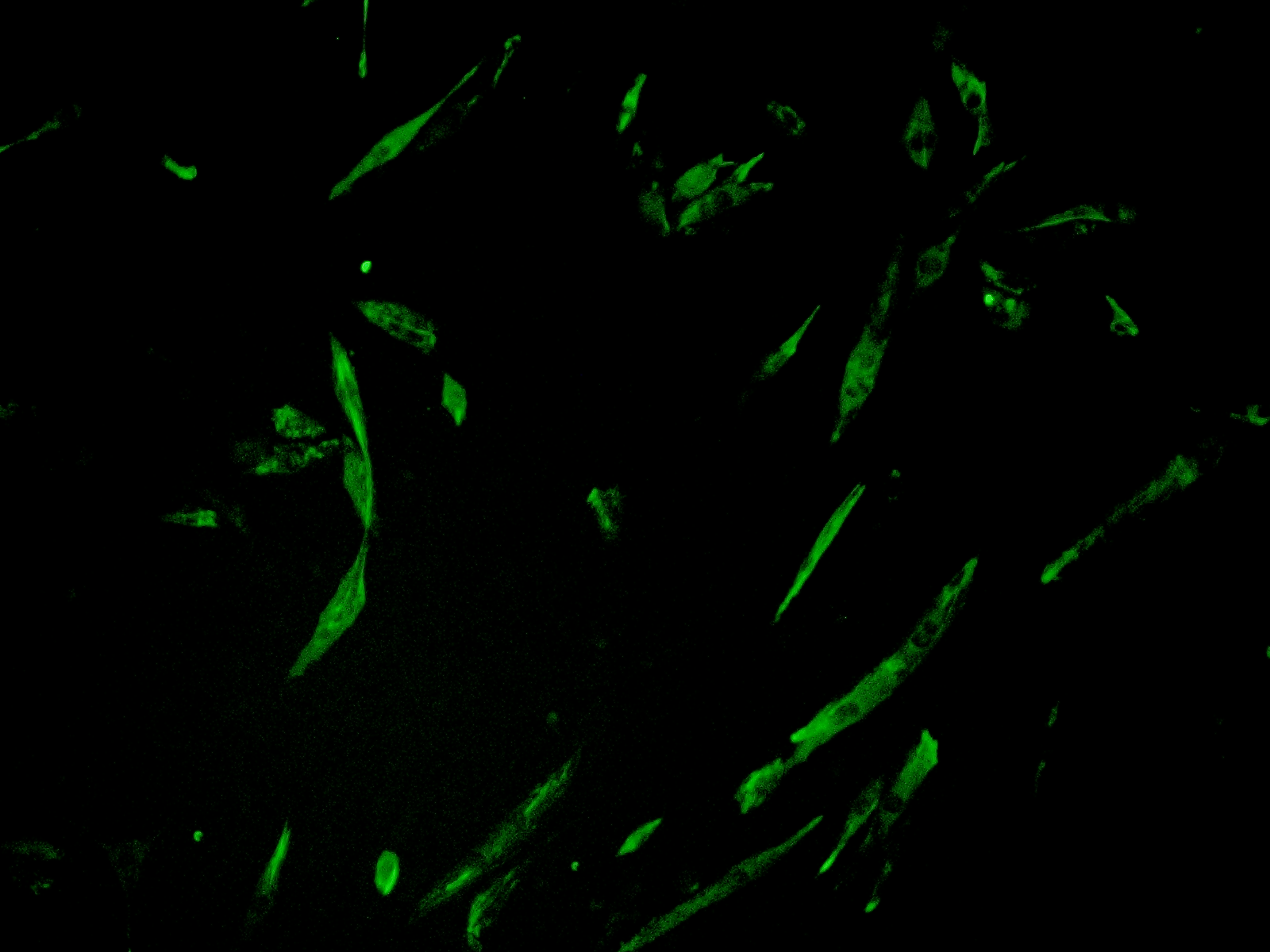

Supplement: Figure 9—source data 2. [file elife-81858-fig9-data2.zip › Figure 9-source data 2/fig9.d/Dex+Val/D+Val (1).tif]

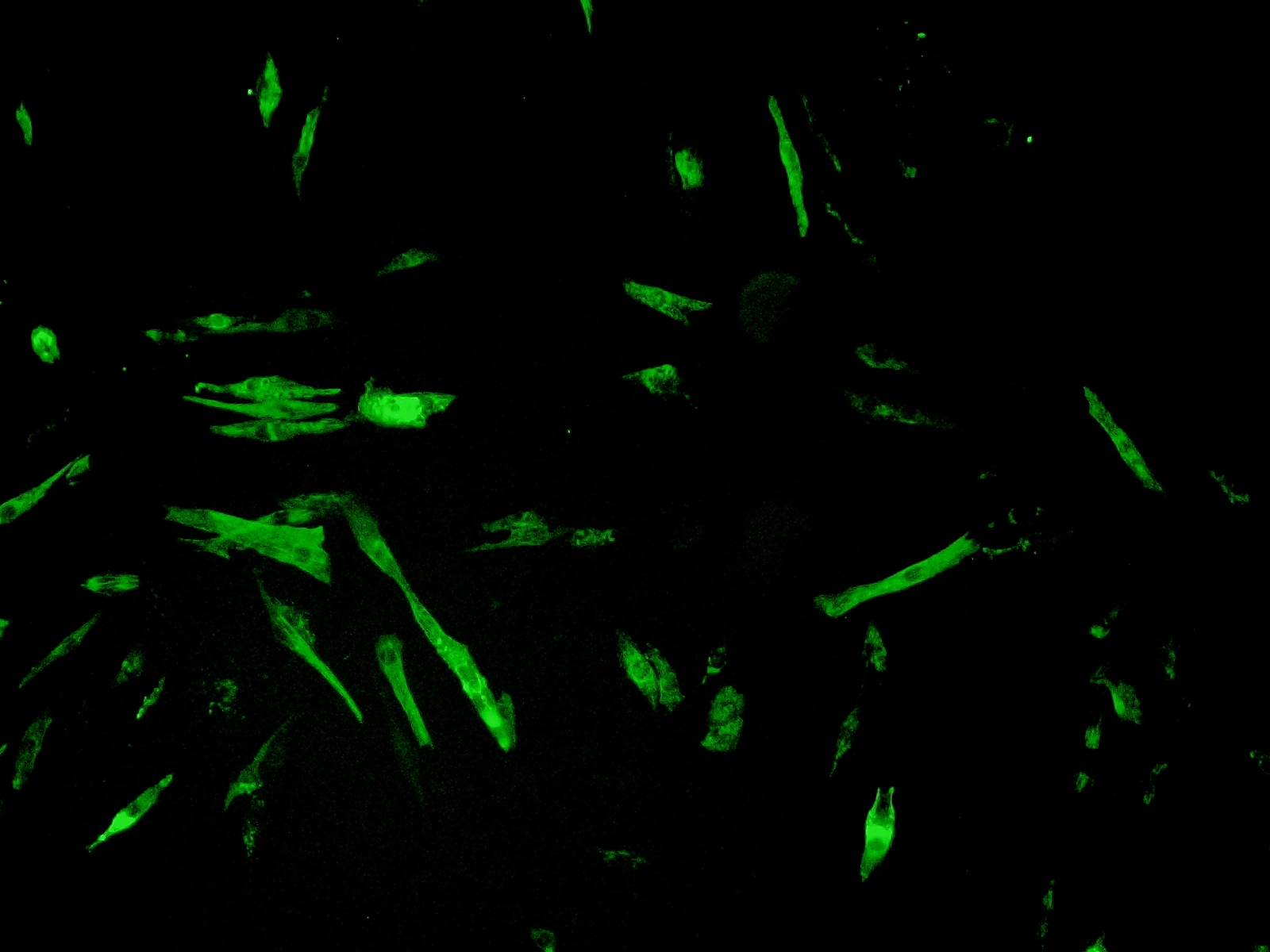

Supplement: Figure 9—source data 2. [file elife-81858-fig9-data2.zip › Figure 9-source data 2/fig9.d/Dex+Val/D+Val (2).tif]

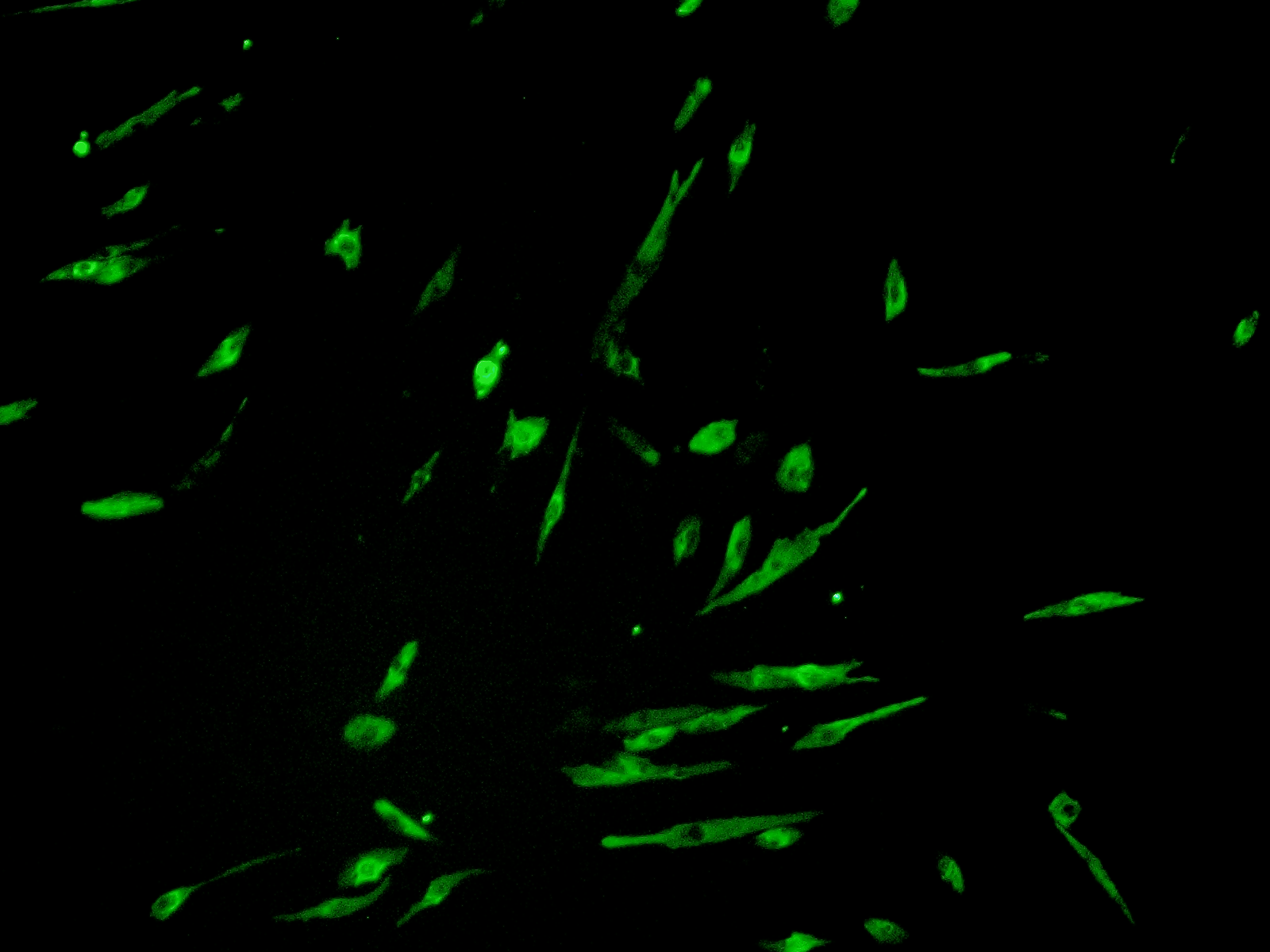

Supplement: Figure 9—source data 2. [file elife-81858-fig9-data2.zip › Figure 9-source data 2/fig9.d/Dex+Val/D+Val (3).tif]

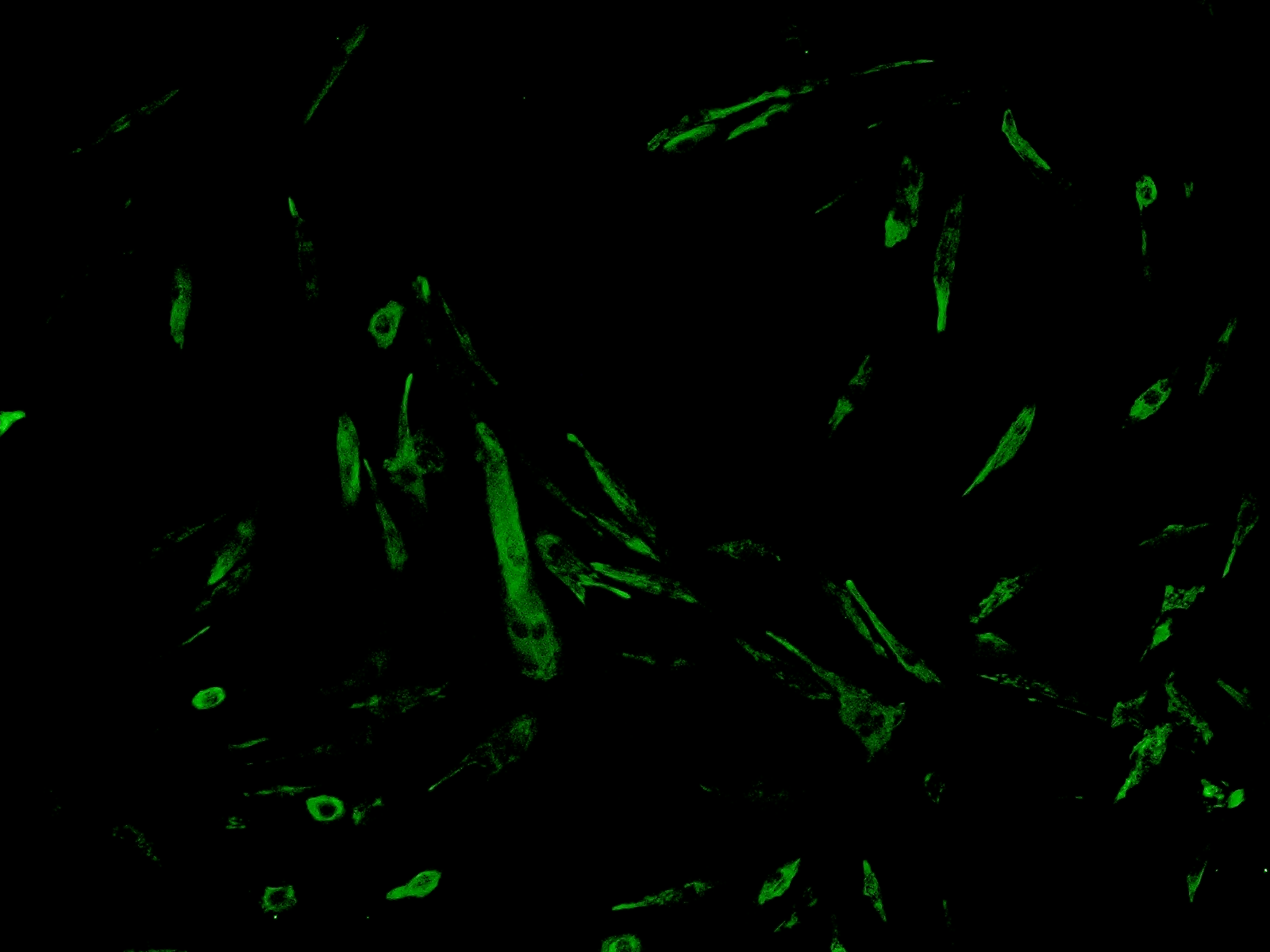

Supplement: Figure 9—source data 2. [file elife-81858-fig9-data2.zip › Figure 9-source data 2/fig9.d/Dex+Val/D+Val (4).tif]

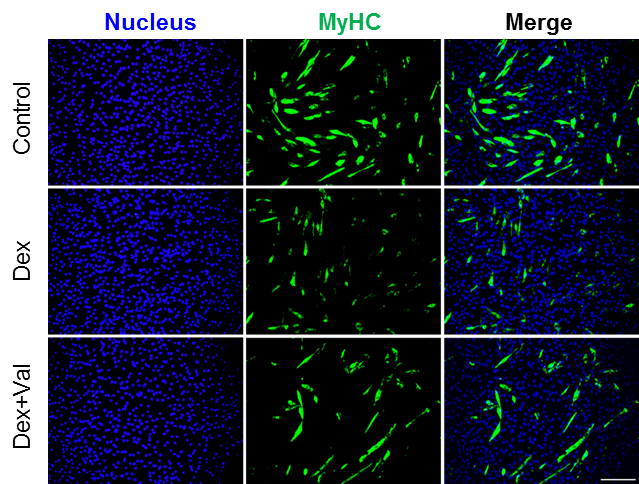

Supplement: Figure 9—source data 2. [file elife-81858-fig9-data2.zip › Figure 9-source data 2/fig9.d/fig.9d.tif]

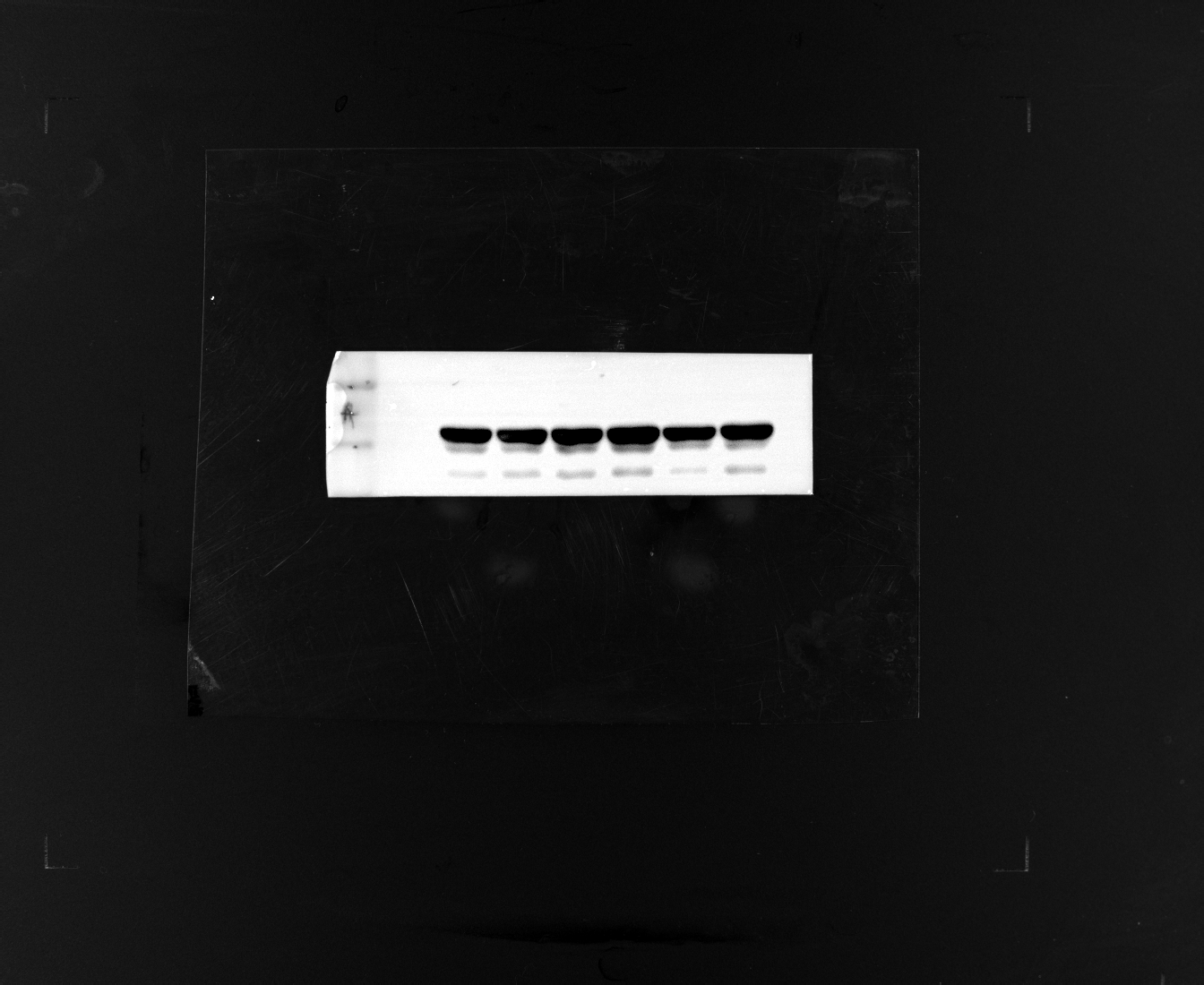

Supplement: Figure 9—source data 3. [file elife-81858-fig9-data3.zip › Figure 9-source data 3/fig9b.Atrogin-1.tif]

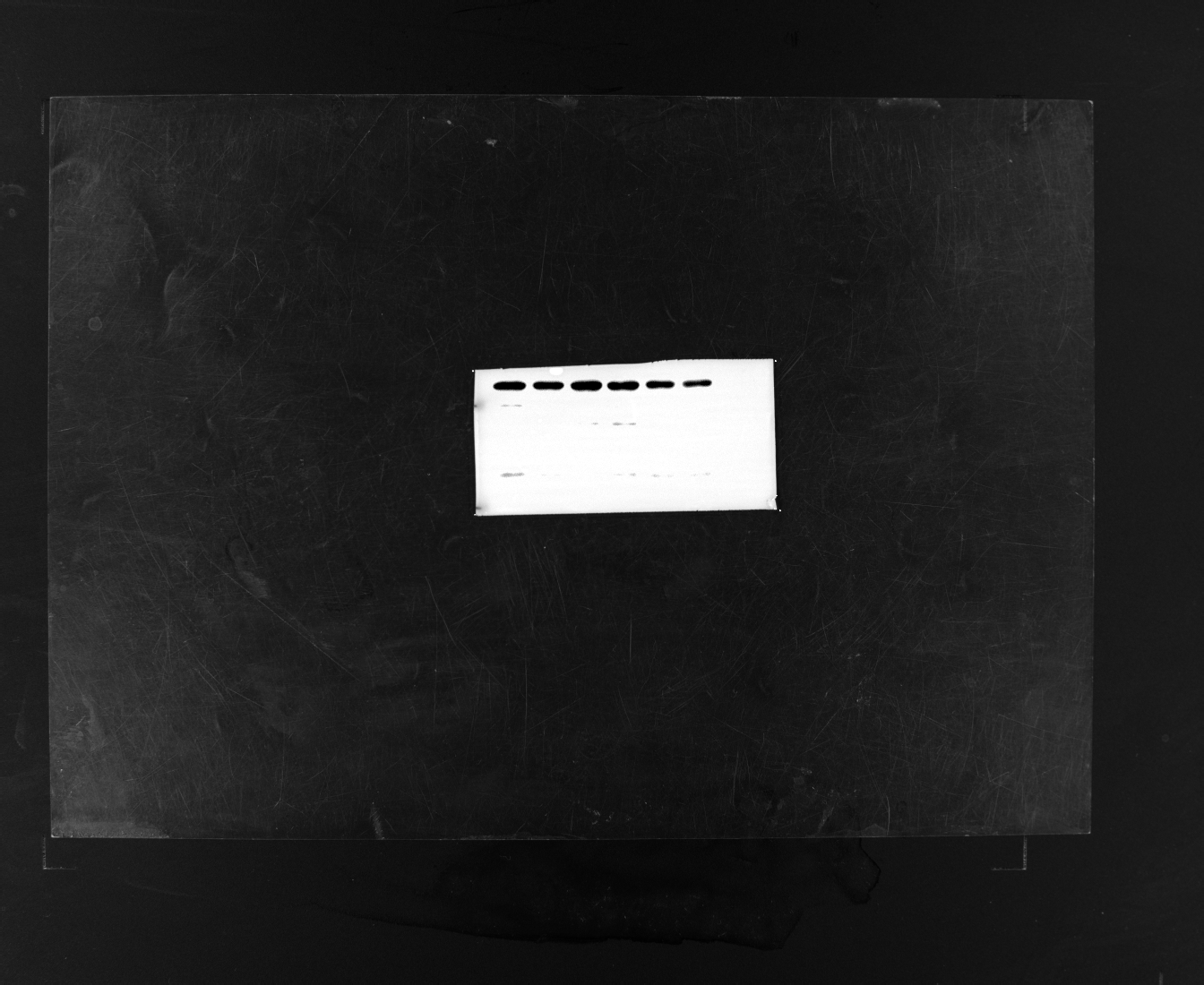

Supplement: Figure 9—source data 3. [file elife-81858-fig9-data3.zip › Figure 9-source data 3/fig9b.MuRF-1.tif]

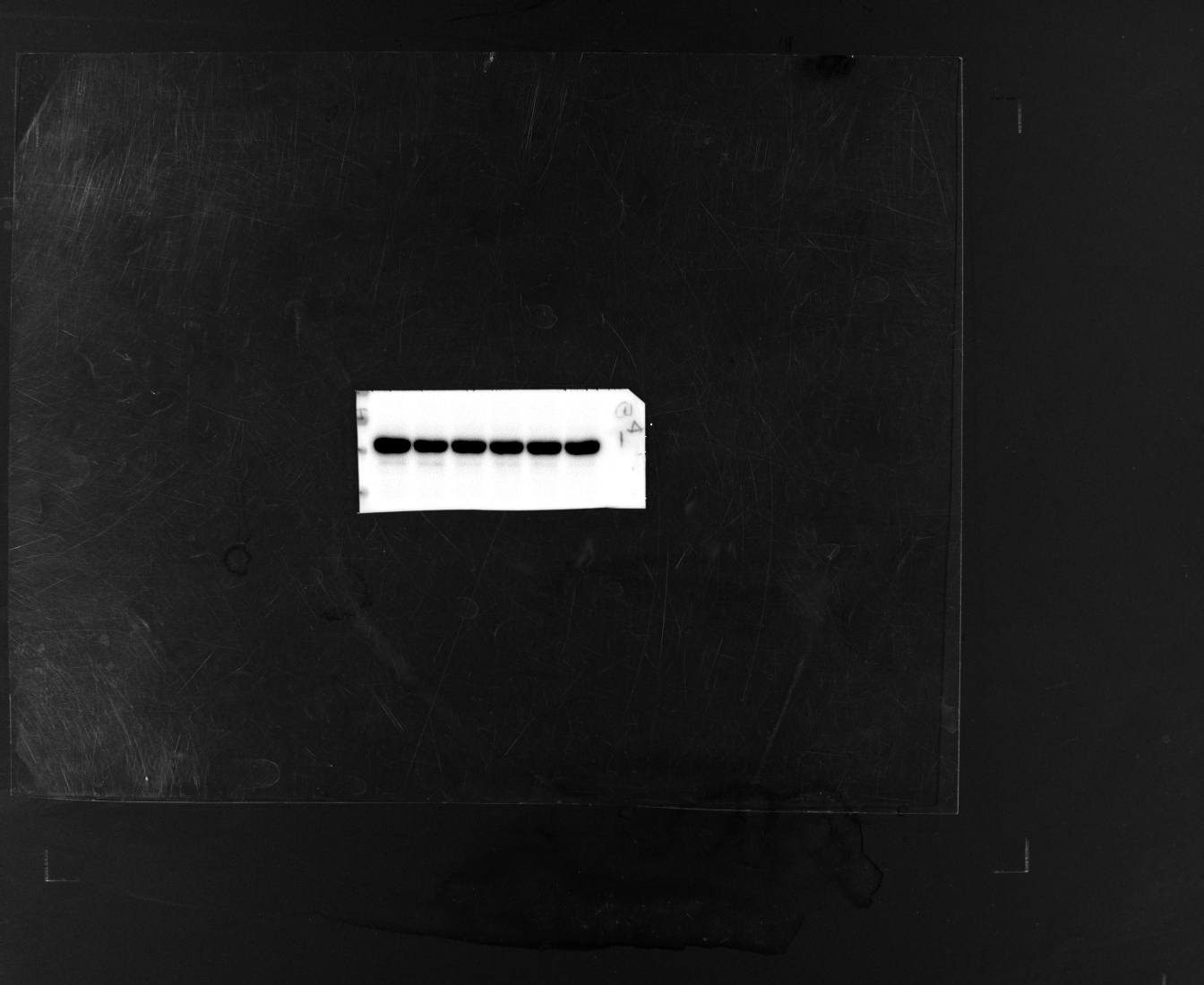

Supplement: Figure 9—source data 3. [file elife-81858-fig9-data3.zip › Figure 9-source data 3/fig9b.actin.tif]

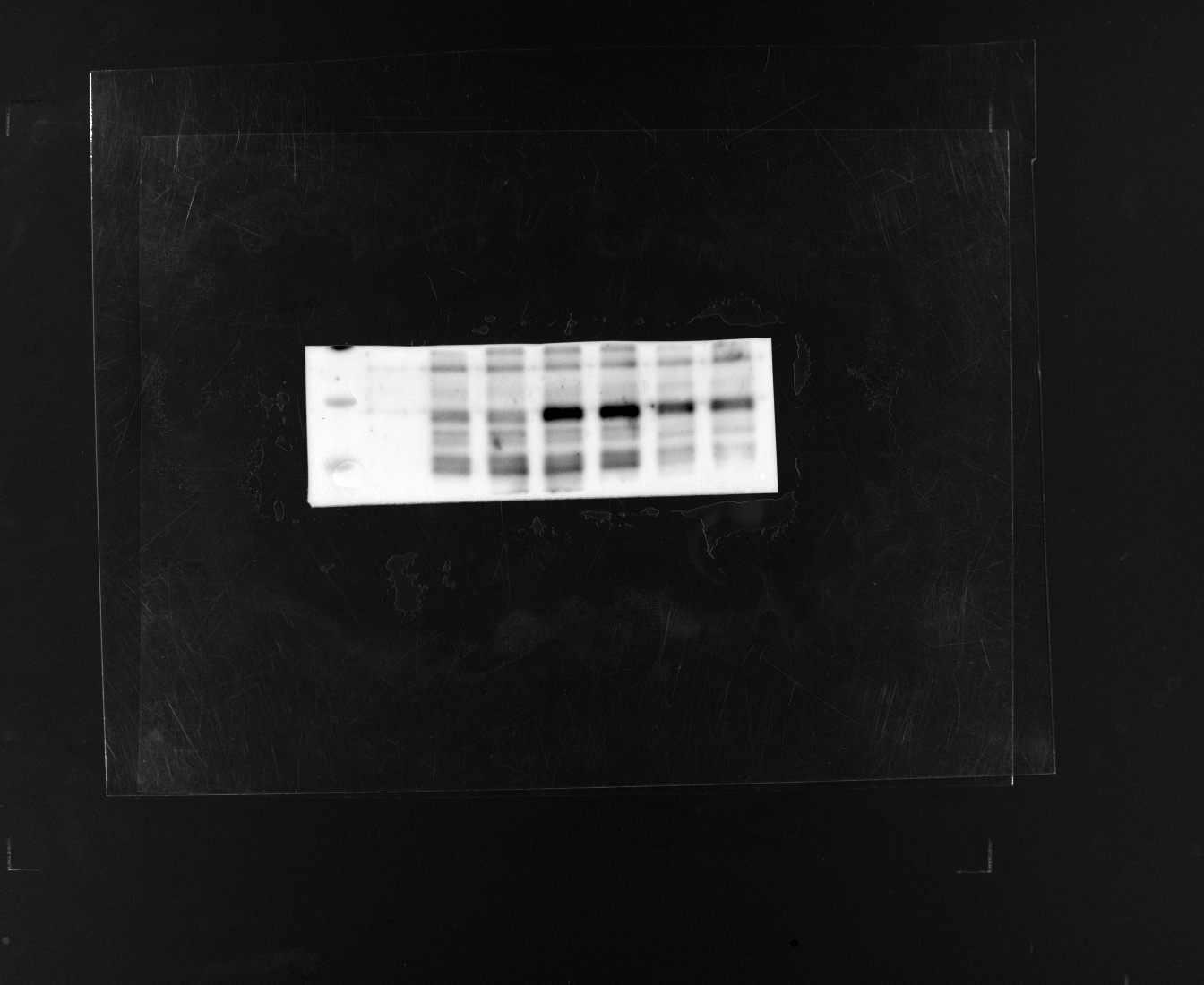

Supplement: Figure 9—source data 3. [file elife-81858-fig9-data3.zip › Figure 9-source data 3/fig9e.Atrogin-1.tif]

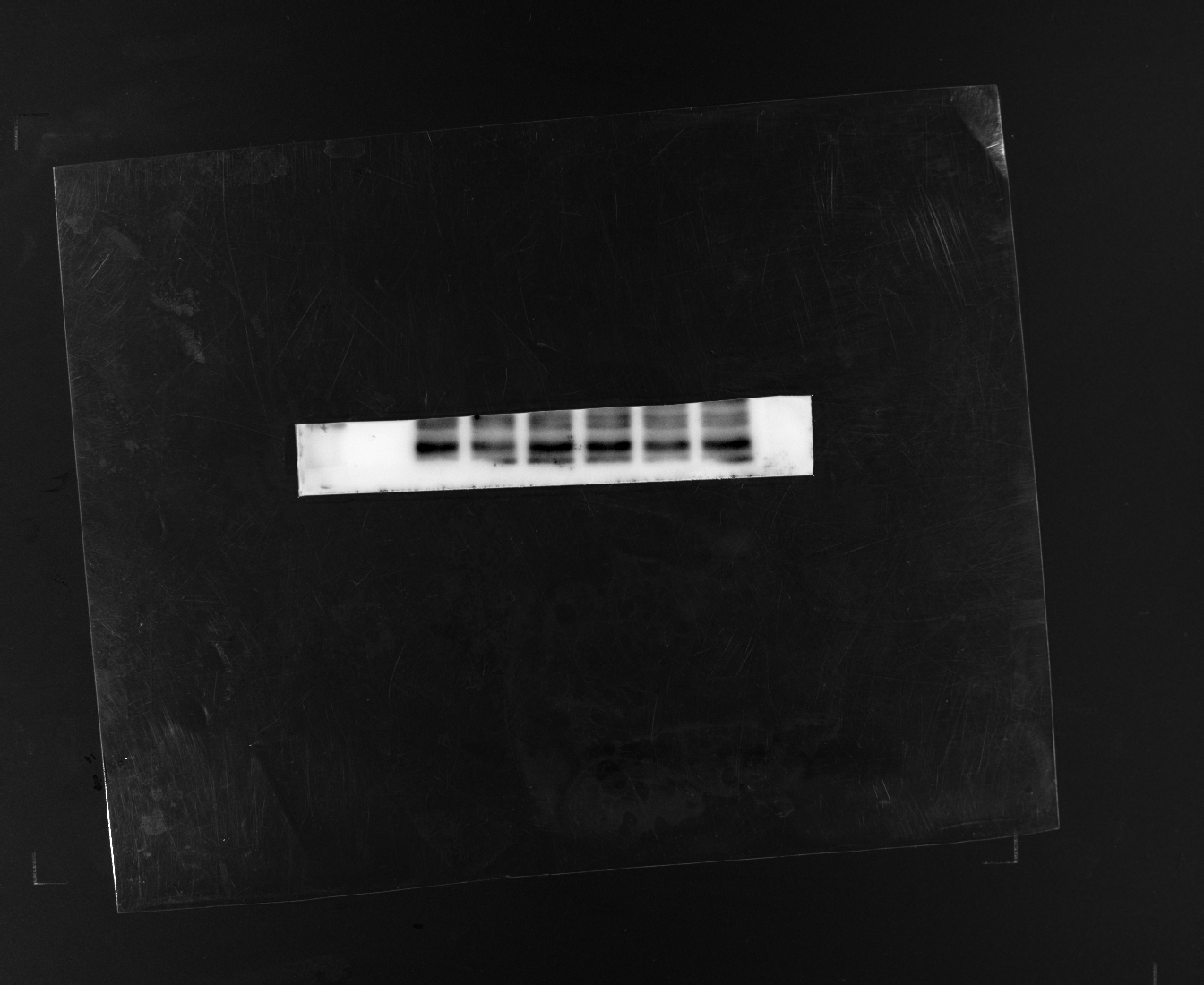

Supplement: Figure 9—source data 3. [file elife-81858-fig9-data3.zip › Figure 9-source data 3/fig9e.MuRF-1.tif]

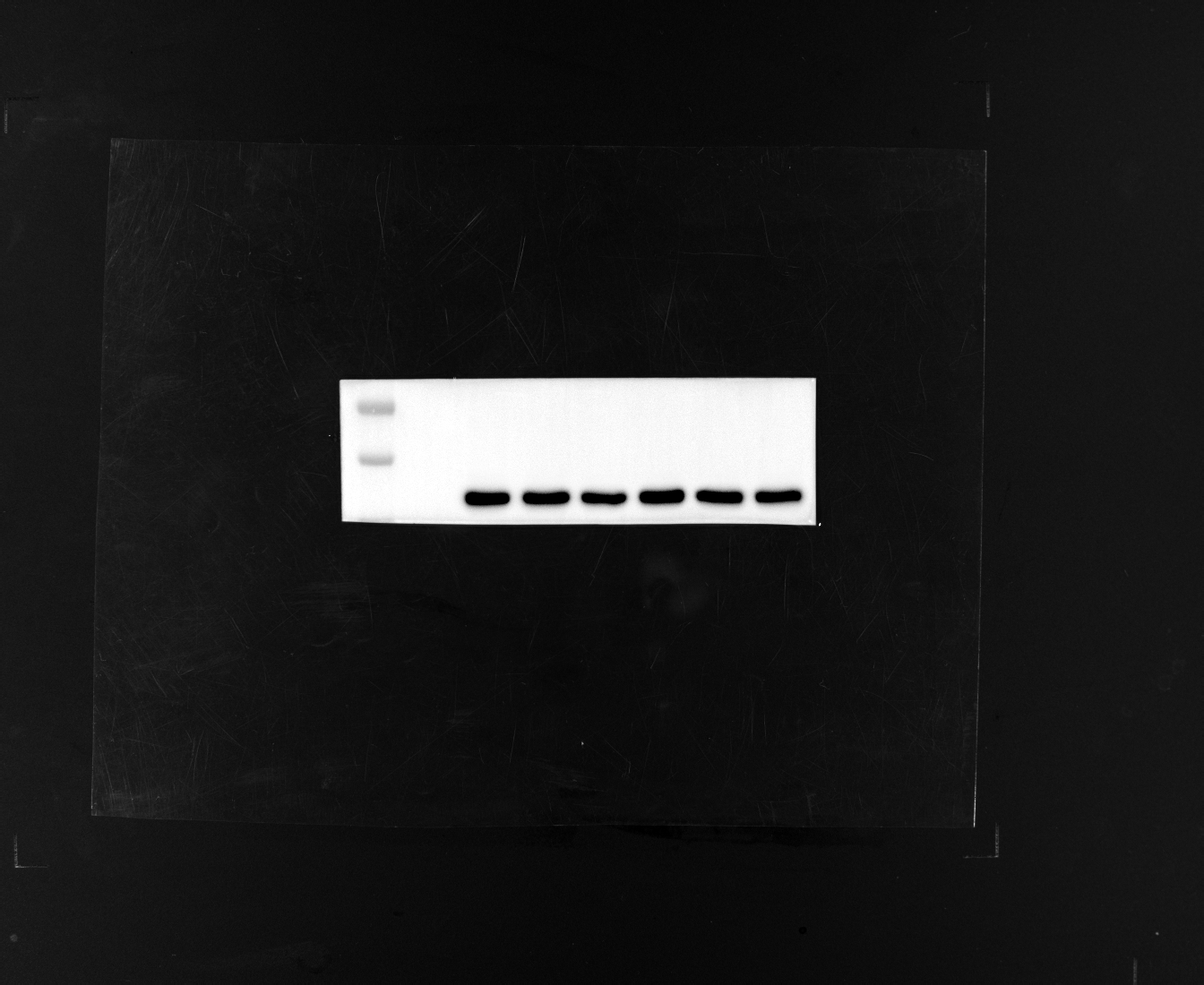

Supplement: Figure 9—source data 3. [file elife-81858-fig9-data3.zip › Figure 9-source data 3/fig9e.actin.tif]
