# Supplementary material for: Fecal transplant from myostatin deletion pigs positively impacts the gut-muscle axis
Source: eLife. 2023 Apr 11;12:e81858. doi: 10.7554/eLife.81858 (PMC10121221; doi:10.7554/eLife.81858)

Figure 7D source data

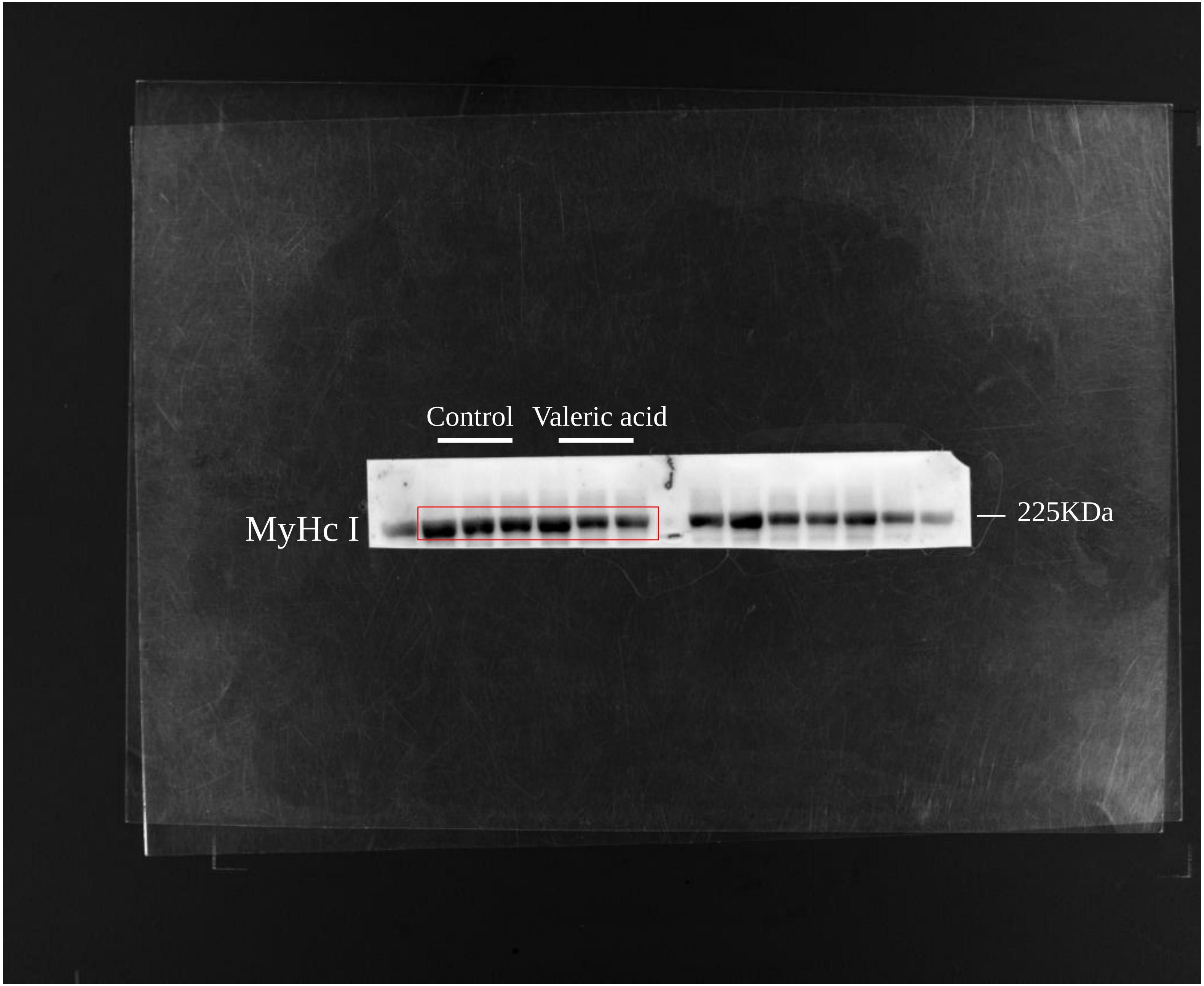

Figure 7D source data

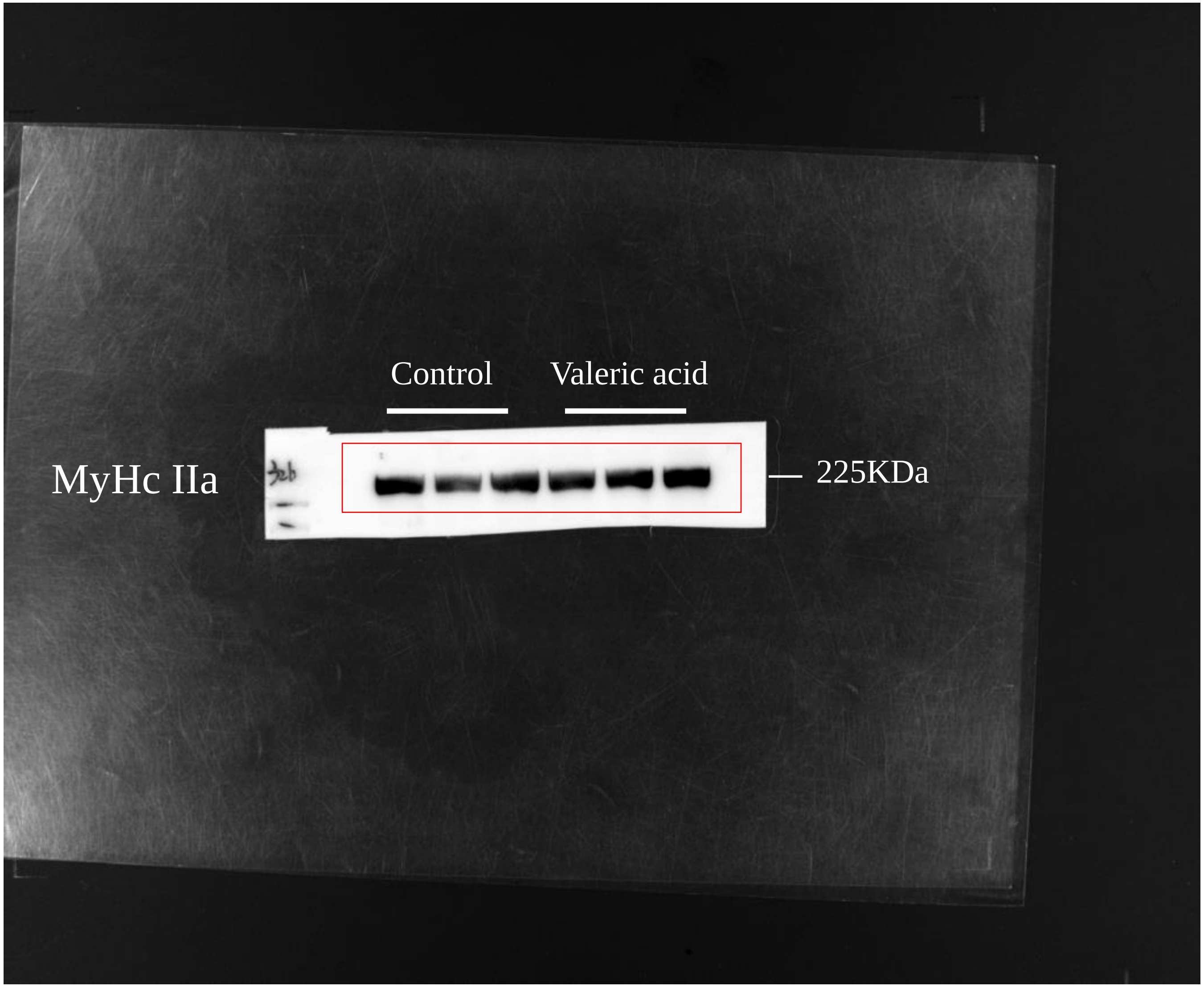

Figure 7D source data

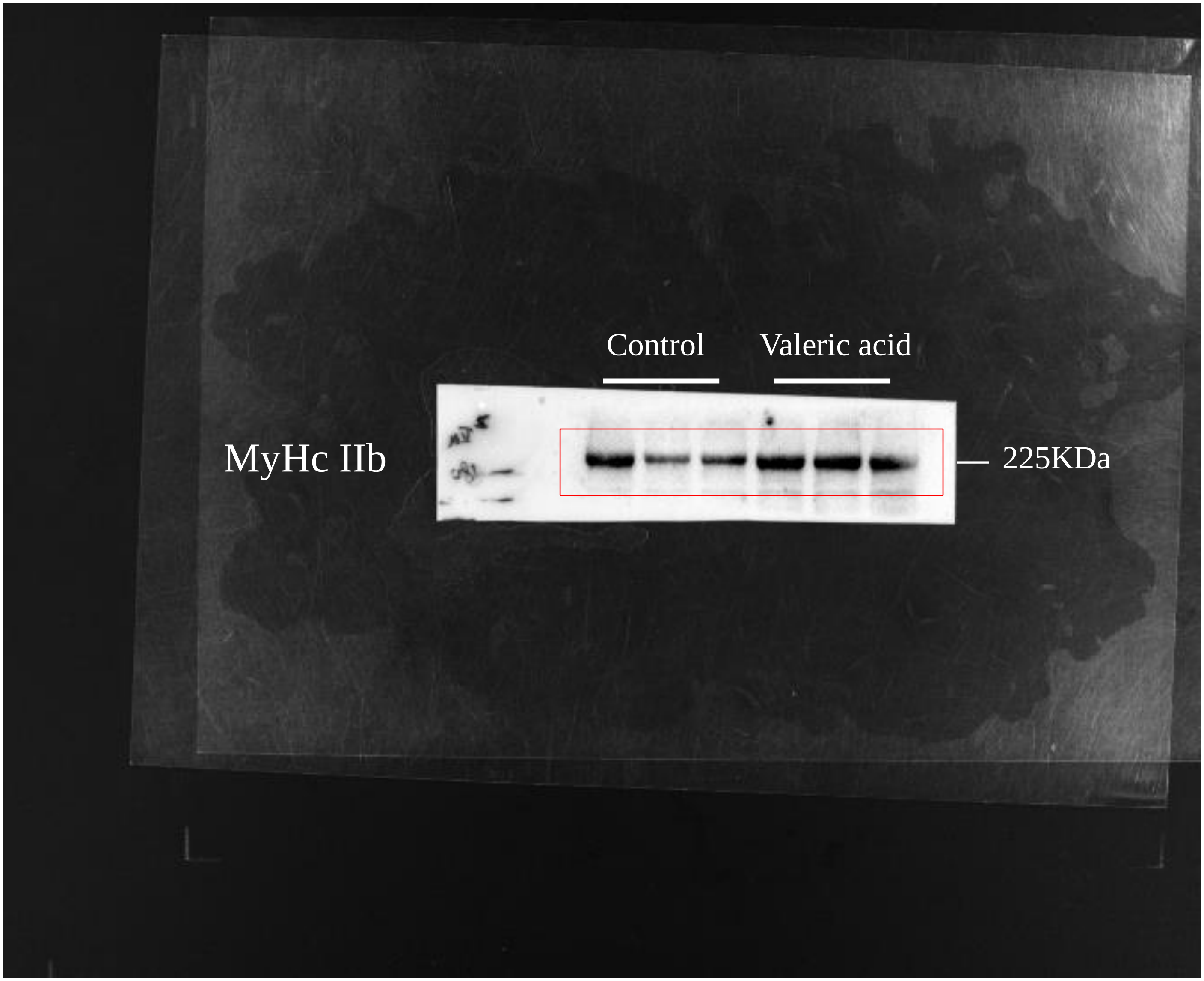

Figure 7D source data

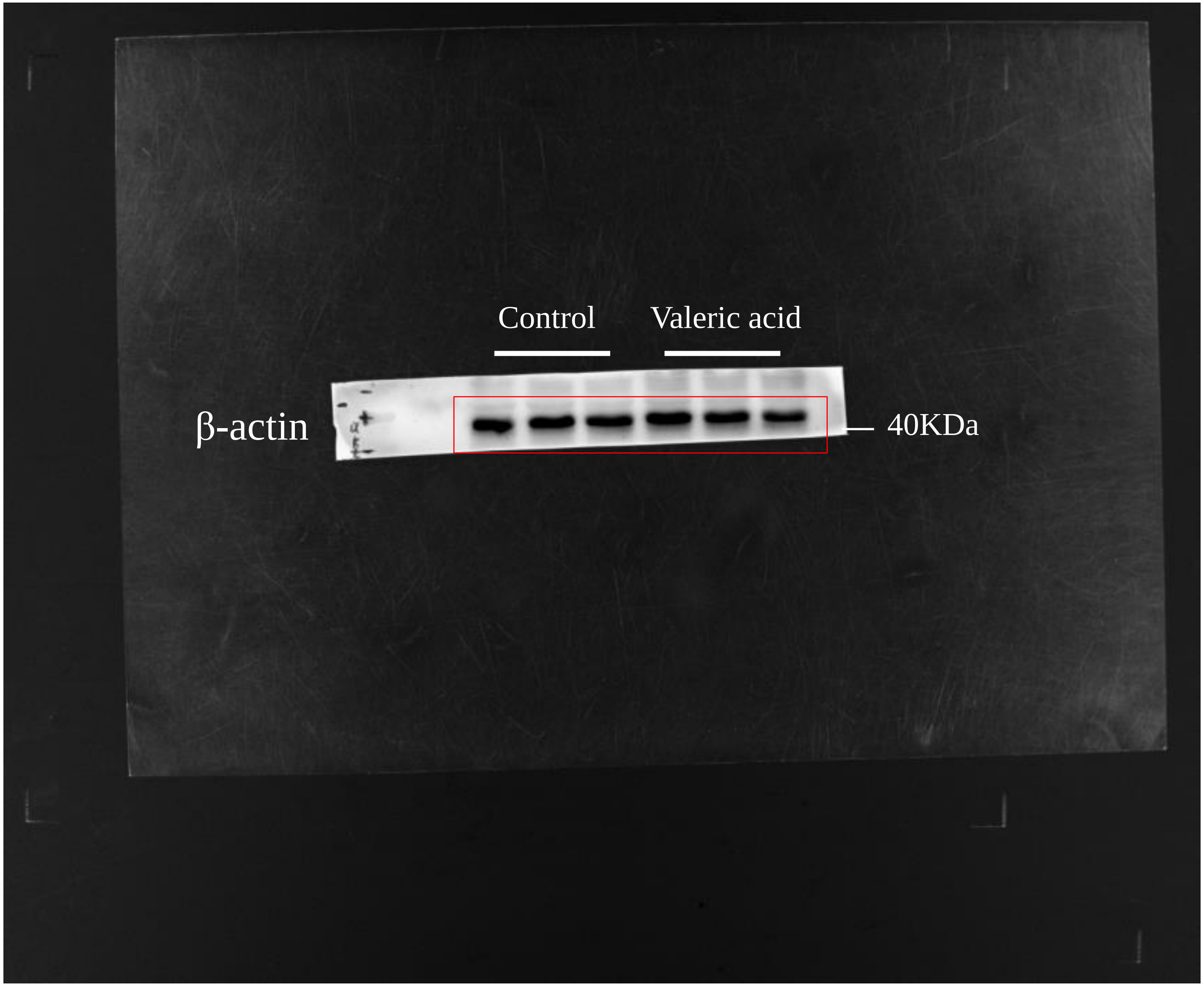

Figure 7E source data

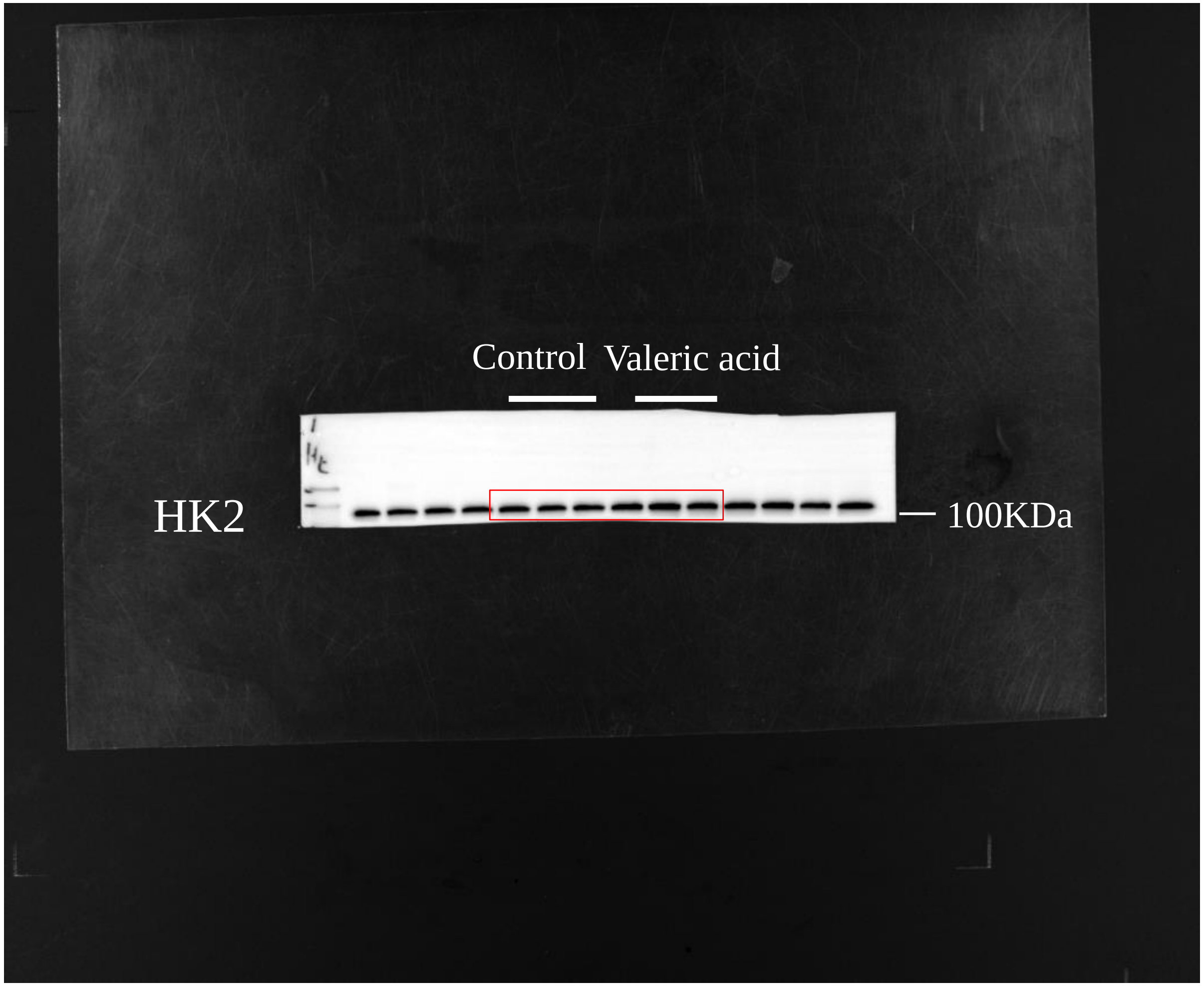

Figure 7E source data

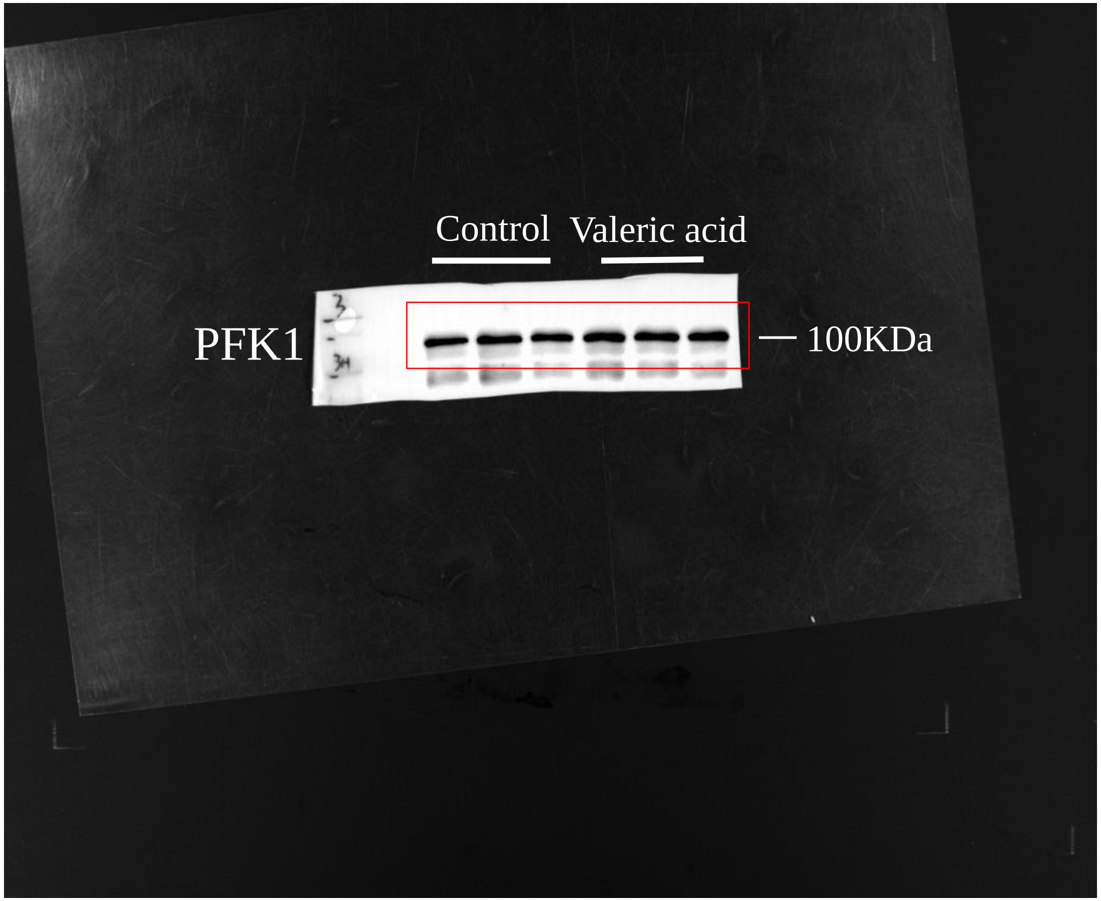

Figure 7E source data

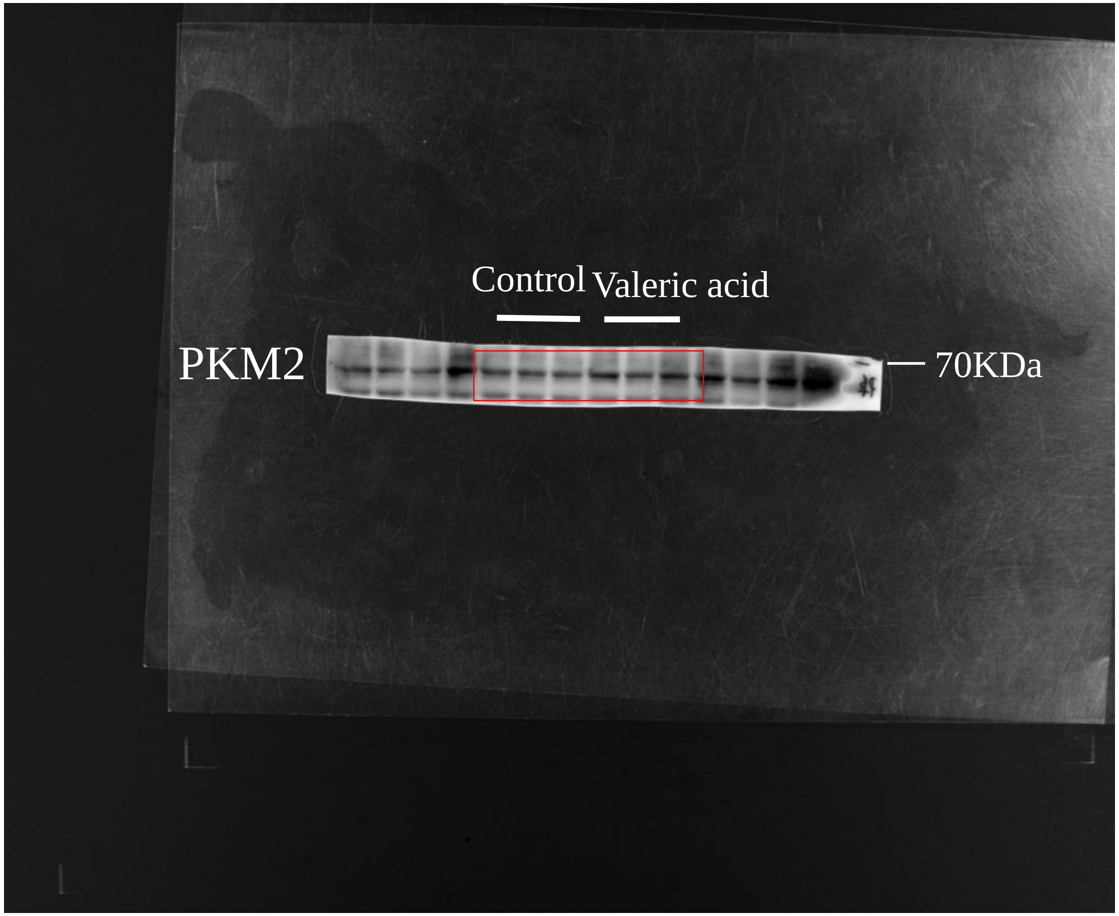

Figure 7E source data

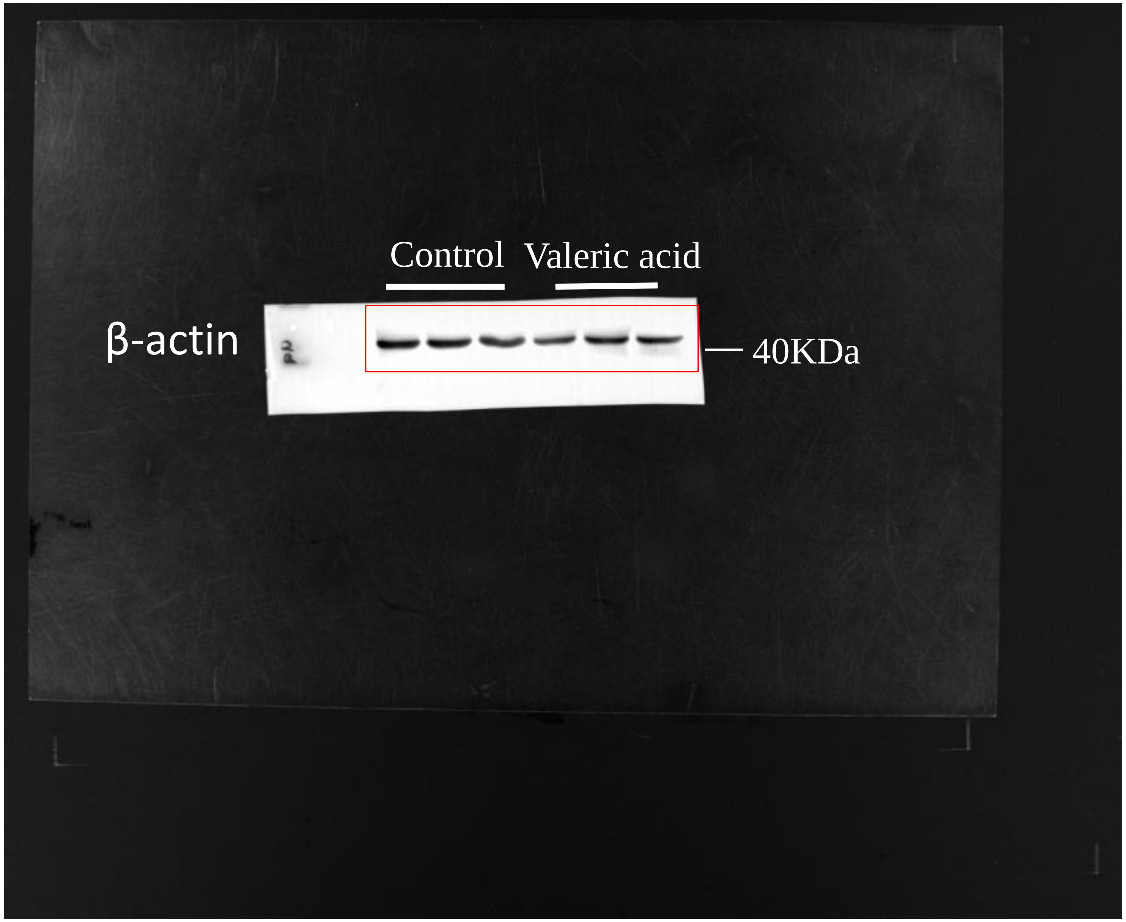

Figure 7F source data

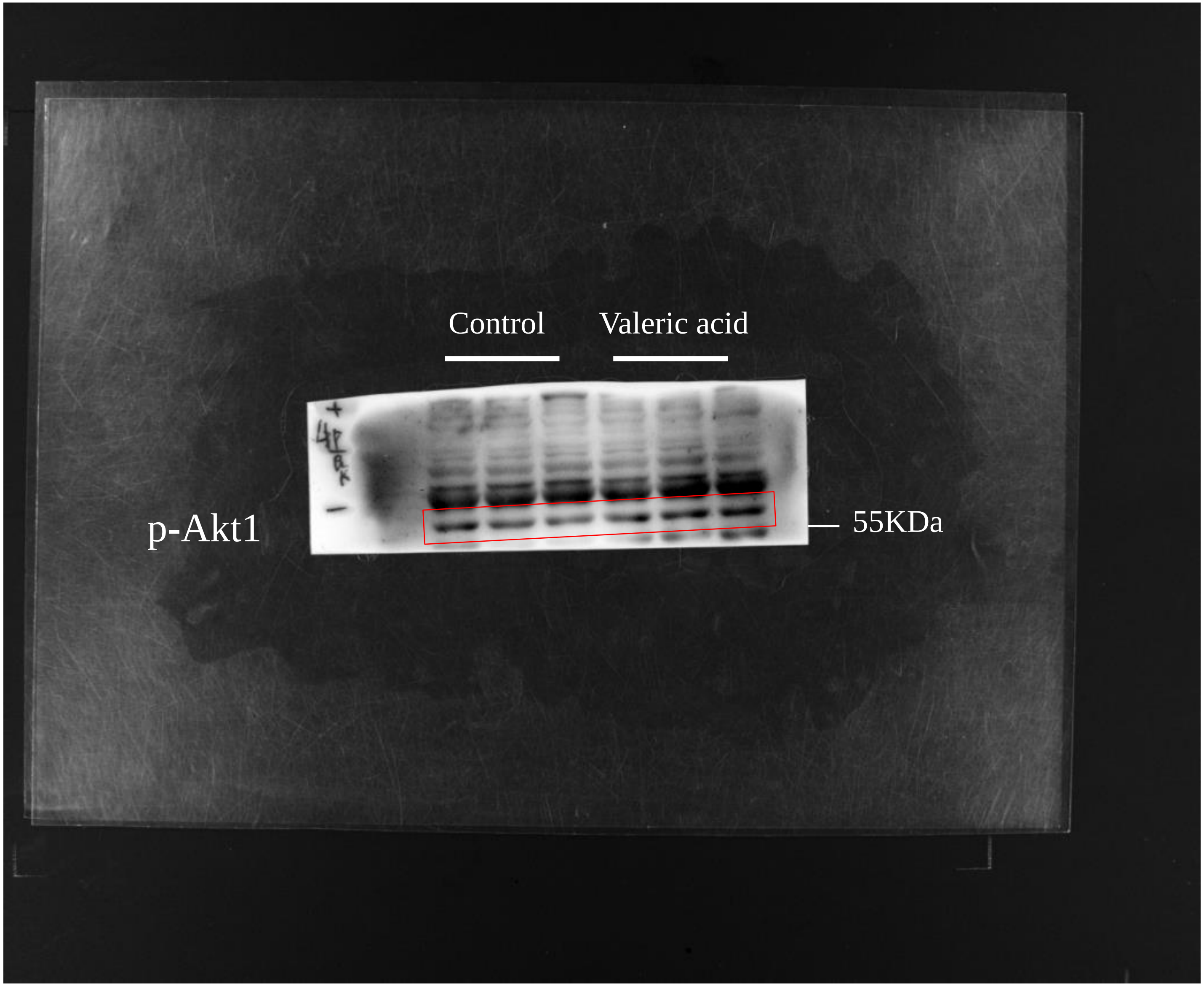

Figure 7F source data

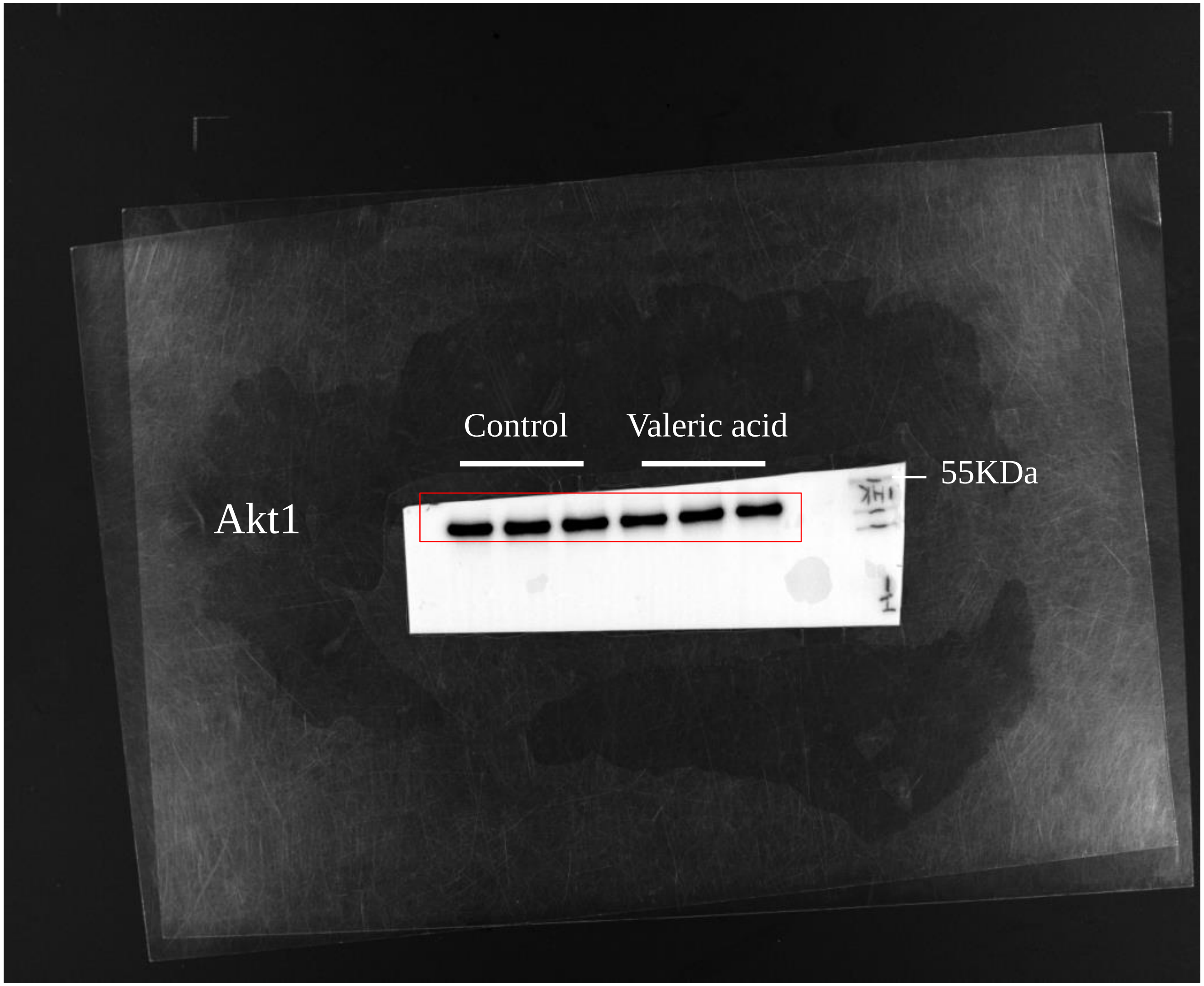

Figure 7F source data

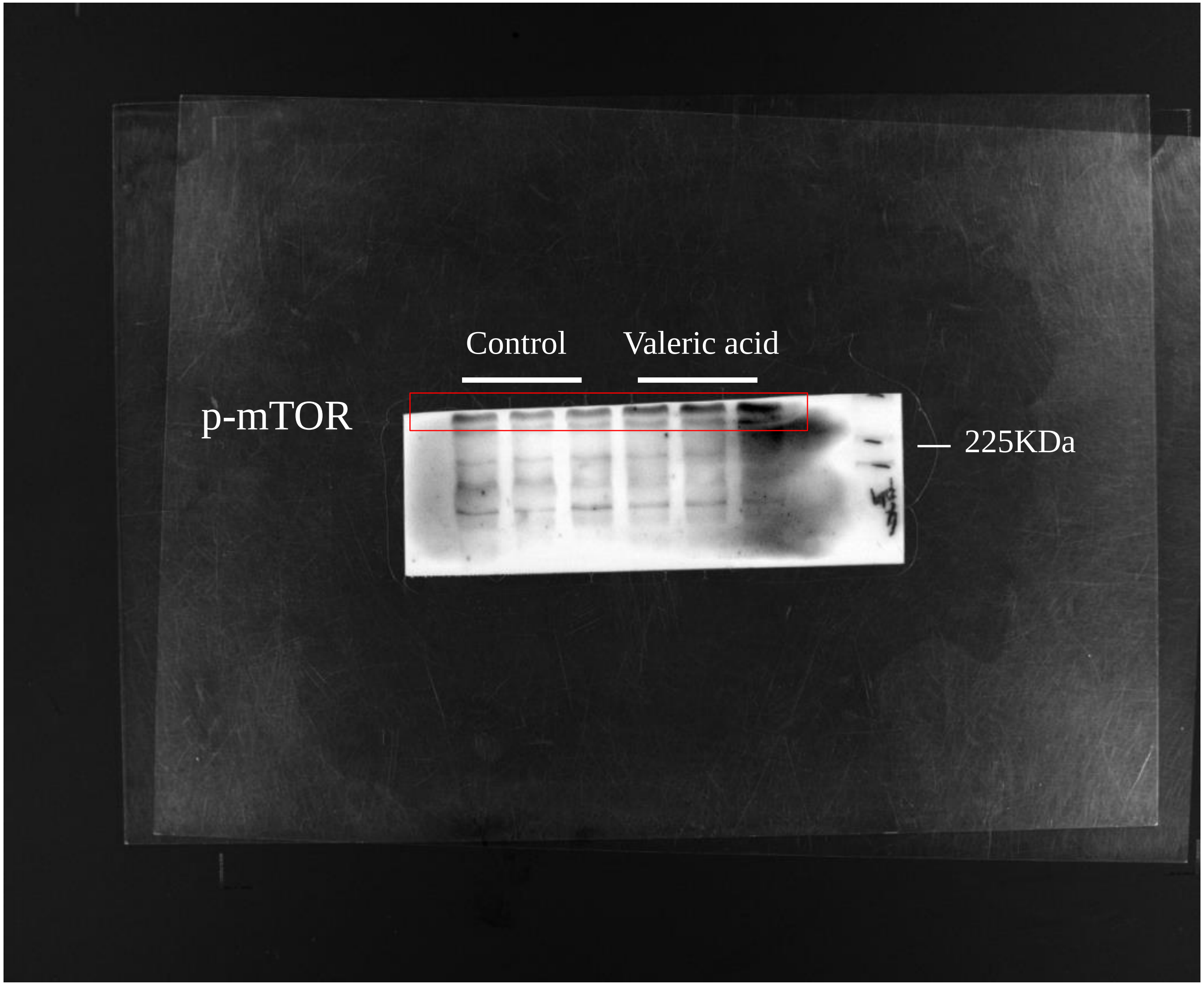

Figure 7F source data

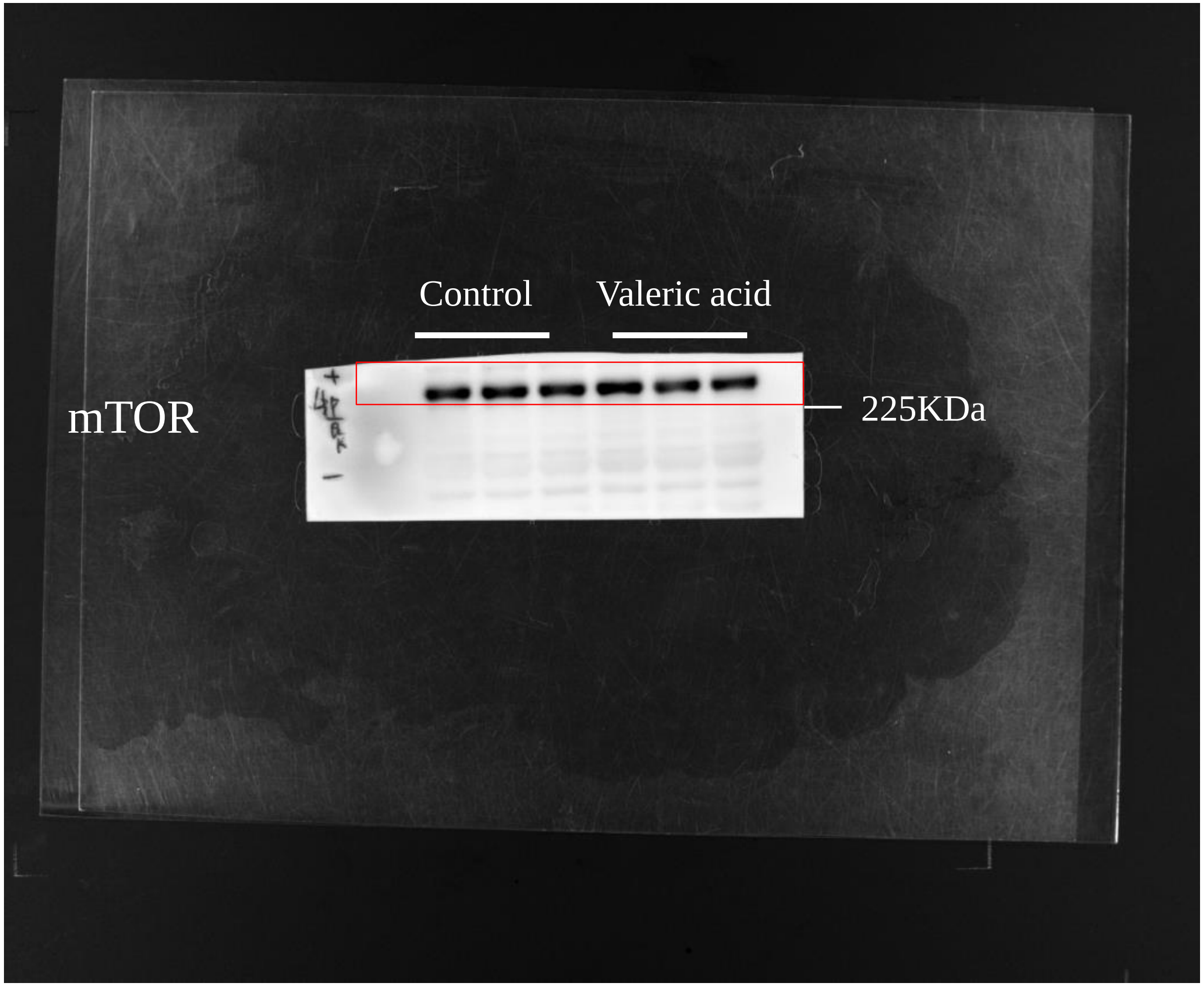

Figure 7F source data

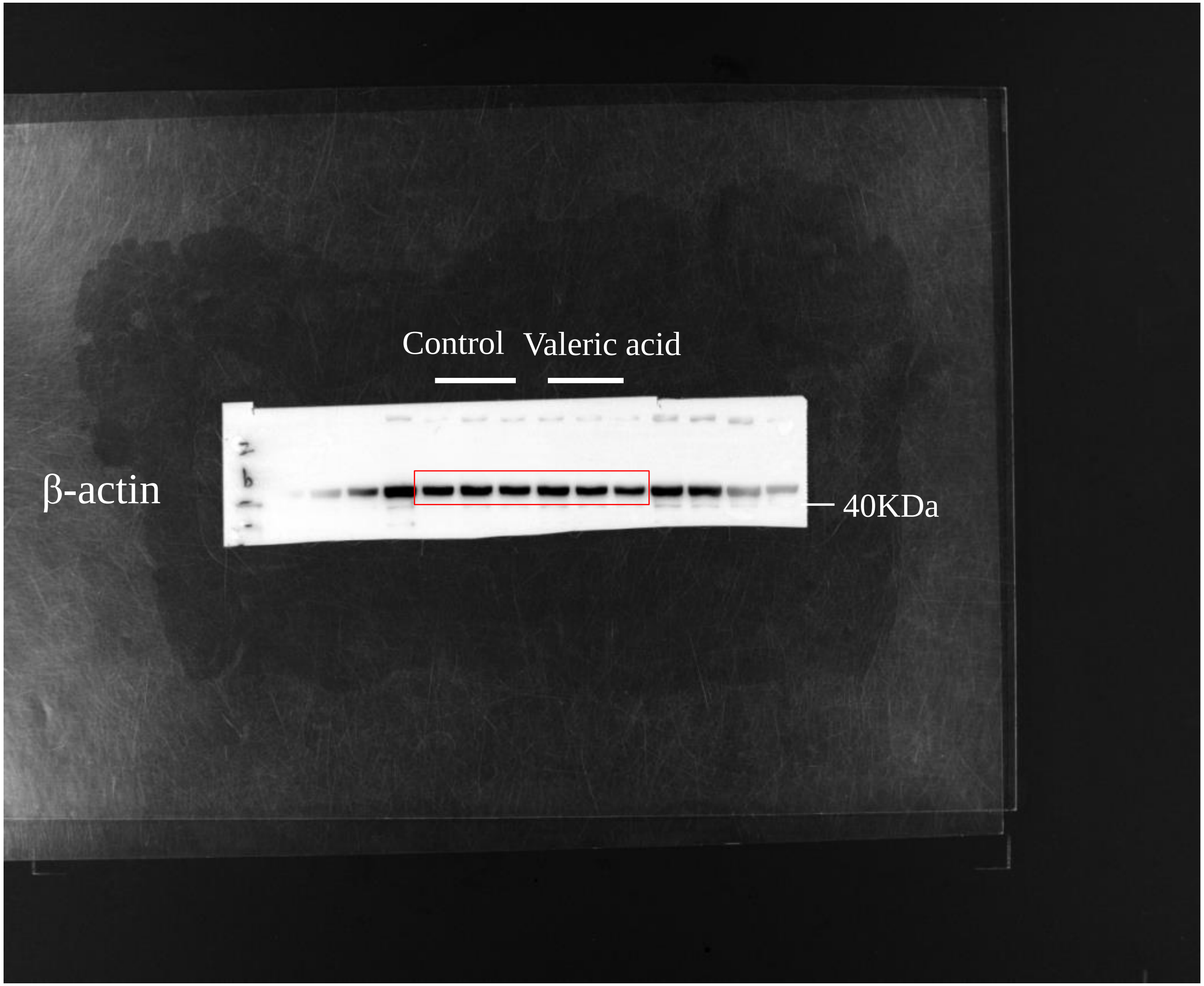

Supplement: Figure 7—source data 2. [file elife-81858-fig7-data2.zip › Figure 7-source data 2/Raw western blot images for Figure 7.pdf]
