# Supplementary material for: Fecal transplant from myostatin deletion pigs positively impacts the gut-muscle axis
Source: eLife. 2023 Apr 11;12:e81858. doi: 10.7554/eLife.81858 (PMC10121221; doi:10.7554/eLife.81858)

Figure 9B source data

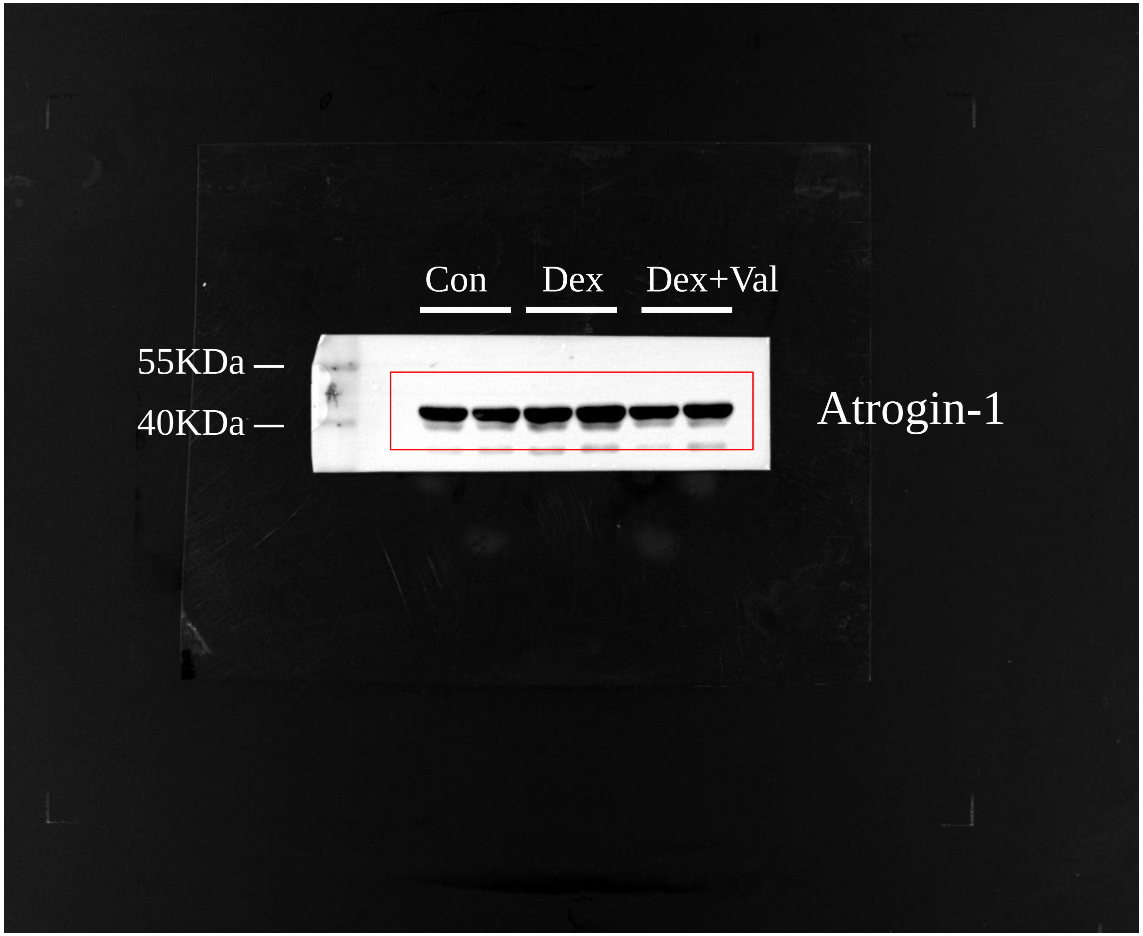

Figure 9B source data

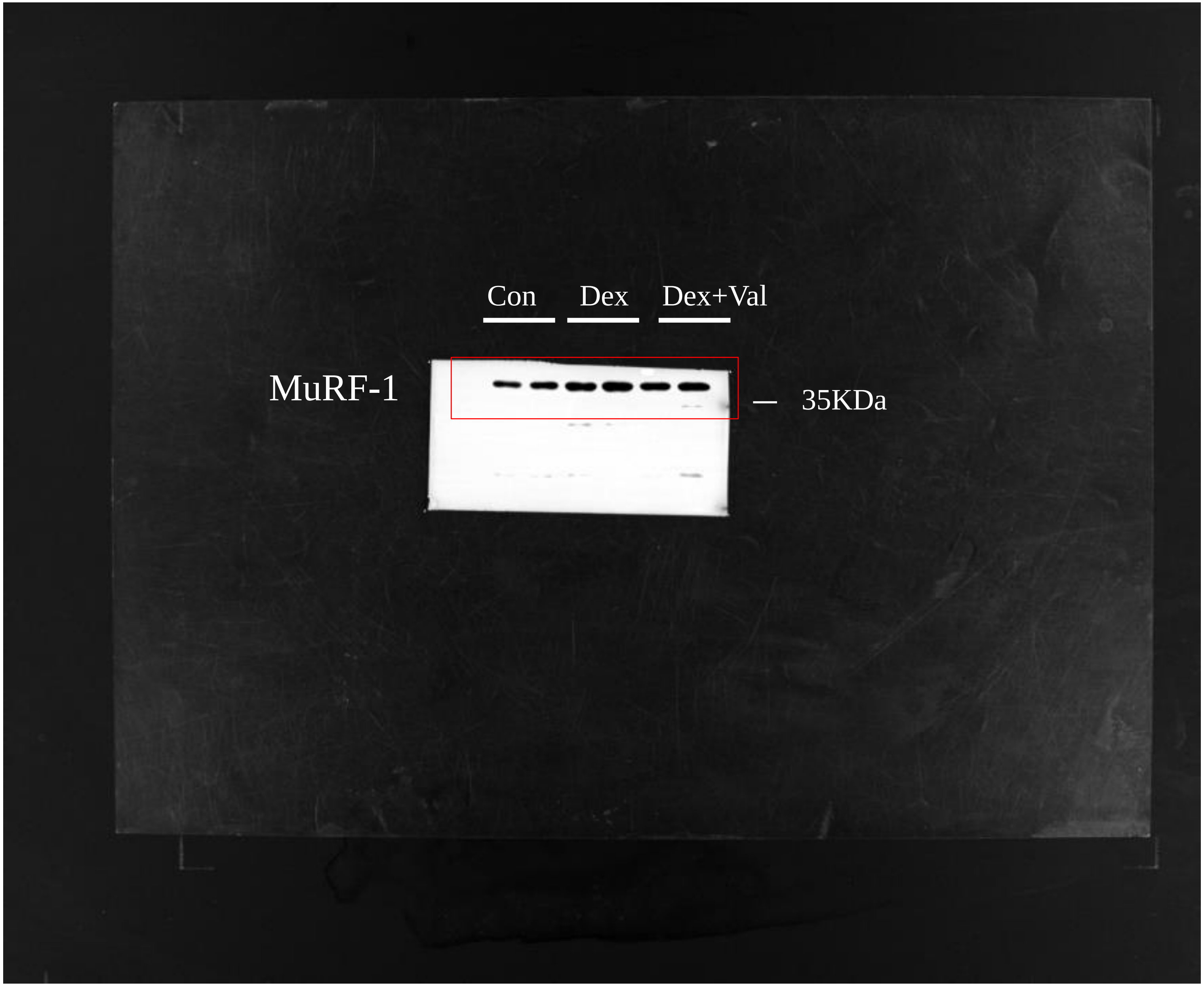

Figure 9B source data

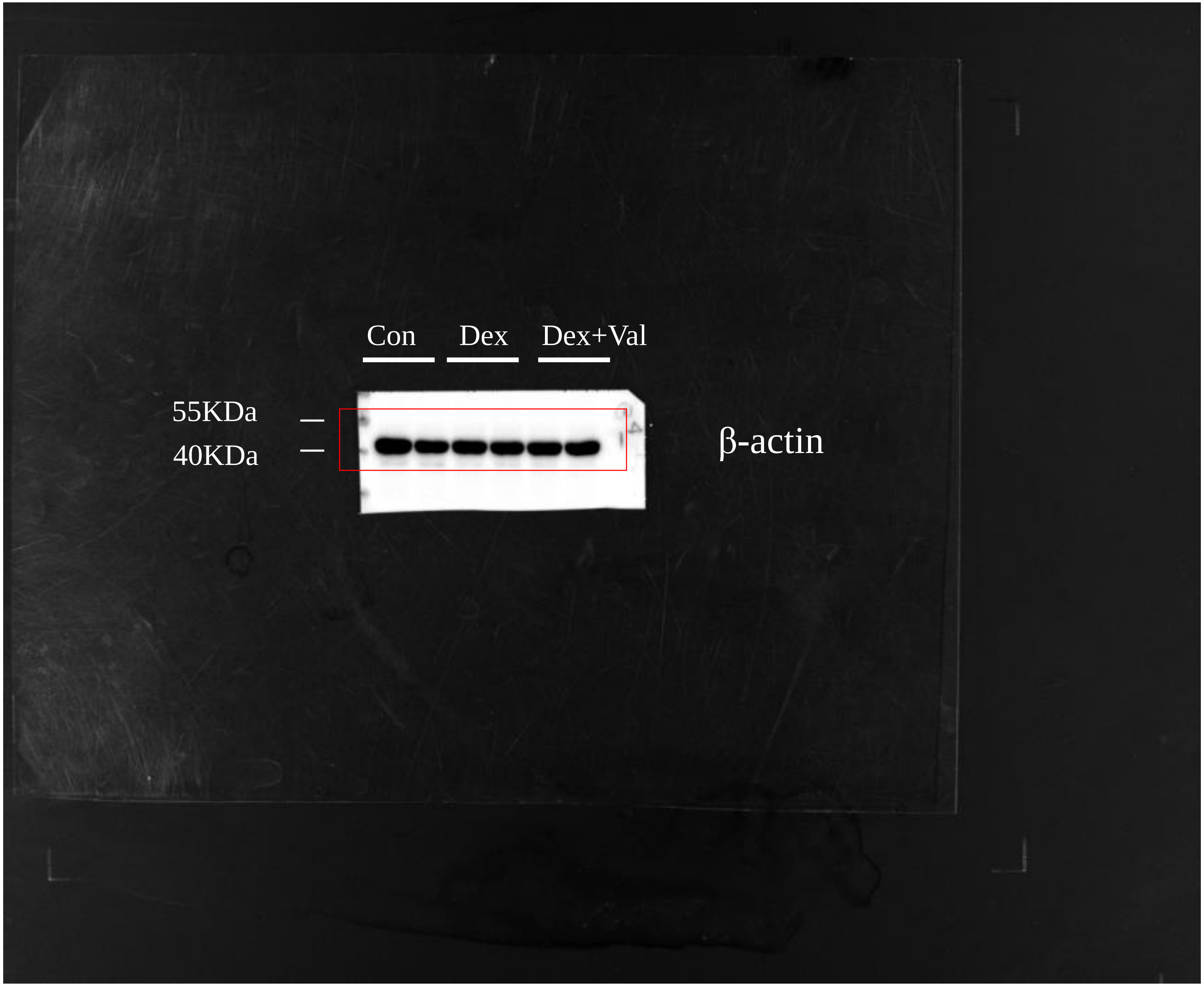

Figure 9E source data

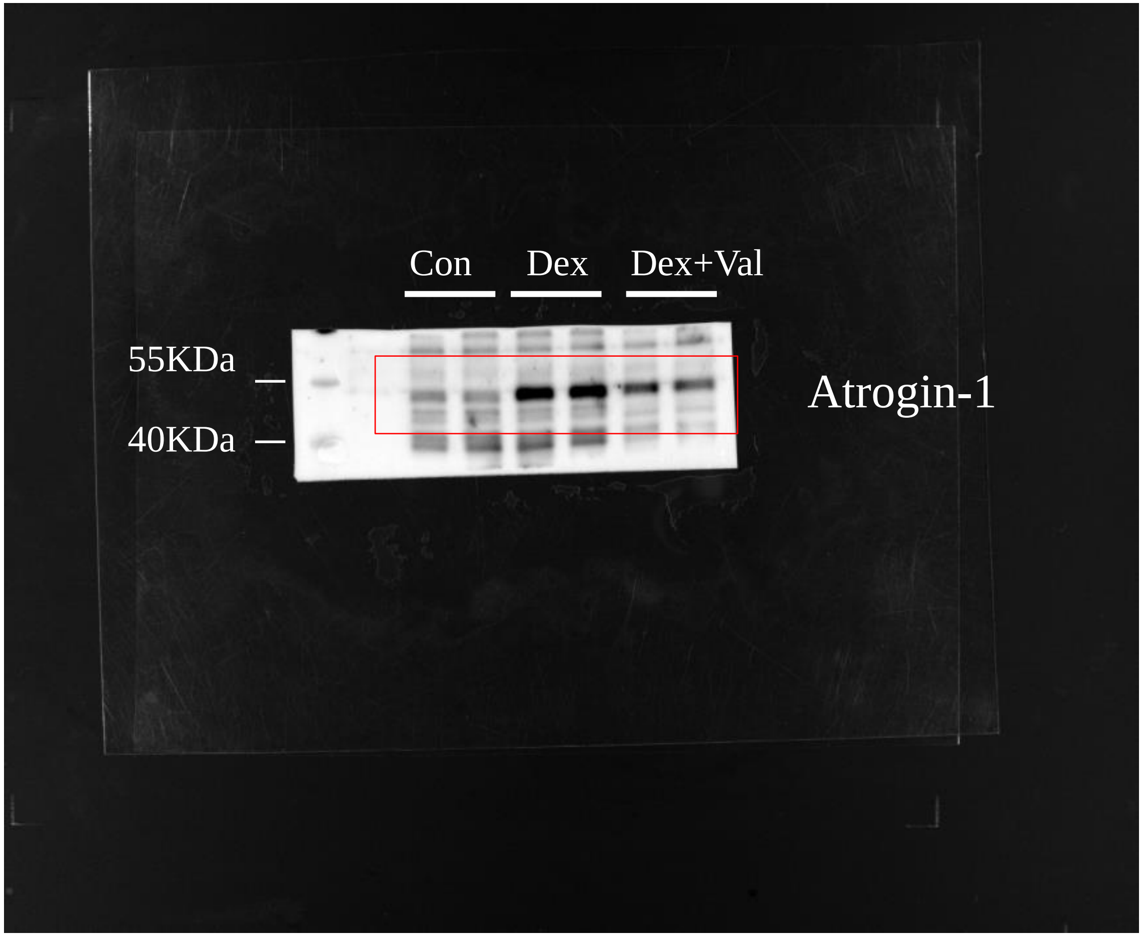

Figure 9E source data

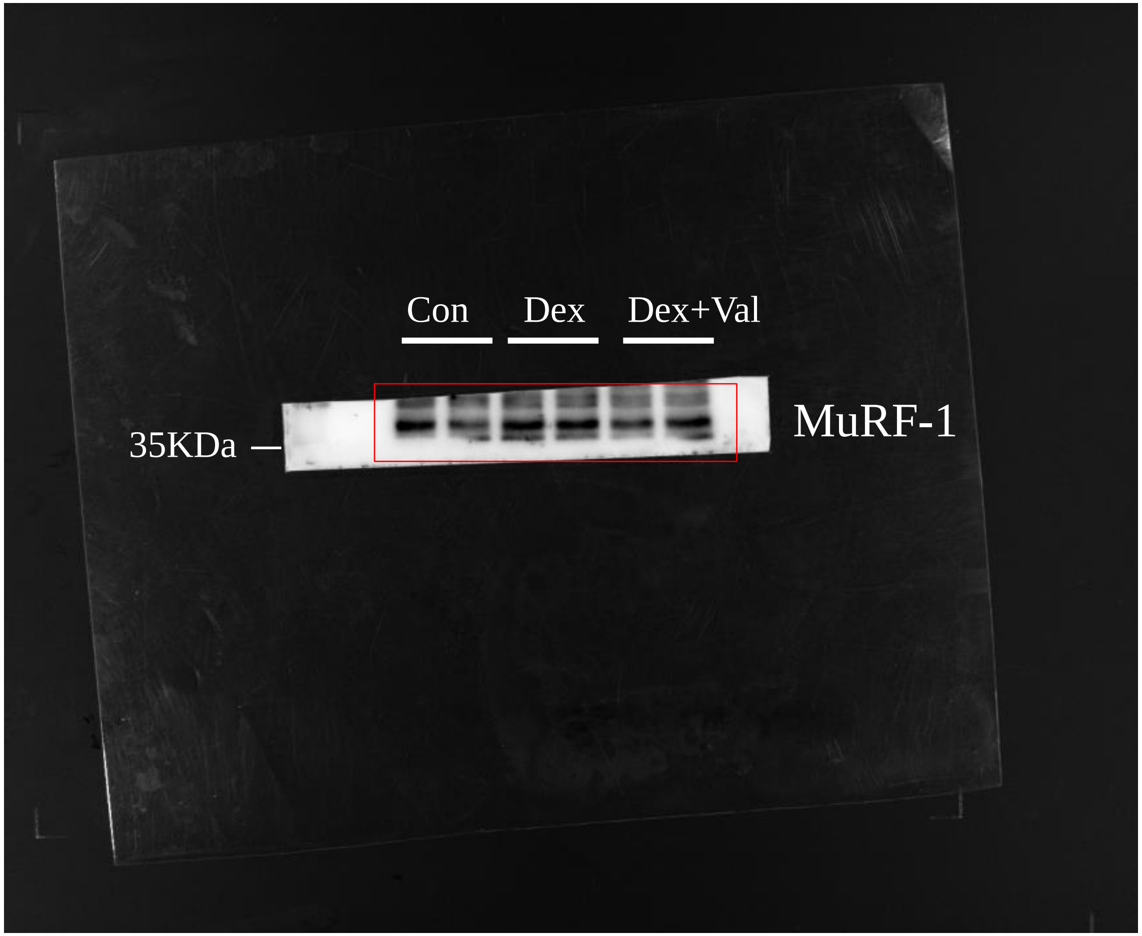

Figure 9E source data

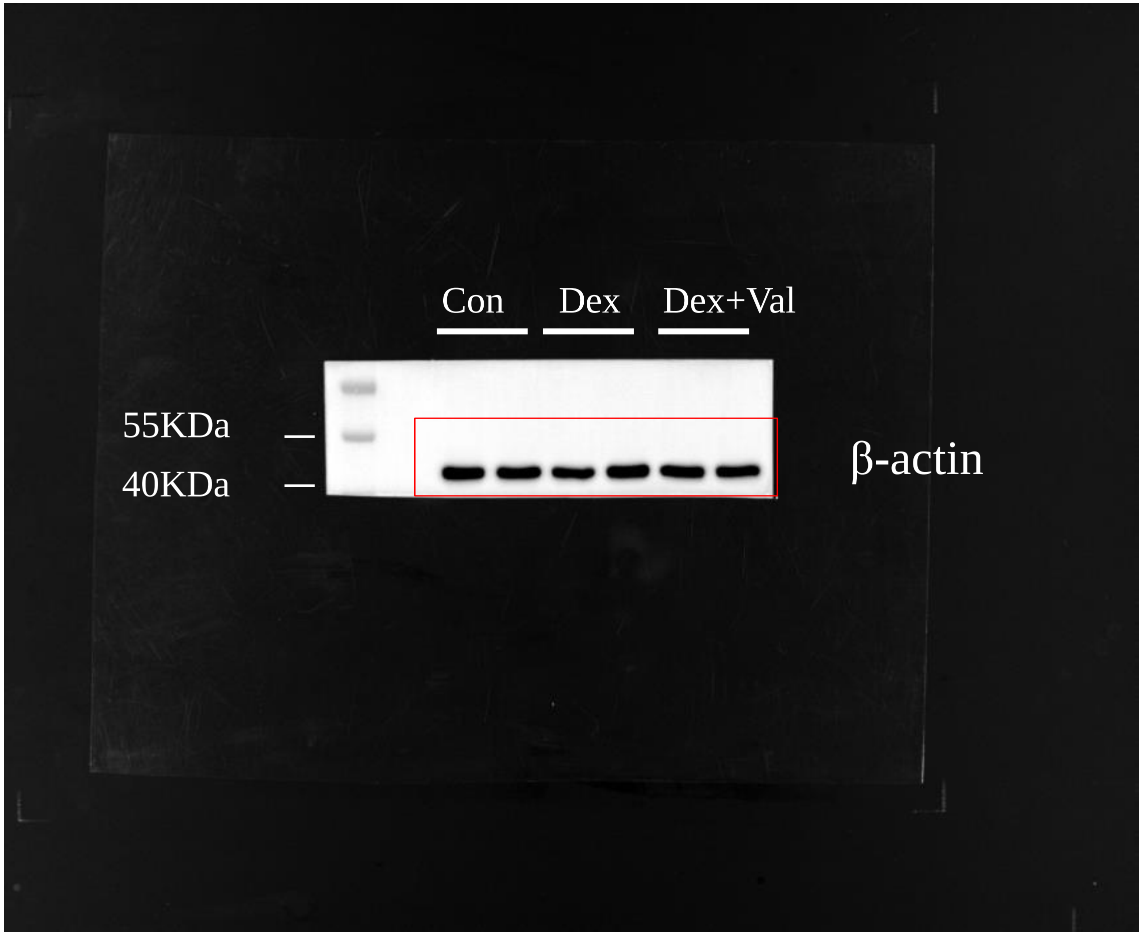

Supplement: Figure 9—source data 3. [file elife-81858-fig9-data3.zip › Figure 9-source data 3/Raw western blot images for Figure 9.pdf]
