# Supplementary material for: Fecal transplant from myostatin deletion pigs positively impacts the gut-muscle axis
Source: eLife. 2023 Apr 11;12:e81858. doi: 10.7554/eLife.81858 (PMC10121221; doi:10.7554/eLife.81858)
Supplement: Supplementary file 1. [file elife-81858-supp1.docx]

**Supporting information for**

Fecal transplant from myostatin deletion pigs positively impacts the gut-muscle axis

Zhao-Bo Luo^2†^, Shengzhong Han^2†^, Xi-Jun Yin^2,3†^, Hongye Liu^2^, Junxia Wang^2^, Meifu Xuan^2^, Chunyun Hao^4^, Danqi Wang^4^, Yize Liu^1^, Shuangyan Chang^2^, Dongxu Li^4^, Kai Gao^2^, Huiling Li^3^, Biaohu Quan^2,3^, Lin-Hu Quan^1^*****, and Jin-Dan Kang^2,3^*****

*Corresponding author

Lin-Hu Quan, Key Laboratory of Natural Medicines of the Changbai Mountain, Ministry of Education, College of Pharmacy, Yanbian University, Yanji, 133002, China, Tel./Fax: +86-433-2436452, Email address: lhquan@ybu.edu.cn.

Jin-Dan Kang, Department of Animal Science, College of Agricultural, Yanbian University; Yanji, 133002, China, Tel./Fax: +86-433-2435623, E-mail address: jdkang@ybu.edu.cn.

**Supplementary File 1**.

Primers sequences used for real-time PCR

| Gene | Primer sequence (5’-3’) | GeneBank accession number |
| --- | --- | --- |
| TJP1 | Forward: AAGCCCTAAGTTCAATCACAATCT | XM_021098896.1 |
|  | Reverse: ATCAAACTCAGGAGGCGGC |  |
| OCLN | Forward: TCCTGGGTGTGATGGTGTTC | XM_005672525.3 |
|  | Reverse: CGTAGAGTCCAGTCACCGCA |  |
| Claudin-1 | Forward: AAACCGTGTGGGAACAACCA | NM_001244539.1 |
|  | Reverse: CACATGAAAATGGCTTCCCTC |  |
| GAPDH | Forward: GCCATCACCATCTTCCAGG | AF017079 |
|  | Reverse: TCACGCCCATCACAAACAT |  |
| FFAR2 | Forward: ATGTAGCCGATGGAAGGAAGAG | NM_146187.4 |
|  | Reverse: CAGCCCATGTCTTCACGGT |  |
| FFAR3 | Forward: TCTTGTATCGACCCCCTGGT | NM_001033316.2 |
|  | Reverse: CTCCTGCGGTCCACTCTTTT |  |
| HCAR2 | Forward: GTGAACCCGATAATAACCGAAGC | NM_030701.3 |
|  | Reverse: TGAGCGGCAATCTCCCTTCT |  |
| Atrogin-1 | Forward: GACTGGACTTCTCGACTGCC | NM_026346.3 |
|  | Reverse: TCAGGGATGTGAGCTGTGAC |  |
| MuRF-1 | Forward: GAGGGGCTACCTTCCTCTCA | NM_001039048.2 |
|  | Reverse: CCAGAGCGTGTCTCACTCAT |  |
| β-actin | Forward: GTACCACCATGTACCCAGGC | NM_007393.5 |
|  | Reverse: AACGCAGCTCAGTAACAGTC |  |
